# One health: a structured review and commentary on trends and themes

**DOI:** 10.1186/s42522-024-00111-x

**Published:** 2024-08-14

**Authors:** Helen Louise Brown, Isabella Grace Pursley, Daniel L. Horton, Roberto M. La Ragione

**Affiliations:** 1 School of Biosciences, Cardiff University, Sir Martin Evans Building, Museum Avenue, Cardiff, CF10 3AX UK; 2 Department of Comparative Biomedical Sciences, School of Veterinary Medicine, University of Surrey, Daphne Jackson Road, Guildford, GU2 7AL UK; 3 Department of Microbial Sciences, School of Biosciences, Edward Jenner Building, University of Surrey, Guildford, GU2 7XH UK

**Keywords:** One health, AMR, Zoonosis, Interpretation, Cross-disciplinary approach, Public health

## Abstract

**Background:**

One Health (OH) is defined as a unifying approach aiming to sustainably balance and optimise the health of people, animals and the ecosystem. It recognises that the health of humans, animals (both domestic and wild), plants and the wider ecosystem are both interdependent and linked. As a concept, it aims to address complex problems requiring input from multiple disciplines. Suitable issues for OH approaches typically include global issues which can widely impact not only the health of humans and animals, but also have a significant environmental impact. Examples include emerging zoonotic diseases and antimicrobial resistance (AMR). Interpretations and use of the term OH differ in the literature and have the potential to dilute its impact. The meaning of OH among the research community has evolved over time. Here, we collate the OH relevant literature from the last two decades, identifying major themes and trends and considering how OH has been embraced differently across various geographical regions.

**Methods and results:**

Bibliographic databases were searched using the term “One Health” AND (“Veterinary” OR “Animal”) AND (“Medicine” OR “Human”) AND (“Environment” OR “Ecosystem”) during the period between 1980 and 2022. Data analysis and narrative synthesis identified themes, similarities, and differences within literature. Web of Science and PubMed returned 948 and 1250 results for the period mentioned above. The predominant literature focused on human health, with veterinary health second, although often to benefit human health. It was found that OH is often utilised as a public health approach, generally towards the end of disease surveillance and control. Interestingly, while authors from low- and middle-income countries were well-represented within studies using the term OH, they were less well-represented as corresponding authors.

**Conclusions:**

The predominant focus of the literature was on human and veterinary health, implying OH approach is human-orientated, despite its suggestion that all domains share a common ‘health’. Potential improvement to OH could be achieved through greater incorporation of the environmental and social sciences for a more encompassing approach.

**Supplementary Information:**

The online version contains supplementary material available at 10.1186/s42522-024-00111-x.

## The background and history to the One Health concept

As the global population expands and becomes ever more industrialised, humans are required to address ever more complex and global issues. The One Health (OH) concept aims to solve complex health issues at the intersection of human, animal, and environmental health by integrating efforts from relevant disciplines across different organisational levels [1]. One Health considers the domains of human, animal, and environmental health as shared, with areas of commonality and each influencing the others by direct and indirect methods. By merging efforts across different fields, OH can allow for the amelioration of issues common between these disciplines whilst reducing detrimental effects to our economy and environment [2]. The definition of One Health has evolved over its history, however, the most recent definition provided by the One Health High-Level Expert Panel considers One Health as an integrated and unifying approach which sustainably balances and optimises the health of people, animals and ecosystems. It recognises that the health of humans, domestic and wild animals, plants and ecosystems are intrinsically linked while also being interdependent [3, 4].

The concept of interdependent health, which is at the core of the OH approach, is not new and has been discovered and rediscovered throughout history. The first mention of OH in its modern form was in 1964. Calvin W. Schwabe, an American veterinarian, argued against compartmentalisation in scientific research, using the term ‘One Medicine’ to represent his own philosophy [5]. He believed that ‘the critical needs of man include the combating of diseases, ensuring enough food, adequate environmental quality, and creation of a society in which human values prevail’. He also considered that medicinal science alone was not an effective method of treatment. The more rounded term “One Health” was then popularised in 2004 through the publication of the 12 Manhattan Principles [6, 7], created by the Wildlife Conservation Society at a conference in New York, USA. The 12 principles urged governing and scientific communities to aspire to a more cross-disciplinary approach, as a necessity in resolving pressing and complex issues that exist at the intersections of health. These issues include antimicrobial resistance (AMR) and emerging zoonotic pathogens, which can be seen drivers at the forefront of OH projects such as the One Health European Joint Programme [8]. Simple solutions may tackle one aspect of a problem, but if other facets belonging to other disciplines are allowed to persist, the problem can reoccur. This was highlighted by the United Kingdom’s Review on Antimicrobial Resistance, produced in the last decade and chaired by Lord Jim O’Neill [9]. The OH approach continues to grow in popularity today, with its usage accelerating in scientific literature, at least in part catalysed through its adoption by organisations such as the World Health Organisation, Food and Agriculture Organisation, and World Organisation for Animal Health [10] and One Health High-Level Expert Panel (OHHLEP) [4]. The OHHLEP was established in May 2021, following discussions across multiple European and global health agencies around how best to support future identification and surveillance of microbes with the potential to cause pandemics. The panel supports agencies by providing evidence-based expert advice, informing future policy and by supporting cooperative activities across national governments. The focus of the OHHLEP is primarily on risk reduction and prevention of zoonotic disease, however, this remit is regularly reviewed, ensuring the continued relevance of the panel to global challenges [4].

One Health also exemplifies the growing trend of holistic approaches that counter the traditional and specialised methods long considered the default in scientific research. These alternative approaches include EcoHealth [11], Planetary Health [12], and conservation medicine [13]. Whilst their origins and aims demarcate them as separate concepts, they share an underlying core belief that different domains of health are interdependent, necessitating a more systematic approach. In tackling complex issues in this way, they claim to nurture innovation of new methods and ideas through integrative work, as well as efficiency through collaborating partners sharing facilities and resources [14]. The divergent aims of these approaches, despite their shared core belief of integration, are a result of their development under different broad categories of science.

Despite the rising popularity of OH, its interpretations and applications differ. These differences impair OH’s potential, diluting its impact and integrative action, and changing the way it is perceived. For instance, Ruegg et al. [2] stated that OH was reactionary to prevalent issues and was distinctly transdisciplinary. Alternatively, Parmley et al. [15] described OH as a concept that focuses on long-term goals, is multidisciplinary, and acts for the ‘surveillance and mitigation of complex public health problems’. These contrasts are not uncommon between OH articles, and so in this review, we investigate the literature to explore the ways in which OH is interpreted, how this has changed over time, and how these interpretations impact OH’s efficacy. The OH literature was previously interrogated in 2015 and the use of the term over time was considered [16], however the recent increase in the OH literature makes further collation and interrogation timely.

In this review, the authors interrogated PubMed and Web of Science databases for papers where OH principles were integral to the work presented. We aimed to understand not only overall themes and trends within the OH field, but also understand how authors interpreted the current, loose, OH definition and applied it within their research.

## Methodology

Searches were conducted using the term “One Health” AND (“Veterinary” OR “Animal”) AND (“Medicine” OR “Human”) AND (“Environment” OR “Ecosystem”) in both the National Library of Medicine PubMed database and Clarivate Web of Science database. Classifications of these three domains were broad – each type of health was considered to pertain to all topics under the umbrella of illnesses and negative impact factors on the wellbeing of either animals, humans, or the environment.

### Primary screening of literature

Primary screening excluded literature that was out of scope to the study or unable to be interpreted. This was determined by scanning titles and abstracts to confirm literature was within the predetermined scope. This included publication between 1980 and 2022, full text available in English and OH implicit or directly referred to within the title and/or abstract. Where literature passed these criteria, it was further manually screened to confirm that OH was integral to the manuscript and its approach.

### Categorisation of retained literature

For all manuscripts included within the primary screen the title and abstract were used to identify the major themes of the Manuscript. One or more of the categories shown in Table 1 were applied to each manuscript, as many terms as necessary were applied to accurately describe the paper as presented by the abstract and title.

**Table 1:** Keywords and their definitions as applied to the primary screening of the manuscripts returned with the defined search string

Category term	Definition of term
**Terms describing the major emphasis of the manuscript**	
Data	Manuscript provides either novel data or a novel synthesis of existing published data
Education	Manuscript focuses on educational interventions or public engagement initiatives
Evaluation	Manuscript provides an evaluation of an existing intervention strategy/initiative/etc.
Opinion	Manuscript presents the authors thoughts on OH, its implementation, success or application to particular challenges
Policy	Manuscript focuses on evaluating implementation of OH initiatives or comments on how its implementation and future use. Implementation can be at any scale (i.e. form local authority to global schemes.
Risk	Manuscript focuses on implementing OH as part of risk analysis or modelling initiatives
**Terms describing the major themes of the manuscript**	
AMR	Major focus of manuscript is antimicrobial resistant organisms or genes
Cancer	Major focus of manuscript is on preventing cancer or understanding links between humans-animal-environmental health which contribute to the development of cancer
Climate	Major focus of manuscript is climate change and its impact on natural or human systems
COVID-19	Major focus of manuscript is on the SARS-CoV-2 outbreak, its impact and/or reach
Non-biological	Major focus of manuscript is on non-biological health impactors. These incorporate pollutants from industry such as heavy metals,
Pathogen	Major focus of manuscript is on a pathogen of interest to either human, animal or environmental systems. The term is used in broadest form to include prokaryotic organisms such as bacteria and viruses (excluding SARS-CoV-2) or complex invertebrate lifeforms including vectors of diseases and parasites. Where the focus of the manuscript is prokaryotic vectors of AMR genes the manuscript was classified as AMR focused not pathogen focused.
**Terms reflecting the major OH domains within the manuscript**	
Domestic	Manuscript focuses on domesticated animals or plants. The term incorporates companion animals and/or pets, agricultural and livestock farming
Environment	The manuscript is focused on environmental sampling and/or consideration of environmental impacts
Human	Manuscript is focused on the human impact of the themes addressed or focuses on human understanding and/or education of themes included
Wild	The manuscript focuses on the impact the major themes will have on non-domesticated vertebrates or invertebrates

### Collection of author country affiliation data

Information was also collected on the country affiliations of authors. For each manuscript, both the corresponding author(s) affiliated countries and those of all additional authors were collected separately. Where more than one author was present from a single country, the total number of authors affiliated with that country were recorded for each paper. Where an author was affiliated to more than one institute within separate countries, they were noted to be affiliated to more than one country. For the corresponding authors, if two or more corresponding authors were present in a manuscript for a single country then each country of affiliation was only recorded once (rather than once per author). This allowed the authors to compile both a list of the total number of authors and their country affiliations and the countries to which each manuscript’s corresponding author was registered. Maps of author affiliations were generated using Microsoft Excel Filled Maps function.

For comparison of the OH data collected with a wider dataset, the Nature Index was used. This Index collates information on a wide range of publications from across the natural and health sciences, including but not limited to publications within Nature journals. Information from the 2023 table for author’s country of origin was used as a comparator to determine if countries within our OH search were over- or under-represented compared to the wider biological sciences fields.

## Results and discussion

### An overview of the OH research field and its general themes and trends following primary screening

**Fig. 1:**
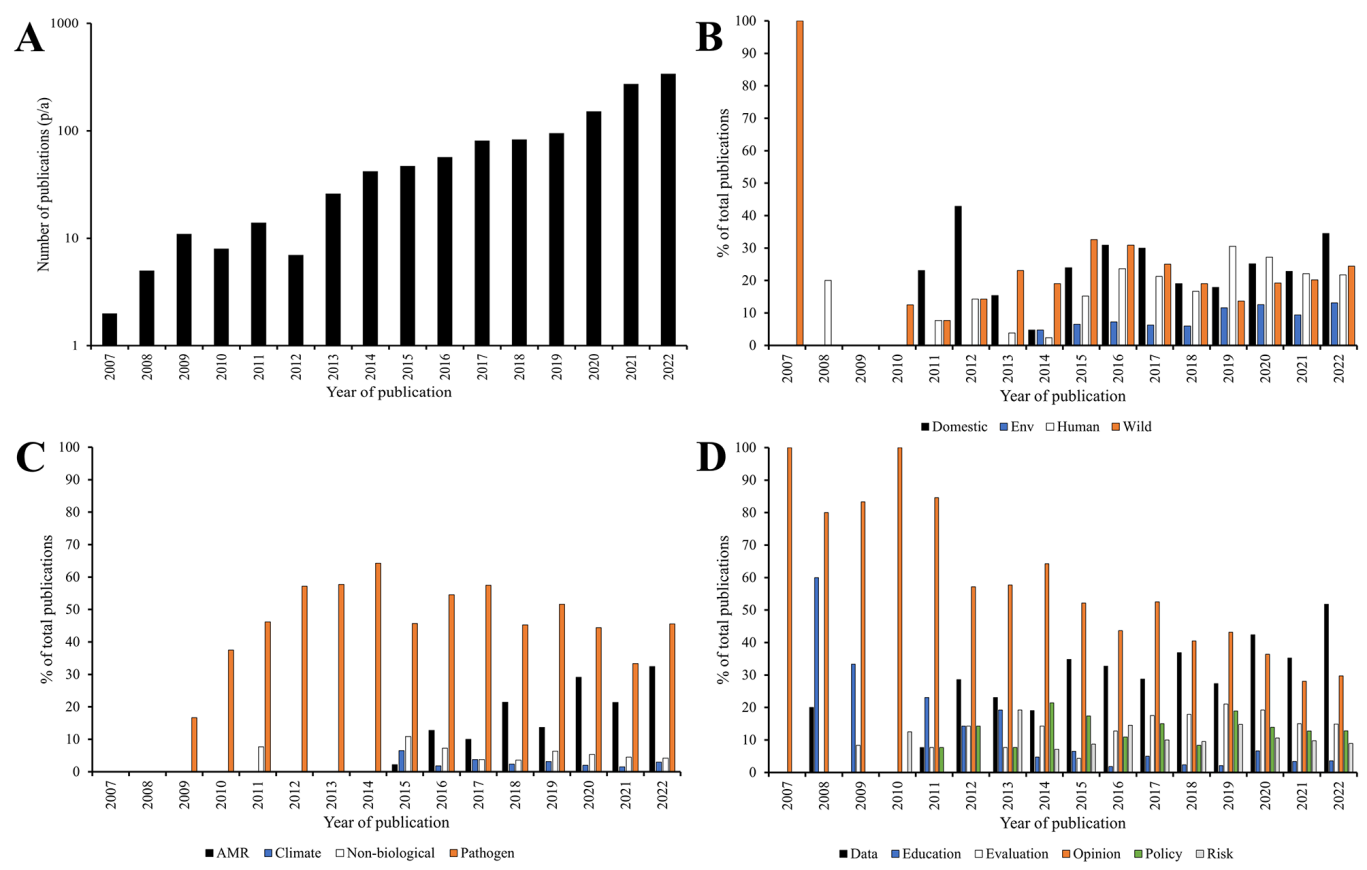
OH publications increase year on year although the major themes of focus are more constant. **A**) the number of manuscripts published in each year using the search terms defined. **B**-**D** each abstract returned by our search was interrogated to identify the major domain (**B**), themes (**C**) and emphasis (**D**) of each manuscript. Where abstracts suggested that the work crossed multiple areas, then as many were included as necessary to fully define the work

A total of 1250 manuscripts were returned via the PubMed search, and 948 via Web of Science. After removal of duplicates, manuscripts not in English or those without a freely available abstract, a total of 1228 individual references were identified and screened as part of the primary screening process. Details of each reference used are presented in Supp. Figure 1. While the number of papers identified by the search string has increased year on year every year since 2012, a marked expansion of the field has occurred since 2019 (Fig. 1a).

Division of the manuscripts by the broad thematic challenges each addressed showed that the OH field is dominated by understanding pathogen transmission between species (Fig. 1b). Where primary data were presented, the research question focused often on the surveillance of a particular organism or population. For example, one study highlighted the diversity and abundance of *Klebsiella spp*. from in and around the Italian city of Pavia [17], an area where carbapenem resistance within the genus has been highlighted as a rising issue [18]. The study found that although *Klebsiella spp.* isolates were readily detected in samples from both wild and domestic animal sources, as well as human and natural environments, significant antibiotic resistance was only detected within hospital settings, highlighting that carbapenem resistance is currently not as widespread as might be assumed. In other studies, surveillance focused on specific animal populations which are known carriers of zoonotic pathogens. Bats were often the focus of surveillance studies, with research focused on the carriage and transmission of viral pathogens such as SAR-CoV-2 and lyssaviruses [19–22]. In all years assessed, a focus on pathogens was the dominant theme of the manuscripts, although from 2015 onwards a trend can be seen showing that AMR is becoming increasingly common (Fig. 1C). It is interesting to note that this trend in increased interest coincided with the commissioning of the UK O’Neill report [23, 24] investigating the global social and economic impact that AMR is expected to have in the future.

The majority of papers returned focused on either presenting research data which could be incorporated into OH research, or providing comment on the OH field, the relevance of using an OH approach to address specific challenges, or the implementation of OH ideas (Fig. 1D).

### One health is frequently framed using an anthropocentric viewpoint

In the literature, OH is consistently applied with an anthropocentric approach to solve our own health issues. When veterinary, environmental and ecology sciences are integrated in studies, they are often utilised as a means of addressing issues impacting human health. Many studies focused on the transmission of zoonotic agents between humans and animals, typically domestic animals which are frequently in close contact with humans (examples include [25–28]. Interestingly even companion animal welfare was considered through this anthropocentric lens, with health of companion animals considered to be reflective of the owners physical and mental wellbeing and/or happiness [29–31]. This anthropocentric focus may alienate and lead to underfunding of non-human disciplines, an issue which is further discussed in two of the manuscripts returned by our search [32, 33] and also highlighted as an issue by OHHLEP, who noted that future OH actions must be effective, fair, equitable and sustainable. The challenge of reaching equity between sectors and disciplines is embedded within the foundational principles of the panel [4].

Despite the dominant focus of humans there are some notable examples within the body of literature addressing how pathogen transmission between animals is of detriment to wild animal populations. Two manuscripts within our search focused on the fungal pathogens of wild amphibian species and their role in the decline of global amphibian numbers [34, 35]. Neither the fungal pathogens nor the frogs themselves are of direct significance to human health, but the decline of the amphibian populations will significantly impact the local ecosystems in which they exist. Although both the papers focused on animal welfare and environmental impact, the point is made that human activity is indirectly contributing to the spread of the pathogens and should therefore be considered as part of a solution to limit disease spread.

### The incorporation of OH into veterinary, medical and public health education is an essential aspect of developing truly sustainable OH projects

Evaluation and implementation of, as well as comment on, OH initiatives was also a common theme detected within our data set. Interestingly, many of the earlier OH-related manuscripts returned by our search focused on upskilling both medical and veterinary students, centering on the description and evaluation of OH-related teaching. It is interesting to note that despite the anthropocentric focus of many of the OH studies identified within our research, a recent survey of medical schools within the US indicated that only 56% of the courses included an OH component [36]. This is in contrast to veterinary education, where OH is seen to be an essential aspect of the curriculum for veterinary students, with several authors stressing the importance of OH education as early 2009 [37–40]. A 2012 survey of veterinary students by Wong and Kogan [38] indicated that although OH teaching was still not an integral aspect of the curriculum for 65.6% of the respondents, 74.2% of them were familiar with OH and 80% considered it to be important for public health.

One Health education is also now available to a wider audience, with several OH-focused Massive Open Online Courses (MOOC) described within our captured literature. Ruiz de Castañeda et al. [41] described and evaluated a five-day interdisciplinary workshop associated with attendance of MOOCs developed by Swiss educators. The workshop incorporated a “Hackathon” approach [42] to address four projects. The majority of participants rated the experience positively, emphasising that the involvement of multiple discipline experts was helpful, and that the involvement of computing experts was essential to the success of the workshop. The same MOOC was also utilised to support university-level education within a Kenyan refugee camp. Students accessing the MOOC also worked on research projects with researchers from the University of Geneva in Kakuma. This project not only supported the continued learning of those displaced but also provided academic collaborators with local knowledge and expertise, essential to the success of interventions [43].

### AMR and zoonotic pathogen transmission are the major health concerns addressed by researchers currently using one health

As might be expected from the humancentric approach many manuscripts took, the zoonotic transmission of bacterial, viral and fungal pathogens or multicellular parasites from animal (both domesticated and wild) to human populations was a particularly common theme within the manuscripts. A secondary, but related area, the persistence and/or transmission of antimicrobial-resistant organisms and/or genes also increased in interest over time. The link between AMR and OH has been explored in detail by [44].

Although many papers captured within the “pathogen” keyword criterion considered viral outbreaks attributed to SARS-CoV viruses [45, 46] it was not until 2022 that over 5% ofthe manuscripts per annum contained substantive relevance to COVID-19 (Fig. 1b). Some authors also chose an OH approach to address a wide range of health issues. For example, an increasing number of authors chose to consider the use of OH approaches when investigating the link between climate change and health [47–63]. In contrast, a small number of papers addressed other health issues such as cancer [64, 65] and obesity [29, 30, 66–68]. It was also interesting to note that while many studies focused on reporting prevalence of AMR and/or zoonotic pathogens, similar approaches were also used to detect other toxic products such as heavy metals [69–76].

### Assessment of author affiliations suggests that low- and middle-income countries are well represented, but a wider degree of global collaboration in each study is required

**Fig. 2:**
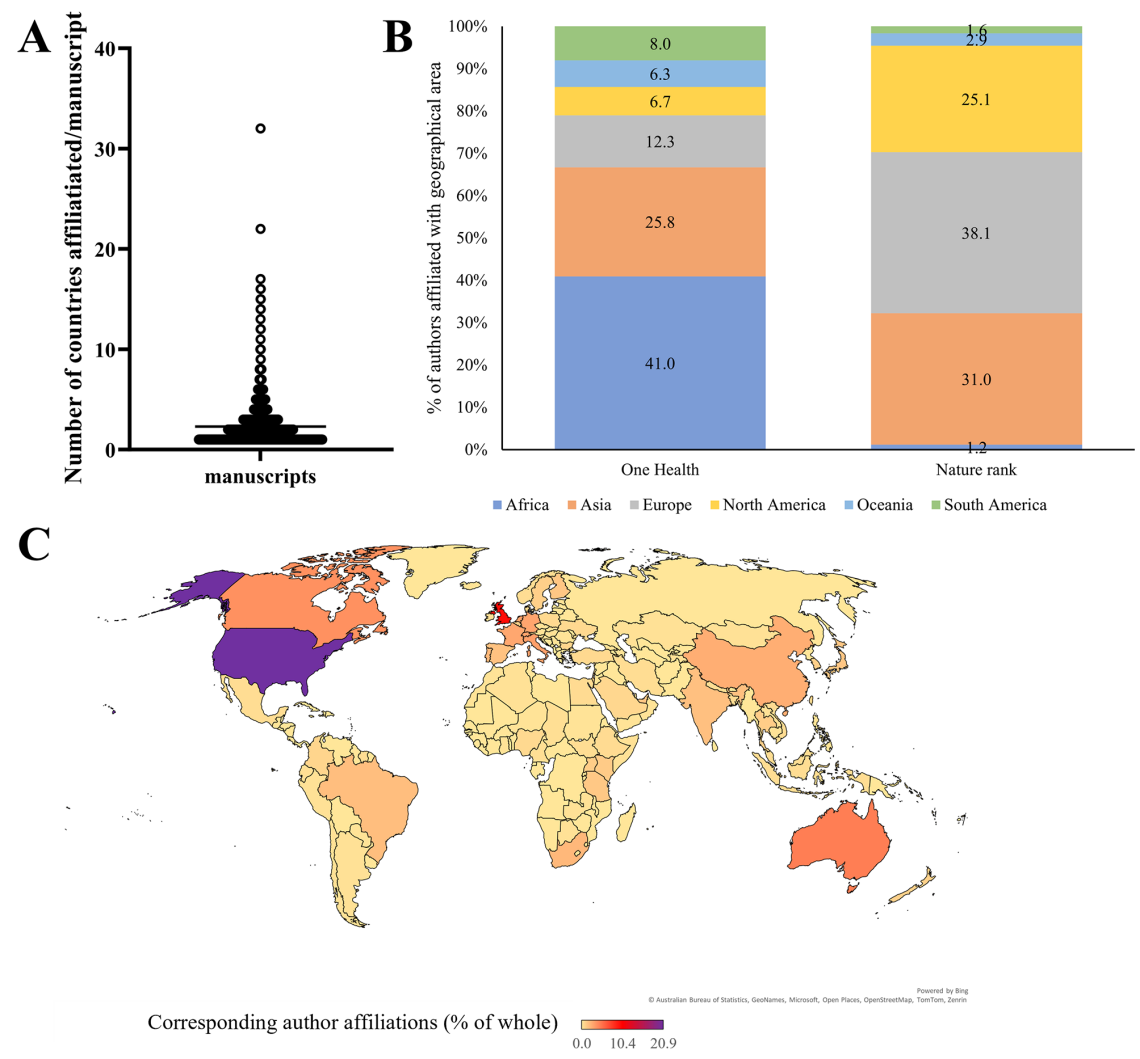
OH manuscripts have a low number of authors, but low- and middle-income countries are well-represented. The number of authors affiliated with each manuscript was tracked to indicate how many contributors might be expected for OH research (**A**). (**B**) The percentage of total authors affiliated with each continent of both manuscripts within our collection (left-hand stacked bar) and the Nature Index (right-hand stacked bar) is also shown, highlighting differences between the OH field and wider natural/health sciences. Finally, (**C**) is a global map showing the percentage of corresponding authors (as a total of all the OH manuscripts) affiliated with each country

We observed that almost half of the manuscripts (47.3%) only contained a single country affiliation (Fig. 2a). The mean number of countries affiliated with each manuscript was 2.2 (median affiliation was 2), with a range of between one and 32 countries. Of the 912 papers screened for country affiliation, only 13 manuscripts had more the 10 countries affiliated with the manuscript. In total 131 countries were represented in the collection.

The US was the dominant country for authors to belong to, with 19% of all authors affiliated with the US (supplementary Fig. 2). European nations were well represented among the top-ranked countries, with the UK (14%), Netherlands (5.3%), Italy (4.9%), France (4.5%), Germany (4.3%) and Switzerland (3%) all appearing in the top 10 ranked countries by number of author affiliations per manuscript.

Comparing the country affiliations of the authors returned by our search and comparing them to information provided within the 2023 country/territory ranking tables in the Nature Index (NI) [77, 78] showed some interesting dissimilarities between the field of biosciences as a whole and manuscripts returned by our search. NI shows that the biosciences field (when measured by the number of manuscripts) is dominated by authors affiliated to institutes within China and the US, followed by Germany, UK and Japan in the top five ranks. Interestingly, while the US was ranked first within our OH collection, China was not so dominant, ranking ninth in our OH ranks. When considering individual nations, nations in Europe and the US were highly ranked, with a large number of authors affiliated with institutes within these countries. However, countries with a smaller research presence (as indicated by the NI) were much more highly represented in our collection. Notable examples include Brazil (11th in the OH rank but 24th in NI), Nigeria (12th in the OH rank but 71st in NI) and St Kitts and Nevis (ranked 35th in OH but 167 in NI). Where we compiled information to reflect affiliation to continents rather than countries, it became apparent that citations from authors in Africa, Oceania and South America were much more common in our OH collection than in the NI, with European authors much less well represented (Fig. 2b). The authors note that their search was restricted to English language papers and as such this may impact the range of manuscripts scrutinised and the country of origin of the authors.

Since many of the OH studies within our collection described interventions or sampling undertaken in Low and Middle-Income Countries (LMIC) it is reassuring that in many cases local authors were included in the manuscripts. Mumford et al. [79] note in that for OH initiatives to be successfully implemented and sustained there is a requirement for the incorporation of both local and global worldviews alongside required methodological expertise. The importance of global viewpoints was highlighted in several studies within our search. One study focusing on reducing soil-transmitted helminth infections in Maasai pastoralists, surveyed Maasai communities to better understand their knowledge of parasite transmission and the intervention strategies implemented [80]. The study found that many communities considered the building of latrines to be unhelpful despite it being a standard intervention strategy. Latrines were not only seen to be a potential danger to livestock and children, but many felt that using the latrine, rather than the land around the villages, announced to the wider community the individuals need to defecate, causing embarrassment. Enforcement of the latrine policy by fining households without latrines was also seen as punitive, discouraging communities from reporting infections to the public health authority and leading to the building of “false latrines” to avoid fines being imposed. Without the collaborative input of researchers from both Tanzania and Canada, it is unlikely that such a complete and honest picture of the issues would have been developed, with further interventions potentially being as unhelpful as the current policies.

### Corresponding authors are still mostly affiliated to high-income countries within the global north

Within the OH manuscript collection, corresponding authors were affiliated with 107 individual countries. The United States of America (US) dominated the corresponding author affiliations, accounting for 21% of all the affiliations. This was followed by the United Kingdom (10%), Australia (5%), Canada (4%) and Italy (3%). Interestingly, China is only responsible for 2.5% of all the corresponding author affiliations, surprising as China is so dominant in the NI. Several recent zoonotic diseases of interest to human health (including bird flu, SARS and the closely related COVID-19) have also been found to have a foothold in China and/or countries within its sphere of influence [81–83]. As such, the authors expected that OH approaches would be commonly included as part of wider control and surveillance strategies. It must however be noted that the search included only English language manuscripts, so manuscripts published in other languages are not captured as part of this work. This may contribute to the reduced profile of Chinese-affiliated authors within our search results.

### The use and impact of terms referring to different collaboration frameworks

An integral part of OH is the promotion of collaboration. A variety of different terms are used to describe this throughout the literature, often interchangeably. However, while they are often conflated, multidisciplinary, interdisciplinary, and transdisciplinary are not the same concept and their use implies different collaboration frameworks [84]. Multidisciplinary collaboration draws knowledge from multiple disciplines while maintaining separation between them. These disciplines are included in the same project, but their methods and ideas are not integrated. Interdisciplinary collaboration requires the harmonization of different disciplines in a coordinated manner, collaborating across boundaries without their removal. Transdisciplinary collaboration represents a more complete integration, transcending traditional boundaries between branches of knowledge to create ideas and methods in what can be considered a ‘new’ approach [85].

Whilst used interchangeably by most, some manuscripts attempted to clarify which of these forms of collaboration an OH approach must subscribe to, leading to a contradiction between manuscripts. In ‘A Blueprint to Evaluate One Health’ [2], the authors specify that OH represents a shift from interdisciplinary approaches to a transdisciplinary one “that integrates society and science by including all stakeholders”. This shift was also described in another article ‘Need for Enhanced Environmental Representation in the Implementation of One Health’ [86]. In contrast, other papers suggested that OH is a concept that is meant to nurture and promote interdisciplinary collaboration [87, 88].

### Careful consideration of the operational levels on which each initiative focuses is essential

As shown in Fig. 1D, a number of the manuscripts returned by our search considered OH from a policy perspective. The manuscripts collected within the “policy” key term were varied, ranging from description of frameworks which can be used to implement OH initiatives [89–91] to description and evaluation of current initiatives [92, 93]. Many of the manuscripts focused on the engagement of high-level stakeholders such as government and/or international health and environmental bodies, with fewer interacting with local communities. Engagement of stakeholders with the power to influence national and international policy is essential both for funding and sustainability of long-term OH projects. Indeed, several authors commented on the essentiality of leadership when tackling wicked problems [94, 95] However, wider engagement with public and community bodies must also be carefully considered for OH initiatives to be truly transformative. We note above, examples of teaching and public engagement initiatives which have begun to raise awareness of OH and its potential, particularly within veterinary medicine, however further work is needed to truly embed OH frameworks.

Without the collaboration and knowledge of local communities, many projects are missing a valuable opportunity to gain a more detailed image of the problems they seek to resolve from the people who are affected. The public is an important stakeholder in the formulation and successful implementation of any solution, and the priorities of the voting public are considered by their elected officials [96]. In this sense, groundwork with local representatives is significant and should be considered as an essential aspect of gaining policymaker support. Working with local representatives, such as in the paper by Henderson et al. [80] shows how grassroots interactions can also allow for a sustained response that prevents an issue persisting, whilst larger projects often treat the symptoms of an issue.

Successful engagement of a wide range of public and policy stakeholders has garnered success for initiatives in other disciplines. Conservation initiatives have been particularly successful collaborations between public, governmental and international partners. Conservation initiatives must take place on macro-, meso- and microscales [97] meaning that success can only be possible if wide support is available [98, 99]. The recent conservation success of sea turtles [100], required complex international cooperation. Similarly, the successful conservation of pandas [101] has been driven by both public and governmental support in China. AMR and zoonosis may not yet have the same position in public awareness as these iconic animals, but this is beginning to change [102] and OH must capitalise on this while maintaining their efforts with national and international government bodies.

## Conclusion/Future perspective

Although popularity of OH is increasing year on year, as indicated in Fig. 1A, The OH field maintains its diversity. While the majority of the authors within our search strongly supported OH as a concept there was significant variation in interpretation of how OH should be utilised to create the greatest impact. Currently, OH has a strong anthropocentric focus, despite the veterinary field being particularly well-represented within our search. Many of the initiatives captured also recognised the need to engage stakeholders outside of traditional academic research fields, including policymakers and local communities, although truly global engagement resulting in an international impact still seems to be an ambition rather than a truism. Our data suggests that much of the control of OH initiatives is still held by high-income nations in the global north, although researchers from LMIC are better represented within our data set than might be expected within health and natural sciences. Since many of the top global public health threats (such as AMR and emerging zoonoses), that OH supports are prevalent in the global south, it is essential that these countries are given greater agency. As such, it is encouraging that newly established OH initiatives, such as the OHHLEP, have included equity between sectors and disciplines, sociopolitical and multicultural parity, and socioecological equilibrium within their foundational principles [4]. Without this parity, and truly global collaboration and cooperation, it is unlikely that OH programmes will be able to reach their full potential.

### Electronic supplementary material

Below is the link to the electronic supplementary material.

Supplementary Material 1

## Data Availability

The datasets used and/or analysed during the current study are available in Supp. Figure 1. Data used in further analysis is available via the corresponding author upon reasonable request. Search strings for both Web of Science and PubMed were archived via searchRxiv [103, 104].
