# Supplementary material for: One health: a structured review and commentary on trends and themes
Source: One Health Outlook. 2024 Aug 14;6:17. doi: 10.1186/s42522-024-00111-x (PMC11323492; doi:10.1186/s42522-024-00111-x)
Supplement: Supplementary file 1 — Supplementary Material 1 [file 42522_2024_111_MOESM1_ESM.pdf]

SourceType	Title	Year	Pages	JournalName	Volume	Issue	Tag	Edition	Month	Publisher
Journal Article	Global rabies control: the role of international organisations and the Global Strategic Plan to eliminate dog-mediated human rabies	2018	741-749	Revue Scientifique Et Technique-Office International Des Epizooties	37	2	domestic--eval--pathogen--global-rabies-control:-the-role-of-international-organisations-and-the-global-strategic-plan-to-eliminate-dog-mediated-human-rabies			
Journal Article	Echinococcosis in Tambool, Central Sudan: a knowledge, attitude and practice (KAP) study	2018	490-494	International Health	10	6	data--human--pathogen--echinococcosis-in-tambool,-central-sudan:-a-knowledge,-attitude-and-practice-(kap)-study			
Journal Article	Are we adequately evaluating subsidized veterinary services? A scoping review	2018	59-69	Prev Vet Med	157		data--eval--are-we-adequately-evaluating-subsidized-veterinary-services?-a-scoping-review	09/08/2018		
Journal Article	Antimicrobial resistance: A round table discussion on the "One Health" concept from the Gulf Cooperation Council Countries. Part One: A focus on Leadership	2018	771-777	J Infect Public Health	11	6	amr--eval--opinion--antimicrobial-resistance:-a-round-table-discussion-on-the-"one-health"-concept-from-the-gulf-cooperation-council-countries.-part-one:-a-focus-on-leadership	07/11/2018		
Journal Article	Use of RFID technology to characterize feeder visitations and contact network of hummingbirds in urban habitats	2018	e0208057	PLoS One	13	12	data--wild--use-of-rfid-technology-to-characterize-feeder-visitations-and-contact-network-of-hummingbirds-in-urban-habitats	13/12/2018		
Journal Article	Strengthening One Health Through Investments in Agricultural Preparedness	2018	92-107	Health Security	16	2	eval--opinion--pathogen--strengthening-one-health-through-investments-in-agricultural-preparedness			
Journal Article	Management of arthropod vector data - Social and ecological dynamics facing the One Health perspective	2018	80-91	Acta Tropica	182		data--pathogen--risk--management-of-arthropod-vector-data---social-and-ecological-dynamics-facing-the-one-health-perspective			
Journal Article	Mosquito control with green nanopesticides: towards the One Health approach? A review of non-target effects	2018	10184-10206	Environ Sci Pollut Res Int	25	11	opinion--pathogen--mosquito-control-with-green-nanopesticides:-towards-the-one-health-approach?-a-review-of-non-target-effects	30/07/2017		
Journal Article	Health workers' knowledge of zoonotic diseases in an endemic region of Western Uganda	2018	850-858	Zoonoses Public Health	65	7	data--human--pathogen--health-workers'-knowledge-of-zoonotic-diseases-in-an-endemic-region-of-western-uganda	05/08/2018		
Journal Article	Contribution of veterinary microbiology to the 'One Health' idea	2018	95-105	Postepy Mikrobiologii	57	2	opinion--pathogen--contribution-of-veterinary-microbiology-to-the-'one-health'-idea			
Journal Article	Defining the risk landscape in the context of pathogen pollution: Toxoplasma gondii in sea otters along the Pacific Rim	2018	171178	R Soc Open Sci	5	7	pathogen--risk--wild--defining-the-risk-landscape-in-the-context-of-pathogen-pollution:-toxoplasma-gondii-in-sea-otters-along-the-pacific-rim	16/08/2018		
Journal Article	One Health (r)Evolution: Learning from the Past to Build a New Future	2018		Viruses	10	12	opinion--one-health-(r)evolution:-learning-from-the-past-to-build-a-new-future	21/12/2018		
Journal Article	A Qualitative Stakeholder Analysis of Avian Influenza Policy in Bangladesh	2018	63-71	Ecohealth	15	1	eval--pathogen--policy--a-qualitative-stakeholder-analysis-of-avian-influenza-policy-in-bangladesh	15/11/2017		
Journal Article	Occurrence of Staphylococcus aureus in swine and swine workplace environments on industrial and antibiotic-free hog operations in North Carolina, USA: A One Health pilot study	2018	88-96	Environ Res	163		data--domestic--pathogen--occurrence-of-staphylococcus-aureus-in-swine-and-swine-workplace-environments-on-industrial-and-antibiotic-free-hog-operations-in-north-carolina,-usa:-a-one-health-pilot-study	13/02/2018		
Journal Article	Endemic and emerging acute virus infections in Indonesia: an overview of the past decade and implications for the future	2018	487-503	Critical Reviews in Microbiology	44	4	data--human--pathogen--risk--endemic-and-emerging-acute-virus-infections-in-indonesia:-an-overview-of-the-past-decade-and-implications-for-the-future			
Journal Article	The One Health Concept: 10 Years Old and a Long Road Ahead	2018	13	Frontiers in Veterinary Science	5		opinion--the-one-health-concept:-10-years-old-and-a-long-road-ahead			
Journal Article	Biofilms and antibiotic susceptibility of multidrug-resistant bacteria from wild animals	2018	20	PeerJ	6		amr--data--pathogen--wild--biofilms-and-antibiotic-susceptibility-of-multidrug-resistant-bacteria-from-wild-animals			
Journal Article	Molecular relatedness of ESBL/AmpC-producing Escherichia coli from humans, animals, food and the environment: a pooled analysis	2018	339-347	Journal of Antimicrobial Chemotherapy	73	2	amr--data--domestic--human--wild--molecular-relatedness-of-esbl/ampc-producing-escherichia-coli-from-humans,-animals,-food-and-the-environment:-a-pooled-analysis			
Journal Article	Methicillin-resistant Staphylococcus aureus: livestock-associated, antimicrobial, and heavy metal resistance	2018	2497-2509	Infect Drug Resist	11		amr--domestic--opinion--methicillin-resistant-staphylococcus-aureus:-livestock-associated,-antimicrobial,-and-heavy-metal-resistance	18/12/2018		
Journal Article	Systems Thinking in Practice: Participatory Modeling as a Foundation for Integrated Approaches to Health	2018	303	Front Vet Sci	5		opinion--systems-thinking-in-practice:-participatory-modeling-as-a-foundation-for-integrated-approaches-to-health	09/01/2019		
Journal Article	Brucellosis in the Addis Ababa dairy cattle: the myths and the realities	2018	396	BMC Vet Res	14	1	data--domestic--pathogen--brucellosis-in-the-addis-ababa-dairy-cattle:-the-myths-and-the-realities	15/12/2018		
Journal Article	A scoping review on the prevalence of Shiga-toxigenic Escherichia coli in wild animal species	2018	911-920	Zoonoses Public Health	65	8	data--pathogen--wild--a-scoping-review-on-the-prevalence-of-shiga-toxigenic-escherichia-coli-in-wild-animal-species	14/08/2018		
Journal Article	Tularemia in Germany-A Re-emerging Zoonosis	2018	40	Front Cell Infect Microbiol	8		opinion--pathogen--tularemia-in-germany-a-re-emerging-zoonosis	06/03/2018		
Journal Article	The impact of human activities and lifestyles on the interlinked microbiota and health of humans and of ecosystems	2018	1018-1038	Sci Total Environ	627		opinion--the-impact-of-human-activities-and-lifestyles-on-the-interlinked-microbiota-and-health-of-humans-and-of-ecosystems	11/02/2018		
Journal Article	One Health-ness Evaluation of Cysticercosis Surveillance Design in Portugal	2018	74	Front Public Health	6		eval--one-health-ness-evaluation-of-cysticercosis-surveillance-design-in-portugal	30/03/2018		
Journal Article	What are the effective solutions to control the dissemination of antibiotic resistance in the environment? A systematic review protocol	2018	9	Environmental Evidence	7	1	amr--env--eval--wild--what-are-the-effective-solutions-to-control-the-dissemination-of-antibiotic-resistance-in-the-environment?-a-systematic-review-protocol			
Journal Article	Heads in the sand: public health and ecological risks of lead-based bullets for wildlife shooting in Australia	2018	287-306	Wildlife Research	45	4	non-bio--opinion--heads-in-the-sand:-public-health-and-ecological-risks-of-lead-based-bullets-for-wildlife-shooting-in-australia			
Journal Article	A One Health Evaluation of the Southern African Centre for Infectious Disease Surveillance	2018	16	Frontiers in Veterinary Science	5		eval--a-one-health-evaluation-of-the-southern-african-centre-for-infectious-disease-surveillance			
Journal Article	Semi-quantitative assessment of disease risks at the human, livestock, wildlife interface for the Republic of Korea using a nationwide survey of experts: A model for other countries	2018	e155-e164	Transboundary and Emerging Diseases	65	1	opinion--pathogen--wild--semi-quantitative-assessment-of-disease-risks-at-the-human,-livestock,-wildlife-interface-for-the-republic-of-korea-using-a-nationwide-survey-of-experts:-a-model-for-other-countries			
Journal Article	Planning a One Health Case Study to Evaluate Methicillin Resistant Staphylococcus aureus and Its Economic Burden in Portugal	2018	9	Frontiers in Microbiology	9		amr--opinion--risk--planning-a-one-health-case-study-to-evaluate-methicillin-resistant-staphylococcus-aureus-and-its-economic-burden-in-portugal			
Journal Article	Characterization of the clonal subpopulation Fiocruz L1-130 of Leptospira interrogans in rats and dogs from Brazil	2018	1361-1367	J Med Microbiol	67	9	data--domestic--pathogen--wild--characterization-of-the-clonal-subpopulation-fiocruz-l1-130-of-leptospira-interrogans-in-rats-and-dogs-from-brazil	31/07/2018		
Journal Article	The challenges of implementing an integrated One Health surveillance system in Australia	2018	e229-e236	Zoonoses Public Health	65	1	opinion--pathogen--the-challenges-of-implementing-an-integrated-one-health-surveillance-system-in-australia	12/12/2017		
Journal Article	The growth and strategic functioning of One Health networks: a systematic analysis	2018	e264-e273	Lancet Planet Health	2	6	eval--policy--the-growth-and-strategic-functioning-of-one-health-networks:-a-systematic-analysis	09/06/2018		
Journal Article	Revisiting Polymorphic Diversity of Aminoglycoside N-Acetyltransferase AAC(6 ')-Ib Based on Bacterial Genomes of Human, Animal, and Environmental Origins	2018	7	Frontiers in Microbiology	9		amr--data--revisiting-polymorphic-diversity-of-aminoglycoside-n-acetyltransferase-aac(6-')-ib-based-on-bacterial-genomes-of-human,-animal,-and-environmental-origins			
Journal Article	Zoonotic Transfer of Clostridium difficile Harboring Antimicrobial Resistance between Farm Animals and Humans	2018		J Clin Microbiol	56	3	data--domestic--human--pathogen--zoonotic-transfer-of-clostridium-difficile-harboring-antimicrobial-resistance-between-farm-animals-and-humans	15/12/2017		
Journal Article	Understanding the Complexities of Food Safety Using a "One Health" Approach	2018		Microbiol Spectr	6	1	opinion--understanding-the-complexities-of-food-safety-using-a-"one-health"-approach	17/02/2018		
Journal Article	Pathogens collections, biobanks and related-data in a One Health legal and ethical perspective	2018	688-696	Parasitology	145	5	pathogen--policy--pathogens-collections,-biobanks-and-related-data-in-a-one-health-legal-and-ethical-perspective			
Journal Article	Investigation of silver (Ag) deposition in tissues from stranded cetaceans by autometallography (AMG)	2018	534-545	Environ Pollut	235		data--non-bio--wild--investigation-of-silver-(ag)-deposition-in-tissues-from-stranded-cetaceans-by-autometallography-(amg)	13/01/2018		
Journal Article	Long-Term Colonization Dynamics of Enterococcus faecalis in Implanted Devices in Research Macaques	2018		Appl Environ Microbiol	84	18	data--pathogen--wild--long-term-colonization-dynamics-of-enterococcus-faecalis-in-implanted-devices-in-research-macaques	15/07/2018		
Journal Article	Institutionalizing One Health: From Assessment to Action	2018	S37-S43	Health Security	16		opinion--institutionalizing-one-health:-from-assessment-to-action			
Journal Article	Understanding Seasonal Changes to Improve Good Practices in Livestock Management	2018	175	Front Public Health	6		data--domestic--understanding-seasonal-changes-to-improve-good-practices-in-livestock-management	03/07/2018		
Journal Article	Long term trends and spatial distribution of animal bite injuries and deaths due to human rabies infection in Uganda, 2001-2015	2018	e0198568	PLoS One	13	8	data--domestic--human--pathogen--risk--wild--long-term-trends-and-spatial-distribution-of-animal-bite-injuries-and-deaths-due-to-human-rabies-infection-in-uganda,-2001-2015	22/08/2018		
Journal Article	Antimicrobial Resistance: a One Health Perspective	2018	26	Microbiology Spectrum	6	2	amr--opinion--antimicrobial-resistance:-a-one-health-perspective			
Journal Article	Ecosystem change and zoonoses in the Anthropocene	2018	755-765	Zoonoses and Public Health	65	7	opinion--pathogen--risk--ecosystem-change-and-zoonoses-in-the-anthropocene			
Journal Article	Antimicrobial resistance in Chile and The One Health paradigm: Dealing with threats to human and veterinary health resulting from antimicrobial use in salmon aquaculture and the clinic	2018	299-303	Revista Chilena De Infectologia	35	3	amr--domestic--human--opinion--wild--antimicrobial-resistance-in-chile-and-the-one-health-paradigm:-dealing-with-threats-to-human-and-veterinary-health-resulting-from-antimicrobial-use-in-salmon-aquaculture-and-the-clinic			
Journal Article	Evaluation of metrics for benchmarking antimicrobial use in the UK dairy industry	2018	379-+	Veterinary Record	182	13	eval--evaluation-of-metrics-for-benchmarking-antimicrobial-use-in-the-uk-dairy-industry			
Journal Article	Epidemiology of animal bite reported at animal bite clinic during 2015-16 at a tertiary care centre of Himachal Pradesh	2018	354-360	Indian Journal of Community Health	30	4	data--human--pathogen--epidemiology-of-animal-bite-reported-at-animal-bite-clinic-during-2015-16-at-a-tertiary-care-centre-of-himachal-pradesh			
Journal Article	What does the fox say? Monitoring antimicrobial resistance in the environment using wild red foxes as an indicator	2018	e0198019	PLoS One	13	5	amr--data--domestic--wild--what-does-the-fox-say?-monitoring-antimicrobial-resistance-in-the-environment-using-wild-red-foxes-as-an-indicator	26/05/2018		
Journal Article	One health in our backyard: Design and evaluation of an experiential learning experience for veterinary medical students	2018	57-64	One Health	5		edu--eval--one-health-in-our-backyard:-design-and-evaluation-of-an-experiential-learning-experience-for-veterinary-medical-students			
Journal Article	Farming, Q fever and public health: agricultural practices and beyond	2018	9	Archives of Public Health	76		domestic--opinion--pathogen--farming,-q-fever-and-public-health:-agricultural-practices-and-beyond			
Journal Article	What do we know about parasites of wildlife in high biodiversity areas with anthropogenic disturbance? The special case of Mexico	2018	155-161	Anim Health Res Rev	19	2	opinion--pathogen--what-do-we-know-about-parasites-of-wildlife-in-high-biodiversity-areas-with-anthropogenic-disturbance?-the-special-case-of-mexico	27/01/2019		
Journal Article	A research agenda to reinforce rabies control: A qualitative and quantitative prioritization	2018	e0006387	PLoS Negl Trop Dis	12	5	eval--human--pathogen--risk--a-research-agenda-to-reinforce-rabies-control:-a-qualitative-and-quantitative-prioritization	05/05/2018		
Journal Article	Preventing Human Salmonella Infections Resulting from Live Poultry Contact through Interventions at Retail Stores	2018	155-166	Journal of Agricultural Safety and Health	24	3	opinion--pathogen--preventing-human-salmonella-infections-resulting-from-live-poultry-contact-through-interventions-at-retail-stores			
Journal Article	Soil Chemistry and the One Health Initiative: Introduction to the Special Section	2018	1305-1309	J Environ Qual	47	6	env--opinion--soil-chemistry-and-the-one-health-initiative:-introduction-to-the-special-section	05/12/2018		
Journal Article	Recent Asian origin of chytrid fungi causing global amphibian declines	2018	621-627	Science	360	6389	data--pathogen--recent-asian-origin-of-chytrid-fungi-causing-global-amphibian-declines	12/05/2018		
Journal Article	Antimicrobial Resistance in Food Animals and the Environment in Nigeria: A Review	2018		Int J Environ Res Public Health	15	6	amr--data--antimicrobial-resistance-in-food-animals-and-the-environment-in-nigeria:-a-review	20/06/2018		
Journal Article	Spread of multidrug-resistant Escherichia coli harboring integron via swine farm waste water treatment plant	2018	36-42	Ecotoxicol Environ Saf	149		amr--data--env--spread-of-multidrug-resistant-escherichia-coli-harboring-integron-via-swine-farm-waste-water-treatment-plant	18/11/2017		
Journal Article	One Environmental Health: an emerging perspective in toxicology	2018		F1000Res	7		cancer--non-bio--opinion--wild--one-environmental-health:-an-emerging-perspective-in-toxicology	14/07/2018		
Journal Article	Review: Animal health and sustainable global livestock systems	2018	1699-1708	Animal	12	8	domestic--opinion--review:-animal-health-and-sustainable-global-livestock-systems	11/04/2018		
Journal Article	Review of Cryptosporidium and Giardia in the eastern part of Europe, 2016	2018		Euro Surveill	23	4	opinion--pathogen--review-of-cryptosporidium-and-giardia-in-the-eastern-part-of-europe,-2016	01/02/2018		
Journal Article	How ownership rights over microorganisms affect infectious disease control and innovation: A root-cause analysis of barriers to data sharing as experienced by key stakeholders	2018	e0195885	PLoS One	13	5	eval--pathogen--policy--how-ownership-rights-over-microorganisms-affect-infectious-disease-control-and-innovation:-a-root-cause-analysis-of-barriers-to-data-sharing-as-experienced-by-key-stakeholders	03/05/2018		
Journal Article	Emerging Biological Risks in a Global Context: An Introduction	2018	1-12	Adv Food Nutr Res	86		pathogen--emerging-biological-risks-in-a-global-context:-an-introduction	06/08/2018		
Journal Article	Public understanding of One Health messages: The role of temporal framing	2018	185-196	Public Understanding of Science	27	2	data--human--policy--public-understanding-of-one-health-messages:-the-role-of-temporal-framing			
Journal Article	Spatial and temporal variation in the community prevalence of antibiotic resistance in Bangladesh: an integrated surveillance study protocol	2018	8	BMJ Open	8	4	amr--human--risk--wild--spatial-and-temporal-variation-in-the-community-prevalence-of-antibiotic-resistance-in-bangladesh:-an-integrated-surveillance-study-protocol			
Journal Article	Human, animal and environmental contributors to antibiotic resistance in low-resource settings: integrating behavioural, epidemiological and One Health approaches	2018	9	Proceedings of the Royal Society B-Biological Sciences	285	1876	amr--opinion--human,-animal-and-environmental-contributors-to-antibiotic-resistance-in-low-resource-settings:-integrating-behavioural,-epidemiological-and-one-health-approaches			
Journal Article	A systems Approach to evaluate one health Initiatives	2018	18	Frontiers in Veterinary Science	5		eval--a-systems-approach-to-evaluate-one-health-initiatives			
Journal Article	First 'Global Flipped Classroom in One Health': From MOOCs to research on real world challenges	2018	37-39	One Health	5		edu--first-'global-flipped-classroom-in-one-health':-from-moocs-to-research-on-real-world-challenges	19/06/2018		
Journal Article	Antimicrobial resistance in aquaculture: Current knowledge and alternatives to tackle the problem	2018	135-143	International Journal of Antimicrobial Agents	52	2	amr--env--opinion--antimicrobial-resistance-in-aquaculture:-current-knowledge-and-alternatives-to-tackle-the-problem			
Journal Article	Preliminary insights into the occurrence of similar clones of extended-spectrum beta-lactamase-producing bacteria in humans, animals and the environment in Tanzania: A systematic review and meta-analysis between 2005 and 2016	2018	1-10	Zoonoses Public Health	65	1	amr--data--preliminary-insights-into-the-occurrence-of-similar-clones-of-extended-spectrum-beta-lactamase-producing-bacteria-in-humans,-animals-and-the-environment-in-tanzania:-a-systematic-review-and-meta-analysis-between-2005-and-2016	24/08/2017		
Journal Article	Fecal indicator bacteria and zoonotic pathogens in marine snow and California mussels (Mytilus californianus)	2018		FEMS Microbiol Ecol	94	11	env--opinion--pathogen--fecal-indicator-bacteria-and-zoonotic-pathogens-in-marine-snow-and-california-mussels-(mytilus-californianus)	22/09/2018		
Journal Article	Prevalence of Selected Zoonotic Diseases and Risk Factors at a Human-Wildlife-Livestock Interface in Mpumalanga Province, South Africa	2018	303-310	Vector Borne Zoonotic Dis	18	6	data--human--pathogen--prevalence-of-selected-zoonotic-diseases-and-risk-factors-at-a-human-wildlife-livestock-interface-in-mpumalanga-province,-south-africa	18/04/2018		
Journal Article	Diversity of Salmonella serotypes from humans, food, domestic animals and wildlife in New South Wales, Australia	2018	11	Bmc Infectious Diseases	18		data--domestic--human--pathogen--diversity-of-salmonella-serotypes-from-humans,-food,-domestic-animals-and-wildlife-in-new-south-wales,-australia			
Journal Article	The role of companion animals in the environmental circulation of tick-borne bacterial pathogens	2018	473-480	Annals of Agricultural and Environmental Medicine	25	3	domestic--opinion--pathogen--the-role-of-companion-animals-in-the-environmental-circulation-of-tick-borne-bacterial-pathogens			
Journal Article	Seroprevalence for Brucella spp. in Baltic ringed seals (Phoca hispida) and East Greenland harp (Pagophilus groenlandicus) and hooded (Cystophora cristata) seals	2018	14-18	Veterinary Immunology and Immunopathology	198		data--pathogen--wild--seroprevalence-for-brucella-spp.-in-baltic-ringed-seals-(phoca-hispida)-and-east-greenland-harp-(pagophilus-groenlandicus)-and-hooded-(cystophora-cristata)-seals			
Journal Article	Multidrug resistance among Escherichia coli and Klebsiella pneumoniae carried in the gut of out-patients from pastoralist communities of Kasese district, Uganda	2018	e0200093	PLoS One	13	7	amr--data--human--multidrug-resistance-among-escherichia-coli-and-klebsiella-pneumoniae-carried-in-the-gut-of-out-patients-from-pastoralist-communities-of-kasese-district,-uganda	18/07/2018		
Journal Article	Study protocol for One Health data collections, analyses and intervention of the Sino-Swedish integrated multisectoral partnership for antibiotic resistance containment (IMPACT)	2018	e017832	BMJ Open	8	1	policy--study-protocol-for-one-health-data-collections,-analyses-and-intervention-of-the-sino-swedish-integrated-multisectoral-partnership-for-antibiotic-resistance-containment-(impact)	24/01/2018		
Journal Article	Clinical one health: A novel healthcare solution for underserved communities	2018	34-36	One Health	6		eval--policy--clinical-one-health:-a-novel-healthcare-solution-for-underserved-communities			
Journal Article	The grand challenge of great ape health and conservation in the anthropocene	2018		Am J Primatol	80	1	opinion--the-grand-challenge-of-great-ape-health-and-conservation-in-the-anthropocene	03/11/2017		
Journal Article	One Health Relationships Between Human, Animal, and Environmental Microbiomes: A Mini-Review	2018	9	Frontiers in Public Health	6		opinion--one-health-relationships-between-human,-animal,-and-environmental-microbiomes:-a-mini-review			
Journal Article	Phenotype diffusion and one health: A proposed framework for investigating the plurality of obesity epidemics across many species	2018	279-290	Zoonoses and Public Health	65	3	opinion--phenotype-diffusion-and-one-health:-a-proposed-framework-for-investigating-the-plurality-of-obesity-epidemics-across-many-species			
Journal Article	Human infectious diseases and the changing climate in the Arctic	2018	703-713	Environment International	121		climate--data--pathogen--human-infectious-diseases-and-the-changing-climate-in-the-arctic			
Journal Article	Equine Methicillin-Resistant Sequence Type 398 Staphylococcus aureus (MRSA) Harbor Mobile Genetic Elements Promoting Host Adaptation	2018	2516	Front Microbiol	9		amr--data--domestic--equine-methicillin-resistant-sequence-type-398-staphylococcus-aureus-(mrsa)-harbor-mobile-genetic-elements-promoting-host-adaptation	09/11/2018		
Journal Article	Climate change and One Health	2018	9	Fems Microbiology Letters	365	11	climate--opinion--climate-change-and-one-health			
Journal Article	EcoHealth and the Determinants of Health: Perspectives of a Small Subset of Canadian Academics in the EcoHealth Community	2018	1688	International Journal of Environmental Research and Public Health	15	8	ecohealth-and-the-determinants-of-health:-perspectives-of-a-small-subset-of-canadian-academics-in-the-ecohealth-community		8	
Journal Article	Sea otters in a dirty ocean	2007	1648-1652	J Am Vet Med Assoc	231	11	opinion--wild--sea-otters-in-a-dirty-ocean	07/12/2007		
Journal Article	One medicine, one university: the DVM/MPH program at the University of Illinois	2008	194-198	J Vet Med Educ	35	2	edu--opinion--one-medicine,-one-university:-the-dvm/mph-program-at-the-university-of-illinois	30/08/2008		
Journal Article	Teaching "one medicine, one health"	2008	169-170	Am J Med	121	3	edu--opinion--teaching-"one-medicine,-one-health"	11/03/2008		
Journal Article	The combined master of public health program at Tufts University	2008	182-186	J Vet Med Educ	35	2	edu--opinion--the-combined-master-of-public-health-program-at-tufts-university	30/08/2008		
Journal Article	Human and veterinary medicine: the priority for public health synergies	2008	577-582	Vet Ital	44	4	opinion--human-and-veterinary-medicine:-the-priority-for-public-health-synergies	01/10/2008		
Journal Article	Demographic and health surveillance of mobile pastoralists in Chad: integration of biometric fingerprint identification into a geographical information system	2008	113-124	Geospatial Health	3	1	data--human--demographic-and-health-surveillance-of-mobile-pastoralists-in-chad:-integration-of-biometric-fingerprint-identification-into-a-geographical-information-system			
Journal Article	'One Toxicology', 'Ecosystem Health' and 'One Health'	2009	97-110	Veterinaria Italiana	45	1	opinion--'one-toxicology',-'ecosystem-health'-and-'one-health'			
Journal Article	Evolution of a transdisciplinary "One Medicine-One Health" approach to global health education at the University of California, Davis	2009	268-274	Prev Vet Med	92	4	opinion--evolution-of-a-transdisciplinary-"one-medicine-one-health"-approach-to-global-health-education-at-the-university-of-california,-davis	13/10/2009		
Journal Article	A Public-Policy Practicum to Address Current Issues in Human, Animal, and Ecosystem Health	2009	397-402	Journal of Veterinary Medical Education	36	4	edu--eval--a-public-policy-practicum-to-address-current-issues-in-human,-animal,-and-ecosystem-health			
Journal Article	Emerging infections: a tribute to the one medicine, one health concept	2009	407-428	Zoonoses Public Health	56	6-7	edu--opinion--pathogen--emerging-infections:-a-tribute-to-the-one-medicine,-one-health-concept	03/06/2009		
Journal Article	'One Health' - the Rosetta stone for 21st century health and health providers	2009	377-382	Veterinaria Italiana	45	3	opinion--'one-health'---the-rosetta-stone-for-21st-century-health-and-health-providers			
Journal Article	Integrating the issues of global and public health into the veterinary education curriculum: a European perspective	2009	745-751	Revue Scientifique Et Technique-Office International Des Epizooties	28	2	edu--opinion--integrating-the-issues-of-global-and-public-health-into-the-veterinary-education-curriculum:-a-european-perspective			
Journal Article	Zoonoses and vector-borne diseases in Croatia - a multidisciplinary approach	2009	55-66	Veterinaria Italiana	45	1	opinion--pathogen--zoonoses-and-vector-borne-diseases-in-croatia---a-multidisciplinary-approach			
Journal Article	One World - One Medicine - One Health: emerging veterinary challenges and opportunities	2009	481-486	Revue Scientifique Et Technique-Office International Des Epizooties	28	2	opinion--one-world---one-medicine---one-health:-emerging-veterinary-challenges-and-opportunities			
Journal Article	Human and animal sentinels for shared health risks	2009	23-34	Veterinaria Italiana	45	1	opinion--human-and-animal-sentinels-for-shared-health-risks			
Journal Article	Veterinary education in Africa: current and future perspectives	2009	105-114	Onderstepoort J Vet Res	76	1	edu--opinion--veterinary-education-in-africa:-current-and-future-perspectives	09/12/2009		
Journal Article	"One Health" - The potential of closer collaboration between human and animal health	2009	410-411	Berliner Und Munchener Tierarztliche Wochenschrift	122	11-12	opinion--"one-health"---the-potential-of-closer-collaboration-between-human-and-animal-health			
Journal Article	One world – one health	2009	259-260	Clinical Medicine	9	3	one-world-–-one-health		6	Royal College of Physicians
Journal Article	One Health approach to identify research needs in bovine and human babesioses: workshop report	2010	11	Parasites & Vectors	3		opinion--pathogen--one-health-approach-to-identify-research-needs-in-bovine-and-human-babesioses:-workshop-report			
Journal Article	Understanding Risk Perceptions to Enhance Communication about Human-Wildlife Interactions and the Impacts of Zoonotic Disease	2010	255-261	Ilar Journal	51	3	opinion--risk--wild--understanding-risk-perceptions-to-enhance-communication-about-human-wildlife-interactions-and-the-impacts-of-zoonotic-disease			
Journal Article	Animal Studies and One Health: IACUC Considerations	2010	288-290	Ilar Journal	51	3	opinion--pathogen--animal-studies-and-one-health:-iacuc-considerations			
Journal Article	Zoonotic Enterohemorrhagic Escherichia coli: A One Health Perspective	2010	221-232	Ilar Journal	51	3	opinion--pathogen--zoonotic-enterohemorrhagic-escherichia-coli:-a-one-health-perspective			
Journal Article	Implications of the One Health Paradigm for Clinical Microbiology	2010	51-56	Clin Microbiol Newsl	32	7	opinion--implications-of-the-one-health-paradigm-for-clinical-microbiology	01/04/2010		
Journal Article	Animals as sentinels: using comparative medicine to move beyond the laboratory	2010	262-267	Ilar j	51	3	opinion--animals-as-sentinels:-using-comparative-medicine-to-move-beyond-the-laboratory	07/12/2010		
Journal Article	Current situation of the most frequent zoonosis in Mexico	2010	430-436	Gaceta Medica De Mexico	146	6	opinion--current-situation-of-the-most-frequent-zoonosis-in-mexico			
Journal Article	One health and climate change: linking environmental and animal health to human health	2010	434-437	N C Med J	71	5	opinion--one-health-and-climate-change:-linking-environmental-and-animal-health-to-human-health	09/04/2011		
Journal Article	Rabies control initiative in Tamil Nadu, India: a test case for the 'One Health' approach	2011	231-239	International Health	3	4	data--domestic--pathogen--true-oh--rabies-control-initiative-in-tamil-nadu,-india:-a-test-case-for-the-'one-health'-approach			
Journal Article	Use of a systems approach and evidence-based One Health for zoonoses research	2011	62-65	J Evid Based Med	4	2	opinion--pathogen--use-of-a-systems-approach-and-evidence-based-one-health-for-zoonoses-research	01/05/2011		
Journal Article	Integrating a One Health approach in education to address global health and sustainability challenges	2011	239-245	Frontiers in Ecology and the Environment	9	4	edu--opinion--integrating-a-one-health-approach-in-education-to-address-global-health-and-sustainability-challenges			
Journal Article	Toxicology, environmental health, and the "One Health" concept	2011	329-332	J Med Toxicol	7	4	eval--human--non-bio--toxicology,-environmental-health,-and-the-"one-health"-concept	06/08/2011		
Journal Article	Towards a conceptual framework to support one-health research for policy on emerging zoonoses	2011	326-331	Lancet Infectious Diseases	11	4	domestic--opinion--pathogen--towards-a-conceptual-framework-to-support-one-health-research-for-policy-on-emerging-zoonoses			
Journal Article	Role of pet dogs and cats in the transmission of helminthic zoonoses in Europe, with a focus on echinococcosis and toxocarosis	2011	41-53	Veterinary Parasitology	182	1	domestic--edu--opinion--pathogen--role-of-pet-dogs-and-cats-in-the-transmission-of-helminthic-zoonoses-in-europe,-with-a-focus-on-echinococcosis-and-toxocarosis			
Journal Article	Issues and special features of animal health research	2011	10	Veterinary Research	42		opinion--issues-and-special-features-of-animal-health-research			
Journal Article	Brucellosis at the animal/ecosystem/human interface at the beginning of the 21st century	2011	118-131	Prev Vet Med	102	2	opinion--pathogen--brucellosis-at-the-animal/ecosystem/human-interface-at-the-beginning-of-the-21st-century	17/05/2011		
Journal Article	One Health in Switzerland: A visionary concept at a crossroads?	2011	8	Swiss Medical Weekly	141		opinion--policy--one-health-in-switzerland:-a-visionary-concept-at-a-crossroads?			
Journal Article	Paradigm shift: contribution of field epidemiology training in advancing the "One Health" approach to strengthen disease surveillance and outbreak investigations in Africa	2011	13	Pan Afr Med J	10 Supp 1	Suppl 1	edu--opinion--paradigm-shift:-contribution-of-field-epidemiology-training-in-advancing-the-"one-health"-approach-to-strengthen-disease-surveillance-and-outbreak-investigations-in-africa	01/03/2012		
Journal Article	The need for veterinary nursing in Nigeria	2011	133-137	Vet Ital	47	2	opinion--the-need-for-veterinary-nursing-in-nigeria	28/06/2011		
Journal Article	A perspective on lyssavirus emergence and perpetuation	2011	662-670	Curr Opin Virol	1	6	opinion--pathogen--wild--a-perspective-on-lyssavirus-emergence-and-perpetuation	24/03/2012		
Journal Article	One health: zoonoses in the exotic animal practice	2011	421-426, v	Vet Clin North Am Exot Anim Pract	14	3	opinion--pathogen--one-health:-zoonoses-in-the-exotic-animal-practice	30/08/2011		
Journal Article	Investigation of an Escherichia coli Environmental Benchmark for Waterborne Pathogens in Agricultural Watersheds in Canada	2012	21-30	Journal of Environmental Quality	41	1	data--domestic--human--pathogen--wild--investigation-of-an-escherichia-coli-environmental-benchmark-for-waterborne-pathogens-in-agricultural-watersheds-in-canada			
Journal Article	Good governance in 'One Health' approaches	2012	561-575	Revue Scientifique Et Technique-Office International Des Epizooties	31	2	domestic--opinion--pathogen--good-governance-in-'one-health'-approaches			
Journal Article	One Health and EcoHealth in Ontario: a qualitative study exploring how holistic and integrative approaches are shaping public health practice in Ontario	2012	358	BMC Public Health	12		data--policy--one-health-and-ecohealth-in-ontario:-a-qualitative-study-exploring-how-holistic-and-integrative-approaches-are-shaping-public-health-practice-in-ontario	18/05/2012		
Journal Article	Emerging zoonoses: the "one health approach"	2012	77-83	Saf Health Work	3	1	opinion--pathogen--emerging-zoonoses:-the-"one-health-approach"	07/09/2012		
Journal Article	Zoonotic diseases and human health: The human influenza example	2012	4	Onderstepoort Journal of Veterinary Research	79	2	opinion--pathogen--zoonotic-diseases-and-human-health:-the-human-influenza-example			
Journal Article	From 'two medicines' to 'One Health' and beyond	2012	5	Onderstepoort Journal of Veterinary Research	79	2	opinion--from-'two-medicines'-to-'one-health'-and-beyond			
Journal Article	A New Model for Companion-Animal Primary Health Care Education	2012	210-216	Journal of Veterinary Medical Education	39	3	domestic--edu--eval--a-new-model-for-companion-animal-primary-health-care-education			
Journal Article	Multi-criteria decision analysis as an innovative approach to managing zoonoses: results from a study on Lyme disease in Canada	2013	897	BMC Public Health	13		pathogen--risk--multi-criteria-decision-analysis-as-an-innovative-approach-to-managing-zoonoses:-results-from-a-study-on-lyme-disease-in-canada	02/10/2013		
Journal Article	Coronaviruses in bats from Mexico	2013	1028-1038	J Gen Virol	94	Pt 5	data--pathogen--wild--coronaviruses-in-bats-from-mexico	01/02/2013		
Journal Article	Tuberculosis infection in wildlife from the Ruaha ecosystem Tanzania: implications for wildlife, domestic animals, and human health	2013	1371-1381	Epidemiology and Infection	141	7	data--pathogen--wild--tuberculosis-infection-in-wildlife-from-the-ruaha-ecosystem-tanzania:-implications-for-wildlife,-domestic-animals,-and-human-health			
Journal Article	Operationalizing a One Health approach to global health challenges	2013	211-216	Comp Immunol Microbiol Infect Dis	36	3	edu--opinion--operationalizing-a-one-health-approach-to-global-health-challenges	29/05/2013		
Journal Article	Wildlife disease and risk perception	2013	841-849	J Wildl Dis	49	4	data--edu--pathogen--risk--wildlife-disease-and-risk-perception	08/02/2014		
Journal Article	Assessing global adoption of one health approaches	2013	228-233	Ecohealth	10	3	eval--policy--assessing-global-adoption-of-one-health-approaches	10/07/2013		
Journal Article	Combating the Triple Threat: The Need for a One Health Approach	2013		Microbiol Spectr	1	1	opinion--combating-the-triple-threat:-the-need-for-a-one-health-approach	01/10/2013		
Journal Article	Rift valley fever in humans and animals in Mayotte, an endemic situation?	2013	e74192	PLoS One	8	9	data--domestic--human--pathogen--risk--wild--rift-valley-fever-in-humans-and-animals-in-mayotte,-an-endemic-situation?	08/10/2013		
Journal Article	Future challenges for parasitology: vector control and one health in the Americas	2013	249-255	Vet Parasitol	195	3-4	opinion--pathogen--future-challenges-for-parasitology:-vector-control-and-one-health-in-the-americas	30/04/2013		
Journal Article	Ecological Approaches to Studying Zoonoses	2013		Microbiol Spectr	1	2	opinion--pathogen--ecological-approaches-to-studying-zoonoses	01/12/2013		
Journal Article	Predicting the public health benefit of vaccinating cattle against Escherichia coli O157	2013	16265-16270	Proceedings of the National Academy of Sciences of the United States of America	110	40	data--pathogen--risk--predicting-the-public-health-benefit-of-vaccinating-cattle-against-escherichia-coli-o157			
Journal Article	One Health in the shrinking world: Experiences with tuberculosis at the human-livestock-wildlife interface	2013	263-268	Comparative Immunology Microbiology and Infectious Diseases	36	3	opinion--pathogen--one-health-in-the-shrinking-world:-experiences-with-tuberculosis-at-the-human-livestock-wildlife-interface			
Journal Article	Transdisciplinary research for complex One Health issues: A scoping review of key concepts	2013	222-229	Preventive Veterinary Medicine	112	3-4	opinion--transdisciplinary-research-for-complex-one-health-issues:-a-scoping-review-of-key-concepts			
Journal Article	Curriculum Asset Mapping for One Health Education	2013	363-369	Journal of Veterinary Medical Education	40	4	edu--eval--curriculum-asset-mapping-for-one-health-education			
Journal Article	Veterinary and public health aspects of Toxocara spp	2013	398-403	Veterinary Parasitology	193	4	domestic--edu--opinion--veterinary-and-public-health-aspects-of-toxocara-spp			
Journal Article	A Canadian application of one health: integration of Salmonella data from various Canadian surveillance programs (2005-2010)	2013	747-756	Foodborne Pathog Dis	10	9	opinion--pathogen--a-canadian-application-of-one-health:-integration-of-salmonella-data-from-various-canadian-surveillance-programs-(2005-2010)	22/06/2013		
Journal Article	Research at the interface between human and veterinary health	2013	187-193	Prev Vet Med	111	3-4	opinion--research-at-the-interface-between-human-and-veterinary-health	25/06/2013		
Journal Article	Toward proof of concept of a one health approach to disease prediction and control	2013		Emerg Infect Dis	19	12	opinion--toward-proof-of-concept-of-a-one-health-approach-to-disease-prediction-and-control	04/12/2013		
Journal Article	Wildlife: the need to better understand the linkages	2013	101-125	Curr Top Microbiol Immunol	365		opinion--pathogen--wild--wildlife:-the-need-to-better-understand-the-linkages	03/11/2012		
Journal Article	Review of Institute of Medicine and National Research Council Recommendations for One Health Initiative	2013	1913-1917	Emerging Infectious Diseases	19	12	opinion--policy--review-of-institute-of-medicine-and-national-research-council-recommendations-for-one-health-initiative			
Journal Article	Parasitic Zoonoses: One Health Surveillance in Northern Saskatchewan	2013	7	Plos Neglected Tropical Diseases	7	3	domestic--pathogen--parasitic-zoonoses:-one-health-surveillance-in-northern-saskatchewan			
Journal Article	Perspective: One Health: A Compelling Convergence	2013	49-55	Academic Medicine	88	1	opinion--perspective:-one-health:-a-compelling-convergence			
Journal Article	Has the Time Come for Big Science in Wildlife Health?	2013	335-338	Ecohealth	10	4	opinion--pathogen--risk--wild--has-the-time-come-for-big-science-in-wildlife-health?			
Journal Article	Toxoplasma gondii, source to sea: higher contribution of domestic felids to terrestrial parasite loading despite lower infection prevalence	2013	277-289	Ecohealth	10	3	data--domestic--pathogen--wild--toxoplasma-gondii,-source-to-sea:-higher-contribution-of-domestic-felids-to-terrestrial-parasite-loading-despite-lower-infection-prevalence	21/09/2013		
Journal Article	Molecules to modeling: Toxoplasma gondii oocysts at the human-animal-environment interface	2013	217-231	Comparative Immunology Microbiology and Infectious Diseases	36	3	opinion--pathogen--molecules-to-modeling:-toxoplasma-gondii-oocysts-at-the-human-animal-environment-interface			
Journal Article	Veterinary Students' Attitudes on One Health: Implications for Curriculum Development at Veterinary Colleges	2013	58-62	Journal of Veterinary Medical Education	40	1	data--edu--veterinary-students'-attitudes-on-one-health:-implications-for-curriculum-development-at-veterinary-colleges			
Journal Article	The Future of One Health	2014	Oh-0018-2012	Microbiol Spectr	2	1	opinion--the-future-of-one-health	01/02/2014		
Journal Article	Review: the important bacterial zoonoses in "one health" concept	2014	144	Front Public Health	2		opinion--pathogen--review:-the-important-bacterial-zoonoses-in-"one-health"-concept	30/10/2014		
Journal Article	Public Health Microbiology of Shiga Toxin-Producing Escherichia coli	2014		Microbiol Spectr	2	6	opinion--pathogen--public-health-microbiology-of-shiga-toxin-producing-escherichia-coli	25/06/2015		
Journal Article	Ecology and conservation: contributions to One Health	2014	615-627	Rev Sci Tech	33	2	env--opinion--wild--ecology-and-conservation:-contributions-to-one-health	25/02/2015		
Journal Article	World Organisation for Animal Health: strengthening Veterinary Services for effective One Health collaboration	2014	639-650	Rev Sci Tech	33	2	opinion--policy--world-organisation-for-animal-health:-strengthening-veterinary-services-for-effective-one-health-collaboration	25/02/2015		
Journal Article	Pathogenic Landscape of Transboundary Zoonotic Diseases in the Mexico-US Border Along the Rio Grande	2014	177	Front Public Health	2		opinion--pathogen--pathogenic-landscape-of-transboundary-zoonotic-diseases-in-the-mexico-us-border-along-the-rio-grande	03/12/2014		
Journal Article	A history of One Health	2014	413-420	Rev Sci Tech	33	2	opinion--a-history-of-one-health	25/02/2015		
Journal Article	Current status of rabies and prospects for elimination	2014	1389-1399	Lancet	384	9951	domestic--opinion--pathogen--current-status-of-rabies-and-prospects-for-elimination			
Journal Article	The Global One Health Paradigm: Challenges and Opportunities for Tackling Infectious Diseases at the Human, Animal, and Environment Interface in Low-Resource Settings	2014	6	Plos Neglected Tropical Diseases	8	11	pathogen--policy--the-global-one-health-paradigm:-challenges-and-opportunities-for-tackling-infectious-diseases-at-the-human,-animal,-and-environment-interface-in-low-resource-settings			
Journal Article	Public Health Perspectives on Aquaculture	2014	227-238	Curr Environ Health Rep	1	3	opinion--public-health-perspectives-on-aquaculture	26/08/2014		
Journal Article	The business case for One Health	2014	E1-6	Onderstepoort J Vet Res	81	2	data--policy--the-business-case-for-one-health	10/07/2014		

Journal Article	One health security: an important component of the global health security agenda	2014	221-224	Biosecur Bioterror	12	5	opinion--one-health-security:-an-important-component-of-the-global-health-security-agenda	26/09/2014
Journal Article	A One Health Framework for the Evaluation of Rabies Control Programmes: A Case Study from Colombo City, Sri Lanka	2014	19	Plos Neglected Tropical Diseases	8	10	eval--pathogen--risk--a-one-health-framework-for-the-evaluation-of-rabies-control-programmes:-a-case-study-from-colombo-city,-sri-lanka	
Journal Article	A need for One Health approach - lessons learned from outbreaks of Rift Valley fever in Saudi Arabia and Sudan	2014		Infect Ecol Epidemiol	4		eval--opinion--pathogen--a-need-for-one-health-approach---lessons-learned-from-outbreaks-of-rift-valley-fever-in-saudi-arabia-and-sudan	08/02/2014
Journal Article	An anthropological approach to teaching health sciences students cultural competency in a field school program	2014	251-256	Acad Med	89	2	edu--eval--an-anthropological-approach-to-teaching-health-sciences-students-cultural-competency-in-a-field-school-program	24/12/2013
Journal Article	GIARDIA IN MOUNTAIN GORILLAS (GORILLA BERINGEI BERINGEI), FOREST BUFFALO (SYNCERUS CAFFER), AND DOMESTIC CATTLE IN VOLCANOES NATIONAL PARK, RWANDA	2014	21-30	Journal of Wildlife Diseases	50	1	data--domestic--pathogen--wild--giardia-in-mountain-gorillas-(gorilla-beringei-beringei),-forest-buffalo-(syncerus-caffer),-and-domestic-cattle-in-volcanoes-national-park,-rwanda	
Journal Article	Preventing and controlling zoonotic tuberculosis: a One Health approach	2014	7-22	Veterinaria Italiana	50	1	opinion--pathogen--preventing-and-controlling-zoonotic-tuberculosis:-a-one-health-approach	
Journal Article	Species diversity of non-tuberculous mycobacteria isolated from humans, livestock and wildlife in the Serengeti ecosystem, Tanzania	2014	616	BMC Infect Dis	14		data--human--pathogen--species-diversity-of-non-tuberculous-mycobacteria-isolated-from-humans,-livestock-and-wildlife-in-the-serengeti-ecosystem,-tanzania	19/11/2014
Journal Article	Emerging and Reemerging Neglected Tropical Diseases: a Review of Key Characteristics, Risk Factors, and the Policy and Innovation Environment	2014	949-979	Clinical Microbiology Reviews	27	4	opinion--pathogen--emerging-and-reemerging-neglected-tropical-diseases:-a-review-of-key-characteristics,-risk-factors,-and-the-policy-and-innovation-environment	
Journal Article	Wildlife disease. Recent introduction of a chytrid fungus endangers Western Palearctic salamanders	2014	630-631	Science	346	6209	data--pathogen--wild--wildlife-disease.-recent-introduction-of-a-chytrid-fungus-endangers-western-palearctic-salamanders	02/11/2014
Journal Article	A Quantitative Prioritisation of Human and Domestic Animal Pathogens in Europe	2014	9	PLoS One	9	8	eval--pathogen--risk--a-quantitative-prioritisation-of-human-and-domestic-animal-pathogens-in-europe	
Journal Article	Hendra virus vaccine, a one health approach to protecting horse, human, and environmental health	2014	372-379	Emerg Infect Dis	20	3	opinion--pathogen--wild--hendra-virus-vaccine,-a-one-health-approach-to-protecting-horse,-human,-and-environmental-health	28/02/2014
Journal Article	The United Nations and One Health: the International Health Regulations (2005) and global health security	2014	659-668	Rev Sci Tech	33	2	opinion--policy--the-united-nations-and-one-health:-the-international-health-regulations-(2005)-and-global-health-security	25/02/2015
Journal Article	One Health: Past Successes and Future Challenges in Three African Contexts	2014	7	Plos Neglected Tropical Diseases	8	5	opinion--pathogen--policy--one-health:-past-successes-and-future-challenges-in-three-african-contexts	
Journal Article	The geo-spatial information infrastructure at the Centre for Control and Prevention of Zoonoses, University of Ibadan, Nigeria: an emerging sustainable One-Health pavilion	2014	65-78	Afr J Med Med Sci	43 Suppl		edu--eval--pathogen--the-geo-spatial-information-infrastructure-at-the-centre-for-control-and-prevention-of-zoonoses,-university-of-ibadan,-nigeria:-an-emerging-sustainable-one-health-pavilion	01/12/2014
Journal Article	Animal Leptospirosis in Latin America and the Caribbean Countries: Reported Outbreaks and Literature Review (2002-2014)	2014	10770-10789	International Journal of Environmental Research and Public Health	11	10	data--pathogen--animal-leptospirosis-in-latin-america-and-the-caribbean-countries:-reported-outbreaks-and-literature-review-(2002-2014)	
Journal Article	One Bioregion/One Health: An Integrative Narrative for Transboundary Planning along the US-Mexico Border	2014	419-440	Glob Soc	28	4	opinion--one-bioregion/one-health:-an-integrative-narrative-for-transboundary-planning-along-the-us-mexico-border	01/01/2014
Journal Article	Reservoir targeted vaccine against Borrelia burgdorferi: a new strategy to prevent Lyme disease transmission	2014	1972-1980	J Infect Dis	209	12	data--pathogen--wild--reservoir-targeted-vaccine-against-borrelia-burgdorferi:-a-new-strategy-to-prevent-lyme-disease-transmission	14/02/2014
Journal Article	Keeping parasitology under the One Health umbrella	2014	369-372	Trends in Parasitology	30	8	opinion--pathogen--keeping-parasitology-under-the-one-health-umbrella	
Journal Article	One medicine - one health - one biology and many proteins: proteomics on the verge of the One Health approach	2014	1226-1227	Molecular Biosystems	10	6	opinion--one-medicine---one-health---one-biology-and-many-proteins:-proteomics-on-the-verge-of-the-one-health-approach	
Journal Article	Spatial predictors of bovine tuberculosis infection and Brucella spp. exposure in pastoralist and agropastoralist livestock herds in the Ruaha ecosystem of Tanzania	2014	837-843	Tropical Animal Health and Production	46	5	data--pathogen--risk--spatial-predictors-of-bovine-tuberculosis-infection-and-brucella-spp.-exposure-in-pastoralist-and-agropastoralist-livestock-herds-in-the-ruaha-ecosystem-of-tanzania	
Journal Article	Making One Health a Reality--Crossing Bureaucratic Boundaries	2014	Oh-0016-2012	Microbiol Spectr	2	1	opinion--policy--making-one-health-a-reality--crossing-bureaucratic-boundaries	01/02/2014
Journal Article	Global distribution of two fungal pathogens threatening endangered sea turtles	2014	e85853	PLoS One	9	1	opinion--pathogen--wild--global-distribution-of-two-fungal-pathogens-threatening-endangered-sea-turtles	28/01/2014
Journal Article	Native rodent species are unlikely sources of infection for Leishmania (Viannia) braziliensis along the Transoceanic Highway in Madre de Dios, Peru	2014	e103358	PLoS One	9	7	data--pathogen--wild--native-rodent-species-are-unlikely-sources-of-infection-for-leishmania-(viannia)-braziliensis-along-the-transoceanic-highway-in-madre-de-dios,-peru	26/07/2014
Journal Article	One Health and Food-Borne Disease: Salmonella Transmission between Humans, Animals, and Plants	2014	Oh-0020-2013	Microbiol Spectr	2	1	opinion--pathogen--one-health-and-food-borne-disease:-salmonella-transmission-between-humans,-animals,-and-plants	01/02/2014
Journal Article	Avian influenza from an ecohealth perspective	2014	4-14	Ecohealth	11	1	opinion--pathogen--avian-influenza-from-an-ecohealth-perspective	26/04/2014
Journal Article	TOWARD A MODERNIZED DEFINITION OF WILDLIFE HEALTH	2014	427-430	Journal of Wildlife Diseases	50	3	env--opinion--wild--toward-a-modernized-definition-of-wildlife-health	
Journal Article	Is One Health delivering results? Introduction	2014	375-379	Revue Scientifique Et Technique-Office International Des Epizooties	33	2	eval--policy--is-one-health-delivering-results?-introduction	
Journal Article	One Medicine One Science: a framework for exploring challenges at the intersection of animals, humans, and the environment	2014	26-44	Ann N Y Acad Sci	1334	1	opinion--pathogen--one-medicine-one-science:-a-framework-for-exploring-challenges-at-the-intersection-of-animals,-humans,-and-the-environment	06/12/2014
Journal Article	Current initiatives in One Health: consolidating the One Health Global Network	2014	421-432	Revue Scientifique Et Technique-Office International Des Epizooties	33	2	opinion--pathogen--policy--current-initiatives-in-one-health:-consolidating-the-one-health-global-network	
Journal Article	The benefits of incorporating the One Health concept into the organisation of Veterinary Services	2014	393-399, 401-406	Rev Sci Tech	33	2	policy--the-benefits-of-incorporating-the-one-health-concept-into-the-organisation-of-veterinary-services	25/02/2015
Journal Article	Emerging zoonotic viral diseases	2014	569-581	Revue Scientifique Et Technique-Office International Des Epizooties	33	2	opinion--pathogen--emerging-zoonotic-viral-diseases	
Journal Article	Epidemiology of Leptospirosis in Africa: A Systematic Review of a Neglected Zoonosis and a Paradigm for 'One Health' in Africa	2015	e0003899	PLoS Negl Trop Dis	9	9	data--domestic--human--pathogen--epidemiology-of-leptospirosis-in-africa:-a-systematic-review-of-a-neglected-zoonosis-and-a-paradigm-for-'one-health'-in-africa	15/09/2015
Journal Article	Academic Institutions and One Health: Building Capacity for Transdisciplinary Research Approaches to Address Complex Health Issues at the Animal-Human-Ecosystem Interface	2015	866-871	Academic Medicine	90	7	opinion--academic-institutions-and-one-health:-building-capacity-for-transdisciplinary-research-approaches-to-address-complex-health-issues-at-the-animal-human-ecosystem-interface	
Journal Article	Need for Enhanced Environmental Representation in the Implementation of One Health	2015	212-219	Ecohealth	12	2	env--opinion--wild--need-for-enhanced-environmental-representation-in-the-implementation-of-one-health	
Journal Article	Non-random patterns in viral diversity	2015	8147	Nat Commun	6		pathogen--data--wild--non-random-patterns-in-viral-diversity	24/09/2015
Journal Article	A framework to promote collective action within the One Health community of practice: Using participatory modelling to enable interdisciplinary, cross-sectoral and multi-level integration	2015	44-48	One Health	1		opinion--policy--a-framework-to-promote-collective-action-within-the-one-health-community-of-practice:-using-participatory-modelling-to-enable-interdisciplinary,-cross-sectoral-and-multi-level-integration	
Journal Article	This could be the start of something big-20 years since the identification of bats as the natural host of Hendra virus	2015	14-16	One Health	1		opinion--pathogen--wild--this-could-be-the-start-of-something-big-20-years-since-the-identification-of-bats-as-the-natural-host-of-hendra-virus	04/08/2015
Journal Article	Seroprevalence of Antibodies against Seal Influenza A(H10N7) Virus in Harbor Seals and Gray Seals from the Netherlands	2015	e0144899	PLoS One	10	12	data--pathogen--wild--seroprevalence-of-antibodies-against-seal-influenza-a(h10n7)-virus-in-harbor-seals-and-gray-seals-from-the-netherlands	15/12/2015
Journal Article	Finding a Place for Systems-Based, Collaborative Research in Emerging Disease Research in Asia	2015	672-684	Ecohealth	12	4	opinion--policy--risk--finding-a-place-for-systems-based,-collaborative-research-in-emerging-disease-research-in-asia	20/11/2015
Journal Article	One Health and paradigms of public biobanking	2015	258-262	J Med Ethics	41	3	opinion--one-health-and-paradigms-of-public-biobanking	27/02/2014
Journal Article	Applying One Health to the Study of Animal-Assisted Interventions	2015	560-562	Ecohealth	12	4	opinion--applying-one-health-to-the-study-of-animal-assisted-interventions	13/06/2015
Journal Article	From "One Health" to "One Communication": The Contribution of Communication in Veterinary Medicine to Public Health	2015	135-149	Vet Sci	2	3	edu--opinion--from-"one-health"-to-"one-communication":-the-contribution-of-communication-in-veterinary-medicine-to-public-health	15/07/2015
Journal Article	Recognizing the Role of Skunks in Human and Animal Rabies Exposures in the Southwest	2015	494-501	Vector Borne Zoonotic Dis	15	8	opinion--pathogen--wild--recognizing-the-role-of-skunks-in-human-and-animal-rabies-exposures-in-the-southwest	15/08/2015
Journal Article	A One Health, participatory epidemiology assessment of anthrax (Bacillus anthracis) management in Western Uganda	2015	44-50	Social Science & Medicine	129		data--pathogen--wild--a-one-health,-participatory-epidemiology-assessment-of-anthrax-(bacillus-anthracis)-management-in-western-uganda	
Journal Article	One Health: An opportunity for an interprofessional approach to healthcare	2015	641-642	Journal of Interprofessional Care	29	6	opinion--policy--one-health:-an-opportunity-for-an-interprofessional-approach-to-healthcare	
Journal Article	Implementing a One Health approach to emerging infectious disease: reflections on the socio-political, ethical and legal dimensions	2015	1307	BMC Public Health	15		opinion--pathogen--implementing-a-one-health-approach-to-emerging-infectious-disease:-reflections-on-the-socio-political,-ethical-and-legal-dimensions	31/12/2015
Journal Article	Climate Change in the North American Arctic: A One Health Perspective	2015	713-725	Ecohealth	12	4	climate--non-bio--opinion--wild--climate-change-in-the-north-american-arctic:-a-one-health-perspective	14/06/2015
Journal Article	The One Health Perspective in Trace Elements Biomonitoring	2015	344-370	J Toxicol Environ Health B Crit Rev	18	7-8	domestic--non-bio--opinion--the-one-health-perspective-in-trace-elements-biomonitoring	23/12/2015
Journal Article	How to reach the poor? Surveillance in low-income countries, lessons from experiences in Cambodia and Madagascar	2015	12-26	Preventive Veterinary Medicine	120	1	domestic--opinion--pathogen--how-to-reach-the-poor?-surveillance-in-low-income-countries,-lessons-from-experiences-in-cambodia-and-madagascar	
Journal Article	Stakeholder Narratives on Trypanosomiasis, Their Effect on Policy and the Scope for One Health	2015	e0004241	PLoS Negl Trop Dis	9	12	domestic--human--pathogen--policy--risk--stakeholder-narratives-on-trypanosomiasis,-their-effect-on-policy-and-the-scope-for-one-health	15/12/2015
Journal Article	Concentration and retention of Toxoplasma gondii oocysts by marine snails demonstrate a novel mechanism for transmission of terrestrial zoonotic pathogens in coastal ecosystems	2015	4527-4537	Environ Microbiol	17	11	data--domestic--pathogen--wild--concentration-and-retention-of-toxoplasma-gondii-oocysts-by-marine-snails-demonstrate-a-novel-mechanism-for-transmission-of-terrestrial-zoonotic-pathogens-in-coastal-ecosystems	03/06/2015
Journal Article	Flying-foxes in the Australian urban environment-community attitudes and opinions	2015	24-30	One Health	1		data--edu--pathogen--wild--flying-foxes-in-the-australian-urban-environment-community-attitudes-and-opinions	
Journal Article	Integrating interdisciplinary methodologies for One Health: goat farm re-implicated as the probable source of an urban Q fever outbreak, the Netherlands, 2009	2015	372	BMC Infect Dis	15		data--domestic--pathogen--risk--integrating-interdisciplinary-methodologies-for-one-health:-goat-farm-re-implicated-as-the-probable-source-of-an-urban-q-fever-outbreak,-the-netherlands,-2009	04/09/2015
Journal Article	Recommendations for the role of social science research in One Health	2015	51-60	Soc Sci Med	129		opinion--recommendations-for-the-role-of-social-science-research-in-one-health	15/10/2014
Journal Article	Targeting Transmission Pathways for Emerging Zoonotic Disease Surveillance and Control	2015	432-437	Vector Borne Zoonotic Dis	15	7	pathogen--risk--targeting-transmission-pathways-for-emerging-zoonotic-disease-surveillance-and-control	18/07/2015
Journal Article	Our time is now - how companion animal veterinarians can transform biomedical science	2015	689-692	J Small Anim Pract	56	12	opinion--our-time-is-now---how-companion-animal-veterinarians-can-transform-biomedical-science	07/01/2016
Journal Article	'One Health' investigation: outbreak of human Salmonella Braenderup infections traced to a mail-order hatchery - United States, 2012-2013	2015	2178-2186	Epidemiology and Infection	143	10	data--domestic--pathogen--'one-health'-investigation:-outbreak-of-human-salmonella-braenderup-infections-traced-to-a-mail-order-hatchery---united-states,-2012-2013	
Journal Article	Ecohealth research in Southeast Asia: past, present and the way forward	2015	5	Infect Dis Poverty	4		policy--ecohealth-research-in-southeast-asia:-past,-present-and-the-way-forward	15/05/2015
Journal Article	Sentinel California sea lions provide insight into legacy organochlorine exposure trends and their association with cancer and infectious disease	2015	37-43	One Health	1		data--non-bio--wild--sentinel-california-sea-lions-provide-insight-into-legacy-organochlorine-exposure-trends-and-their-association-with-cancer-and-infectious-disease	14/09/2015
Journal Article	Atlantic Bottlenose Dolphins (Tursiops truncatus) as A Sentinel for Exposure to Mercury in Humans: Closing the Loop	2015	407-422	Vet Sci	2	4	non-bio--opinion--wild--atlantic-bottlenose-dolphins-(tursiops-truncatus)-as-a-sentinel-for-exposure-to-mercury-in-humans:-closing-the-loop	12/11/2015
Journal Article	Policies on pets for healthy cities: a conceptual framework	2015	976-986	Health Promot Int	30	4	domestic--human--policy--policies-on-pets-for-healthy-cities:-a-conceptual-framework	04/04/2014
Journal Article	Public health ethics and more-than-human solidarity	2015	61-67	Social Science & Medicine	129		human--opinion--public-health-ethics-and-more-than-human-solidarity	
Journal Article	One Health - a strategy for resilience in a changing arctic	2015	10	International Journal of Circumpolar Health	74		climate--env--opinion--one-health---a-strategy-for-resilience-in-a-changing-arctic	
Journal Article	Putative connection between zoonotic multiresistant extended-spectrum beta-lactamase (ESBL)-producing Escherichia coli in dog feces from a veterinary campus and clinical isolates from dogs	2015	25334	Infect Ecol Epidemiol	5		amr--data--domestic--pathogen--putative-connection-between-zoonotic-multiresistant-extended-spectrum-beta-lactamase-(esbl)-producing-escherichia-coli-in-dog-feces-from-a-veterinary-campus-and-clinical-isolates-from-dogs	07/02/2015
Journal Article	Leptospirosis in Rio Grande do Sul, Brazil: An Ecosystem Approach in the Animal-Human Interface	2015	20	Plos Neglected Tropical Diseases	9	11	data--human--pathogen--leptospirosis-in-rio-grande-do-sul,-brazil:-an-ecosystem-approach-in-the-animal-human-interface	
Journal Article	Who Let the Dogs Out? Communicating First Nations Perspectives on a Canine Veterinary Intervention Through Digital Storytelling	2015	592-601	Ecohealth	12	4	data--domestic--who-let-the-dogs-out?-communicating-first-nations-perspectives-on-a-canine-veterinary-intervention-through-digital-storytelling	
Journal Article	Stabilizing Dog Populations and Improving Animal and Public Health Through a Participatory Approach in Indigenous Communities	2015	445-455	Zoonoses and Public Health	62	6	data--domestic--stabilizing-dog-populations-and-improving-animal-and-public-health-through-a-participatory-approach-in-indigenous-communities	
Journal Article	Surveillance for Toxoplasma gondii in California mussels (Mytilus californianus) reveals transmission of atypical genotypes from land to sea	2015	4177-4188	Environ Microbiol	17	11	data--env--pathogen--wild--surveillance-for-toxoplasma-gondii-in-california-mussels-(mytilus-californianus)-reveals-transmission-of-atypical-genotypes-from-land-to-sea	05/11/2014
Journal Article	Toward integrated opisthorchiasis control in northeast Thailand: the Lawa project	2015	361-367	Acta Trop	141	Pt B	eval--human--pathogen--wild--toward-integrated-opisthorchiasis-control-in-northeast-thailand:-the-lawa-project	08/08/2014
Journal Article	One Health surveillance - More than a buzz word?	2015	124-130	Preventive Veterinary Medicine	120	1	edu--opinion--policy--one-health-surveillance---more-than-a-buzz-word?	
Journal Article	Disrupted seasonal biology impacts health, food security and ecosystems	2015	10	Proceedings of the Royal Society B-Biological Sciences	282	1817	climate--human--opinion--disrupted-seasonal-biology-impacts-health,-food-security-and-ecosystems	
Journal Article	Ebola Needs One Bioethics	2015	96-102	Ethics Policy & Environment	18	1	opinion--pathogen--ebola-needs-one-bioethics	
Journal Article	Organochlorine pesticides and PCBs in Southern Right Whales (Eubalaena australis) breeding at Península Valdés, Argentina	2015	605-615	Sci Total Environ	518-519		data--non-bio--wild--organochlorine-pesticides-and-pcbs-in-southern-right-whales-(eubalaena-australis)-breeding-at-península-valdés,-argentina	31/03/2015
Journal Article	Barriers to, Efforts in, and Optimization of Integrated One Health Surveillance: A Review and Synthesis	2015	368-384	Ecohealth	12	2	opinion--policy--barriers-to,-efforts-in,-and-optimization-of-integrated-one-health-surveillance:-a-review-and-synthesis	
Journal Article	Prevalence of Leptospira antibodies in wild boars (Sus scrofa) from Northern Portugal: risk factor analysis	2015	2126-2130	Epidemiol Infect	143	10	data--pathogen--wild--prevalence-of-leptospira-antibodies-in-wild-boars-(sus-scrofa)-from-northern-portugal:-risk-factor-analysis	19/12/2014
Journal Article	The dawn of Structural One Health: a new science tracking disease emergence along circuits of capital	2015	68-77	Soc Sci Med	129		opinion--the-dawn-of-structural-one-health:-a-new-science-tracking-disease-emergence-along-circuits-of-capital	15/10/2014
Journal Article	Epidemiological Characteristics of Human and Animal Rabies in Azerbaijan	2015	111-118	Zoonoses and Public Health	62	2	eval--pathogen--epidemiological-characteristics-of-human-and-animal-rabies-in-azerbaijan	
Journal Article	Trends in health surveillance and joint service delivery for pastoralists in West and Central Africa	2016	683-691	Revue Scientifique Et Technique-Office International Des Epizooties	35	2	domestic--opinion--pathogen--trends-in-health-surveillance-and-joint-service-delivery-for-pastoralists-in-west-and-central-africa	
Journal Article	Hepatitis E virus: Assessment of the epidemiological situation in humans in Europe, 2014/15	2016	9-16	J Clin Virol	82		eval--pathogen--hepatitis-e-virus:-assessment-of-the-epidemiological-situation-in-humans-in-europe,-2014/15	10/07/2016
Journal Article	One healthTransdisciplinary opportunities for SETAC leadership in integrating and improving the health of people, animals, and the environment	2016	2383-2391	Environmental Toxicology and Chemistry	35	10	non-bio--opinion--one-healthtransdisciplinary-opportunities-for-setac-leadership-in-integrating-and-improving-the-health-of-people,-animals,-and-the-environment	
Journal Article	Sentinel Animals in a One Health Approach to Harmful Cyanobacterial and Algal Blooms	2016		Vet Sci	3	2	non-bio--opinion--sentinel-animals-in-a-one-health-approach-to-harmful-cyanobacterial-and-algal-blooms	07/05/2016
Journal Article	Transdisciplinary Project Communication and Knowledge Sharing Experiences in Tanzania and Zambia through a One Health Lens	2016	10	Front Public Health	4		domestic--eval--transdisciplinary-project-communication-and-knowledge-sharing-experiences-in-tanzania-and-zambia-through-a-one-health-lens	24/02/2016
Journal Article	Spatiotemporal Analysis of the Genetic Diversity of Seal Influenza A(H10N7) Virus, Northwestern Europe	2016	4269-4277	J Virol	90	9	data--pathogen--wild--spatiotemporal-analysis-of-the-genetic-diversity-of-seal-influenza-a(h10n7)-virus,-northwestern-europe	29/01/2016
Journal Article	One Health approach to controlling a Q fever outbreak on an Australian goat farm	2016	1129-1141	Epidemiol Infect	144	6	data--domestic--eval--human--pathogen--one-health-approach-to-controlling-a-q-fever-outbreak-on-an-australian-goat-farm	24/10/2015
Journal Article	U.S. domestic cats as sentinels for perfluoroalkyl substances: Possible linkages with housing, obesity, and disease	2016	145-153	Environ Res	151		data--domestic--non-bio--u.s.-domestic-cats-as-sentinels-for-perfluoroalkyl-substances:-possible-linkages-with-housing,-obesity,-and-disease	21/10/2016
Journal Article	Aquaculture as yet another environmental gateway to the development and globalisation of antimicrobial resistance	2016	E127-E133	Lancet Infectious Diseases	16	7	amr--domestic--opinion--aquaculture-as-yet-another-environmental-gateway-to-the-development-and-globalisation-of-antimicrobial-resistance	
Journal Article	Integrating one health in national health policies of developing countries: India's lost opportunities	2016	87	Infect Dis Poverty	5	1	opinion--pathogen--policy--integrating-one-health-in-national-health-policies-of-developing-countries:-india's-lost-opportunities	08/10/2016
Journal Article	Towards better-informed consent: Research with livestock-keepers and informal traders in East Africa	2016	135-141	Prev Vet Med	128		eval--human--opinion--towards-better-informed-consent:-research-with-livestock-keepers-and-informal-traders-in-east-africa	31/05/2016
Journal Article	Culling and the Common Good: Re-evaluating Harms and Benefits under the One Health Paradigm	2016	244-254	Public Health Ethics	9	3	domestic--opinion--pathogen--wild--culling-and-the-common-good:-re-evaluating-harms-and-benefits-under-the-one-health-paradigm	30/10/2016
Journal Article	Position Paper: EUFEPS Network on Veterinary Medicines Initiative: An interdisciplinary forum to support Veterinary Pharmacology and promote the development of new pharmaceuticals for Animal Health	2016	I-VII	European Journal of Pharmaceutical Sciences	91		opinion--position-paper:-eufeps-network-on-veterinary-medicines-initiative:-an-interdisciplinary-forum-to-support-veterinary-pharmacology-and-promote-the-development-of-new-pharmaceuticals-for-animal-health	
Journal Article	Salmonella Typhimurium and Multidirectional Communication in the Gut	2016	18	Frontiers in Microbiology	7		opinion--pathogen--salmonella-typhimurium-and-multidirectional-communication-in-the-gut	
Journal Article	Wildlife Trade and Human Health in Lao PDR: An Assessment of the Zoonotic Disease Risk in Markets	2016	e0150666	PLoS One	11	3	data--pathogen--wild--wildlife-trade-and-human-health-in-lao-pdr:-an-assessment-of-the-zoonotic-disease-risk-in-markets	24/03/2016
Journal Article	Endemicity of chytridiomycosis features pathogen overdispersion	2016	806-816	J Anim Ecol	85	3	data--pathogen--wild--endemicity-of-chytridiomycosis-features-pathogen-overdispersion	06/02/2016
Journal Article	Using Domestic and Free-Ranging Arctic Canid Models for Environmental Molecular Toxicology Research	2016	1990-1999	Environ Sci Technol	50	4	climate--opinion--wild--using-domestic-and-free-ranging-arctic-canid-models-for-environmental-molecular-toxicology-research	06/01/2016
Journal Article	'WE CAN'T GET WORMS FROM COW DUNG': REPORTED KNOWLEDGE OF PARASITISM AMONG PASTORALIST YOUTH ATTENDING SECONDARY SCHOOL IN THE NGORONGORO CONSERVATION AREA, TANZANIA	2016	746-766	J Biosoc Sci	48	6	eval--human--pathogen--'we-can't-get-worms-from-cow-dung':-reported-knowledge-of-parasitism-among-pastoralist-youth-attending-secondary-school-in-the-ngorongoro-conservation-area,-tanzania	10/10/2015
Journal Article	Parasites, Plants, and People	2016	430-432	Trends in Parasitology	32	6	amr--opinion--pathogen--parasites,-plants,-and-people	
Journal Article	MOSQUITOES IN MOOSE COUNTRY: A MOSQUITO SURVEY OF NORTHERN MINNESOTA	2016	83-90	Journal of the American Mosquito Control Association	32	2	data--pathogen--wild--mosquitoes-in-moose-country:-a-mosquito-survey-of-northern-minnesota	
Journal Article	Genome Analysis of Clostridium difficile PCR Ribotype 014 Lineage in Australian Pigs and Humans Reveals a Diverse Genetic Repertoire and Signatures of Long-Range Interspecies Transmission	2016	2138	Front Microbiol	7		amr--data--domestic--human--pathogen--genome-analysis-of-clostridium-difficile-pcr-ribotype-014-lineage-in-australian-pigs-and-humans-reveals-a-diverse-genetic-repertoire-and-signatures-of-long-range-interspecies-transmission	27/01/2017
Journal Article	Prevalence of algal toxins in Alaskan marine mammals foraging in a changing arctic and subarctic environment	2016	13-24	Harmful Algae	55		data--non-bio--wild--prevalence-of-algal-toxins-in-alaskan-marine-mammals-foraging-in-a-changing-arctic-and-subarctic-environment	12/01/2017
Journal Article	A One Health Message about Bats Increases Intentions to Follow Public Health Guidance on Bat Rabies	2016	e0156205	PLoS One	11	5	eval--human--pathogen--wild--a-one-health-message-about-bats-increases-intentions-to-follow-public-health-guidance-on-bat-rabies	26/05/2016
Journal Article	Climatic suitability influences species specific abundance patterns of Australian flying foxes and risk of Hendra virus spillover	2016	115-121	One Health	2		pathogen--risk--wild--climatic-suitability-influences-species-specific-abundance-patterns-of-australian-flying-foxes-and-risk-of-hendra-virus-spillover	16/06/2017
Journal Article	Devastating Transboundary Impacts of Sea Star Wasting Disease on Subtidal Asteroids	2016	e0163190	PLoS One	11	10	data--wild--devastating-transboundary-impacts-of-sea-star-wasting-disease-on-subtidal-asteroids	27/10/2016
Journal Article	Dynamics, co-infections and characteristics of zoonotic tick-borne pathogens in Hokkaido small mammals, Japan	2016	922-928	Ticks and Tick-Borne Diseases	7	5	data--pathogen--wild--dynamics,-co-infections-and-characteristics-of-zoonotic-tick-borne-pathogens-in-hokkaido-small-mammals,-japan	
Journal Article	The role of environmental health in One Health: A Uganda perspective	2016	157-160	One Health	2		opinion--the-role-of-environmental-health-in-one-health:-a-uganda-perspective	16/06/2017
Journal Article	Interventions in live poultry markets for the control of avian influenza: a systematic review	2016	55-64	One Health	2		data--domestic--pathogen--risk--interventions-in-live-poultry-markets-for-the-control-of-avian-influenza:-a-systematic-review	24/05/2016
Journal Article	Molecular and epidemiological population-based integrative analysis of human and animal Mycobacterium bovis infections in a low-prevalence setting	2016	30-36	Veterinary Microbiology	195		data--domestic--human--pathogen--molecular-and-epidemiological-population-based-integrative-analysis-of-human-and-animal-mycobacterium-bovis-infections-in-a-low-prevalence-setting	
Journal Article	Prioritizing zoonotic diseases in Ethiopia using a one health approach	2016	131-135	One Health	2		domestic--human--pathogen--policy--wild--prioritizing-zoonotic-diseases-in-ethiopia-using-a-one-health-approach	22/02/2017
Journal Article	A One Health approach to antimicrobial resistance surveillance: is there a business case for it?	2016	422-427	International Journal of Antimicrobial Agents	48	4	opinion--a-one-health-approach-to-antimicrobial-resistance-surveillance:-is-there-a-business-case-for-it?	
Journal Article	Colistin in Pig Production: Chemistry, Mechanism of Antibacterial Action, Microbial Resistance Emergence, and One Health Perspectives	2016	22	Frontiers in Microbiology	7		amr--opinion--colistin-in-pig-production:-chemistry,-mechanism-of-antibacterial-action,-microbial-resistance-emergence,-and-one-health-perspectives	
Journal Article	One Health capacity building in sub-Saharan Africa	2016	34032	Infect Ecol Epidemiol	6		opinion--policy--one-health-capacity-building-in-sub-saharan-africa	03/12/2016
Journal Article	Clonal spread and interspecies transmission of clinically relevant ESBL-producing Escherichia coli of ST410--another successful pandemic clone?	2016		FEMS Microbiol Ecol	92	1	amr--data--domestic--wild--clonal-spread-and-interspecies-transmission-of-clinically-relevant-esbl-producing-escherichia-coli-of-st410--another-successful-pandemic-clone?	15/12/2015
Journal Article	High adult mortality in disease-challenged frog populations increases vulnerability to drought	2016	1453-1460	J Anim Ecol	85	6	pathogen--risk--wild--high-adult-mortality-in-disease-challenged-frog-populations-increases-vulnerability-to-drought	26/10/2016
Journal Article	Community-based surveillance of zoonotic parasites in a 'One Health' world: A systematic review	2016	166-174	One Health	2		data--pathogen--policy--community-based-surveillance-of-zoonotic-parasites-in-a-'one-health'-world:-a-systematic-review	
Journal Article	Toxoplasmosis and Toxocariasis: An Assessment of Human Immunodeficiency Virus Comorbidity and Health-Care Costs in Canada	2016	168-174	American Journal of Tropical Medicine and Hygiene	95	1	human--pathogen--risk--toxoplasmosis-and-toxocariasis:-an-assessment-of-human-immunodeficiency-virus-comorbidity-and-health-care-costs-in-canada	
Journal Article	40 years of veterinary papers in JAC - what have we learnt?	2016	2681-2690	J Antimicrob Chemother	71	10	amr--opinion--40-years-of-veterinary-papers-in-jac---what-have-we-learnt?	24/09/2016
Journal Article	Advancing the "One Health" workforce by integrating ecosystem health practice into veterinary medical education: The Envirovet Summer Institute	2016	170-183	Health Education Journal	75	2	edu--eval--human--advancing-the-"one-health"-workforce-by-integrating-ecosystem-health-practice-into-veterinary-medical-education:-the-envirovet-summer-institute	
Journal Article	One Health training and research activities in Western Europe	2016	33703	Infect Ecol Epidemiol	6		amr--opinion--one-health-training-and-research-activities-in-western-europe	03/12/2016
Journal Article	Habitat Management to Reduce Human Exposure to Trypanosoma cruzi and Western Conenose Bugs (Triatoma protracta)	2016	525-534	Ecohealth	13	3	data--pathogen--risk--wild--habitat-management-to-reduce-human-exposure-to-trypanosoma-cruzi-and-western-conenose-bugs-(triatoma-protracta)	16/08/2016
Journal Article	Dingoes (Canis dingo Meyer, 1793) continue to be an important reservoir host of Dirofilaria immitis in low density housing areas in Australia	2016	6-10	Vet Parasitol	215		data--domestic--pathogen--wild--dingoes-(canis-dingo-meyer,-1793)-continue-to-be-an-important-reservoir-host-of-dirofilaria-immitis-in-low-density-housing-areas-in-australia	23/01/2016
Journal Article	Leadership, governance and partnerships are essential One Health competencies	2016	161-163	One Health	2		opinion--leadership,-governance-and-partnerships-are-essential-one-health-competencies	
Journal Article	Canine brucellosis in Turkey from an environmental perspective	2016	131-136	Turkish Journal of Veterinary & Animal Sciences	40	2	domestic--opinion--pathogen--canine-brucellosis-in-turkey-from-an-environmental-perspective	
Journal Article	Human Q fever incidence is associated to spatiotemporal environmental conditions	2016	77-87	One Health	2		domestic--env--human--pathogen--risk--human-q-fever-incidence-is-associated-to-spatiotemporal-environmental-conditions	
Journal Article	Coastal development and precipitation drive pathogen flow from land to sea: evidence from a Toxoplasma gondii and felid host system	2016	29252	Sci Rep	6		env--pathogen--risk--coastal-development-and-precipitation-drive-pathogen-flow-from-land-to-sea:-evidence-from-a-toxoplasma-gondii-and-felid-host-system	28/07/2016
Journal Article	Access to human, animal, and environmental journals is still limited for the One Health community	2016	100-108	Journal of the Medical Library Association	104	2	data--domestic--env--human--wild--access-to-human,-animal,-and-environmental-journals-is-still-limited-for-the-one-health-community	
Journal Article	Biodiversity and health: Lessons and recommendations from an interdisciplinary conference to advise Southeast Asian research, society and policy	2016	29-46	Infection Genetics and Evolution	40		opinion--biodiversity-and-health:-lessons-and-recommendations-from-an-interdisciplinary-conference-to-advise-southeast-asian-research,-society-and-policy	
Journal Article	The Sero-epidemiology of Coxiella burnetii in Humans and Cattle, Western Kenya: Evidence from a Cross-Sectional Study	2016	e0005032	PLoS Negl Trop Dis	10	10	data--domestic--env--human--pathogen--risk--the-sero-epidemiology-of-coxiella-burnetii-in-humans-and-cattle,-western-kenya:-evidence-from-a-cross-sectional-study	08/10/2016
Journal Article	One health - an ecological and evolutionary framework for tackling Neglected Zoonotic Diseases	2016	313-333	Evolutionary Applications	9	2	opinion--pathogen--one-health---an-ecological-and-evolutionary-framework-for-tackling-neglected-zoonotic-diseases	
Journal Article	Global Solutions to Regional Challenges: Bridging the One Health Divide in the Caribbean	2016	8-10	One Health	2		policy--global-solutions-to-regional-challenges:-bridging-the-one-health-divide-in-the-caribbean	
Journal Article	One Health in China	2016	33843	Infect Ecol Epidemiol	6		opinion--one-health-in-china	03/12/2016
Journal Article	Emerging and reemerging zoonoses and their importance for animal health and production	2016	510-519	Pesquisa Agropecuaria Brasileira	51	5	opinion--emerging-and-reemerging-zoonoses-and-their-importance-for-animal-health-and-production	
Journal Article	The future of pastoralism: an introduction	2016	335-340	Revue Scientifique Et Technique-Office International Des Epizooties	35	2	domestic--human--opinion--the-future-of-pastoralism:-an-introduction	
Journal Article	Taking forward a 'One Health' approach for turning the tide against the Middle East respiratory syndrome coronavirus and other zoonotic pathogens with epidemic potential	2016	5-9	International Journal of Infectious Diseases	47		pathogen--policy--taking-forward-a-'one-health'-approach-for-turning-the-tide-against-the-middle-east-respiratory-syndrome-coronavirus-and-other-zoonotic-pathogens-with-epidemic-potential	
Journal Article	Changing Patterns of Emerging Zoonotic Diseases in Wildlife, Domestic Animals, and Humans Linked to Biodiversity Loss and Globalization	2017	315-318	Ilar Journal	58	3	climate--opinion--pathogen--changing-patterns-of-emerging-zoonotic-diseases-in-wildlife,-domestic-animals,-and-humans-linked-to-biodiversity-loss-and-globalization	
Journal Article	How should we respond to the emergence of plasmid-mediated colistin resistance in humans and animals?	2017	77-84	Int J Infect Dis	54		amr--domestic--opinion--how-should-we-respond-to-the-emergence-of-plasmid-mediated-colistin-resistance-in-humans-and-animals?	05/12/2016
Journal Article	Hyperferritinemia is a potential marker of chronic chikungunya: A retrospective study on the Island of Curaçao during the 2014-2015 outbreak	2017	31-38	J Clin Virol	86		data--human--pathogen--hyperferritinemia-is-a-potential-marker-of-chronic-chikungunya:-a-retrospective-study-on-the-island-of-curaçao-during-the-2014-2015-outbreak	03/12/2016
Journal Article	Engaging research with policy and action: what are the challenges of responding to zoonotic disease in Africa?	2017		Philos Trans R Soc Lond B Biol Sci	372	1725	eval--pathogen--policy--engaging-research-with-policy-and-action:-what-are-the-challenges-of-responding-to-zoonotic-disease-in-africa?	07/06/2017
Journal Article	A systematic review of zoonotic enteric parasitic diseases among nomadic and pastoral people	2017	22	PLoS One	12	11	pathogen--risk--a-systematic-review-of-zoonotic-enteric-parasitic-diseases-among-nomadic-and-pastoral-people	
Journal Article	One Health Solutions to Obesity in People and Their Pets	2017	326-333	Journal of Comparative Pathology	156	4	domestic--human--obesity--opinion--one-health-solutions-to-obesity-in-people-and-their-pets	
Journal Article	Evaluating one health: Are we demonstrating effectiveness?	2017	5-10	One Health	3		data--eval--evaluating-one-health:-are-we-demonstrating-effectiveness?	
Journal Article	Spatial and simultaneous representative seroprevalence of anti-Toxoplasma gondii antibodies in owners and their domiciled dogs in a major city of southern Brazil	2017	18	PLoS One	12	7	data--domestic--human--pathogen--spatial-and-simultaneous-representative-seroprevalence-of-anti-toxoplasma-gondii-antibodies-in-owners-and-their-domiciled-dogs-in-a-major-city-of-southern-brazil	
Journal Article	One Health: parasites and beyond	2017	1-6	Parasitology	144	1	opinion--pathogen--one-health:-parasites-and-beyond	31/08/2016
Journal Article	Framework to Define Structure and Boundaries of Complex Health Intervention Systems: The ALERT Project	2017	12	Frontiers in Public Health	5		domestic--eval--framework-to-define-structure-and-boundaries-of-complex-health-intervention-systems:-the-alert-project	
Journal Article	Wide exposure to Coxiella burnetii in ruminant and feline species living in a natural environment: zoonoses in a human-livestock-wildlife interface	2017	478-481	Epidemiol Infect	145	3	data--pathogen--wild--wide-exposure-to-coxiella-burnetii-in-ruminant-and-feline-species-living-in-a-natural-environment:-zoonoses-in-a-human-livestock-wildlife-interface	26/10/2016
Journal Article	Obesity and Associated Comorbidities in People and Companion Animals: A One Health Perspective	2017	296-309	J Comp Pathol	156	4	domestic--human--obesity--opinion--obesity-and-associated-comorbidities-in-people-and-companion-animals:-a-one-health-perspective	04/05/2017
Journal Article	Analysis of Food Safety and Security Challenges in Emerging African Food Producing Areas through a One Health Lens: The Dairy Chains in Mali	2017	57-67	J Food Prot	80	1	domestic--opinion--policy--analysis-of-food-safety-and-security-challenges-in-emerging-african-food-producing-areas-through-a-one-health-lens:-the-dairy-chains-in-mali	22/02/2017
Journal Article	Rabies elimination research: juxtaposing optimism, pragmatism and realism	2017		Proc Biol Sci	284	1869	opinion--pathogen--rabies-elimination-research:-juxtaposing-optimism,-pragmatism-and-realism	22/12/2017
Journal Article	One Health contributions towards more effective and equitable approaches to health in low- and middle-income countries	2017		Philos Trans R Soc Lond B Biol Sci	372	1725	opinion--pathogen--one-health-contributions-towards-more-effective-and-equitable-approaches-to-health-in-low--and-middle-income-countries	07/06/2017
Journal Article	One Health, emerging infectious diseases and wildlife: two decades of progress?	2017		Philos Trans R Soc Lond B Biol Sci	372	1725	opinion--one-health,-emerging-infectious-diseases-and-wildlife:-two-decades-of-progress?	07/06/2017
Journal Article	One Health for a changing world: new perspectives from Africa	2017		Philos Trans R Soc Lond B Biol Sci	372	1725	opinion--one-health-for-a-changing-world:-new-perspectives-from-africa	07/06/2017
Journal Article	Checklist for One Health Epidemiological Reporting of Evidence (COHERE)	2017	14-21	One Health	4		policy--checklist-for-one-health-epidemiological-reporting-of-evidence-(cohere)	22/08/2017
Journal Article	ISVEE 14 Yucatan 2015 14th Symposium of the International Society for Veterinary Epidemiology and Economics	2017	109-111	Prev Vet Med	137	Pt B	edu--opinion--policy--isvee-14-yucatan-2015-14th-symposium-of-the-international-society-for-veterinary-epidemiology-and-economics	30/09/2016
Journal Article	Molecular identification and characterization of piroplasm species in Hokkaido sika deer (Cervus nippon yesoensis), Japan	2017	802-807	Ticks and Tick-Borne Diseases	8	5	data--pathogen--wild--molecular-identification-and-characterization-of-piroplasm-species-in-hokkaido-sika-deer-(cervus-nippon-yesoensis),-japan	
Journal Article	Potential disease transmission from wild geese and swans to livestock, poultry and humans: a review of the scientific literature from a One Health perspective	2017	1300450	Infect Ecol Epidemiol	7	1	opinion--pathogen--wild--potential-disease-transmission-from-wild-geese-and-swans-to-livestock,-poultry-and-humans:-a-review-of-the-scientific-literature-from-a-one-health-perspective	02/06/2017
Journal Article	Multiple-host pathogens in domestic hunting dogs in Nicaragua's Bosawás Biosphere Reserve	2017	183-190	Acta Trop	167		data--domestic--pathogen--wild--multiple-host-pathogens-in-domestic-hunting-dogs-in-nicaragua's-bosawás-biosphere-reserve	26/12/2016
Journal Article	The Hotspot for (Global) One Health in Primary Food Production: Aflatoxin M1 in Dairy Products	2017	11	Frontiers in Public Health	5		domestic--non-bio--opinion--the-hotspot-for-(global)-one-health-in-primary-food-production:-aflatoxin-m1-in-dairy-products	
Journal Article	Detection of picobirnaviruses in vervet monkeys (Chlorocebus sabaeus): Molecular characterization of complete genomic segment-2	2017	13-18	Virus Research	230		data--domestic--pathogen--wild--detection-of-picobirnaviruses-in-vervet-monkeys-(chlorocebus-sabaeus):-molecular-characterization-of-complete-genomic-segment-2	
Journal Article	Review of Antibiotic Resistance in the Indian Ocean Commission: A Human and Animal Health Issue	2017	162	Front Public Health	5		amr--opinion--review-of-antibiotic-resistance-in-the-indian-ocean-commission:-a-human-and-animal-health-issue	22/07/2017
Journal Article	Should we be treating animal schistosomiasis in Africa? The need for a One Health economic evaluation of schistosomiasis control in people and their livestock	2017	244-247	Transactions of the Royal Society of Tropical Medicine and Hygiene	111	6	domestic--opinion--policy--should-we-be-treating-animal-schistosomiasis-in-africa?-the-need-for-a-one-health-economic-evaluation-of-schistosomiasis-control-in-people-and-their-livestock	
Journal Article	Poor livestock keepers: ecosystem-poverty-health interactions	2017		Philos Trans R Soc Lond B Biol Sci	372	1725	opinion--pathogen--poor-livestock-keepers:-ecosystem-poverty-health-interactions	07/06/2017
Journal Article	Rhipicephalus sanguineus importation into the UK: surveillance, risk, public health awareness and One Health response	2017	119-+	Veterinary Record	180	5	domestic--opinion--pathogen--rhipicephalus-sanguineus-importation-into-the-uk:-surveillance,-risk,-public-health-awareness-and-one-health-response	
Journal Article	Epidemiological Risk Factors for Animal Influenza A Viruses Overcoming Species Barriers	2017	342-360	Ecohealth	14	2	data--pathogen--risk--epidemiological-risk-factors-for-animal-influenza-a-viruses-overcoming-species-barriers	20/05/2017
Journal Article	IMPLICATIONS OF URBAN AND RURAL AGRICULTURAL PRACTICES ON THE TRANSMISSION OF LEPTOSPIROSIS	2017	725-741	Agrociencia	51	7	domestic--opinion--pathogen--implications-of-urban-and-rural-agricultural-practices-on-the-transmission-of-leptospirosis	
Journal Article	RETROSPECTIVE ANALYSIS OF THE EPIDEMIOLOGIC LITERATURE, 1990-2015, ON WILDLIFE-ASSOCIATED DISEASES FROM THE REPUBLIC OF KOREA	2017	5-18	Journal of Wildlife Diseases	53	1	domestic--opinion--pathogen--wild--retrospective-analysis-of-the-epidemiologic-literature,-1990-2015,-on-wildlife-associated-diseases-from-the-republic-of-korea	
Journal Article	Eco-social processes influencing infectious disease emergence and spread	2017	26-36	Parasitology	144	1	opinion--eco-social-processes-influencing-infectious-disease-emergence-and-spread	10/09/2016
Journal Article	Molecular Characterization of Salmonella from Human and Animal Origins in Uganda	2017	4604789	Int J Bacteriol	2017		data--domestic--human--pathogen--wild--molecular-characterization-of-salmonella-from-human-and-animal-origins-in-uganda	22/06/2017
Journal Article	The need for European OneHealth/EcoHealth networks	2017	8	Archives of Public Health	75		opinion--policy--the-need-for-european-onehealth/ecohealth-networks	
Journal Article	Quantitative Shedding of Multiple Genotypes of Cryptosporidium and Giardia by Deer Mice (Peromyscus maniculatus) in a Major Agricultural Region on the California Central Coast	2017	819-828	J Food Prot	80	5	data--domestic--pathogen--wild--quantitative-shedding-of-multiple-genotypes-of-cryptosporidium-and-giardia-by-deer-mice-(peromyscus-maniculatus)-in-a-major-agricultural-region-on-the-california-central-coast	08/04/2017
Journal Article	Local disease-ecosystem-livelihood dynamics: reflections from comparative case studies in Africa	2017		Philos Trans R Soc Lond B Biol Sci	372	1725	eval--opinion--pathogen--local-disease-ecosystem-livelihood-dynamics:-reflections-from-comparative-case-studies-in-africa	07/06/2017

Journal Article	Understanding Giardia infections among rural communities using the one health approach	2017	349-354	Acta Tropica	176		data--env--pathogen--understanding-giardia-infections-among-rural-communities-using-the-one-health-approach	
Journal Article	Pathological findings in the red fox (Vulpes vulpes), stone marten (Martes foina) and raccoon dog (Nyctereutes procyonoides), with special emphasis on infectious and zoonotic agents in Northern Germany	2017	20	PLoS One	12	4	data--pathogen--wild--pathological-findings-in-the-red-fox-(vulpes-vulpes),-stone-marten-(martes-foina)-and-raccoon-dog-(nyctereutes-procyonoides),-with-special-emphasis-on-infectious-and-zoonotic-agents-in-northern-germany	
Journal Article	A Comparison of Three Holistic Approaches to Health: One Health, EcoHealth, and Planetary Health	2017	163	Front Vet Sci	4		eval--opinion--a-comparison-of-three-holistic-approaches-to-health:-one-health,-ecohealth,-and-planetary-health	01/11/2017
Journal Article	An evaluation of the virulence and adherence properties of avian pathogenic Escherichia coli	2017	22-26	One Health	4		data--domestic--pathogen--an-evaluation-of-the-virulence-and-adherence-properties-of-avian-pathogenic-escherichia-coli	20/09/2017
Journal Article	Ecological determinants of health: food and environment on human health	2017	9002-9015	Environmental Science and Pollution Research	24	10	opinion--ecological-determinants-of-health:-food-and-environment-on-human-health	
Journal Article	Views from many worlds: unsettling categories in interdisciplinary research on endemic zoonotic diseases	2017	9	Philosophical Transactions of the Royal Society B-Biological Sciences	372	1725	domestic--human--pathogen--policy--views-from-many-worlds:-unsettling-categories-in-interdisciplinary-research-on-endemic-zoonotic-diseases	
Journal Article	Veterinary epidemiology: Forging a path toward one health	2017	147-150	Preventive Veterinary Medicine	137		eval--opinion--veterinary-epidemiology:-forging-a-path-toward-one-health	
Journal Article	Primates on display: Potential disease consequences beyond bushmeat	2017	32-43	American Journal of Physical Anthropology	162		human--opinion--pathogen--wild--primates-on-display:-potential-disease-consequences-beyond-bushmeat	
Journal Article	High prevalence of Seoul hantavirus in a breeding colony of pet rats	2017	3115-3124	Epidemiology and Infection	145	15	data--pathogen--wild--high-prevalence-of-seoul-hantavirus-in-a-breeding-colony-of-pet-rats	
Journal Article	The science behind One Health: at the interface of humans, animals, and the environment	2017	12-32	Annals of the New York Academy of Sciences	1395	1	edu--eval--policy--the-science-behind-one-health:-at-the-interface-of-humans,-animals,-and-the-environment	
Journal Article	Rethinking Human-Nonhuman Primate Contact and Pathogenic Disease Spillover	2017	840-850	Ecohealth	14	4	human--opinion--pathogen--wild--rethinking-human-nonhuman-primate-contact-and-pathogenic-disease-spillover	19/11/2017
Journal Article	Implementing One Health as an integrated approach to health in Rwanda	2017	e000121	BMJ Glob Health	2	1	opinion--policy--implementing-one-health-as-an-integrated-approach-to-health-in-rwanda	08/06/2017
Journal Article	Zinc oxide enriched peat influence Escherichia coli infection related diarrhea, growth rates, serum and tissue zinc levels in Norwegian piglets around weaning: five case herd trials	2017	14	Porcine Health Manag	3		data--domestic--non-bio--wild--zinc-oxide-enriched-peat-influence-escherichia-coli-infection-related-diarrhea,-growth-rates,-serum-and-tissue-zinc-levels-in-norwegian-piglets-around-weaning:-five-case-herd-trials	07/07/2017
Journal Article	Spatial Overlap Between People and Non-human Primates in a Fragmented Landscape	2017	88-99	Ecohealth	14	1	data--human--risk--wild--spatial-overlap-between-people-and-non-human-primates-in-a-fragmented-landscape	08/12/2016
Journal Article	Law as a fixture between the One Health interfaces of emerging diseases	2017	241-243	Trans R Soc Trop Med Hyg	111	6	opinion--pathogen--law-as-a-fixture-between-the-one-health-interfaces-of-emerging-diseases	19/10/2017
Journal Article	Developing a local antimicrobial resistance action plan: the Cornwall One Health Antimicrobial Resistance Group	2017	2661-2665	J Antimicrob Chemother	72	9	amr--eval--developing-a-local-antimicrobial-resistance-action-plan:-the-cornwall-one-health-antimicrobial-resistance-group	09/06/2017
Journal Article	Antibiotic Resistance in an Indian Rural Community: A 'One-Health' Observational Study on Commensal Coliform from Humans, Animals, and Water	2017	13	International Journal of Environmental Research and Public Health	14	4	amr--data--domestic--env--human--antibiotic-resistance-in-an-indian-rural-community:-a-'one-health'-observational-study-on-commensal-coliform-from-humans,-animals,-and-water	
Journal Article	Incorporating one health into medical education	2017	45	BMC Med Educ	17	1	edu--opinion--incorporating-one-health-into-medical-education	24/02/2017
Journal Article	Comparative aspects of canine and human inflammatory breast cancer	2017	288-300	Semin Oncol	44	4	cancer--opinion--comparative-aspects-of-canine-and-human-inflammatory-breast-cancer	13/03/2018
Journal Article	Spatial, seasonal and climatic predictive models of Rift Valley fever disease across Africa	2017		Philos Trans R Soc Lond B Biol Sci	372	1725	climate--human--pathogen--risk--spatial,-seasonal-and-climatic-predictive-models-of-rift-valley-fever-disease-across-africa	07/06/2017
Journal Article	A process for developing multisectoral strategies for zoonoses: the case of leptospirosis in Fiji	2017	671	BMC Public Health	17	1	eval--human--pathogen--a-process-for-developing-multisectoral-strategies-for-zoonoses:-the-case-of-leptospirosis-in-fiji	24/08/2017
Journal Article	AIDS, Avian flu, SARS, MERS, Ebola, Zika… what next?	2017	4470-4474	Vaccine	35	35 Pt A	opinion--pathogen--aids,-avian-flu,-sars,-mers,-ebola,-zika…-what-next?	22/06/2017
Journal Article	Who or what is 'the public' in critical public health? Reflections on posthumanism and anthropological engagements with One Health	2017	314-324	Critical Public Health	27	3	domestic--human--opinion--policy--who-or-what-is-'the-public'-in-critical-public-health?-reflections-on-posthumanism-and-anthropological-engagements-with-one-health	
Journal Article	Dog-bites, rabies and One Health: Towards improved coordination in research, policy and practice	2017	126-133	Soc Sci Med	187		eval--opinion--pathogen--dog-bites,-rabies-and-one-health:-towards-improved-coordination-in-research,-policy-and-practice	07/07/2017
Journal Article	A Blueprint to evaluate One Health	2017	5	Frontiers in Public Health	5		eval--a-blueprint-to-evaluate-one-health	
Journal Article	Using a One Health approach to assess the impact of parasitic disease in livestock: how does it add value?	2017	15-25	Parasitology	144	1	opinion--pathogen--policy--using-a-one-health-approach-to-assess-the-impact-of-parasitic-disease-in-livestock:-how-does-it-add-value?	05/04/2016
Journal Article	Diversity of Vibrio navarrensis Revealed by Genomic Comparison: Veterinary Isolates Are Related to Strains Associated with Human Illness and Sewage Isolates While Seawater Strains Are More Distant	2017	13	Frontiers in Microbiology	8		data--env--pathogen--diversity-of-vibrio-navarrensis-revealed-by-genomic-comparison:-veterinary-isolates-are-related-to-strains-associated-with-human-illness-and-sewage-isolates-while-seawater-strains-are-more-distant	
Journal Article	Online surveillance of media health event reporting in Nepal: digital disease detection from a One Health perspective	2017	7	Bmc International Health and Human Rights	17		data--eval--online-surveillance-of-media-health-event-reporting-in-nepal:-digital-disease-detection-from-a-one-health-perspective	
Journal Article	People, Patches, and Parasites: The Case of Trypanosomiasis in Zimbabwe	2017	643-654	Human Ecology	45	5	human--pathogen--risk--wild--people,-patches,-and-parasites:-the-case-of-trypanosomiasis-in-zimbabwe	
Journal Article	Integrative modelling for One Health: pattern, process and participation	2017		Philos Trans R Soc Lond B Biol Sci	372	1725	opinion--pathogen--integrative-modelling-for-one-health:-pattern,-process-and-participation	07/06/2017
Journal Article	Multidrug and Mupirocin Resistance in Environmental Methicillin-Resistant Staphylococcus aureus (MRSA) Isolates from Homes of People Diagnosed with Community-Onset MRSA Infection	2017	14	Applied and Environmental Microbiology	83	22	amr--data--domestic--human--pathogen--risk--multidrug-and-mupirocin-resistance-in-environmental-methicillin-resistant-staphylococcus-aureus-(mrsa)-isolates-from-homes-of-people-diagnosed-with-community-onset-mrsa-infection	
Journal Article	Optimization of human, animal, and environmental health by using the One Health approach	2017	263-268	Journal of Veterinary Science	18		human--opinion--policy--wild--optimization-of-human,-animal,-and-environmental-health-by-using-the-one-health-approach	
Journal Article	A veterinary perspective on One Health in the Arctic	2017	11	Acta Veterinaria Scandinavica	59		climate--non-bio--opinion--pathogen--wild--a-veterinary-perspective-on-one-health-in-the-arctic	
Journal Article	The Lawa model: A sustainable, integrated opisthorchiasis control program using the EcoHealth approach in the Lawa Lake region of Thailand	2017	346-354	Parasitol Int	66	4	eval--pathogen--wild--the-lawa-model:-a-sustainable,-integrated-opisthorchiasis-control-program-using-the-ecohealth-approach-in-the-lawa-lake-region-of-thailand	29/11/2016
Journal Article	BUILDing BLaST: promoting rural students' biomedical research careers using a culturally responsive, one health approach	2017	13	BMC Proc	11	Suppl 12	edu--building-blast:-promoting-rural-students'-biomedical-research-careers-using-a-culturally-responsive,-one-health-approach	30/01/2018
Journal Article	Rational use of antimicrobials in animal production: a prerequisite to stem the tide of antimicrobial resistance	2017	1846-1857	Current Science	113	10	amr--domestic--opinion--rational-use-of-antimicrobials-in-animal-production:-a-prerequisite-to-stem-the-tide-of-antimicrobial-resistance	
Journal Article	Hepatitis E Virus and Related Viruses in Animals	2017	37-52	Transboundary and Emerging Diseases	64	1	opinion--pathogen--hepatitis-e-virus-and-related-viruses-in-animals	
Journal Article	Multidrug resistant Enterobacteriaceae in New Zealand: a current perspective	2017	62-70	N Z Vet J	65	2	amr--domestic--human--opinion--multidrug-resistant-enterobacteriaceae-in-new-zealand:-a-current-perspective	09/12/2016
Journal Article	Agriculture and Agri-Food Canada's research program on antimicrobial resistance	2017	224-227	Can Commun Dis Rep	43	11	amr--domestic--risk--agriculture-and-agri-food-canada's-research-program-on-antimicrobial-resistance	18/05/2018
Journal Article	Microbiological diagnosis and antimicrobial sensitivity profiles in diseased free-living raptors	2017	442-450	Avian Pathology	46	4	data--pathogen--wild--microbiological-diagnosis-and-antimicrobial-sensitivity-profiles-in-diseased-free-living-raptors	
Journal Article	Comparing national infectious disease surveillance systems: China and the Netherlands	2017	415	BMC Public Health	17	1	eval--pathogen--risk--comparing-national-infectious-disease-surveillance-systems:-china-and-the-netherlands	10/05/2017
Journal Article	Precision wildlife medicine: applications of the human-centred precision medicine revolution to species conservation	2017	1792-1805	Global Change Biology	23	5	opinion--pathogen--wild--precision-wildlife-medicine:-applications-of-the-human-centred-precision-medicine-revolution-to-species-conservation	
Journal Article	Comparison of the Performance of Different Microbial Source Tracking Markers among European and North African Regions	2017	760-766	Journal of Environmental Quality	46	4	data--env--pathogen--comparison-of-the-performance-of-different-microbial-source-tracking-markers-among-european-and-north-african-regions	
Journal Article	Subtype analysis of zoonotic pathogen Cryptosporidium skunk genotype	2017	20-25	Infection Genetics and Evolution	55		data--env--pathogen--subtype-analysis-of-zoonotic-pathogen-cryptosporidium-skunk-genotype	
Journal Article	One health approach in Nepal: Scope, opportunities and challenges	2019	100101	One Health	8		eval--opinion--one-health-approach-in-nepal:-scope,-opportunities-and-challenges	06/09/2019
Journal Article	Transdisciplinary and social-ecological health frameworks-Novel approaches to emerging parasitic and vector-borne diseases	2019	14	Parasite Epidemiology and Control	4		env--opinion--pathogen--policy--transdisciplinary-and-social-ecological-health-frameworks-novel-approaches-to-emerging-parasitic-and-vector-borne-diseases	
Journal Article	From Four-Way Linking to a One Health Platform in Egypt: institutionalisation of a multidisciplinary and multisectoral One Health system	2019	261-270	Revue Scientifique Et Technique-Office International Des Epizooties	38	1	opinion--pathogen--policy--from-four-way-linking-to-a-one-health-platform-in-egypt:-institutionalisation-of-a-multidisciplinary-and-multisectoral-one-health-system	
Journal Article	The good, the bad and the ugly: framing debates on nature in a One Health community	2019	1729-1738	Sustain Sci	14	6	opinion--policy--the-good,-the-bad-and-the-ugly:-framing-debates-on-nature-in-a-one-health-community	01/01/2019
Journal Article	Systematic Review of Important Bacterial Zoonoses in Africa in the Last Decade in Light of the "One Health" Concept	2019	29	Pathogens	8	2	data--human--pathogen--policy--risk--systematic-review-of-important-bacterial-zoonoses-in-africa-in-the-last-decade-in-light-of-the-"one-health"-concept	
Journal Article	A One Health perspective to identify environmental factors that affect Rift Valley fever transmission in Gezira state, Central Sudan	2019	10	Tropical Medicine and Health	47	1	data--human--pathogen--risk--a-one-health-perspective-to-identify-environmental-factors-that-affect-rift-valley-fever-transmission-in-gezira-state,-central-sudan	
Journal Article	One Health Surveillance: perceived benefits and workforce motivations	2019	251-260	Revue Scientifique Et Technique-Office International Des Epizooties	38	1	eval--human--one-health-surveillance:-perceived-benefits-and-workforce-motivations	
Journal Article	Policy and Science for Global Health Security: Shaping the Course of International Health	2019		Trop Med Infect Dis	4	2	opinion--policy-and-science-for-global-health-security:-shaping-the-course-of-international-health	13/04/2019
Journal Article	Risk factors for bacterial zoonotic pathogens in acutely febrile patients in Mpumalanga Province, South Africa	2019	458-469	Zoonoses Public Health	66	5	domestic--human--opinion--pathogen--risk--risk-factors-for-bacterial-zoonotic-pathogens-in-acutely-febrile-patients-in-mpumalanga-province,-south-africa	13/03/2019
Journal Article	One Health Surveillance: A Matrix to Evaluate Multisectoral Collaboration	2019	109	Front Vet Sci	6		eval--opinion--policy--one-health-surveillance:-a-matrix-to-evaluate-multisectoral-collaboration	21/05/2019
Journal Article	Making Vector-Borne Disease Surveillance Work: New Opportunities From the SDG Perspectives	2019	232	Front Vet Sci	6		human--opinion--pathogen--policy--making-vector-borne-disease-surveillance-work:-new-opportunities-from-the-sdg-perspectives	06/08/2019
Journal Article	The Influence of Climate and Livestock Reservoirs on Human Cases of Giardiasis	2019	116-127	Ecohealth	16	1	domestic--env--human--pathogen--risk--the-influence-of-climate-and-livestock-reservoirs-on-human-cases-of-giardiasis	24/10/2018
Journal Article	Proficiency Testing of Virus Diagnostics Based on Bioinformatics Analysis of Simulated In Silico High-Throughput Sequencing Data Sets	2019		J Clin Microbiol	57	8	eval--proficiency-testing-of-virus-diagnostics-based-on-bioinformatics-analysis-of-simulated-in-silico-high-throughput-sequencing-data-sets	07/06/2019
Journal Article	Identifying hotspots for antibiotic resistance emergence and selection, and elucidating pathways to human exposure: Application of a systems-thinking approach to aquaculture systems	2019	1344-1356	Sci Total Environ	687		amr--env--opinion--risk--wild--identifying-hotspots-for-antibiotic-resistance-emergence-and-selection,-and-elucidating-pathways-to-human-exposure:-application-of-a-systems-thinking-approach-to-aquaculture-systems	16/08/2019
Journal Article	Insights about the Epidemiology of Dog Bites in a Canadian City Using a Dog Aggression Scale and Administrative Data	2019		Animals (Basel)	9	6	eval--human--non-bio--insights-about-the-epidemiology-of-dog-bites-in-a-canadian-city-using-a-dog-aggression-scale-and-administrative-data	09/06/2019
Journal Article	Antibiotic resistance in foodborne bacteria	2019	41-44	Trends in Food Science & Technology	84		amr--opinion--antibiotic-resistance-in-foodborne-bacteria	
Journal Article	A One Health investigation of Salmonella enterica serovar Wangata in north-eastern New South Wales, Australia, 2016-2017	2019	11	Epidemiology and Infection	147		data--human--pathogen--risk--wild--a-one-health-investigation-of-salmonella-enterica-serovar-wangata-in-north-eastern-new-south-wales,-australia,-2016-2017	
Journal Article	One Health Approach Reveals the Absence of Methicillin-Resistant Staphylococcus aureus in Autochthonous Cattle and Their Environments	2019	5	Frontiers in Microbiology	10		data--domestic--env--human--pathogen--one-health-approach-reveals-the-absence-of-methicillin-resistant-staphylococcus-aureus-in-autochthonous-cattle-and-their-environments	
Journal Article	Understanding the Epidemiology of Multi-Drug Resistant Gram-Negative Bacilli in the Middle East Using a One Health Approach	2019	1941	Front Microbiol	10		amr--human--opinion--understanding-the-epidemiology-of-multi-drug-resistant-gram-negative-bacilli-in-the-middle-east-using-a-one-health-approach	12/09/2019
Journal Article	Shiga toxin-producing Escherichia coli in wild ungulates	2019	203-209	Science of the Total Environment	651		data--pathogen--wild--shiga-toxin-producing-escherichia-coli-in-wild-ungulates	
Journal Article	Strategies for tackling Taenia solium taeniosis/cysticercosis: A systematic review and comparison of transmission models, including an assessment of the wider Taeniidae family transmission models	2019	e0007301	PLoS Negl Trop Dis	13	4	data--pathogen--risk--strategies-for-tackling-taenia-solium-taeniosis/cysticercosis:-a-systematic-review-and-comparison-of-transmission-models,-including-an-assessment-of-the-wider-taeniidae-family-transmission-models	11/04/2019
Journal Article	One Health and Antibiotic Resistance in Agroecosystems	2019	414-419	Ecohealth	16	3	env--opinion--one-health-and-antibiotic-resistance-in-agroecosystems	16/03/2018
Journal Article	Factors that enable effective One Health collaborations - A scoping review of the literature	2019	e0224660	PLoS One	14	12	eval--pathogen--factors-that-enable-effective-one-health-collaborations---a-scoping-review-of-the-literature	05/12/2019
Journal Article	One Health in Action: Operational Aspects of an Integrated Surveillance System for Zoonoses in Western Kenya	2019	13	Frontiers in Veterinary Science	6		eval--human--pathogen--risk--one-health-in-action:-operational-aspects-of-an-integrated-surveillance-system-for-zoonoses-in-western-kenya	
Journal Article	Qatar experience on One Health approach for middle-east respiratory syndrome coronavirus, 2012-2017: A viewpoint	2019	100090	One Health	7		edu--eval--pathogen--policy--qatar-experience-on-one-health-approach-for-middle-east-respiratory-syndrome-coronavirus,-2012-2017:-a-viewpoint	24/04/2019
Journal Article	Unbiased Assessment of Abundance of Rhipicephalus sanguineus sensu lato Ticks, Canine Exposure to Spotted Fever Group Rickettsia, and Risk Factors in Mexicali, Mexico	2019	22-32	American Journal of Tropical Medicine and Hygiene	101	1	eval--human--pathogen--unbiased-assessment-of-abundance-of-rhipicephalus-sanguineus-sensu-lato-ticks,-canine-exposure-to-spotted-fever-group-rickettsia,-and-risk-factors-in-mexicali,-mexico	
Journal Article	Gender Analysis for One Health: Theoretical Perspectives and Recommendations for Practice	2019	306-316	Ecohealth	16	2	edu--gender-analysis-for-one-health:-theoretical-perspectives-and-recommendations-for-practice	25/04/2019
Journal Article	Emergence and Spread of Extended Spectrum β-Lactamase Producing Enterobacteriaceae (ESBL-PE) in Pigs and Exposed Workers: A Multicentre Comparative Study between Cameroon and South Africa	2019		Pathogens	8	1	amr--data--domestic--human--risk--emergence-and-spread-of-extended-spectrum-β-lactamase-producing-enterobacteriaceae-(esbl-pe)-in-pigs-and-exposed-workers:-a-multicentre-comparative-study-between-cameroon-and-south-africa	19/01/2019
Journal Article	A one health perspective on dairy production and dairy food safety	2019	100086	One Health	7		domestic--opinion--a-one-health-perspective-on-dairy-production-and-dairy-food-safety	27/03/2019
Journal Article	Whole Genome Sequencing: Bridging One-Health Surveillance of Foodborne Diseases	2019	11	Frontiers in Public Health	7		opinion--pathogen--policy--whole-genome-sequencing:-bridging-one-health-surveillance-of-foodborne-diseases	
Journal Article	EcoHealth and One Health: A theory-focused review in response to calls for convergence	2019	105058	Environ Int	132		opinion--ecohealth-and-one-health:-a-theory-focused-review-in-response-to-calls-for-convergence	02/09/2019
Journal Article	Disease epidemic and a marine heat wave are associated with the continental-scale collapse of a pivotal predator (Pycnopodia helianthoides)	2019	eaau7042	Sci Adv	5	1	env--opinion--pathogen--disease-epidemic-and-a-marine-heat-wave-are-associated-with-the-continental-scale-collapse-of-a-pivotal-predator-(pycnopodia-helianthoides)	08/02/2019
Journal Article	Seasonal variation and persistence of tungiasis infestation in dogs in an endemic community, Bahia State (Brazil): longitudinal study	2019	1711-1718	Parasitol Res	118	6	data--domestic--pathogen--seasonal-variation-and-persistence-of-tungiasis-infestation-in-dogs-in-an-endemic-community,-bahia-state-(brazil):-longitudinal-study	13/04/2019
Journal Article	Some One Health based control strategies for the Middle East respiratory syndrome coronavirus	2019	100102	One Health	8		domestic--opinion--pathogen--some-one-health-based-control-strategies-for-the-middle-east-respiratory-syndrome-coronavirus	06/09/2019
Journal Article	Advancing Planetary Health in Australia: focus on emerging infections and antimicrobial resistance	2019	e001283	BMJ Glob Health	4	2	opinion--advancing-planetary-health-in-australia:-focus-on-emerging-infections-and-antimicrobial-resistance	30/05/2019
Journal Article	Organic ultraviolet filters in nearshore waters and in the invasive lionfish (Pterois volitans) in Grenada, West Indies	2019	11	PLoS One	14	7	data--env--non-bio--organic-ultraviolet-filters-in-nearshore-waters-and-in-the-invasive-lionfish-(pterois-volitans)-in-grenada,-west-indies	
Journal Article	One health in the circumpolar North	2019	1607502	Int J Circumpolar Health	78	1	human--opinion--one-health-in-the-circumpolar-north	27/04/2019
Journal Article	Epidemiology and antibiotic susceptibility of Vibrio cholerae associated with the 2017 outbreak in Kasese district, Uganda	2019	1405	BMC Public Health	19	1	data--human--pathogen--epidemiology-and-antibiotic-susceptibility-of-vibrio-cholerae-associated-with-the-2017-outbreak-in-kasese-district,-uganda	31/10/2019
Journal Article	Public preferences for One Health approaches to emerging infectious diseases: A discrete choice experiment	2019	164-171	Soc Sci Med	228		eval--human--public-preferences-for-one-health-approaches-to-emerging-infectious-diseases:-a-discrete-choice-experiment	29/03/2019
Journal Article	Opportunities for One Health policies to reduce poverty	2019	135-144	Rev Sci Tech	38	1	opinion--opportunities-for-one-health-policies-to-reduce-poverty	01/10/2019
Journal Article	Epidemiology of dog-mediated zoonotic diseases in Algeria: a One Health control approach	2019	17-20	New Microbes New Infect	28		data--domestic--human--pathogen--epidemiology-of-dog-mediated-zoonotic-diseases-in-algeria:-a-one-health-control-approach	26/02/2019
Journal Article	One Health approach in the prevention and control of mycobacterial infections in Tanzania: lessons learnt and future perspectives	2019	2	One Health Outlook	1		opinion--one-health-approach-in-the-prevention-and-control-of-mycobacterial-infections-in-tanzania:-lessons-learnt-and-future-perspectives	27/11/2019
Journal Article	Comparative knowledge, attitudes, and practices regarding anthrax, brucellosis, and rabies in three districts of northern Tanzania	2019	14	BMC Public Health	19	1	eval--human--pathogen--comparative-knowledge,-attitudes,-and-practices-regarding-anthrax,-brucellosis,-and-rabies-in-three-districts-of-northern-tanzania	
Journal Article	Expanding beyond zoonoses: the benefits of a national One Health coordination mechanism to address antimicrobial resistance and other shared health threats at the human animal environment interface in Kenya	2019	155-171	Revue Scientifique Et Technique-Office International Des Epizooties	38	1	eval--human--policy--expanding-beyond-zoonoses:-the-benefits-of-a-national-one-health-coordination-mechanism-to-address-antimicrobial-resistance-and-other-shared-health-threats-at-the-human-animal-environment-interface-in-kenya	
Journal Article	Genomic Delineation of Zoonotic Origins of Clostridium difficile	2019	164	Front Public Health	7		opinion--pathogen--genomic-delineation-of-zoonotic-origins-of-clostridium-difficile	10/07/2019
Journal Article	Optimization of Electronic Medical Records for Data Mining Using a Common Data Model	2019	100364	Top Companion Anim Med	37		domestic--eval--human--policy--optimization-of-electronic-medical-records-for-data-mining-using-a-common-data-model	16/12/2019
Journal Article	First evidence of a new simian adenovirus clustering with Human mastadenovirus F viruses	2019	147	Virol J	16	1	human--pathogen--risk--wild--first-evidence-of-a-new-simian-adenovirus-clustering-with-human-mastadenovirus-f-viruses	30/11/2019
Journal Article	An integrated health delivery platform, targeting soil-transmitted helminths (STH) and canine mediated human rabies, results in cost savings and increased breadth of treatment for STH in remote communities in Tanzania	2019	1398	BMC Public Health	19	1	eval--human--pathogen--policy--an-integrated-health-delivery-platform,-targeting-soil-transmitted-helminths-(sth)-and-canine-mediated-human-rabies,-results-in-cost-savings-and-increased-breadth-of-treatment-for-sth-in-remote-communities-in-tanzania	30/10/2019
Journal Article	Owners' Attitudes toward Their Companion Dogs Are Associated with the Owners' Depression Symptoms-An Exploratory Study in South Korea	2019		Int J Environ Res Public Health	16	19	eval--human--non-bio--owners'-attitudes-toward-their-companion-dogs-are-associated-with-the-owners'-depression-symptoms-an-exploratory-study-in-south-korea	27/09/2019
Journal Article	A framework for improved one health governance and policy making for antimicrobial use	2019	6	Bmj Global Health	4	5	amr--opinion--a-framework-for-improved-one-health-governance-and-policy-making-for-antimicrobial-use	
Journal Article	Antimicrobial resistance from a one health perspective in Cameroon: a systematic review and meta-analysis	2019	1135	BMC Public Health	19	1	amr--data--domestic--human--antimicrobial-resistance-from-a-one-health-perspective-in-cameroon:-a-systematic-review-and-meta-analysis	21/08/2019
Journal Article	Epidemiology of hepatitis E in South-East Europe in the "One Health" concept	2019	3168-3182	World Journal of Gastroenterology	25	25	data--domestic--env--human--pathogen--wild--epidemiology-of-hepatitis-e-in-south-east-europe-in-the-"one-health"-concept	
Journal Article	Attributable sources of community-acquired carriage of Escherichia coli containing beta-lactam antibiotic resistance genes: a population-based modelling study	2019	E357-E369	Lancet Planetary Health	3	8	amr--risk--attributable-sources-of-community-acquired-carriage-of-escherichia-coli-containing-beta-lactam-antibiotic-resistance-genes:-a-population-based-modelling-study	
Journal Article	Serological and Molecular Investigation on Toxoplasma gondii Infection in Wild Birds	2019		Pathogens	8	2	data--pathogen--wild--serological-and-molecular-investigation-on-toxoplasma-gondii-infection-in-wild-birds	01/05/2019
Journal Article	HEVnet: a One Health, collaborative, interdisciplinary network and sequence data repository for enhanced hepatitis E virus molecular typing, characterisation and epidemiological investigations	2019	42-47	Eurosurveillance	24	10	eval--pathogen--policy--hevnet:-a-one-health,-collaborative,-interdisciplinary-network-and-sequence-data-repository-for-enhanced-hepatitis-e-virus-molecular-typing,-characterisation-and-epidemiological-investigations	
Journal Article	Understanding temporal and spatial variations of viral disease in the US: The need for a one-health-based data collection and analysis approach	2019	100105	One Health	8		opinion--pathogen--understanding-temporal-and-spatial-variations-of-viral-disease-in-the-us:-the-need-for-a-one-health-based-data-collection-and-analysis-approach	12/11/2019
Journal Article	A water-focused one-health approach for early detection and prevention of viral outbreaks	2019	9	One Health	7		human--opinion--pathogen--a-water-focused-one-health-approach-for-early-detection-and-prevention-of-viral-outbreaks	
Journal Article	Emerging infectious diseases and biological invasions: a call for a One Health collaboration in science and management	2019	181577	R Soc Open Sci	6	3	opinion--pathogen--emerging-infectious-diseases-and-biological-invasions:-a-call-for-a-one-health-collaboration-in-science-and-management	30/04/2019
Journal Article	Identifying climate-sensitive infectious diseases in animals and humans in Northern regions	2019	53	Acta Vet Scand	61	1	climate--data--identifying-climate-sensitive-infectious-diseases-in-animals-and-humans-in-northern-regions	16/11/2019
Journal Article	A systematic review and meta-analysis of canine, feline and human Toxocara infections in sub-Saharan Africa	2019	e96	J Helminthol	94		data--domestic--pathogen--a-systematic-review-and-meta-analysis-of-canine,-feline-and-human-toxocara-infections-in-sub-saharan-africa	05/11/2019
Journal Article	Highly variable vancomycin-resistant enterococci in the north-eastern part of the Czech Republic	2019	16-22	Lett Appl Microbiol	69	1	amr--data--highly-variable-vancomycin-resistant-enterococci-in-the-north-eastern-part-of-the-czech-republic	31/01/2019
Journal Article	Loggerhead sea turtles as sentinels in the western Mediterranean: antibiotic resistance and environment-related modifications of Gram-negative bacteria	2019	110575	Mar Pollut Bull	149		data--pathogen--wild--loggerhead-sea-turtles-as-sentinels-in-the-western-mediterranean:-antibiotic-resistance-and-environment-related-modifications-of-gram-negative-bacteria	25/09/2019
Journal Article	Synergising tools for capacity assessment and One Health operationalisation	2019	71-89	Revue Scientifique Et Technique-Office International Des Epizooties	38	1	eval--opinion--policy--synergising-tools-for-capacity-assessment-and-one-health-operationalisation	
Journal Article	Toward a quantification of risks at the nexus of conservation and health: The case of bushmeat markets in Lao PDR	2019	732-745	Science of the Total Environment	676		human--pathogen--risk--toward-a-quantification-of-risks-at-the-nexus-of-conservation-and-health:-the-case-of-bushmeat-markets-in-lao-pdr	
Journal Article	Local applications but global implications: Can pesticides drive microorganisms to develop antimicrobial resistance?	2019	177-189	Science of the Total Environment	654		env--non-bio--opinion--local-applications-but-global-implications:-can-pesticides-drive-microorganisms-to-develop-antimicrobial-resistance?	
Journal Article	Surveillance of Mosquitoes (Diptera, Culicidae) in a Northern Central Region of Spain: Implications for the Medical Community	2019	9	Frontiers in Veterinary Science	6		eval--pathogen--policy--surveillance-of-mosquitoes-(diptera,-culicidae)-in-a-northern-central-region-of-spain:-implications-for-the-medical-community	
Journal Article	Molecular epidemiology of Cryptosporidium spp. in calves in Estonia: high prevalence of Cryptosporidium parvum shedding and 10 subtypes identified	2019	261-267	Parasitology	146	2	data--domestic--pathogen--molecular-epidemiology-of-cryptosporidium-spp.-in-calves-in-estonia:-high-prevalence-of-cryptosporidium-parvum-shedding-and-10-subtypes-identified	
Journal Article	Epidemiology of Taenia saginata taeniosis/cysticercosis: a systematic review of the distribution in the Middle East and North Africa	2019	113	Parasit Vectors	12	1	data--domestic--pathogen--epidemiology-of-taenia-saginata-taeniosis/cysticercosis:-a-systematic-review-of-the-distribution-in-the-middle-east-and-north-africa	17/03/2019
Journal Article	Odds, challenges and new approaches in the control of helminthiasis, an Asian study	2019	9	Parasite Epidemiology and Control	4		opinion--pathogen--odds,-challenges-and-new-approaches-in-the-control-of-helminthiasis,-an-asian-study	
Journal Article	Amphibian fungal panzootic causes catastrophic and ongoing loss of biodiversity	2019	1459-1463	Science	363	6434	opinion--pathogen--wild--amphibian-fungal-panzootic-causes-catastrophic-and-ongoing-loss-of-biodiversity	30/03/2019
Journal Article	Long-Tailed Macaques (Macaca fascicularis) in Urban Landscapes: Gastrointestinal Parasitism and Barriers for Healthy Coexistence in Northeast Thailand	2019	357-364	American Journal of Tropical Medicine and Hygiene	100	2	data--pathogen--wild--long-tailed-macaques-(macaca-fascicularis)-in-urban-landscapes:-gastrointestinal-parasitism-and-barriers-for-healthy-coexistence-in-northeast-thailand	
Journal Article	Critically important antibiotics: criteria and approaches for measuring and reducing their use in food animal agriculture	2019	8-16	Annals of the New York Academy of Sciences	1441	1	amr--opinion--critically-important-antibiotics:-criteria-and-approaches-for-measuring-and-reducing-their-use-in-food-animal-agriculture	
Journal Article	Integrated aquaculture contributes to the transfer of mcr-1 between animals and humans via the aquaculture supply chain	2019	10	Environment International	130		amr--data--env--integrated-aquaculture-contributes-to-the-transfer-of-mcr-1-between-animals-and-humans-via-the-aquaculture-supply-chain	
Journal Article	Peridomestic small Indian mongoose: An invasive species posing as potential zoonotic risk for leptospirosis in the Caribbean	2019	166-170	Acta Tropica	190		data--pathogen--wild--peridomestic-small-indian-mongoose:-an-invasive-species-posing-as-potential-zoonotic-risk-for-leptospirosis-in-the-caribbean	
Journal Article	Divergent geography of Salmonella Wangata and Salmonella Typhimurium epidemiology in New South Wales, Australia	2019	100092	One Health	7		climate--human--opinion--pathogen--divergent-geography-of-salmonella-wangata-and-salmonella-typhimurium-epidemiology-in-new-south-wales,-australia	25/04/2019
Journal Article	Importance of a One Health approach in advancing global health security and the Sustainable Development Goals	2019	145-154	Revue Scientifique Et Technique-Office International Des Epizooties	38	1	opinion--importance-of-a-one-health-approach-in-advancing-global-health-security-and-the-sustainable-development-goals	
Journal Article	Combating Antimicrobial Resistance in Singapore: A Qualitative Study Exploring the Policy Context, Challenges, Facilitators, and Proposed Strategies	2019		Antibiotics (Basel)	8	4	amr--human--policy--combating-antimicrobial-resistance-in-singapore:-a-qualitative-study-exploring-the-policy-context,-challenges,-facilitators,-and-proposed-strategies	02/11/2019
Journal Article	Integration of wildlife and environmental health into a One Health approach	2019	91-102	Revue Scientifique Et Technique-Office International Des Epizooties	38	1	climate--opinion--integration-of-wildlife-and-environmental-health-into-a-one-health-approach	
Journal Article	Antibiotic-resistant Escherichia coli and Salmonella spp. associated with dairy cattle and farm environment having public health significance	2019	984-993	Veterinary World	12	7	amr--data--domestic--antibiotic-resistant-escherichia-coli-and-salmonella-spp.-associated-with-dairy-cattle-and-farm-environment-having-public-health-significance	
Journal Article	Assessing health systems in Guinea for prevention and control of priority zoonotic diseases: A One Health approach	2019	11	One Health	7		eval--pathogen--policy--assessing-health-systems-in-guinea-for-prevention-and-control-of-priority-zoonotic-diseases:-a-one-health-approach	
Journal Article	Toxoplasma gondii infection and toxoplasmosis in farm animals: Risk factors and economic impact	2019	32	Food and Waterborne Parasitology	15		domestic--opinion--pathogen--toxoplasma-gondii-infection-and-toxoplasmosis-in-farm-animals:-risk-factors-and-economic-impact	
Journal Article	Vector-borne nematode diseases in pets and humans in the Mediterranean Basin: An update	2019	1630-1643	Veterinary World	12	10	domestic--opinion--pathogen--vector-borne-nematode-diseases-in-pets-and-humans-in-the-mediterranean-basin:-an-update	
Journal Article	Examination of unintended consequences of antibiotic use restrictions in food-producing animals: Sub-analysis of a systematic review	2019	100095	One Health	7		amr--risk--examination-of-unintended-consequences-of-antibiotic-use-restrictions-in-food-producing-animals:-sub-analysis-of-a-systematic-review	14/06/2019
Journal Article	The Mandate for a Global "One Health" Approach to Antimicrobial Resistance Surveillance	2019	227-228	Am J Trop Med Hyg	100	2	the-mandate-for-a-global-"one-health"-approach-to-antimicrobial-resistance-surveillance	05/01/2019
Journal Article	Control of Taenia solium; A Case for Public and Private Sector Investment	2019	10	Frontiers in Veterinary Science	6		pathogen--risk--control-of-taenia-solium;-a-case-for-public-and-private-sector-investment	
Journal Article	Antibiotic Resistance Genes in the Human-Impacted Environment: A One Health Perspective	2019	273-282	Pedosphere	29	3	opinion--policy--antibiotic-resistance-genes-in-the-human-impacted-environment:-a-one-health-perspective	
Journal Article	Transboundary animal diseases as re-emerging threats - Impact on one health	2019	193-196	Seminars in Diagnostic Pathology	36	3	transboundary-animal-diseases-as-re-emerging-threats---impact-on-one-health	
Journal Article	Resistance in the Environmental Pathogenic Fungus Aspergillus fumigatus	2019	61-63	Medical Mycology Journal	60	3	opinion--pathogen--resistance-in-the-environmental-pathogenic-fungus-aspergillus-fumigatus	
Journal Article	Lead pollution from hunting ammunition in Argentina and current state of lead shot replacement efforts	2019	1015-1022	Ambio	48	9	env--non-bio--opinion--wild--lead-pollution-from-hunting-ammunition-in-argentina-and-current-state-of-lead-shot-replacement-efforts	11/04/2019
Journal Article	One Health - Cycling of diverse microbial communities as a connecting force for soil, plant, animal, human and ecosystem health	2019	927-937	Science of the Total Environment	664		opinion--one-health---cycling-of-diverse-microbial-communities-as-a-connecting-force-for-soil,-plant,-animal,-human-and-ecosystem-health	
Journal Article	One Health as a moral dilemma: Towards a socially responsible zoonotic disease control	2019	26-34	Zoonoses and Public Health	66	1	policy--one-health-as-a-moral-dilemma:-towards-a-socially-responsible-zoonotic-disease-control	
Journal Article	Strengthening multi-sectoral collaboration on critical health issues: One Health Systems Mapping and Analysis Resource Toolkit (OH-SMART) for operationalizing One Health	2019	e0219197	PLoS One	14	7	eval--strengthening-multi-sectoral-collaboration-on-critical-health-issues:-one-health-systems-mapping-and-analysis-resource-toolkit-(oh-smart)-for-operationalizing-one-health	06/07/2019
Journal Article	Viral haemorrhagic fevers in the Middle East	2019	185-198	Revue Scientifique Et Technique-Office International Des Epizooties	38	1	opinion--pathogen--viral-haemorrhagic-fevers-in-the-middle-east	
Journal Article	Metal Levels in Whales from the Gulf of Maine: A One Environmental Health approach	2019	653-660	Chemosphere	216		data--non-bio--wild--metal-levels-in-whales-from-the-gulf-of-maine:-a-one-environmental-health-approach	
Journal Article	Molecular Identification of a Novel Hantavirus in Malaysian Bronze Tube-Nosed Bats (Murina aenea)	2019	8	Viruses-Basel	11	10	data--pathogen--wild--molecular-identification-of-a-novel-hantavirus-in-malaysian-bronze-tube-nosed-bats-(murina-aenea)	
Journal Article	One health approach to address zoonotic diseases	2020	6-8	Indian Journal of Community Medicine	45		opinion--policy--one-health-approach-to-address-zoonotic-diseases	
Journal Article	Antimicrobial resistance situation in animal health of Bangladesh	2020	2713-2727	Veterinary World	13	12	opinion--policy--risk--antimicrobial-resistance-situation-in-animal-health-of-bangladesh	
Journal Article	The Pearl River Declaration: a timely call for enhancing health security through fostering a regional one health collaboration in the Asia-Pacific	2020	79	Global Health	16	1	eval--human--policy--the-pearl-river-declaration:-a-timely-call-for-enhancing-health-security-through-fostering-a-regional-one-health-collaboration-in-the-asia-pacific	08/09/2020
Journal Article	Challenge infection model for MERS-CoV based on naturally infected camels	2020	77	Virol J	17	1	domestic--pathogen--risk--challenge-infection-model-for-mers-cov-based-on-naturally-infected-camels	20/06/2020
Journal Article	Colistin Update on Its Mechanism of Action and Resistance, Present and Future Challenges	2020	12	Microorganisms	8	11	amr--opinion--policy--colistin-update-on-its-mechanism-of-action-and-resistance,-present-and-future-challenges	
Journal Article	Integrating epidemiological and economic models to identify the cost of foodborne diseases	2020	107832	Exp Parasitol	210		opinion--risk--integrating-epidemiological-and-economic-models-to-identify-the-cost-of-foodborne-diseases	01/02/2020
Journal Article	Application of one health approach in training at Makerere University: experiences from the one health workforce project in Uganda	2020	9	One Health Outlook	2	1	edu--application-of-one-health-approach-in-training-at-makerere-university:-experiences-from-the-one-health-workforce-project-in-uganda	
Journal Article	Extended-spectrum ß-lactamase-producing Escherichia coli among humans, chickens and poultry environments in Abuja, Nigeria	2020	8	One Health Outlook	2		data--domestic--human--pathogen--extended-spectrum-ß-lactamase-producing-escherichia-coli-among-humans,-chickens-and-poultry-environments-in-abuja,-nigeria	09/04/2021
Journal Article	Genome-Based Analysis of Extended-Spectrum β-Lactamase-Producing Escherichia coli in the Aquatic Environment and Nile Perch (Lates niloticus) of Lake Victoria, Tanzania	2020	108	Front Microbiol	11		amr--data--wild--genome-based-analysis-of-extended-spectrum-β-lactamase-producing-escherichia-coli-in-the-aquatic-environment-and-nile-perch-(lates-niloticus)-of-lake-victoria,-tanzania	11/03/2020
Journal Article	Genetic uniformity, geographical spread and anthropogenic habitat modifications of lymnaeid vectors found in a One Health initiative in the highest human fascioliasis hyperendemic of the Bolivian Altiplano	2020	19	Parasites & Vectors	13	1	climate--data--pathogen--genetic-uniformity,-geographical-spread-and-anthropogenic-habitat-modifications-of-lymnaeid-vectors-found-in-a-one-health-initiative-in-the-highest-human-fascioliasis-hyperendemic-of-the-bolivian-altiplano	
Journal Article	Practising co-production and interdisciplinarity: Challenges and implications for one health research	2020	7	Preventive Veterinary Medicine	177		opinion--policy--practising-co-production-and-interdisciplinarity:-challenges-and-implications-for-one-health-research	
Journal Article	Antimicrobial Resistance Traits of Escherichia coli Isolated from Dairy Manure and Freshwater Ecosystems Are Similar to One Another but Differ from Associated Clinical Isolates	2020		Microorganisms	8	5	amr--data--domestic--env--human--antimicrobial-resistance-traits-of-escherichia-coli-isolated-from-dairy-manure-and-freshwater-ecosystems-are-similar-to-one-another-but-differ-from-associated-clinical-isolates	21/05/2020
Journal Article	Seroprevalence of Toxoplasma gondii in wild boar and deer in Brandenburg, Germany	2020	601-606	Zoonoses and Public Health	67	6	data--pathogen--wild--seroprevalence-of-toxoplasma-gondii-in-wild-boar-and-deer-in-brandenburg,-germany	
Journal Article	One Health education in Kakuma refugee camp (Kenya): From a MOOC to projects on real world challenges	2020	100158	One Health	10		edu--eval--human--one-health-education-in-kakuma-refugee-camp-(kenya):-from-a-mooc-to-projects-on-real-world-challenges	28/08/2020
Journal Article	Characteristics of One Health surveillance systems: A systematic literature review	2020	104560	Prev Vet Med	181		data--eval--policy--characteristics-of-one-health-surveillance-systems:-a-systematic-literature-review	12/12/2018
Journal Article	Operationalizing the One Health Approach in Uganda: Challenges and Opportunities	2020	250-257	J Epidemiol Glob Health	10	4	eval--human--policy--operationalizing-the-one-health-approach-in-uganda:-challenges-and-opportunities	04/10/2020
Journal Article	Spatial epidemiological patterns suggest mechanisms of land-sea transmission for Sarcocystis neurona in a coastal marine mammal	2020	3683	Sci Rep	10	1	data--pathogen--wild--spatial-epidemiological-patterns-suggest-mechanisms-of-land-sea-transmission-for-sarcocystis-neurona-in-a-coastal-marine-mammal	01/03/2020
Journal Article	Epidemiological study of Giardia duodenalis infection in companion dogs from the metropolitan area of São Paulo Brazil	2020	765-773	Zoonoses Public Health	67	7	data--domestic--pathogen--epidemiological-study-of-giardia-duodenalis-infection-in-companion-dogs-from-the-metropolitan-area-of-são-paulo-brazil	30/04/2020
Journal Article	Zoonosis: An Emerging Link to Antibiotic Resistance Under "One Health Approach"	2020	139-152	Indian J Microbiol	60	2	amr--data--domestic--risk--zoonosis:-an-emerging-link-to-antibiotic-resistance-under-"one-health-approach"	08/04/2020
Journal Article	One Health in hospitals: how understanding the dynamics of people, animals, and the hospital built-environment can be used to better inform interventions for antimicrobial-resistant gram-positive infections	2020	78	Antimicrob Resist Infect Control	9	1	human--opinion--pathogen--one-health-in-hospitals:-how-understanding-the-dynamics-of-people,-animals,-and-the-hospital-built-environment-can-be-used-to-better-inform-interventions-for-antimicrobial-resistant-gram-positive-infections	04/06/2020
Journal Article	Risks associated with animal-assisted intervention programs: A literature review	2020	101145	Complement Ther Clin Pract	39		human--opinion--pathogen--risk--risks-associated-with-animal-assisted-intervention-programs:-a-literature-review	08/05/2020
Journal Article	Recalibrating Veterinary Medicine through Animal Welfare Science and Ethics for the 2020s	2020		Animals (Basel)	10	4	edu--eval--human--recalibrating-veterinary-medicine-through-animal-welfare-science-and-ethics-for-the-2020s	15/04/2020
Journal Article	Multiple New Paralytic Shellfish Toxin Vectors in Offshore North Sea Benthos, a Deep Secret Exposed	2020		Mar Drugs	18	8	data--domestic--non-bio--wild--multiple-new-paralytic-shellfish-toxin-vectors-in-offshore-north-sea-benthos,-a-deep-secret-exposed	06/08/2020
Journal Article	Leatherback sea turtle (Dermochelys coriacea) hatch success and essential and nonessential metals in eggs and embryos from nests in St. Kitts (2015)	2020	111726	Mar Pollut Bull	161	Pt A	data--non-bio--wild--leatherback-sea-turtle-(dermochelys-coriacea)-hatch-success-and-essential-and-nonessential-metals-in-eggs-and-embryos-from-nests-in-st.-kitts-(2015)	13/10/2020
Journal Article	Sampling scale and season influence the observed relationship between the density of deer and questing Ixodes ricinus nymphs	2020	493	Parasit Vectors	13	1	data--pathogen--risk--wild--sampling-scale-and-season-influence-the-observed-relationship-between-the-density-of-deer-and-questing-ixodes-ricinus-nymphs	01/10/2020
Journal Article	Emergence and Spread of Different ESBL-Producing Salmonella enterica Serovars in Hospitalized Horses Sharing a Highly Transferable IncM2 CTX-M-3-Encoding Plasmid	2020	616032	Front Microbiol	11		data--domestic--pathogen--emergence-and-spread-of-different-esbl-producing-salmonella-enterica-serovars-in-hospitalized-horses-sharing-a-highly-transferable-incm2-ctx-m-3-encoding-plasmid	05/01/2021
Journal Article	Environmental and health crises: Anthropocene diseases that call for redesigning our food system	2020	10	Cahiers Agricultures	29		opinion--environmental-and-health-crises:-anthropocene-diseases-that-call-for-redesigning-our-food-system	
Journal Article	Brucellosis in ruminants and pastoralists in Borena, Southern Ethiopia	2020	17	Plos Neglected Tropical Diseases	14	7	domestic--human--pathogen--risk--brucellosis-in-ruminants-and-pastoralists-in-borena,-southern-ethiopia	
Journal Article	Short report on implications of Covid-19 and emerging zoonotic infectious diseases for pastoralists and Africa	2020	12	Pastoralism	10	1	covid--opinion--short-report-on-implications-of-covid-19-and-emerging-zoonotic-infectious-diseases-for-pastoralists-and-africa	17/06/2020

Journal Article	Volunteer based approach to dog vaccination campaigns to eliminate human rabies: Lessons from Laikipia County, Kenya	2020	24	Plos Neglected Tropical Diseases	14	7	domestic--eval--human--pathogen--volunteer-based-approach-to-dog-vaccination-campaigns-to-eliminate-human-rabies:-lessons-from-laikipia-county,-kenya			
Journal Article	HEALTH ASSESSMENT OF WILD LOWLAND TAPIRS (TAPIRUS TERRESTRIS) IN THE HIGHLY THREATENED CERRADO BIOME, BRAZIL	2020	34-46	J Wildl Dis	56	1	data--non-bio--wild--health-assessment-of-wild-lowland-tapirs-(tapirus-terrestris)-in-the-highly-threatened-cerrado-biome,-brazil	19/09/2019		
Journal Article	Teaching the basics of the One Health concept to undergraduate veterinary students	2020	219-225	Research in Veterinary Science	133		edu--eval--human--teaching-the-basics-of-the-one-health-concept-to-undergraduate-veterinary-students			
Journal Article	Foundational health content in environmental studies, sciences, and sustainability education: report of a workshop	2020	327-333	Journal of Environmental Studies and Sciences	10	3	edu--human--policy--foundational-health-content-in-environmental-studies,-sciences,-and-sustainability-education:-report-of-a-workshop			
Journal Article	Helping to heal nature and ourselves through human-rights-based and gender-responsive One Health	2020	22	One Health Outlook	2	1	human--opinion--policy--helping-to-heal-nature-and-ourselves-through-human-rights-based-and-gender-responsive-one-health	24/11/2020		
Journal Article	Emerging contaminants affect the microbiome of water systems-strategies for their mitigation	2020	11	Npj Clean Water	3	1	env--eval--non-bio--opinion--emerging-contaminants-affect-the-microbiome-of-water-systems-strategies-for-their-mitigation			
Journal Article	Operationalization of One Health and tripartite collaboration in the Asia-Pacific region	2020	21-25	WHO South East Asia J Public Health	9	1	edu--eval--human--pathogen--operationalization-of-one-health-and-tripartite-collaboration-in-the-asia-pacific-region	29/04/2020		
Journal Article	COVID-19-Zoonosis or Emerging Infectious Disease?	2020	596944	Front Public Health	8		covid--opinion--risk--covid-19-zoonosis-or-emerging-infectious-disease?	17/12/2020		
Journal Article	One Health Aotearoa: a transdisciplinary initiative to improve human, animal and environmental health in New Zealand	2020	4	One Health Outlook	2	1	opinion--policy--one-health-aotearoa:-a-transdisciplinary-initiative-to-improve-human,-animal-and-environmental-health-in-new-zealand	25/08/2020		
Journal Article	Reflecting on One Health in Action During the COVID-19 Response	2020	6	Frontiers in Veterinary Science	7		covid--eval--opinion--reflecting-on-one-health-in-action-during-the-covid-19-response			
Journal Article	Food Safety Considerations Related to the Consumption and Handling of Game Meat in North America	2020		Vet Sci	7	4	human--opinion--pathogen--policy--food-safety-considerations-related-to-the-consumption-and-handling-of-game-meat-in-north-america	02/12/2020		
Journal Article	The SARS-CoV-2 outbreak from a one health perspective	2020	6	One Health	10		covid--opinion--the-sars-cov-2-outbreak-from-a-one-health-perspective			
Journal Article	Tackling antimicrobial resistance in Bangladesh: A scoping review of policy and practice in human, animal and environment sectors	2020	e0227947	PLoS One	15	1	amr--data--tackling-antimicrobial-resistance-in-bangladesh:-a-scoping-review-of-policy-and-practice-in-human,-animal-and-environment-sectors	28/01/2020		
Journal Article	Between roost contact is essential for maintenance of European bat lyssavirus type-2 in Myotis daubentonii bat reservoir: 'The Swarming Hypothesis'	2020	1740	Sci Rep	10	1	pathogen--risk--wild--between-roost-contact-is-essential-for-maintenance-of-european-bat-lyssavirus-type-2-in-myotis-daubentonii-bat-reservoir:-'the-swarming-hypothesis'	06/02/2020		
Journal Article	Fakes and chemicals: indigenous medicine in contemporary Kenya and implications for health equity	2020	199	Int J Equity Health	19	1	data--human--fakes-and-chemicals:-indigenous-medicine-in-contemporary-kenya-and-implications-for-health-equity	09/11/2020		
Journal Article	Symposium report: emerging threats for human health - impact of socioeconomic and climate change on zooanthroponosis in the Republic of Sakha (Yakutia), Russia	2020	1715698	Int J Circumpolar Health	79	1	climate--opinion--pathogen--symposium-report:-emerging-threats-for-human-health---impact-of-socioeconomic-and-climate-change-on-zooanthroponosis-in-the-republic-of-sakha-(yakutia),-russia	13/02/2020		
Journal Article	One Biosecurity: a unified concept to integrate human, animal, plant, and environmental health	2020	539-549	Emerg Top Life Sci	4	5	opinion--one-biosecurity:-a-unified-concept-to-integrate-human,-animal,-plant,-and-environmental-health	29/10/2020		
Journal Article	The state of One Health research across disciplines and sectors - a bibliometric analysis	2020	7	One Health	10		eval--opinion--the-state-of-one-health-research-across-disciplines-and-sectors---a-bibliometric-analysis			
Journal Article	Coronavirus testing indicates transmission risk increases along wildlife supply chains for human consumption in Viet Nam, 2013-2014	2020	e0237129	PLoS One	15	8	covid--data--pathogen--wild--coronavirus-testing-indicates-transmission-risk-increases-along-wildlife-supply-chains-for-human-consumption-in-viet-nam,-2013-2014	11/08/2020		
Journal Article	Drivers of Antibiotic Resistance Transmission in Low- and Middle-Income Countries from a "One Health" Perspective-A Review	2020	23	Antibiotics-Basel	9	7	amr--opinion--drivers-of-antibiotic-resistance-transmission-in-low--and-middle-income-countries-from-a-"one-health"-perspective-a-review			
Journal Article	Helminth Parasites among Rodents in the Middle East Countries: A Systematic Review and Meta-Analysis	2020		Animals (Basel)	10	12	data--pathogen--wild--helminth-parasites-among-rodents-in-the-middle-east-countries:-a-systematic-review-and-meta-analysis	16/12/2020		
Journal Article	Distribution of phenotypic and genotypic antimicrobial resistance and virulence genes in Vibrio parahaemolyticus isolated from cultivated oysters and estuarine water	2020		FEMS Microbiol Ecol	96	8	amr--data--wild--distribution-of-phenotypic-and-genotypic-antimicrobial-resistance-and-virulence-genes-in-vibrio-parahaemolyticus-isolated-from-cultivated-oysters-and-estuarine-water	03/05/2020		
Journal Article	Prevalence, Antibiotic-Resistance, and Virulence Characteristics of Vibrio parahaemolyticus in Restaurant Fish Tanks in Seoul, South Korea	2020	209-214	Foodborne Pathog Dis	17	3	data--domestic--pathogen--prevalence,-antibiotic-resistance,-and-virulence-characteristics-of-vibrio-parahaemolyticus-in-restaurant-fish-tanks-in-seoul,-south-korea	07/11/2019		
Journal Article	Global shifts in mammalian population trends reveal key predictors of virus spillover risk	2020	20192736	Proc Biol Sci	287	1924	opinion--pathogen--global-shifts-in-mammalian-population-trends-reveal-key-predictors-of-virus-spillover-risk	08/04/2020		
Journal Article	Distribution and antimicrobial resistance profiles of bacterial species in stray dogs, hospital-admitted dogs, and veterinary staff in South Korea	2020	12	Preventive Veterinary Medicine	184		amr--data--domestic--human--pathogen--distribution-and-antimicrobial-resistance-profiles-of-bacterial-species-in-stray-dogs,-hospital-admitted-dogs,-and-veterinary-staff-in-south-korea			
Journal Article	Prevalence and molecular characterization of Cryptosporidium species in poultry in Bangladesh	2020	100122	One Health	9		data--domestic--pathogen--prevalence-and-molecular-characterization-of-cryptosporidium-species-in-poultry-in-bangladesh	06/05/2020		
Journal Article	Lessons Learned From the Stakeholder Engagement in Research: Application of Spatial Analytical Tools in One Health Problems	2020	9	Frontiers in Veterinary Science	7		eval--human--policy--lessons-learned-from-the-stakeholder-engagement-in-research:-application-of-spatial-analytical-tools-in-one-health-problems			
Journal Article	Genetic diversity and risk factors for the transmission of antimicrobial resistance across human, animals and environmental compartments in East Africa: a review	2020	127	Antimicrob Resist Infect Control	9	1	amr--opinion--pathogen--genetic-diversity-and-risk-factors-for-the-transmission-of-antimicrobial-resistance-across-human,-animals-and-environmental-compartments-in-east-africa:-a-review	09/08/2020		
Journal Article	Assessment of Drivers of Antimicrobial Use and Resistance in Poultry and Domestic Pig Farming in the Msimbazi River Basin in Tanzania	2020	20	Antibiotics-Basel	9	12	amr--domestic--eval--human--policy--assessment-of-drivers-of-antimicrobial-use-and-resistance-in-poultry-and-domestic-pig-farming-in-the-msimbazi-river-basin-in-tanzania			
Journal Article	Antimicrobial use and resistance in food-producing animals and the environment: an African perspective	2020	37	Antimicrob Resist Infect Control	9	1	amr--eval--opinion--antimicrobial-use-and-resistance-in-food-producing-animals-and-the-environment:-an-african-perspective	04/03/2020		
Journal Article	Prevalence and Epidemiology of Multidrug-Resistant Pathogens in the Food Chain and the Urban Environment in Northwestern Germany	2020		Antibiotics (Basel)	9	10	amr--data--domestic--prevalence-and-epidemiology-of-multidrug-resistant-pathogens-in-the-food-chain-and-the-urban-environment-in-northwestern-germany	22/10/2020		
Journal Article	Spatial transmission risk during the 2007-2010 Q fever epidemic in The Netherlands: Analysis of the farm-to-farm and farm-to-resident transmission	2020	e0227491	PLoS One	15	2	domestic--pathogen--risk--spatial-transmission-risk-during-the-2007-2010-q-fever-epidemic-in-the-netherlands:-analysis-of-the-farm-to-farm-and-farm-to-resident-transmission	06/02/2020		
Journal Article	Demystifying and Demonstrating the Value of a One Health Approach to Parasitological Challenges	2020	109202	Vet Parasitol	287		eval--human--opinion--demystifying-and-demonstrating-the-value-of-a-one-health-approach-to-parasitological-challenges	13/10/2020		
Journal Article	Analysis of adenovirus DNA detected in rodent species from the Democratic Republic of the Congo indicates potentially novel adenovirus types	2020	100640	New Microbes New Infect	34		data--pathogen--wild--analysis-of-adenovirus-dna-detected-in-rodent-species-from-the-democratic-republic-of-the-congo-indicates-potentially-novel-adenovirus-types	07/02/2020		
Journal Article	Linking humans, their animals, and the environment again: a decolonized and more-than-human approach to "One Health"	2020	55	Parasite	27		human--opinion--linking-humans,-their-animals,-and-the-environment-again:-a-decolonized-and-more-than-human-approach-to-"one-health"	04/11/2020		
Journal Article	Prevalence and distribution of schistosomiasis in human, livestock, and snail populations in northern Senegal: a One Health epidemiological study of a multi-host system	2020	e330-e342	Lancet Planet Health	4	8	data--human--pathogen--wild--prevalence-and-distribution-of-schistosomiasis-in-human,-livestock,-and-snail-populations-in-northern-senegal:-a-one-health-epidemiological-study-of-a-multi-host-system	18/08/2020		
Journal Article	Lead seasonality in humans, animals, and the natural environment	2020	11	Environmental Research	180		non-bio--risk--lead-seasonality-in-humans,-animals,-and-the-natural-environment			
Journal Article	Clostridium difficile in soil conditioners, mulches and garden mixes with evidence of a clonal relationship with historical food and clinical isolates	2020	672-680	Environ Microbiol Rep	12	6	data--env--pathogen--clostridium-difficile-in-soil-conditioners,-mulches-and-garden-mixes-with-evidence-of-a-clonal-relationship-with-historical-food-and-clinical-isolates	26/09/2020		
Journal Article	Manure as a Potential Hotspot for Antibiotic Resistance Dissemination by Horizontal Gene Transfer Events	2020		Vet Sci	7	3	amr--env--opinion--manure-as-a-potential-hotspot-for-antibiotic-resistance-dissemination-by-horizontal-gene-transfer-events	23/08/2020		
Journal Article	Development, implementation, and evaluation of a novel multidisciplinary one health course for university undergraduates	2020	100121	One Health	9		edu--eval--human--development,-implementation,-and-evaluation-of-a-novel-multidisciplinary-one-health-course-for-university-undergraduates	07/03/2020		
Journal Article	Defining Pollinator Health: A Holistic Approach Based on Ecological, Genetic, and Physiological Factors	2020	269-294	Annu Rev Anim Biosci	8		env--opinion--defining-pollinator-health:-a-holistic-approach-based-on-ecological,-genetic,-and-physiological-factors	17/10/2019		
Journal Article	Serosurvey of Treponema pallidum infection among children with skin ulcers in the Tarangire-Manyara ecosystem, northern Tanzania	2020	392	BMC Infect Dis	20	1	data--human--pathogen--serosurvey-of-treponema-pallidum-infection-among-children-with-skin-ulcers-in-the-tarangire-manyara-ecosystem,-northern-tanzania	05/06/2020		
Journal Article	A One Health Study of the Genetic Relatedness of Klebsiella pneumoniae and Their Mobile Elements in the East of England	2020	219-226	Clin Infect Dis	70	2	data--env--human--pathogen--a-one-health-study-of-the-genetic-relatedness-of-klebsiella-pneumoniae-and-their-mobile-elements-in-the-east-of-england	07/03/2019		
Journal Article	Farm dust resistomes and bacterial microbiomes in European poultry and pig farms	2020	10	Environment International	143		amr--data--domestic--env--farm-dust-resistomes-and-bacterial-microbiomes-in-european-poultry-and-pig-farms			
Journal Article	One Health in Practice: Using Integrated Bite Case Management to Increase Detection of Rabid Animals in Tanzania	2020	13	Front Public Health	8		edu--eval--human--pathogen--one-health-in-practice:-using-integrated-bite-case-management-to-increase-detection-of-rabid-animals-in-tanzania	03/03/2020		
Journal Article	Biosecurity: tools, behaviours and concepts	2020	449-452	Emerg Top Life Sci	4	5	human--opinion--policy--risk--biosecurity:-tools,-behaviours-and-concepts	15/12/2020		
Journal Article	One Health operational framework for action for the Eastern Mediterranean Region, focusing on zoonotic diseases	2020	720-725	East Mediterr Health J	26	6	policy--one-health-operational-framework-for-action-for-the-eastern-mediterranean-region,-focusing-on-zoonotic-diseases	06/07/2020		
Journal Article	Need for a sustainable use of medicinal products: environmental impacts of ivermectin	2020	492-496	Ann Ist Super Sanita	56	4	amr--env--risk--need-for-a-sustainable-use-of-medicinal-products:-environmental-impacts-of-ivermectin	22/12/2020		
Journal Article	Donkey Fascioliasis Within a One Health Control Action: Transmission Capacity, Field Epidemiology, and Reservoir Role in a Human Hyperendemic Area	2020	591384	Front Vet Sci	7		opinion--pathogen--wild--donkey-fascioliasis-within-a-one-health-control-action:-transmission-capacity,-field-epidemiology,-and-reservoir-role-in-a-human-hyperendemic-area	01/12/2020		
Journal Article	Human Respiratory Syncytial Virus Detected in Mountain Gorilla Respiratory Outbreaks	2020	449-460	Ecohealth	17	4	data--human--pathogen--wild--human-respiratory-syncytial-virus-detected-in-mountain-gorilla-respiratory-outbreaks	22/12/2020		
Journal Article	Disease risk analysis in sea turtles: A baseline study to inform conservation efforts	2020	e0230760	PLoS One	15	10	pathogen--risk--wild--disease-risk-analysis-in-sea-turtles:-a-baseline-study-to-inform-conservation-efforts	24/10/2020		
Journal Article	Rabies knowledge and practices among human and veterinary health workers in Chad	2020	105180	Acta Trop	202		edu--eval--pathogen--rabies-knowledge-and-practices-among-human-and-veterinary-health-workers-in-chad	23/09/2019		
Journal Article	Anthropogenic landscapes increase Campylobacter jejuni infections in urbanizing banded mongoose (Mungos mungo): A one health approach	2020	15	Plos Neglected Tropical Diseases	14	3	data--pathogen--wild--anthropogenic-landscapes-increase-campylobacter-jejuni-infections-in-urbanizing-banded-mongoose-(mungos-mungo):-a-one-health-approach			
Journal Article	Aspergillosis, Avian Species and the One Health Perspective: The Possible Importance of Birds in Azole Resistance	2020		Microorganisms	8	12	opinion--pathogen--wild--aspergillosis,-avian-species-and-the-one-health-perspective:-the-possible-importance-of-birds-in-azole-resistance	24/12/2020		
Journal Article	Antimicrobial use and production system shape the fecal, environmental, and slurry resistomes of pig farms	2020	164	Microbiome	8	1	amr--data--domestic--antimicrobial-use-and-production-system-shape-the-fecal,-environmental,-and-slurry-resistomes-of-pig-farms	21/11/2020		
Journal Article	Mites and ticks of reptiles and amphibians in Brazil	2020	105515	Acta Trop	208		data--pathogen--wild--mites-and-ticks-of-reptiles-and-amphibians-in-brazil	15/05/2020		
Journal Article	Key Limitations and New Insights Into the Toxoplasma gondii Parasite Stage Switching for Future Vaccine Development in Human, Livestock, and Cats	2020	22	Frontiers in Cellular and Infection Microbiology	10		opinion--pathogen--key-limitations-and-new-insights-into-the-toxoplasma-gondii-parasite-stage-switching-for-future-vaccine-development-in-human,-livestock,-and-cats			
Journal Article	Assessment of climate change impact on the malaria vector Anopheles hyrcanus, West Nile disease, and incidence of melanoma in the Vojvodina Province (Serbia) using data from a regional climate model	2020	e0227679	PLoS One	15	1	climate--data--human--pathogen--assessment-of-climate-change-impact-on-the-malaria-vector-anopheles-hyrcanus,-west-nile-disease,-and-incidence-of-melanoma-in-the-vojvodina-province-(serbia)-using-data-from-a-regional-climate-model	16/01/2020		
Journal Article	Merging Metagenomics and Spatial Epidemiology To Understand the Distribution of Antimicrobial Resistance Genes from Enterobacteriaceae in Wild Owls	2020	16	Applied and Environmental Microbiology	86	20	amr--data--wild--merging-metagenomics-and-spatial-epidemiology-to-understand-the-distribution-of-antimicrobial-resistance-genes-from-enterobacteriaceae-in-wild-owls			
Journal Article	An Ethnographic Approach to Characterizing Potential Pathways of Zoonotic Disease Transmission from Wild Meat in Guyana	2020	424-436	Ecohealth	17	4	eval--human--pathogen--an-ethnographic-approach-to-characterizing-potential-pathways-of-zoonotic-disease-transmission-from-wild-meat-in-guyana			
Journal Article	Implications of antibiotics use during the COVID-19 pandemic: present and future	2020	3413-3416	J Antimicrob Chemother	75	12	amr--covid--opinion--implications-of-antibiotics-use-during-the-covid-19-pandemic:-present-and-future	25/08/2020		
Journal Article	The challenges of investigating antimicrobial resistance in Vietnam - what benefits does a One Health approach offer the animal and human health sectors?	2020	213	BMC Public Health	20	1	amr--domestic--eval--human--the-challenges-of-investigating-antimicrobial-resistance-in-vietnam---what-benefits-does-a-one-health-approach-offer-the-animal-and-human-health-sectors?	13/02/2020		
Journal Article	Bovine tuberculosis at the human-livestock-wildlife interface and its control through one health approach in the Ethiopian Somali Pastoralists: A review	2020	5	One Health	9		opinion--pathogen--bovine-tuberculosis-at-the-human-livestock-wildlife-interface-and-its-control-through-one-health-approach-in-the-ethiopian-somali-pastoralists:-a-review			
Journal Article	Theoretical risk of genetic reassortment should not impede development of live, attenuated Rift Valley fever (RVF) vaccines commentary on the draft WHO RVF Target Product Profile	2020	100060	Vaccine X	5		pathogen--policy--theoretical-risk-of-genetic-reassortment-should-not-impede-development-of-live,-attenuated-rift-valley-fever-(rvf)-vaccines-commentary-on-the-draft-who-rvf-target-product-profile	28/04/2020		
Journal Article	High Genomic Diversity and Heterogenous Origins of Pathogenic and Antibiotic-Resistant Escherichia coli in Household Settings Represent a Challenge to Reducing Transmission in Low-Income Settings	2020		mSphere	5	1	amr--data--domestic--env--human--high-genomic-diversity-and-heterogenous-origins-of-pathogenic-and-antibiotic-resistant-escherichia-coli-in-household-settings-represent-a-challenge-to-reducing-transmission-in-low-income-settings	17/01/2020		
Journal Article	Reproduction of East-African bats may guide risk mitigation for coronavirus spillover	2020	2	One Health Outlook	2		covid--data--pathogen--wild--reproduction-of-east-african-bats-may-guide-risk-mitigation-for-coronavirus-spillover	07/02/2020		
Journal Article	A one health framework to estimate the cost of antimicrobial resistance	2020	14	Antimicrobial Resistance and Infection Control	9	1	eval--policy--a-one-health-framework-to-estimate-the-cost-of-antimicrobial-resistance			
Journal Article	A clinically important, plasmid-borne antibiotic resistance gene (beta-lactamase TEM-116) present in desert soils	2020	6	Science of the Total Environment	719		amr--data--env--a-clinically-important,-plasmid-borne-antibiotic-resistance-gene-(beta-lactamase-tem-116)-present-in-desert-soils			
Journal Article	Quantitatively evaluating the cross-sectoral and One Health impact of interventions: A scoping review and case study of antimicrobial resistance	2020	10	One Health	11		data--eval--policy--quantitatively-evaluating-the-cross-sectoral-and-one-health-impact-of-interventions:-a-scoping-review-and-case-study-of-antimicrobial-resistance			
Journal Article	Antimicrobial drug resistant non-typhoidal Salmonella enterica in commercial poultry value chain in Chitwan, Nepal	2020	18	One Health Outlook	2		amr--data--domestic--env--eval--human--antimicrobial-drug-resistant-non-typhoidal-salmonella-enterica-in-commercial-poultry-value-chain-in-chitwan,-nepal	09/04/2021		
Journal Article	Detecting national human enteric disease outbreaks linked to animal contact in the United States of America	2020	471-480	Revue Scientifique Et Technique-Office International Des Epizooties	39	2	domestic--opinion--pathogen--detecting-national-human-enteric-disease-outbreaks-linked-to-animal-contact-in-the-united-states-of-america			
Journal Article	One Health: How Interdependence Enriches Veterinary Ethics Education	2020	11	Animals	10	1	edu--human--opinion--one-health:-how-interdependence-enriches-veterinary-ethics-education			
Journal Article	Molecular characterization ofBrucellaspecies detected in humans and domestic ruminants of pastoral areas in Kagera ecosystem, Tanzania	2020	711-719	Veterinary Medicine and Science	6	4	data--domestic--pathogen--molecular-characterization-ofbrucellaspecies-detected-in-humans-and-domestic-ruminants-of-pastoral-areas-in-kagera-ecosystem,-tanzania			
Journal Article	National action to combat AMR: a One-Health approach to assess policy priorities in action plans	2020	10	Bmj Global Health	5	7	amr--policy--national-action-to-combat-amr:-a-one-health-approach-to-assess-policy-priorities-in-action-plans			
Journal Article	Profiling the public health risk of canine rabies transmission in Kogi state, Nigeria	2020	100154	One Health	10		human--pathogen--risk--profiling-the-public-health-risk-of-canine-rabies-transmission-in-kogi-state,-nigeria	30/10/2020		
Journal Article	Genomic and Resistance Epidemiology of Gram-Negative Bacteria in Africa: a Systematic Review and Phylogenomic Analyses from a One Health Perspective	2020		mSystems	5	6	amr--data--pathogen--genomic-and-resistance-epidemiology-of-gram-negative-bacteria-in-africa:-a-systematic-review-and-phylogenomic-analyses-from-a-one-health-perspective	26/11/2020		
Journal Article	A One Health Perspective on the Human-Companion Animal Relationship with Emphasis on Zoonotic Aspects	2020	29	International Journal of Environmental Research and Public Health	17	11	domestic--opinion--pathogen--a-one-health-perspective-on-the-human-companion-animal-relationship-with-emphasis-on-zoonotic-aspects			
Journal Article	Antimicrobial Resistance in Veterinary Medicine: An Overview	2020		Int J Mol Sci	21	6	amr--opinion--antimicrobial-resistance-in-veterinary-medicine:-an-overview	15/03/2020		
Journal Article	Understanding Antibiotic Residues and Pathogens Flow in Wastewater from Smallholder Pig Farms to Agriculture Field in Ha Nam Province, Vietnam	2020	1178630220943206	Environ Health Insights	14		amr--data--domestic--env--human--understanding-antibiotic-residues-and-pathogens-flow-in-wastewater-from-smallholder-pig-farms-to-agriculture-field-in-ha-nam-province,-vietnam	23/10/2020		
Journal Article	Country Income Is Only One of the Tiles: The Global Journey of Antimicrobial Resistance among Humans, Animals, and Environment	2020		Antibiotics (Basel)	9	8	amr--opinion--country-income-is-only-one-of-the-tiles:-the-global-journey-of-antimicrobial-resistance-among-humans,-animals,-and-environment	06/08/2020		
Journal Article	Biosecurity measures in European beekeeping	2020	725-735	Revue Scientifique Et Technique-Office International Des Epizooties	39	3	opinion--pathogen--wild--biosecurity-measures-in-european-beekeeping			
Journal Article	Antimicrobial use in food animals and human health: time to implement 'One Health' approach	2020	181	Antimicrob Resist Infect Control	9	1	amr--opinion--antimicrobial-use-in-food-animals-and-human-health:-time-to-implement-'one-health'-approach	09/11/2020		
Journal Article	Predicting disease risk areas through co-production of spatial models: The example of Kyasanur Forest Disease in India's forest landscapes	2020	27	Plos Neglected Tropical Diseases	14	4	pathogen--risk--predicting-disease-risk-areas-through-co-production-of-spatial-models:-the-example-of-kyasanur-forest-disease-in-india's-forest-landscapes			
Journal Article	Inadequate socialisation, inactivity, and urban living environment are associated with social fearfulness in pet dogs	2020	10	Scientific Reports	10	1	data--domestic--non-bio--inadequate-socialisation,-inactivity,-and-urban-living-environment-are-associated-with-social-fearfulness-in-pet-dogs			
Journal Article	The economics of antimicrobial resistance in veterinary medicine: Optimizing societal benefits through mesoeconomic approaches from public and private perspectives	2020	10	One Health	10		amr--opinion--the-economics-of-antimicrobial-resistance-in-veterinary-medicine:-optimizing-societal-benefits-through-mesoeconomic-approaches-from-public-and-private-perspectives			
Journal Article	Leptospira Infection in African Green Monkeys in an Endemic Area: An Opportunity for Comparative Studies in a Natural Environment	2020	9	Pathogens	9	6	data--pathogen--wild--leptospira-infection-in-african-green-monkeys-in-an-endemic-area:-an-opportunity-for-comparative-studies-in-a-natural-environment			
Journal Article	Estimates of the global and continental burden of animal tuberculosis in key livestock species worldwide: A meta-analysis study	2020	100169	One Health	10		data--domestic--pathogen--estimates-of-the-global-and-continental-burden-of-animal-tuberculosis-in-key-livestock-species-worldwide:-a-meta-analysis-study	03/11/2020		
Journal Article	Movement patterns of free-roaming dogs on heterogeneous urban landscapes: Implications for rabies control	2020	104978	Prev Vet Med	178		data--domestic--pathogen--movement-patterns-of-free-roaming-dogs-on-heterogeneous-urban-landscapes:-implications-for-rabies-control	18/04/2020		
Journal Article	Microbial assisted phytodepuration for water reclamation: Environmental benefits and threats	2020	11	Chemosphere	241		amr--env--eval--opinion--microbial-assisted-phytodepuration-for-water-reclamation:-environmental-benefits-and-threats			
Journal Article	Surveillance for Harmful Algal Bloom Events and Associated Human and Animal Illnesses - One Health Harmful Algal Bloom System, United States, 2016-2018	2020	1889-1894	MMWR Morb Mortal Wkly Rep	69	50	data--env--human--pathogen--surveillance-for-harmful-algal-bloom-events-and-associated-human-and-animal-illnesses---one-health-harmful-algal-bloom-system,-united-states,-2016-2018	18/12/2020		
Journal Article	Bioaerosol sampling optimization for community exposure assessment in cities with poor sanitation: A one health cross-sectional study	2020	7	Science of the Total Environment	738		data--env--human--pathogen--bioaerosol-sampling-optimization-for-community-exposure-assessment-in-cities-with-poor-sanitation:-a-one-health-cross-sectional-study			
Journal Article	Multidrug-Resistant CTX-M and CMY-2 ProducingEscherichia coliIsolated from Healthy Household Dogs from the Great Metropolitan Area, Costa Rica	2020	1421-1428	Microbial Drug Resistance	26	11	amr--data--domestic--multidrug-resistant-ctx-m-and-cmy-2-producingescherichia-coliisolated-from-healthy-household-dogs-from-the-great-metropolitan-area,-costa-rica			
Journal Article	A One Health Perspective to Recognize Fusarium as Important in Clinical Practice	2020	13	Journal of Fungi	6	4	opinion--pathogen--a-one-health-perspective-to-recognize-fusarium-as-important-in-clinical-practice			
Journal Article	Escherichia coli, Salmonella spp., and Staphylococcus aureus susceptibility to antimicrobials of human and veterinary importance in poultry sector of India	2020	11	Journal of Food Safety	40	1	amr--data--domestic--escherichia-coli,-salmonella-spp.,-and-staphylococcus-aureus-susceptibility-to-antimicrobials-of-human-and-veterinary-importance-in-poultry-sector-of-india			
Journal Article	Prevalence and antimicrobial resistance of MRSA across different pig age groups in an intensive pig production system in Australia	2020	576-586	Zoonoses Public Health	67	5	amr--data--domestic--pathogen--prevalence-and-antimicrobial-resistance-of-mrsa-across-different-pig-age-groups-in-an-intensive-pig-production-system-in-australia	28/05/2020		
Journal Article	The Landscape of Anthrax Prevention and Control: Stakeholders' Perceptive in Odisha, India	2020	14	International Journal of Environmental Research and Public Health	17	9	eval--human--pathogen--policy--the-landscape-of-anthrax-prevention-and-control:-stakeholders'-perceptive-in-odisha,-india			
Journal Article	Bacterial Diversity and Antibiotic Susceptibility of Sparus aurata from Aquaculture	2020		Microorganisms	8	9	amr--data--env--pathogen--wild--bacterial-diversity-and-antibiotic-susceptibility-of-sparus-aurata-from-aquaculture	06/09/2020		
Journal Article	Updated research agenda for water, sanitation and antimicrobial resistance	2020	858-866	J Water Health	18	6	amr--env--eval--opinion--updated-research-agenda-for-water,-sanitation-and-antimicrobial-resistance	18/12/2020		
Journal Article	COVID-19: An Appeal for an Intersectoral Approach to Tackle With the Emergency	2020	302	Front Public Health	8		covid--opinion--covid-19:-an-appeal-for-an-intersectoral-approach-to-tackle-with-the-emergency	03/07/2020		
Journal Article	One Health in the context of coronavirus outbreaks: A systematic literature review	2020	9	One Health	10		covid--eval--data--pathogen--one-health-in-the-context-of-coronavirus-outbreaks:-a-systematic-literature-review			
Journal Article	Dynamics of mcr-1 prevalence and mcr-1-positive Escherichia coli after the cessation of colistin use as a feed additive for animals in China: a prospective cross-sectional and whole genome sequencing-based molecular epidemiological study	2020	E34-E43	Lancet Microbe	1	1	amr--data--domestic--dynamics-of-mcr-1-prevalence-and-mcr-1-positive-escherichia-coli-after-the-cessation-of-colistin-use-as-a-feed-additive-for-animals-in-china:-a-prospective-cross-sectional-and-whole-genome-sequencing-based-molecular-epidemiological-study			
Journal Article	High Prevalence of Clostridium difficile in Home Gardens in Western Australia	2020	9	Applied and Environmental Microbiology	87	1	data--env--pathogen--high-prevalence-of-clostridium-difficile-in-home-gardens-in-western-australia			
Journal Article	Irrigation System and COVID-19 Recurrence: A Potential Risk Factor in the Transmission of SARS-CoV-2	2020	2903-2905	Acs Chemical Neuroscience	11	19	covid--env--opinion--irrigation-system-and-covid-19-recurrence:-a-potential-risk-factor-in-the-transmission-of-sars-cov-2			
Journal Article	An ethical investigation into the microbiome: the intersection of agriculture, genetics, and the obesity epidemic	2020	13	Gut Microbes	12	1	non-bio--obesity--opinion--an-ethical-investigation-into-the-microbiome:-the-intersection-of-agriculture,-genetics,-and-the-obesity-epidemic			
Journal Article	Extended-spectrum β-lactamase-producing Escherichia coli isolated from raw vegetables in South Korea	2020	19721	Sci Rep	10	1	amr--data--domestic--extended-spectrum-β-lactamase-producing-escherichia-coli-isolated-from-raw-vegetables-in-south-korea	14/11/2020		
Journal Article	Sustainable aquaculture through the One Health lens	2020	468-474	Nat Food	1	8	opinion--policy--wild--sustainable-aquaculture-through-the-one-health-lens	01/08/2020		
Journal Article	Global prevalence of Cryptosporidium infection in rodents: A systematic review and meta-analysis	2020	9	Preventive Veterinary Medicine	182		data--pathogen--wild--global-prevalence-of-cryptosporidium-infection-in-rodents:-a-systematic-review-and-meta-analysis			
Journal Article	Human Relationships with Domestic and Other Animals: One Health, One Welfare, One Biology	2020	21	Animals	10	1	opinion--human-relationships-with-domestic-and-other-animals:-one-health,-one-welfare,-one-biology			
Journal Article	Transmission of severe acute respiratory syndrome coronavirus 2 (SARS-CoV-2) to animals: an updated review	2020	11	Journal of Translational Medicine	18	1	covid--domestic--opinion--wild--transmission-of-severe-acute-respiratory-syndrome-coronavirus-2-(sars-cov-2)-to-animals:-an-updated-review			
Journal Article	Absence of SARS-CoV-2 infection in cats and dogs in close contact with a cluster of COVID-19 patients in a veterinary campus	2020	100164	One Health	10		covid--data--domestic--absence-of-sars-cov-2-infection-in-cats-and-dogs-in-close-contact-with-a-cluster-of-covid-19-patients-in-a-veterinary-campus	10/09/2020		
Journal Article	Factors associated with lead (Pb) exposure on dogs around a Pb mining area, Kabwe, Zambia	2020	9	Chemosphere	247		data--domestic--non-bio--factors-associated-with-lead-(pb)-exposure-on-dogs-around-a-pb-mining-area,-kabwe,-zambia			
Journal Article	Investigation of Ctenocephalides felis on domestic dogs and Rickettsia felis infection in the Democratic Republic of Sao Tome and Principe	2020	892-902	Zoonoses Public Health	67	8	data--domestic--pathogen--investigation-of-ctenocephalides-felis-on-domestic-dogs-and-rickettsia-felis-infection-in-the-democratic-republic-of-sao-tome-and-principe	05/11/2020		
Journal Article	Driving Adoption and Commercialization of Subunit Vaccines for Bovine Tuberculosis and Johne's Disease: Policy Choices and Implications for Food Security	2020	18	Vaccines	8	4	domestic--opinion--pathogen--driving-adoption-and-commercialization-of-subunit-vaccines-for-bovine-tuberculosis-and-johne's-disease:-policy-choices-and-implications-for-food-security			
Journal Article	No evidence for sylvatic cycles of chikungunya, dengue and Zika viruses in African green monkeys (Chlorocebus aethiops sabaeus) on St. Kitts, West Indies	2020	540	Parasit Vectors	13	1	data--pathogen--wild--no-evidence-for-sylvatic-cycles-of-chikungunya,-dengue-and-zika-viruses-in-african-green-monkeys-(chlorocebus-aethiops-sabaeus)-on-st.-kitts,-west-indies	01/11/2020		
Journal Article	Ethical Decision-Making in Zoonotic Disease Control How Do One Health Strategies Function in the Netherlands?	2020	239-259	Journal of Agricultural & Environmental Ethics	33	2	eval--human--pathogen--ethical-decision-making-in-zoonotic-disease-control-how-do-one-health-strategies-function-in-the-netherlands?			
Journal Article	The one health problem of azole resistance in Aspergillus fumigatus: current insights and future research agenda	2020	202-214	Fungal Biology Reviews	34	4	amr--eval--human--opinion--the-one-health-problem-of-azole-resistance-in-aspergillus-fumigatus:-current-insights-and-future-research-agenda			
Journal Article	In-Depth Genomic Characterization of a Meropenem-nonsusceptible Pseudomonas otitidis Strain Contaminating Chicken Carcass	2020	6	Acta Scientiae Veterinariae	48		amr--data--domestic--in-depth-genomic-characterization-of-a-meropenem-nonsusceptible-pseudomonas-otitidis-strain-contaminating-chicken-carcass			
Journal Article	Climate change and other risk drivers of animal health and zoonotic disease emergencies: the need for a multidisciplinary and multisectoral approach to disaster risk management	2020	461-470	Revue Scientifique Et Technique-Office International Des Epizooties	39	2	opinion--climate-change-and-other-risk-drivers-of-animal-health-and-zoonotic-disease-emergencies:-the-need-for-a-multidisciplinary-and-multisectoral-approach-to-disaster-risk-management			
Journal Article	COVID-19 in India: Making a Case for the One Health Surveillance System	2020	135-138	Indian Journal of Public Health	64		covid--opinion--covid-19-in-india:-making-a-case-for-the-one-health-surveillance-system			
Journal Article	Wild Micromammals as Bioindicators of Antibiotic Resistance in Ecopathology in Northern Italy	2020	10	Animals	10	7	amr--data--wild--wild-micromammals-as-bioindicators-of-antibiotic-resistance-in-ecopathology-in-northern-italy			
Journal Article	Towards integrated surveillance-response systems for the prevention of future pandemics	2020	6	Infectious Diseases of Poverty	9	1	opinion--pathogen--towards-integrated-surveillance-response-systems-for-the-prevention-of-future-pandemics			
Journal Article	The One Health European Joint Programme (OHEJP), 2018–2022: an exemplary One Health initiative	2020	1037-1039	Journal of Medical Microbiology	69	8	the-one-health-european-joint-programme-(ohejp),-2018–2022:-an-exemplary-one-health-initiative		7	Microbiology Society
Journal Article	Evaluating the Integration of One Health in Surveillance Systems for Antimicrobial Use and Resistance: A Conceptual Framework	2021	611931	Front Vet Sci	8		amr--eval--evaluating-the-integration-of-one-health-in-surveillance-systems-for-antimicrobial-use-and-resistance:-a-conceptual-framework	13/04/2021		
Journal Article	Raw meat based diet (RMBD) for household pets as potential door opener to parasitic load of domestic and urban environment. Revival of understated zoonotic hazards? A review	2021	100327	One Health	13		domestic--opinion--pathogen--raw-meat-based-diet-(rmbd)-for-household-pets-as-potential-door-opener-to-parasitic-load-of-domestic-and-urban-environment.-revival-of-understated-zoonotic-hazards?-a-review	30/09/2021		
Journal Article	Molecular epidemiology of Brucella species in mixed livestock-human ecosystems in Kenya	2021	8881	Sci Rep	11	1	data--domestic--human--pathogen--molecular-epidemiology-of-brucella-species-in-mixed-livestock-human-ecosystems-in-kenya	25/04/2021		
Journal Article	The potential of using E. coli as an indicator for the surveillance of antimicrobial resistance (AMR) in the environment	2021	152-158	Current Opinion in Microbiology	64		amr--env--opinion--the-potential-of-using-e.-coli-as-an-indicator-for-the-surveillance-of-antimicrobial-resistance-(amr)-in-the-environment			
Journal Article	Intersectoral collaboration shaping One Health in the policy agenda: A comparative analysis of Ghana and India	2021	9	One Health	13		policy--intersectoral-collaboration-shaping-one-health-in-the-policy-agenda:-a-comparative-analysis-of-ghana-and-india			
Journal Article	A serological survey of brucellosis in wildlife in four major National Parks of Uganda	2021	95	BMC Vet Res	17	1	data--pathogen--wild--a-serological-survey-of-brucellosis-in-wildlife-in-four-major-national-parks-of-uganda	03/03/2021		
Journal Article	Operationalising the "One Health" approach in India: facilitators of and barriers to effective cross-sector convergence for zoonoses prevention and control	2021	21	BMC Public Health	21	1	eval--policy--operationalising-the-"one-health"-approach-in-india:-facilitators-of-and-barriers-to-effective-cross-sector-convergence-for-zoonoses-prevention-and-control			
Journal Article	Antibiotic Resistance: One Health One World Outlook	2021	771510	Front Cell Infect Microbiol	11		amr--opinion--antibiotic-resistance:-one-health-one-world-outlook	14/12/2021		
Journal Article	Genetic relatedness of multidrug resistant Escherichia coli isolated from humans, chickens and poultry environments	2021	13	Antimicrobial Resistance and Infection Control	10	1	amr--data--domestic--human--genetic-relatedness-of-multidrug-resistant-escherichia-coli-isolated-from-humans,-chickens-and-poultry-environments			
Journal Article	COVID-19: an opportunity to re-evaluate the implementation of a One Health approach to tackling emerging infections in Nigeria and other sub-Saharan African countries	2021	6	Journal of the Egyptian Public Health Association	96	1	covid--opinion--covid-19:-an-opportunity-to-re-evaluate-the-implementation-of-a-one-health-approach-to-tackling-emerging-infections-in-nigeria-and-other-sub-saharan-african-countries			
Journal Article	Circulation of Babesia Species and Their Exposure to Humans through Ixodes Ricinus	2021		Pathogens	10	4	data--env--pathogen--circulation-of-babesia-species-and-their-exposure-to-humans-through-ixodes-ricinus	04/04/2021		
Journal Article	One Health Perspectives on New Emerging Viral Diseases in African Wild Great Apes	2021		Pathogens	10	10	human--opinion--wild--one-health-perspectives-on-new-emerging-viral-diseases-in-african-wild-great-apes	24/10/2021		
Journal Article	A One Health perspective on the issue of the antibiotic resistance	2021	14	Parasite	28		opinion--a-one-health-perspective-on-the-issue-of-the-antibiotic-resistance			
Journal Article	From Modern Planetary Health to Decolonial Promotion of One Health of Peripheries	2021	637897	Front Public Health	9		opinion--from-modern-planetary-health-to-decolonial-promotion-of-one-health-of-peripheries	29/06/2021		
Journal Article	One Health initiative in the Bolivian Altiplano human fascioliasis hyperendemic area: Lymnaeid biology, population dynamics, microecology and climatic factor influences	2021	24	Revista Brasileira De Parasitologia Veterinaria	30	2	opinion--pathogen--one-health-initiative-in-the-bolivian-altiplano-human-fascioliasis-hyperendemic-area:-lymnaeid-biology,-population-dynamics,-microecology-and-climatic-factor-influences			
Journal Article	Zoonotic enteric parasites in Mongolian people, animals, and the environment: Using One Health to address shared pathogens	2021	24	Plos Neglected Tropical Diseases	15	7	data--domestic--env--human--pathogen--wild--zoonotic-enteric-parasites-in-mongolian-people,-animals,-and-the-environment:-using-one-health-to-address-shared-pathogens			
Journal Article	Antimicrobial resistance in wildlife and in the built environment in a wildlife rehabilitation center	2021	100298	One Health	13		amr--data--wild--antimicrobial-resistance-in-wildlife-and-in-the-built-environment-in-a-wildlife-rehabilitation-center	18/08/2021		
Journal Article	Increasing awareness for tick-borne encephalitis virus using small ruminants as suitable sentinels: Preliminary observations	2021	6	One Health	12		data--domestic--pathogen--increasing-awareness-for-tick-borne-encephalitis-virus-using-small-ruminants-as-suitable-sentinels:-preliminary-observations			
Journal Article	A Survey on One Health Approach in Colombia and Some Latin American Countries: From a Fragmented Health Organization to an Integrated Health Response to Global Challenges	2021	18	Frontiers in Public Health	9		eval--policy--a-survey-on-one-health-approach-in-colombia-and-some-latin-american-countries:-from-a-fragmented-health-organization-to-an-integrated-health-response-to-global-challenges			
Journal Article	The Power of Music to Prevent and Control Emerging Infectious Diseases	2021	756152	Front Med (Lausanne)	8		edu--human--opinion--policy--the-power-of-music-to-prevent-and-control-emerging-infectious-diseases	14/12/2021		
Journal Article	One Digital Health Is FAIR	2021	57-58	Stud Health Technol Inform	287		opinion--policy--one-digital-health-is-fair	20/11/2021		
Journal Article	One Digital Health: A Unified Framework for Future Health Ecosystems	2021	e22189	J Med Internet Res	23	2	opinion--one-digital-health:-a-unified-framework-for-future-health-ecosystems	26/01/2021		
Journal Article	Evaluating Integrated Surveillance for Antimicrobial Use and Resistance in England: A Qualitative Study	2021	16	Frontiers in Veterinary Science	8		amr--eval--opinion--evaluating-integrated-surveillance-for-antimicrobial-use-and-resistance-in-england:-a-qualitative-study			
Journal Article	Didelphis spp. opossums and their parasites in the Americas: A One Health perspective	2021	4091-4111	Parasitology Research	120	12	opinion--pathogen--wild--didelphis-spp.-opossums-and-their-parasites-in-the-americas:-a-one-health-perspective			
Journal Article	Kyasanur Forest Disease, is our surveillance system healthy to prevent a larger outbreak? A mixed-method study, Shivamogga, Karnataka, India: 2019	2021	S50-S61	International Journal of Infectious Diseases	110		eval--human--pathogen--risk--kyasanur-forest-disease,-is-our-surveillance-system-healthy-to-prevent-a-larger-outbreak?-a-mixed-method-study,-shivamogga,-karnataka,-india:-2019			
Journal Article	Multisectoral cost analysis of a human and livestock anthrax outbreak in Songwe Region, Tanzania (December 2018-January 2019), using a novel Outbreak Costing Tool	2021	7	One Health	13		pathogen--risk--multisectoral-cost-analysis-of-a-human-and-livestock-anthrax-outbreak-in-songwe-region,-tanzania-(december-2018-january-2019),-using-a-novel-outbreak-costing-tool			
Journal Article	One Health drivers of antibacterial resistance: Quantifying the relative impacts of human, animal and environmental use and transmission	2021	8	One Health	12		amr--risk--one-health-drivers-of-antibacterial-resistance:-quantifying-the-relative-impacts-of-human,-animal-and-environmental-use-and-transmission			
Journal Article	Engaging Stakeholders in the Design of One Health Surveillance Systems: A Participatory Approach	2021	646458	Front Vet Sci	8		policy--engaging-stakeholders-in-the-design-of-one-health-surveillance-systems:-a-participatory-approach	11/06/2021		
Journal Article	Spillover, hybridization, and persistence in schistosome transmission dynamics at the human-animal interface	2021	11	Proceedings of the National Academy of Sciences of the United States of America	118	41	domestic--pathogen--risk--spillover,-hybridization,-and-persistence-in-schistosome-transmission-dynamics-at-the-human-animal-interface			
Journal Article	Host and geographic differences in prevalence and diversity of gastrointestinal helminths of foxes (Vulpes vulpes), coyotes (Canis latrans) and wolves (Canis lupus) in Québec, Canada	2021	126-137	Int J Parasitol Parasites Wildl	16		data--pathogen--wild--host-and-geographic-differences-in-prevalence-and-diversity-of-gastrointestinal-helminths-of-foxes-(vulpes-vulpes),-coyotes-(canis-latrans)-and-wolves-(canis-lupus)-in-québec,-canada	24/09/2021		
Journal Article	Identifying the research gap of zoonotic disease in displacement: a systematic review	2021	25	Glob Health Res Policy	6	1	data--human--pathogen--identifying-the-research-gap-of-zoonotic-disease-in-displacement:-a-systematic-review	18/07/2021		
Journal Article	Social Sciences in One Health: Insights From Multiple Worlds Perspectives on the Dam Rupture in Brumadinho-Brazil	2021	8	Frontiers in Public Health	9		opinion--social-sciences-in-one-health:-insights-from-multiple-worlds-perspectives-on-the-dam-rupture-in-brumadinho-brazil			
Journal Article	Transmission Dynamics of Shiga Toxin-Producing Escherichia coli in New Zealand Cattle from Farm to Slaughter	2021		Appl Environ Microbiol	87	11	data--pathogen--transmission-dynamics-of-shiga-toxin-producing-escherichia-coli-in-new-zealand-cattle-from-farm-to-slaughter	28/03/2021		
Journal Article	A one health glossary to support communication and information exchange between the human health, animal health and food safety sectors	2021	7	One Health	13		opinion--policy--a-one-health-glossary-to-support-communication-and-information-exchange-between-the-human-health,-animal-health-and-food-safety-sectors			
Journal Article	A proposal to leverage high-quality veterinary diagnostic laboratory large data streams for animal health, public health, and One Health	2021	399-409	J Vet Diagn Invest	33	3	opinion--policy--a-proposal-to-leverage-high-quality-veterinary-diagnostic-laboratory-large-data-streams-for-animal-health,-public-health,-and-one-health	27/03/2021		
Journal Article	One hundred years of zoonoses research in the Horn of Africa: A scoping review	2021	e0009607	PLoS Negl Trop Dis	15	7	opinion--pathogen--one-hundred-years-of-zoonoses-research-in-the-horn-of-africa:-a-scoping-review	17/07/2021		
Journal Article	Identification and Characterization of Campylobacter Species in Livestock, Humans, and Water in Livestock Owning Households of Peri-urban Addis Ababa, Ethiopia: A One Health Approach	2021	750551	Front Public Health	9		data--domestic--env--human--pathogen--wild--identification-and-characterization-of-campylobacter-species-in-livestock,-humans,-and-water-in-livestock-owning-households-of-peri-urban-addis-ababa,-ethiopia:-a-one-health-approach	21/12/2021		
Journal Article	Antimicrobial use and resistance data in human and animal sectors in the Lao PDR: evidence to inform policy	2021	12	Bmj Global Health	6	12	amr--data--antimicrobial-use-and-resistance-data-in-human-and-animal-sectors-in-the-lao-pdr:-evidence-to-inform-policy			
Journal Article	One Health, "Disease X" & the challenge of "Unknown" Unknowns	2021	264-271	Indian J Med Res	153	3	policy--one-health,-"disease-x"-&-the-challenge-of-"unknown"-unknowns	29/04/2021		
Journal Article	A Survey on One Health Perception and Experiences in Europe and Neighboring Areas	2021	15	Frontiers in Public Health	9		eval--human--a-survey-on-one-health-perception-and-experiences-in-europe-and-neighboring-areas			
Journal Article	Effects of Human Settlements and Spatial Distribution of Wing Vein Length, Wing Fray Categories and Hunger Stages in Glossina morsitans morsitans (Diptera: Glossinidae) and Glossina pallidipes (Diptera: Glossinidae) in Areas Devoid of Cattle in North-Eastern Zambia	2021	891-899	J Med Entomol	58	2	data--human--pathogen--wild--effects-of-human-settlements-and-spatial-distribution-of-wing-vein-length,-wing-fray-categories-and-hunger-stages-in-glossina-morsitans-morsitans-(diptera:-glossinidae)-and-glossina-pallidipes-(diptera:-glossinidae)-in-areas-devoid-of-cattle-in-north-eastern-zambia	30/10/2020		
Journal Article	Major zoonotic diseases of public health importance in Bangladesh	2021	1199-1210	Vet Med Sci	7	4	opinion--pathogen--major-zoonotic-diseases-of-public-health-importance-in-bangladesh	03/03/2021		
Journal Article	An analysis of national action plans on antimicrobial resistance in Southeast Asia using a governance framework approach	2021	100084	Lancet Reg Health West Pac	7		amr--policy--an-analysis-of-national-action-plans-on-antimicrobial-resistance-in-southeast-asia-using-a-governance-framework-approach	31/07/2021		
Journal Article	Cross-sectional study on intestinal parasite infections in different ecological zones of the Department of La Paz, Bolivia	2021	6	One Health	13		data--human--pathogen--cross-sectional-study-on-intestinal-parasite-infections-in-different-ecological-zones-of-the-department-of-la-paz,-bolivia			
Journal Article	Biodiversity and Human Health Interlinkages in Higher Education Offerings: A First Global Overview	2021	637901	Front Public Health	9		edu--biodiversity-and-human-health-interlinkages-in-higher-education-offerings:-a-first-global-overview	16/03/2021		
Journal Article	Improving the knowledge of high school students about zoonotic diseases from pets in Medellin-Colombia	2021	3091-3098	Veterinary World	14	12	eval--human--pathogen--improving-the-knowledge-of-high-school-students-about-zoonotic-diseases-from-pets-in-medellin-colombia			

Journal Article	Risk of SARS-CoV-2 transmission from humans to bats - An Australian assessment	2021	100247	One Health	13		covid--domestic--human--risk--wild--risk-of-sars-cov-2-transmission-from-humans-to-bats---an-australian-assessment	11/05/2021
Journal Article	First Global Report of Plasmid-Mediated mcr-1 and Extended-Spectrum Beta-Lactamase-Producing Escherichia coli from Sheep in Portugal	2021		Antibiotics (Basel)	10	11	data--domestic--pathogen--first-global-report-of-plasmid-mediated-mcr-1-and-extended-spectrum-beta-lactamase-producing-escherichia-coli-from-sheep-in-portugal	28/11/2021
Journal Article	"He Who Relies on His Brother's Property Dies Poor": The Complex Narratives of Livestock Care in Northern Tanzania	2021	18	Frontiers in Veterinary Science	8		human--pathogen--risk--"he-who-relies-on-his-brother's-property-dies-poor":-the-complex-narratives-of-livestock-care-in-northern-tanzania	
Journal Article	Detecting genes associated with antimicrobial resistance and pathogen virulence in three New Zealand rivers	2021	15	PeerJ	9		amr--data--env--detecting-genes-associated-with-antimicrobial-resistance-and-pathogen-virulence-in-three-new-zealand-rivers	
Journal Article	"Health in" and "Health of" Social-Ecological Systems: A Practical Framework for the Management of Healthy and Resilient Agricultural and Natural Ecosystems	2021	15	Frontiers in Public Health	8		opinion--policy--"health-in"-and-"health-of"-social-ecological-systems:-a-practical-framework-for-the-management-of-healthy-and-resilient-agricultural-and-natural-ecosystems	
Journal Article	Operationalisation of consensual One Health roadmaps in countries for improved IHR capacities and health security	2021		BMJ Glob Health	6	7	eval--human--policy--operationalisation-of-consensual-one-health-roadmaps-in-countries-for-improved-ihr-capacities-and-health-security	03/07/2021
Journal Article	Reduction in antimicrobial resistance prevalence in Escherichia coli from a pig farm following withdrawal of group antimicrobial treatment	2021	8	Veterinary Microbiology	258		amr--data--domestic--reduction-in-antimicrobial-resistance-prevalence-in-escherichia-coli-from-a-pig-farm-following-withdrawal-of-group-antimicrobial-treatment	
Journal Article	Representations of Free-Living and Unrestrained Dogs as an Emerging Public Health Issue in Australian Newspapers	2021		Int J Environ Res Public Health	18	11	domestic--eval--human--opinion--pathogen--policy--representations-of-free-living-and-unrestrained-dogs-as-an-emerging-public-health-issue-in-australian-newspapers	03/06/2021
Journal Article	The Zoonotic Parasite Dirofilaria repens Emerged in the Baltic Countries Estonia, Latvia, and Lithuania in 2008-2012 and Became Established and Endemic in a Decade	2021	1-5	Vector Borne Zoonotic Dis	21	1	opinion--pathogen--wild--the-zoonotic-parasite-dirofilaria-repens-emerged-in-the-baltic-countries-estonia,-latvia,-and-lithuania-in-2008-2012-and-became-established-and-endemic-in-a-decade	29/09/2020
Journal Article	Assessing the risks of SARS-CoV-2 in wildlife	2021	7	One Health Outlook	3		opinion--policy--wild--assessing-the-risks-of-sars-cov-2-in-wildlife	10/04/2021
Journal Article	Systems-thinking approach to identify and assess feasibility of potential interventions to reduce antibiotic use in tilapia farming in Egypt	2021	736735	Aquaculture	540		amr--policy--risk--systems-thinking-approach-to-identify-and-assess-feasibility-of-potential-interventions-to-reduce-antibiotic-use-in-tilapia-farming-in-egypt	20/07/2021
Journal Article	Linking the Mycobacterium ulcerans environment to Buruli ulcer disease: Progress and challenges	2021	100311	One Health	13		human--opinion--pathogen--policy--wild--linking-the-mycobacterium-ulcerans-environment-to-buruli-ulcer-disease:-progress-and-challenges	07/09/2021
Journal Article	Survey of One Health programs in U.S. medical schools and development of a novel one health elective for medical students	2021	100231	One Health	12		edu--eval--human--survey-of-one-health-programs-in-u.s.-medical-schools-and-development-of-a-novel-one-health-elective-for-medical-students	18/03/2021
Journal Article	Environmental contamination by pet pharmaceuticals: A hidden problem	2021	147827	Sci Total Environ	788		amr--domestic--opinion--pathogen--environmental-contamination-by-pet-pharmaceuticals:-a-hidden-problem	18/06/2021
Journal Article	On the need for integrating cancer into the One Health perspective	2021	2571-2575	Evolutionary Applications	14	11	cancer--domestic--human--opinion--wild--on-the-need-for-integrating-cancer-into-the-one-health-perspective	
Journal Article	A Call for One Health in Medical Education: How the COVID-19 Pandemic Underscores the Need to Integrate Human, Animal, and Environmental Health	2021	951-953	Acad Med	96	7	edu--opinion--pathogen--a-call-for-one-health-in-medical-education:-how-the-covid-19-pandemic-underscores-the-need-to-integrate-human,-animal,-and-environmental-health	27/03/2021
Journal Article	Prevalence and phenotypic characterization of Salmonella enterica isolates from three species of wild marine turtles in Grenada, West Indies	2021	222-229	Vet World	14	1	data--pathogen--wild--prevalence-and-phenotypic-characterization-of-salmonella-enterica-isolates-from-three-species-of-wild-marine-turtles-in-grenada,-west-indies	02/03/2021
Journal Article	lPrevalence and zoonotic transmission of colistin-resistant and carbapenemase-producing Enterobacterales on German pig farms	2021	6	One Health	13		amr--data--domestic--lprevalence-and-zoonotic-transmission-of-colistin-resistant-and-carbapenemase-producing-enterobacterales-on-german-pig-farms	
Journal Article	Diversity and Risk Factors Associated with Multidrug and Methicillin-Resistant Staphylococci Isolated from Cats Admitted to a Veterinary Clinic in Eastern Province, Saudi Arabia	2021		Antibiotics (Basel)	10	4	amr--domestic--human--diversity-and-risk-factors-associated-with-multidrug-and-methicillin-resistant-staphylococci-isolated-from-cats-admitted-to-a-veterinary-clinic-in-eastern-province,-saudi-arabia	04/04/2021
Journal Article	Exploring the One Health Perspective in Sweden's Policies for Containing Antibiotic Resistance	2021		Antibiotics (Basel)	10	5	amr--eval--policy--exploring-the-one-health-perspective-in-sweden's-policies-for-containing-antibiotic-resistance	03/06/2021
Journal Article	Systematic review and meta-analysis of integrated studies on antimicrobial resistance genes in Africa-A One Health perspective	2021	1153-1163	Trop Med Int Health	26	10	amr--data--domestic--env--human--wild--systematic-review-and-meta-analysis-of-integrated-studies-on-antimicrobial-resistance-genes-in-africa-a-one-health-perspective	18/06/2021
Journal Article	One Health Approach: An Overview of Q Fever in Livestock, Wildlife and Humans in Asturias (Northwestern Spain)	2021	12	Animals	11	5	data--domestic--pathogen--wild--one-health-approach:-an-overview-of-q-fever-in-livestock,-wildlife-and-humans-in-asturias-(northwestern-spain)	
Journal Article	Genomic Analysis of Carbapenem-Resistant Pseudomonas aeruginosa Isolated From Urban Rivers Confirms Spread of Clone Sequence Type 277 Carrying Broad Resistome and Virulome Beyond the Hospital	2021	701921	Front Microbiol	12		amr--data--env--genomic-analysis-of-carbapenem-resistant-pseudomonas-aeruginosa-isolated-from-urban-rivers-confirms-spread-of-clone-sequence-type-277-carrying-broad-resistome-and-virulome-beyond-the-hospital	21/09/2021
Journal Article	Healthy ecosystems for human and animal health: Science diplomacy for responsible development in the Arctic The Nordic Centre of Excellence, Clinf.org (Climate-change effects on the epidemiology of infectious diseases and the impacts on Northern societies)	2021	7	Polar Record	57		opinion--pathogen--risk--healthy-ecosystems-for-human-and-animal-health:-science-diplomacy-for-responsible-development-in-the-arctic-the-nordic-centre-of-excellence,-clinf.org-(climate-change-effects-on-the-epidemiology-of-infectious-diseases-and-the-impacts-on-northern-societies)	
Journal Article	Seabirds as anthropization indicators in two different tropical biotopes: A One Health approach to the issue of antimicrobial resistance genes pollution in oceanic islands	2021	11	Science of the Total Environment	754		amr--data--wild--seabirds-as-anthropization-indicators-in-two-different-tropical-biotopes:-a-one-health-approach-to-the-issue-of-antimicrobial-resistance-genes-pollution-in-oceanic-islands	
Journal Article	Occurrence and Quantification of Antimicrobial Resistance Genes in the Gastrointestinal Microbiome of Two Wild Seabird Species With Contrasting Behaviors	2021	9	Frontiers in Veterinary Science	8		amr--data--wild--occurrence-and-quantification-of-antimicrobial-resistance-genes-in-the-gastrointestinal-microbiome-of-two-wild-seabird-species-with-contrasting-behaviors	
Journal Article	SARS-CoV-2 at the human-animal interphase: A review	2021	e08496	Heliyon	7	12	covid--domestic--human--opinion--wild--sars-cov-2-at-the-human-animal-interphase:-a-review	07/12/2021
Journal Article	The one health landscape in Sub-Saharan African countries	2021	100325	One Health	13		eval--policy--the-one-health-landscape-in-sub-saharan-african-countries	30/09/2021
Journal Article	Lead exposure in dogs fed game meat and offal from culled invasive species in El Palmar National Park, Argentina	2021	45486-45495	Environ Sci Pollut Res Int	28	33	domestic--non-bio--opinion--lead-exposure-in-dogs-fed-game-meat-and-offal-from-culled-invasive-species-in-el-palmar-national-park,-argentina	18/04/2021
Journal Article	The contribution of veterinary public health to the management of the COVID-19 pandemic from a One Health perspective	2021	100230	One Health	12		covid--opinion--the-contribution-of-veterinary-public-health-to-the-management-of-the-covid-19-pandemic-from-a-one-health-perspective	09/03/2021
Journal Article	Lack of Evidence That Bird Feeders Are a Source of Salmonellosis during Winter in Poland	2021	7	Animals	11	6	data--env--pathogen--wild--lack-of-evidence-that-bird-feeders-are-a-source-of-salmonellosis-during-winter-in-poland	
Journal Article	Multiple sequence types, virulence determinants and antimicrobial resistance genes in multidrug- and colistin-resistant Escherichia coli from agricultural and non-agricultural soils	2021	9	Environmental Pollution	288		amr--data--pathogen--wild--multiple-sequence-types,-virulence-determinants-and-antimicrobial-resistance-genes-in-multidrug--and-colistin-resistant-escherichia-coli-from-agricultural-and-non-agricultural-soils	
Journal Article	Health promotion and harm reduction attributes in One Health literature: A scoping review	2021	100284	One Health	13		edu--human--opinion--health-promotion-and-harm-reduction-attributes-in-one-health-literature:-a-scoping-review	13/08/2021
Journal Article	Secrets of the Astute Red Fox (Vulpes vulpes, Linnaeus, 1758): An Inside-Ecosystem Secret Agent Serving One Health	2021	15	Environments	8	10	opinion--wild--secrets-of-the-astute-red-fox-(vulpes-vulpes,-linnaeus,-1758):-an-inside-ecosystem-secret-agent-serving-one-health	
Journal Article	Extended-Spectrum β-Lactam Resistant Klebsiella pneumoniae and Escherichia coli in Wild European Hedgehogs (Erinaceus europeus) Living in Populated Areas	2021		Animals (Basel)	11	10	amr--data--wild--extended-spectrum-β-lactam-resistant-klebsiella-pneumoniae-and-escherichia-coli-in-wild-european-hedgehogs-(erinaceus-europeus)-living-in-populated-areas	24/10/2021
Journal Article	Antimicrobial resistance in Ethiopia: A systematic review and meta-analysis of prevalence in foods, food handlers, animals, and the environment	2021	100286	One Health	13		amr--data--domestic--antimicrobial-resistance-in-ethiopia:-a-systematic-review-and-meta-analysis-of-prevalence-in-foods,-food-handlers,-animals,-and-the-environment	15/07/2021
Journal Article	A One Health Approach to Child Stunting: Evidence and Research Agenda	2021	1620-1624	American Journal of Tropical Medicine and Hygiene	104	5	child-health--human--opinion--a-one-health-approach-to-child-stunting:-evidence-and-research-agenda	
Journal Article	Female and male-controlled livestock holdings impact pastoralist food security and women's dietary diversity	2021	3	One Health Outlook	3		data--eval--human--female-and-male-controlled-livestock-holdings-impact-pastoralist-food-security-and-women's-dietary-diversity	09/04/2021
Journal Article	A high prevalence of bla(OXA-48) in Klebsiella (Raoultella) ornithinolytica and related species in hospital wastewater in South West England	2021		Microb Genom	7	3	data--env--pathogen--a-high-prevalence-of-bla(oxa-48)-in-klebsiella-(raoultella)-ornithinolytica-and-related-species-in-hospital-wastewater-in-south-west-england	09/01/2021
Journal Article	Tracking Animal Reservoirs of Pathogenic Leptospira: The Right Test for the Right Claim	2021		Trop Med Infect Dis	6	4	data--pathogen--wild--tracking-animal-reservoirs-of-pathogenic-leptospira:-the-right-test-for-the-right-claim	24/12/2021
Journal Article	Prioritizing zoonotic diseases using a multisectoral, One Health approach for The Economic Community of West African States (ECOWAS)	2021	24	One Health Outlook	3	1	eval--human--policy--prioritizing-zoonotic-diseases-using-a-multisectoral,-one-health-approach-for-the-economic-community-of-west-african-states-(ecowas)	24/11/2021
Journal Article	Critical Evaluation of Cross-Sectoral Collaborations to Inform the Implementation of the "One Health" Approach in Guadeloupe	2021	652079	Front Public Health	9		eval--critical-evaluation-of-cross-sectoral-collaborations-to-inform-the-implementation-of-the-"one-health"-approach-in-guadeloupe	20/08/2021
Journal Article	From the One Health Perspective: Schistosomiasis Japonica and Flooding	2021		Pathogens	10	12	opinion--pathogen--wild--from-the-one-health-perspective:-schistosomiasis-japonica-and-flooding	29/12/2021
Journal Article	Zoonotic parasites in farmed exotic animals in China: Implications to public health	2021	241-247	Int J Parasitol Parasites Wildl	14		opinion--pathogen--wild--zoonotic-parasites-in-farmed-exotic-animals-in-china:-implications-to-public-health	27/04/2021
Journal Article	Longitudinal study on the effects of growth-promoting and therapeutic antibiotics on the dynamics of chicken cloacal and litter microbiomes and resistomes	2021	178	Microbiome	9	1	amr--data--domestic--longitudinal-study-on-the-effects-of-growth-promoting-and-therapeutic-antibiotics-on-the-dynamics-of-chicken-cloacal-and-litter-microbiomes-and-resistomes	30/08/2021
Journal Article	Dental fluorosis among people and livestock living on Gihaya Island in Lake Kivu, Rwanda	2021	23	One Health Outlook	3	1	data--domestic--human--non-bio--dental-fluorosis-among-people-and-livestock-living-on-gihaya-island-in-lake-kivu,-rwanda	21/12/2021
Journal Article	A scoping review of rodent-borne pathogen presence, exposure, and transmission at zoological institutions	2021	105345	Prev Vet Med	193		data--pathogen--wild--a-scoping-review-of-rodent-borne-pathogen-presence,-exposure,-and-transmission-at-zoological-institutions	07/06/2021
Journal Article	Antimicrobial Resistance in Humans, Animals, Water and Household Environs in Rural Andean Peru: Exploring Dissemination Pathways through the One Health Lens	2021	16	International Journal of Environmental Research and Public Health	18	9	amr--data--domestic--env--human--antimicrobial-resistance-in-humans,-animals,-water-and-household-environs-in-rural-andean-peru:-exploring-dissemination-pathways-through-the-one-health-lens	
Journal Article	One Health Evaluation of Antimicrobial Use and Resistance Surveillance: A Novel Tool for Evaluating Integrated, One Health Antimicrobial Resistance and Antimicrobial Use Surveillance Programs	2021	693703	Front Public Health	9		amr--eval--policy--one-health-evaluation-of-antimicrobial-use-and-resistance-surveillance:-a-novel-tool-for-evaluating-integrated,-one-health-antimicrobial-resistance-and-antimicrobial-use-surveillance-programs	24/08/2021
Journal Article	Advancing One Biosecurity to Address the Pandemic Risks of Biological Invasions	2021	708-721	Bioscience	71	7	opinion--advancing-one-biosecurity-to-address-the-pandemic-risks-of-biological-invasions	
Journal Article	Overview of Cattle Diseases Listed Under Category C, D or E in the Animal Health Law for Which Control Programmes Are in Place Within Europe	2021	688078	Front Vet Sci	8		domestic--eval--risk--overview-of-cattle-diseases-listed-under-category-c,-d-or-e-in-the-animal-health-law-for-which-control-programmes-are-in-place-within-europe	17/08/2021
Journal Article	Assessing Environmental Factors within the One Health Approach	2021		Medicina (Kaunas)	57	3	data--env--eval--assessing-environmental-factors-within-the-one-health-approach	04/04/2021
Journal Article	An Epidemiological Survey Regarding Ticks and Tick-Borne Diseases among Livestock Owners in Punjab, Pakistan: A One Health Context	2021		Pathogens	10	3	an-epidemiological-survey-regarding-ticks-and-tick-borne-diseases-among-livestock-owners-in-punjab,-pakistan:-a-one-health-context	04/04/2021
Journal Article	Presence of Antibiotic Residues and Antibiotic Resistant Bacteria in Cattle Manure Intended for Fertilization of Agricultural Fields: A One Health Perspective	2021		Antibiotics (Basel)	10	4	presence-of-antibiotic-residues-and-antibiotic-resistant-bacteria-in-cattle-manure-intended-for-fertilization-of-agricultural-fields:-a-one-health-perspective	01/05/2021
Journal Article	Canine Leishmaniasis in Morocco: A Descriptive Prospective Clinical Study	2021	12	Veterinary Medicine International	2021		canine-leishmaniasis-in-morocco:-a-descriptive-prospective-clinical-study	
Journal Article	Australian Bat Lyssavirus: Analysis of National Bat Surveillance Data from 2010 to 2016	2021		Viruses	13	2	australian-bat-lyssavirus:-analysis-of-national-bat-surveillance-data-from-2010-to-2016	31/01/2021
Journal Article	Prioritization of zoonotic diseases of public health significance in Nigeria using the one-health approach	2021	100257	One Health	13		prioritization-of-zoonotic-diseases-of-public-health-significance-in-nigeria-using-the-one-health-approach	28/05/2021
Journal Article	How animal agriculture stakeholders define, perceive, and are impacted by antimicrobial resistance: challenging the Wellcome Trust's Reframing Resistance principles	2021	893-909	Agric Human Values	38	4	how-animal-agriculture-stakeholders-define,-perceive,-and-are-impacted-by-antimicrobial-resistance:-challenging-the-wellcome-trust's-reframing-resistance-principles	16/11/2021
Journal Article	Investigation of Armigeres subalbatus, a vector of zoonotic Brugia pahangi filariasis in plantation areas in Suratthani, Southern Thailand	2021	100261	One Health	13		investigation-of-armigeres-subalbatus,-a-vector-of-zoonotic-brugia-pahangi-filariasis-in-plantation-areas-in-suratthani,-southern-thailand	25/05/2021
Journal Article	Improving the dialogue between public health and ecosystem science on antimicrobial resistance	2021	1251-1256	Oikos	130	8	improving-the-dialogue-between-public-health-and-ecosystem-science-on-antimicrobial-resistance	
Journal Article	A demographic survey on the prevalence of gastrointestinal parasites based on socioeconomic determinants in Pakistan	2021	1738-1743	J Infect Dev Ctries	15	11	a-demographic-survey-on-the-prevalence-of-gastrointestinal-parasites-based-on-socioeconomic-determinants-in-pakistan	14/12/2021
Journal Article	Rodent Ectoparasites in the Middle East: A Systematic Review and Meta-Analysis	2021		Pathogens	10	2	rodent-ectoparasites-in-the-middle-east:-a-systematic-review-and-meta-analysis	13/02/2021
Journal Article	Rodent-Related Zoonotic Pathogens at the Human-Animal-Environment Interface in Qatar: A Systematic Review and Meta-Analysis	2021		Int J Environ Res Public Health	18	11	rodent-related-zoonotic-pathogens-at-the-human-animal-environment-interface-in-qatar:-a-systematic-review-and-meta-analysis	03/06/2021
Journal Article	Multisectoral one health approach to make aquaculture and fisheries resilient to a future pandemic-like situation	2021	449-463	Fish and Fisheries	22	2	multisectoral-one-health-approach-to-make-aquaculture-and-fisheries-resilient-to-a-future-pandemic-like-situation	
Journal Article	Easy Access to Antibiotics; Spread of Antimicrobial Resistance and Implementation of One Health Approach in India	2021	444-452	J Epidemiol Glob Health	11	4	easy-access-to-antibiotics;-spread-of-antimicrobial-resistance-and-implementation-of-one-health-approach-in-india	05/11/2021
Journal Article	Blastocystis One Health Approach in a Rural Community of Northern Thailand: Prevalence, Subtypes and Novel Transmission Routes	2021	746340	Front Microbiol	12		blastocystis-one-health-approach-in-a-rural-community-of-northern-thailand:-prevalence,-subtypes-and-novel-transmission-routes	28/12/2021
Journal Article	Identification of Tapeworm Species in Genetically Characterised Grey Wolves Recolonising Central Europe	2021	1063-1067	Acta Parasitol	66	3	identification-of-tapeworm-species-in-genetically-characterised-grey-wolves-recolonising-central-europe	11/02/2021
Journal Article	Developing a one health approach by using a multi-dimensional matrix	2021	100289	One Health	13		developing-a-one-health-approach-by-using-a-multi-dimensional-matrix	27/07/2021
Journal Article	One map: Using geospatial analysis to understand lead exposure across humans, animals, and the environment in an urban US city	2021	100341	One Health	13		one-map:-using-geospatial-analysis-to-understand-lead-exposure-across-humans,-animals,-and-the-environment-in-an-urban-us-city	12/11/2021
Journal Article	Anthropocentric framings of One Health: an analysis of international antimicrobial resistance policy documents	2021	306-315	Critical Public Health	31	3	anthropocentric-framings-of-one-health:-an-analysis-of-international-antimicrobial-resistance-policy-documents	
Journal Article	Implications of Zoonoses From Hunting and Use of Wildlife in North American Arctic and Boreal Biomes: Pandemic Potential, Monitoring, and Mitigation	2021	627654	Front Public Health	9		implications-of-zoonoses-from-hunting-and-use-of-wildlife-in-north-american-arctic-and-boreal-biomes:-pandemic-potential,-monitoring,-and-mitigation	25/05/2021
Journal Article	Antimicrobial resistance profiles of Escherichia coli from swine farms using different antimicrobials and management systems	2021	689-695	Veterinary World	14	3	antimicrobial-resistance-profiles-of-escherichia-coli-from-swine-farms-using-different-antimicrobials-and-management-systems	
Journal Article	High beta-Lactam and Quinolone Resistance of Enterobacteriaceae from the Respiratory Tract of Sheep and Goat with Respiratory Disease	2021	12	Animals	11	8	high-beta-lactam-and-quinolone-resistance-of-enterobacteriaceae-from-the-respiratory-tract-of-sheep-and-goat-with-respiratory-disease	
Journal Article	Prioritizing zoonotic diseases utilizing the One Health approach: Jordan's experience	2021	100262	One Health	13		prioritizing-zoonotic-diseases-utilizing-the-one-health-approach:-jordan's-experience	25/05/2021
Journal Article	Antibiotic resistome from the One-Health perspective: understanding and controlling antimicrobial resistance transmission	2021	301-309	Experimental and Molecular Medicine	53	3	antibiotic-resistome-from-the-one-health-perspective:-understanding-and-controlling-antimicrobial-resistance-transmission	
Journal Article	Gain and loss of antibiotic resistant genes in multidrug resistant bacteria: One Health perspective	2021	535-545	J Microbiol	59	6	gain-and-loss-of-antibiotic-resistant-genes-in-multidrug-resistant-bacteria:-one-health-perspective	21/04/2021
Journal Article	Dysbiosis of the Urinary Bladder Microbiome in Cats with Chronic Kidney Disease	2021	e0051021	mSystems	6	4	dysbiosis-of-the-urinary-bladder-microbiome-in-cats-with-chronic-kidney-disease	28/07/2021
Journal Article	A Pilot Study on the Microbiome of Amblyomma hebraeum Tick Stages Infected and Non-Infected with Rickettsia africae	2021		Pathogens	10	8	a-pilot-study-on-the-microbiome-of-amblyomma-hebraeum-tick-stages-infected-and-non-infected-with-rickettsia-africae	29/08/2021
Journal Article	Efforts to Identify and Combat Antimicrobial Resistance in Uganda: A Systematic Review	2021		Trop Med Infect Dis	6	2	efforts-to-identify-and-combat-antimicrobial-resistance-in-uganda:-a-systematic-review	03/06/2021
Journal Article	A Systematic Review of Environmental Sustainability in Veterinary Practice	2021	8	Topics in Companion Animal Medicine	44		a-systematic-review-of-environmental-sustainability-in-veterinary-practice	
Journal Article	Unpacking the Theory Behind One Health Food Safety Programs: A Vietnam Case Study	2021	763410	Front Vet Sci	8		unpacking-the-theory-behind-one-health-food-safety-programs:-a-vietnam-case-study	21/12/2021
Journal Article	Increasing rabies data availability: The example of a One Health research project in Chad, Côte d'Ivoire and Mali	2021	105808	Acta Trop	215		increasing-rabies-data-availability:-the-example-of-a-one-health-research-project-in-chad,-côte-d'ivoire-and-mali	02/01/2021
Journal Article	The urban lead (Pb) burden in humans, animals and the natural environment	2021	20	Environmental Research	193		the-urban-lead-(pb)-burden-in-humans,-animals-and-the-natural-environment	
Journal Article	Antigenic evolution of contemporary clade 2.3.4.4 HPAI H5 influenza A viruses and impact on vaccine use for mitigation and control	2021	3794-3798	Vaccine	39	29	antigenic-evolution-of-contemporary-clade-2.3.4.4-hpai-h5-influenza-a-viruses-and-impact-on-vaccine-use-for-mitigation-and-control	
Journal Article	Comprehensive Genomic Investigation of Tigecycline Resistance Gene tet(X4)-Bearing Strains Expanding among Different Settings	2021	13	Microbiology Spectrum	9	3	comprehensive-genomic-investigation-of-tigecycline-resistance-gene-tet(x4)-bearing-strains-expanding-among-different-settings	
Journal Article	Ecological and public health significance of Enterocytozoon bieneusi	2021	100209	One Health	12		ecological-and-public-health-significance-of-enterocytozoon-bieneusi	12/01/2021
Journal Article	Operationalizing the ECOWAS regional one health coordination mechanism (2016-2019): Scoping review on progress, challenges and way forward	2021	100291	One Health	13		operationalizing-the-ecowas-regional-one-health-coordination-mechanism-(2016-2019):-scoping-review-on-progress,-challenges-and-way-forward	27/07/2021
Journal Article	Tuberculosis outbreak in intensive swine farming from southern Brazil	2021	5	Ciencia Rural	51	11	tuberculosis-outbreak-in-intensive-swine-farming-from-southern-brazil	
Journal Article	Characterisation of the rumen resistome in Spanish dairy cattle	2021	13	Animal Microbiome	3	1	characterisation-of-the-rumen-resistome-in-spanish-dairy-cattle	
Journal Article	Antimicrobial resistance in Chilean marine-farmed salmon: Improving food safety through One Health	2021	8	One Health	12		antimicrobial-resistance-in-chilean-marine-farmed-salmon:-improving-food-safety-through-one-health	
Journal Article	Epidemiological and numerical simulation of rabies spreading from canines to various human populations in mainland China	2021	21	Plos Neglected Tropical Diseases	15	7	epidemiological-and-numerical-simulation-of-rabies-spreading-from-canines-to-various-human-populations-in-mainland-china	
Journal Article	What evidence exists on the impact of specific ecosystem components and functions on infectious diseases? A systematic map	2021	31	Environmental Evidence	10	1	what-evidence-exists-on-the-impact-of-specific-ecosystem-components-and-functions-on-infectious-diseases?-a-systematic-map	
Journal Article	Simulation of the mucosal environment in the re-construction of the synthetic gut microbial ecosystem	2021	106351	J Microbiol Methods	191		simulation-of-the-mucosal-environment-in-the-re-construction-of-the-synthetic-gut-microbial-ecosystem	29/10/2021
Journal Article	First neuroinvasive human case of West Nile Disease in Southern Italy: Results of the 'One Health' approach	2021	2463-2472	Veterinary Medicine and Science	7	6	first-neuroinvasive-human-case-of-west-nile-disease-in-southern-italy:-results-of-the-'one-health'-approach	
Journal Article	Taeniid cestodes in a wolf pack living in a highly anthropic hilly agro-ecosystem	2021	10	Parasite	28		taeniid-cestodes-in-a-wolf-pack-living-in-a-highly-anthropic-hilly-agro-ecosystem	06/02/2021
Journal Article	Applying a One Health Approach in Global Health and Medicine: Enhancing Involvement of Medical Schools and Global Health Centers	2021	30	Ann Glob Health	87	1	applying-a-one-health-approach-in-global-health-and-medicine:-enhancing-involvement-of-medical-schools-and-global-health-centers	06/04/2021
Journal Article	Gaps in health security related to wildlife and environment affecting pandemic prevention and preparedness, 2007-2020	2021	342-350b	Bull World Health Organ	99	5	gaps-in-health-security-related-to-wildlife-and-environment-affecting-pandemic-prevention-and-preparedness,-2007-2020	08/05/2021
Journal Article	OneHealth Approaches Contribute Towards Antimicrobial Resistance: Malaysian Perspective	2021	718774	Front Microbiol	12		onehealth-approaches-contribute-towards-antimicrobial-resistance:-malaysian-perspective	12/11/2021
Journal Article	Antimicrobial Resistance Profiles of Coagulase-Negative Staphylococci in Community-Based Healthy Individuals in Germany	2021	9	Frontiers in Public Health	9		antimicrobial-resistance-profiles-of-coagulase-negative-staphylococci-in-community-based-healthy-individuals-in-germany	
Journal Article	The Honey Bee: An Active Biosampler of Environmental Pollution and a Possible Warning Biomarker for Human Health	2021	14	Applied Sciences-Basel	11	14	the-honey-bee:-an-active-biosampler-of-environmental-pollution-and-a-possible-warning-biomarker-for-human-health	
Journal Article	Tackling modern-day crises: Why understanding multilevel interconnectivity is vital	2021	e2000294	Bioessays	43	3	opinion--tackling-modern-day-crises:-why-understanding-multilevel-interconnectivity-is-vital	15/12/2020
Journal Article	Knowledge Gaps in the Understanding of Antimicrobial Resistance in Canada	2021	726484	Front Public Health	9		amr--opinion--knowledge-gaps-in-the-understanding-of-antimicrobial-resistance-in-canada	16/11/2021
Journal Article	Delivering Modern Global Health Learning Requires New Obligations and Approaches	2021	68	Ann Glob Health	87	1	edu--human--delivering-modern-global-health-learning-requires-new-obligations-and-approaches	27/07/2021
Journal Article	Antimicrobial Resistance in Rural Settings in Latin America: A Scoping Review with a One Health Lens	2021	18	International Journal of Environmental Research and Public Health	18	18	amr--data--opinion--risk--antimicrobial-resistance-in-rural-settings-in-latin-america:-a-scoping-review-with-a-one-health-lens	
Journal Article	Zoonotic Dirofilaria immitis and Dirofilaria repens infection in humans and an integrative approach to the diagnosis	2021	106083	Acta Trop	223		data--domestic--pathogen--zoonotic-dirofilaria-immitis-and-dirofilaria-repens-infection-in-humans-and-an-integrative-approach-to-the-diagnosis	09/08/2021
Journal Article	Molecular detection of vector-borne agents in ectoparasites and reptiles from Brazil	2021	101585	Ticks Tick Borne Dis	12	1	data--pathogen--wild--molecular-detection-of-vector-borne-agents-in-ectoparasites-and-reptiles-from-brazil	29/10/2020
Journal Article	Role of reptiles and associated arthropods in the epidemiology of rickettsioses: A one health paradigm	2021	e0009090	PLoS Negl Trop Dis	15	2	data--human--pathogen--role-of-reptiles-and-associated-arthropods-in-the-epidemiology-of-rickettsioses:-a-one-health-paradigm	18/02/2021
Journal Article	An exploration of the protective effect of rodent species richness on the geographical expansion of Lassa fever in West Africa	2021	e0009108	PLoS Negl Trop Dis	15	2	data--pathogen--risk--wild--an-exploration-of-the-protective-effect-of-rodent-species-richness-on-the-geographical-expansion-of-lassa-fever-in-west-africa	02/02/2021
Journal Article	Trends, relationships and case attribution of antibiotic resistance between children and environmental sources in rural India	2021	11	Scientific Reports	11	1	amr--data--domestic--env--human--risk--wild--trends,-relationships-and-case-attribution-of-antibiotic-resistance-between-children-and-environmental-sources-in-rural-india	
Journal Article	CAFOs, novel influenza, and the need for One Health approaches	2021	100246	One Health	13		domestic--opinion--pathogen--policy--cafos,-novel-influenza,-and-the-need-for-one-health-approaches	18/05/2021
Journal Article	Systematic Review of Hepatitis E Virus in Brazil: A One-Health Approach of the Human-Animal-Environment Triad	2021		Animals (Basel)	11	8	data--domestic--pathogen--systematic-review-of-hepatitis-e-virus-in-brazil:-a-one-health-approach-of-the-human-animal-environment-triad	28/08/2021
Journal Article	Surveillance system for avian influenza in wild birds and implications of its improvement with insights into the highly pathogenic avian influenza outbreaks in Japan	2021	13	Preventive Veterinary Medicine	187		eval--opinion--risk--wild--surveillance-system-for-avian-influenza-in-wild-birds-and-implications-of-its-improvement-with-insights-into-the-highly-pathogenic-avian-influenza-outbreaks-in-japan	
Journal Article	Exposure to domoic acid is an ecological driver of cardiac disease in southern sea otters(☆)	2021	101973	Harmful Algae	101		data--non-bio--wild--exposure-to-domoic-acid-is-an-ecological-driver-of-cardiac-disease-in-southern-sea-otters(☆)	03/02/2021
Journal Article	Hepatitis E virus in professionally exposed: A reason for concern?	2021	723-730	World J Hepatol	13	7	domestic--human--opinion--pathogen--hepatitis-e-virus-in-professionally-exposed:-a-reason-for-concern?	10/08/2021
Journal Article	Antimicrobial Resistance Situational Analysis 2019-2020: Design and Performance for Human Health Surveillance in Uganda	2021		Trop Med Infect Dis	6	4	amr--eval--policy--antimicrobial-resistance-situational-analysis-2019-2020:-design-and-performance-for-human-health-surveillance-in-uganda	27/10/2021
Journal Article	Access, use and disposal of antimicrobials among humans and animals in Wakiso district, Uganda: a qualitative study	2021	12	Journal of Pharmaceutical Policy and Practice	14	1	amr--data--eval--human--access,-use-and-disposal-of-antimicrobials-among-humans-and-animals-in-wakiso-district,-uganda:-a-qualitative-study	
Journal Article	Live and Wet Markets: Food Access versus the Risk of Disease Emergence	2021	573-581	Trends Microbiol	29	7	pathogen--risk--live-and-wet-markets:-food-access-versus-the-risk-of-disease-emergence	14/03/2021
Journal Article	The role of Environmental Health in preventing antimicrobial resistance in low- and middle-income countries	2021	100	Environ Health Prev Med	26	1	amr--env--opinion--the-role-of-environmental-health-in-preventing-antimicrobial-resistance-in-low--and-middle-income-countries	07/10/2021
Journal Article	Antimicrobial Use Surveillance Indicators for Finfish Aquaculture Production: A Review	2021	595152	Front Vet Sci	8		amr--eval--policy--antimicrobial-use-surveillance-indicators-for-finfish-aquaculture-production:-a-review	30/03/2021
Journal Article	Association Between COVID-19 Exposure and Self-reported Compliance With Public Health Guidelines Among Essential Employees at an Institution of Higher Education in the US	2021	e2116543	JAMA Netw Open	4	7	covid--eval--human--association-between-covid-19-exposure-and-self-reported-compliance-with-public-health-guidelines-among-essential-employees-at-an-institution-of-higher-education-in-the-us	22/07/2021
Journal Article	The Behavior of Consumers and Producers of Food of Animal Origin and Their Impacts in One Health	2021	641634	Front Vet Sci	8		edu--human--the-behavior-of-consumers-and-producers-of-food-of-animal-origin-and-their-impacts-in-one-health	02/07/2021
Journal Article	Microbial Contamination of Chicken Litter Manure and Antimicrobial Resistance Threat in an Urban Area Setting in Cameroon	2021	12	Antibiotics-Basel	10	1	amr--data--domestic--pathogen--microbial-contamination-of-chicken-litter-manure-and-antimicrobial-resistance-threat-in-an-urban-area-setting-in-cameroon	
Journal Article	Prospective One Health genetic surveillance in Vietnam identifies distinct bla(CTX-M)-harbouring Escherichia coli in food-chain and human-derived samples	2021	1515.e1-1515.e8	Clin Microbiol Infect	27	10	amr--data--domestic--prospective-one-health-genetic-surveillance-in-vietnam-identifies-distinct-bla(ctx-m)-harbouring-escherichia-coli-in-food-chain-and-human-derived-samples	22/01/2021
Journal Article	Perceptions of dog owners towards canine gastrointestinal parasitism and associated human health risk in Southeast Queensland	2021	100226	One Health	12		data--domestic--pathogen--perceptions-of-dog-owners-towards-canine-gastrointestinal-parasitism-and-associated-human-health-risk-in-southeast-queensland	06/03/2021
Journal Article	A qualitative assessment of the context and enabling environment for the control of Taenia solium infections in endemic settings	2021	e0009470	PLoS Negl Trop Dis	15	6	data--eval--human--pathogen--a-qualitative-assessment-of-the-context-and-enabling-environment-for-the-control-of-taenia-solium-infections-in-endemic-settings	12/06/2021
Journal Article	Antimicrobial resistance in Galapagos tortoises as an indicator of the growing human footprint*	2021	9	Environmental Pollution	284		amr--data--wild--antimicrobial-resistance-in-galapagos-tortoises-as-an-indicator-of-the-growing-human-footprint*	
Journal Article	Antimicrobial resistance: causes, consequences, diagnostic and therapeutic challenges in modern medicine	2021	8	Medycyna Weterynaryjna-Veterinary Medicine-Science and Practice	77	12	amr--opinion--antimicrobial-resistance:-causes,-consequences,-diagnostic-and-therapeutic-challenges-in-modern-medicine	
Journal Article	Evaluation of bat adenoviruses suggests co-evolution and host roosting behaviour as drivers for diversity	2021		Microb Genom	7	4	data--pathogen--wild--evaluation-of-bat-adenoviruses-suggests-co-evolution-and-host-roosting-behaviour-as-drivers-for-diversity	20/04/2021
Journal Article	The One Health approach to incident management of the 2019 Lassa fever outbreak response in Nigeria	2021	100346	One Health	13		eval--opinion--pathogen--the-one-health-approach-to-incident-management-of-the-2019-lassa-fever-outbreak-response-in-nigeria	26/11/2021
Journal Article	COVID 19: A CALL TO ONE HEALTH ACTION	2021	S31-s37	Ann Ib Postgrad Med	19	Suppl 1	covid--opinion--covid-19:-a-call-to-one-health-action	01/02/2022
Journal Article	Occurrence of tick-borne pathogens in questing Ixodes ricinus ticks from Wester Ross, Northwest Scotland	2021	430	Parasit Vectors	14	1	data--env--pathogen--occurrence-of-tick-borne-pathogens-in-questing-ixodes-ricinus-ticks-from-wester-ross,-northwest-scotland	28/08/2021
Journal Article	A One-Health Quantitative Model to Assess the Risk of Antibiotic Resistance Acquisition in Asian Populations: Impact of Exposure Through Food, Water, Livestock and Humans	2021	1427-1446	Risk Analysis	41	8	amr--domestic--env--human--risk--wild--a-one-health-quantitative-model-to-assess-the-risk-of-antibiotic-resistance-acquisition-in-asian-populations:-impact-of-exposure-through-food,-water,-livestock-and-humans	
Journal Article	Veterinary-Prescribed Physical Activity: Feasibility and Acceptability among Veterinary Staff and Dog Owners	2021		Int J Environ Res Public Health	18	5	data--domestic--non-bio--veterinary-prescribed-physical-activity:-feasibility-and-acceptability-among-veterinary-staff-and-dog-owners	07/03/2021
Journal Article	Antimicrobial Resistance Patterns and Risk Factors Associated with Salmonella spp. Isolates from Poultry Farms in the East Coast of Peninsular Malaysia: A Cross-Sectional Study	2021		Pathogens	10	9	data--domestic--env--pathogen--antimicrobial-resistance-patterns-and-risk-factors-associated-with-salmonella-spp.-isolates-from-poultry-farms-in-the-east-coast-of-peninsular-malaysia:-a-cross-sectional-study	29/09/2021
Journal Article	Emergence and Spread of Cephalosporinases in Wildlife: A Review	2021		Animals (Basel)	11	6	amr--data--wild--emergence-and-spread-of-cephalosporinases-in-wildlife:-a-review	03/07/2021
Journal Article	One Health Approach to Rickettsiosis: A Five-Year Study on Spotted Fever Group Rickettsiae in Ticks Collected From Humans, Animals and Environment	2021		Microorganisms	10	1	data--env--pathogen--one-health-approach-to-rickettsiosis:-a-five-year-study-on-spotted-fever-group-rickettsiae-in-ticks-collected-from-humans,-animals-and-environment	22/01/2022
Journal Article	Emerging and re-emerging viral infections in India	2021	E628-e634	J Prev Med Hyg	62	3	eval--pathogen--policy--emerging-and-re-emerging-viral-infections-in-india	16/12/2021
Journal Article	A Critical Review of Disinfection Processes to Control SARS-CoV-2 Transmission in the Food Industry	2021	16	Foods	10	2	covid--eval--opinion--a-critical-review-of-disinfection-processes-to-control-sars-cov-2-transmission-in-the-food-industry	
Journal Article	Dog bite Emergency department presentations in Brisbane metro south: Epidemiology and exploratory medical geography for targeted interventions	2021	100204	One Health	12		domestic--non-bio--risk--dog-bite-emergency-department-presentations-in-brisbane-metro-south:-epidemiology-and-exploratory-medical-geography-for-targeted-interventions	29/12/2020
Journal Article	Antimicrobial multidrug resistance in the era of COVID-19: a forgotten plight?	2021	21	Antimicrob Resist Infect Control	10	1	amr--opinion--policy--antimicrobial-multidrug-resistance-in-the-era-of-covid-19:-a-forgotten-plight?	31/01/2021
Journal Article	Antibiotic-Resistant Bacteria in Aquaculture and Climate Change: A Challenge for Health in the Mediterranean Area	2021		Int J Environ Res Public Health	18	11	amr--climate--opinion--antibiotic-resistant-bacteria-in-aquaculture-and-climate-change:-a-challenge-for-health-in-the-mediterranean-area	03/06/2021
Journal Article	Bushmeat, wet markets, and the risks of pandemics: Exploring the nexus through systematic review of scientific disclosures	2021	1-11	Environ Sci Policy	124		covid--opinion--bushmeat,-wet-markets,-and-the-risks-of-pandemics:-exploring-the-nexus-through-systematic-review-of-scientific-disclosures	01/10/2021
Journal Article	From the Approach to the Concept: One Health in Latin America-Experiences and Perspectives in Brazil, Chile, and Colombia	2021	18	Frontiers in Public Health	9		opinion--from-the-approach-to-the-concept:-one-health-in-latin-america-experiences-and-perspectives-in-brazil,-chile,-and-colombia	
Journal Article	Klebsiella pneumoniae Complex Harboring mcr-1, mcr-7, and mcr-8 Isolates from Slaughtered Pigs in Thailand	2021		Microorganisms	9	12	amr--data--domestic--klebsiella-pneumoniae-complex-harboring-mcr-1,-mcr-7,-and-mcr-8-isolates-from-slaughtered-pigs-in-thailand	25/12/2021
Journal Article	One welfare impacts of COVID-19-A summary of key highlights within the one welfare framework	2021	10	Applied Animal Behaviour Science	236		covid--opinion--one-welfare-impacts-of-covid-19-a-summary-of-key-highlights-within-the-one-welfare-framework	
Journal Article	Use of the Milan Pet Quality of Life Instrument (MPQL) to Measure Pets' Quality of Life during COVID-19	2021		Animals (Basel)	11	5	domestic--eval--use-of-the-milan-pet-quality-of-life-instrument-(mpql)-to-measure-pets'-quality-of-life-during-covid-19	03/06/2021
Journal Article	Wildlife as Sentinels of Antimicrobial Resistance in Germany?	2021	15	Frontiers in Veterinary Science	7		amr--data--pathogen--wild--wildlife-as-sentinels-of-antimicrobial-resistance-in-germany?	
Journal Article	Dissemination of Extended-Spectrum-β-Lactamase-Producing Enterobacter cloacae Complex from a Hospital to the Nearby Environment in Guadeloupe (French West Indies): ST114 Lineage Coding for a Successful IncHI2/ST1 Plasmid	2021		Antimicrob Agents Chemother	65	3	amr--data--env--dissemination-of-extended-spectrum-β-lactamase-producing-enterobacter-cloacae-complex-from-a-hospital-to-the-nearby-environment-in-guadeloupe-(french-west-indies):-st114-lineage-coding-for-a-successful-inchi2/st1-plasmid	29/12/2020
Journal Article	A One Health perspective of the impacts of microplastics on animal, human and environmental health	2021	146094	Sci Total Environ	777		non-bio--opinion--a-one-health-perspective-of-the-impacts-of-microplastics-on-animal,-human-and-environmental-health	08/03/2021
Journal Article	Extended spectrum beta-lactamase-producing Escherichia coli surveillance in the human, food chain, and environment sectors: Tricycle project (pilot) in Indonesia	2021	100331	One Health	13		amr--data--domestic--env--human--extended-spectrum-beta-lactamase-producing-escherichia-coli-surveillance-in-the-human,-food-chain,-and-environment-sectors:-tricycle-project-(pilot)-in-indonesia	12/10/2021
Journal Article	Evidence of Borrelia theileri in Wild and Domestic Animals in the Kafue Ecosystem of Zambia	2021		Microorganisms	9	11	data--domestic--pathogen--wild--evidence-of-borrelia-theileri-in-wild-and-domestic-animals-in-the-kafue-ecosystem-of-zambia	28/11/2021
Journal Article	The Coexistence of Blastocystis spp. in Humans, Animals and Environmental Sources from 2010-2021 in Asia	2021	40	Biology-Basel	10	10	opinion--pathogen--the-coexistence-of-blastocystis-spp.-in-humans,-animals-and-environmental-sources-from-2010-2021-in-asia	
Journal Article	Crop protection practices and viral zoonotic risks within a One Health framework	2021	145172	Sci Total Environ	774		domestic--opinion--pathogen--crop-protection-practices-and-viral-zoonotic-risks-within-a-one-health-framework	22/02/2021
Journal Article	Biosecurity Concept: Origins, Evolution and Perspectives	2021		Animals (Basel)	12	1	opinion--biosecurity-concept:-origins,-evolution-and-perspectives	12/01/2022
Journal Article	Transmission modelling of environmentally persistent zoonotic diseases: a systematic review	2021	e466-e478	Lancet Planet Health	5	7	pathogen--risk--transmission-modelling-of-environmentally-persistent-zoonotic-diseases:-a-systematic-review	11/07/2021
Journal Article	A POTENTIAL ZOONOTIC PARASITE: CRYPTOSPORIDIUM PARVUM TRANSMISSION IN RATS, PIGS AND HUMANS IN WEST LOMBOK, INDONESIA	2021	44-51	Afr J Infect Dis	15	2	data--domestic--human--pathogen--wild--a-potential-zoonotic-parasite:-cryptosporidium-parvum-transmission-in-rats,-pigs-and-humans-in-west-lombok,-indonesia	24/04/2021
Journal Article	Should the Increased Awareness of the One Health Approach Brought by the COVID-19 Pandemic Be Used to Further Tackle the Challenge of Antimicrobial Resistance?	2021	8	Antibiotics-Basel	10	4	amr--covid--domestic--opinion--should-the-increased-awareness-of-the-one-health-approach-brought-by-the-covid-19-pandemic-be-used-to-further-tackle-the-challenge-of-antimicrobial-resistance?	
Journal Article	One Health Pandemic Prevention and Mitigation: The Role of FDA	2021	200-234	Food and Drug Law Journal	76	2	opinion--policy--one-health-pandemic-prevention-and-mitigation:-the-role-of-fda	
Journal Article	One Health in Indigenous Communities: A Critical Review of the Evidence	2021	12	International Journal of Environmental Research and Public Health	18	21	eval--human--opinion--one-health-in-indigenous-communities:-a-critical-review-of-the-evidence	
Journal Article	Human Colonization with Extended-Spectrum Beta-Lactamase-Producing E. coli in Relation to Animal and Environmental Exposures in Bangladesh: An Observational One Health Study	2021	13	Environmental Health Perspectives	129	3	amr--data--human--risk--human-colonization-with-extended-spectrum-beta-lactamase-producing-e.-coli-in-relation-to-animal-and-environmental-exposures-in-bangladesh:-an-observational-one-health-study	
Journal Article	The Role of Biosecurity in the Control of Campylobacter: A Qualitative Study of the Attitudes and Perceptions of UK Broiler Farm Workers	2021	751699	Front Vet Sci	8		data--eval--human--pathogen--the-role-of-biosecurity-in-the-control-of-campylobacter:-a-qualitative-study-of-the-attitudes-and-perceptions-of-uk-broiler-farm-workers	08/01/2022
Journal Article	A One Health Approach to Defining Animal and Human Helminth Exposure Risks in a Tribal Village in Southern India	2021	196-203	American Journal of Tropical Medicine and Hygiene	105	1	data--domestic--env--pathogen--risk--a-one-health-approach-to-defining-animal-and-human-helminth-exposure-risks-in-a-tribal-village-in-southern-india	
Journal Article	A comprehensive bibliometric overview: antibiotic resistance and Escherichia coli in natural water	2021	32256-32263	Environmental Science and Pollution Research	28	25	amr--opinion--a-comprehensive-bibliometric-overview:-antibiotic-resistance-and-escherichia-coli-in-natural-water	
Journal Article	Role of soil in the regulation of human and plant pathogens: soils' contributions to people	2021	13	Philosophical Transactions of the Royal Society B-Biological Sciences	376	1834	env--opinion--pathogen--role-of-soil-in-the-regulation-of-human-and-plant-pathogens:-soils'-contributions-to-people	
Journal Article	Circulating Brucella species in wild animals of the Serengeti ecosystem, Tanzania	2021	9	One Health Outlook	3	1	data--pathogen--wild--circulating-brucella-species-in-wild-animals-of-the-serengeti-ecosystem,-tanzania	
Journal Article	Spillover of zoonotic pathogens: A review of reviews	2021	563-577	Zoonoses and Public Health	68	6	eval--pathogen--risk--spillover-of-zoonotic-pathogens:-a-review-of-reviews	
Journal Article	The meiofauna as neglected carriers of antibiotic resistant and pathogenic bacteria in freshwater ecosystems	2021	14	Journal of Limnology	80	3	amr--data--wild--the-meiofauna-as-neglected-carriers-of-antibiotic-resistant-and-pathogenic-bacteria-in-freshwater-ecosystems	
Journal Article	Socializing One Health: an innovative strategy to investigate social and behavioral risks of emerging viral threats	2021	11	One Health Outlook	3	1	eval--human--pathogen--policy--socializing-one-health:-an-innovative-strategy-to-investigate-social-and-behavioral-risks-of-emerging-viral-threats	16/05/2021

Journal Article	Molecular detection and genetic diversity of Rickettsia spp. in pet dogs and their infesting ticks in Harbin, northeastern China	2021	9	Bmc Veterinary Research	17	1	data--domestic--pathogen--molecular-detection-and-genetic-diversity-of-rickettsia-spp.-in-pet-dogs-and-their-infesting-ticks-in-harbin,-northeastern-china	
Journal Article	Prevalence of non-typhoidal Salmonella and risk factors on poultry farms in Chitwan, Nepal	2021	426-436	Veterinary World	14	2	data--domestic--pathogen--prevalence-of-non-typhoidal-salmonella-and-risk-factors-on-poultry-farms-in-chitwan,-nepal	
Journal Article	Foodborne Illness Outbreak Investigation for One Health Postsecondary Education	2021	12	Journal of Microbiology & Biology Education	22	2	edu--eval--foodborne-illness-outbreak-investigation-for-one-health-postsecondary-education	
Journal Article	Epidemiological profiling of SARS-CoV-2 with focus on one-health approaches in mitigating COVID-19 pandemic	2021	791-802	Indian Journal of Animal Sciences	91	10	covid--opinion--epidemiological-profiling-of-sars-cov-2-with-focus-on-one-health-approaches-in-mitigating-covid-19-pandemic	
Journal Article	Unpacking the myth between increased government initiatives and reduced selling of dead live stocks in China: An approach towards exploring hidden danger of zoonotic diseases	2021	100344	One Health	13		domestic--pathogen--policy--risk--unpacking-the-myth-between-increased-government-initiatives-and-reduced-selling-of-dead-live-stocks-in-china:-an-approach-towards-exploring-hidden-danger-of-zoonotic-diseases	23/11/2021
Journal Article	Black-and-White Ruffed Lemur (Varecia variegata) in Captivity: Analysis of the Oral Microbiota in a One Health Perspective	2021	9	Animals	11	10	amr--data--wild--black-and-white-ruffed-lemur-(varecia-variegata)-in-captivity:-analysis-of-the-oral-microbiota-in-a-one-health-perspective	
Journal Article	Environmental Spread of Antibiotic Resistance	2021		Antibiotics (Basel)	10	6	amr--opinion--environmental-spread-of-antibiotic-resistance	03/06/2021
Journal Article	Tetracycline Exposure Alters Key Gut Microbiota in Africanized Honey Bees (Apis mellifera scutellata x spp.)	2021	11	Frontiers in Ecology and Evolution	9		amr--data--wild--tetracycline-exposure-alters-key-gut-microbiota-in-africanized-honey-bees-(apis-mellifera-scutellata-x-spp.)	
Journal Article	One health disparities and COVID-19	2021	70-77	Evolution Medicine and Public Health	9	1	covid--eval--one-health-disparities-and-covid-19	
Journal Article	PastoralScape: An Environment-Driven Model of Vaccination Decision Making within Pastoralist Groups in East Africa	2021	21	Jasss-the Journal of Artificial Societies and Social Simulation	24	4	climate--domestic--human--pathogen--risk--pastoralscape:-an-environment-driven-model-of-vaccination-decision-making-within-pastoralist-groups-in-east-africa	
Journal Article	Strategies for Implementing a One Welfare Framework into Emergency Management	2021	12	Animals	11	11	opinion--policy--strategies-for-implementing-a-one-welfare-framework-into-emergency-management	
Journal Article	One Health Aquaculture - a personal perspective	2021	188-191	Bulletin of the European Association of Fish Pathologists	41	5	opinion--one-health-aquaculture---a-personal-perspective	
Journal Article	Serology for Borrelia spp. in Northwest Italy: A Climate-Matched 10-Year Trend	2021		Life (Basel)	11	12	climate--data--human--pathogen--risk--serology-for-borrelia-spp.-in-northwest-italy:-a-climate-matched-10-year-trend	25/12/2021
Journal Article	Detection and characterization of ESBL-producing Enterobacteriaceae from the gut of subsistence farmers, their livestock, and the surrounding environment in rural Nepal	2021	13	Scientific Reports	11	1	amr--data--human--detection-and-characterization-of-esbl-producing-enterobacteriaceae-from-the-gut-of-subsistence-farmers,-their-livestock,-and-the-surrounding-environment-in-rural-nepal	
Journal Article	A One Health approach to assessing occupational exposure to antimicrobial resistance in Thailand: The FarmResist project	2021	e0245250	PLoS One	16	1	amr--data--domestic--human--wild--a-one-health-approach-to-assessing-occupational-exposure-to-antimicrobial-resistance-in-thailand:-the-farmresist-project	29/01/2021
Journal Article	Canine hyperactivity, impulsivity, and inattention share similar demographic risk factors and behavioural comorbidities with human ADHD	2021	9	Translational Psychiatry	11	1	data--domestic--canine-hyperactivity,-impulsivity,-and-inattention-share-similar-demographic-risk-factors-and-behavioural-comorbidities-with-human-adhd	
Journal Article	The occurrence and abundance of infective stages of zoonotic nematodes in selected edible fish sold in Australian fish markets	2021	104833	Microb Pathog	154		data--human--pathogen--risk--wild--the-occurrence-and-abundance-of-infective-stages-of-zoonotic-nematodes-in-selected-edible-fish-sold-in-australian-fish-markets	13/03/2021
Journal Article	Viral Zoonoses of National Importance in Ghana: Advancements and Opportunities for Enhancing Capacities for Early Detection and Response	2021	8	Journal of Tropical Medicine	2021		opinion--pathogen--policy--viral-zoonoses-of-national-importance-in-ghana:-advancements-and-opportunities-for-enhancing-capacities-for-early-detection-and-response	
Journal Article	The effects of fipronil on emotional and cognitive behaviors in mammals	2021	104847	Pestic Biochem Physiol	175		data--domestic--non-bio--the-effects-of-fipronil-on-emotional-and-cognitive-behaviors-in-mammals	18/05/2021
Journal Article	Synzootics	2021	2744-2754	Journal of Animal Ecology	90	12	opinion--synzootics	
Journal Article	Global prevalence of Cryptosporidium spp. in cats: A systematic review and meta-analysis	2021	77-85	Research in Veterinary Science	137		data--domestic--human--pathogen--global-prevalence-of-cryptosporidium-spp.-in-cats:-a-systematic-review-and-meta-analysis	
Journal Article	A review of the toxicology of oil in vertebrates: what we have learned following the Deepwater Horizon oil spill	2021	355-394	J Toxicol Environ Health B Crit Rev	24	8	data--non-bio--wild--a-review-of-the-toxicology-of-oil-in-vertebrates:-what-we-have-learned-following-the-deepwater-horizon-oil-spill	21/09/2021
Journal Article	Lead exposure in consumers of culled invasive alien mammals in El Palmar National Park, Argentina	2021	42432-42443	Environ Sci Pollut Res Int	28	31	data--human--non-bio--wild--lead-exposure-in-consumers-of-culled-invasive-alien-mammals-in-el-palmar-national-park,-argentina	05/04/2021
Journal Article	Cross-Sectoral Zoonotic Disease Surveillance in Western Kenya: Identifying Drivers and Barriers Within a Resource Constrained Setting	2021	13	Frontiers in Veterinary Science	8		eval--human--cross-sectoral-zoonotic-disease-surveillance-in-western-kenya:-identifying-drivers-and-barriers-within-a-resource-constrained-setting	
Journal Article	Emergence of colistin resistance genes (mcr-1) in Escherichia coli among widely distributed wild ungulates	2021	7	Environmental Pollution	291		amr--data--wild--emergence-of-colistin-resistance-genes-(mcr-1)-in-escherichia-coli-among-widely-distributed-wild-ungulates	
Journal Article	Lead concentrations and isotope ratios in blood, breastmilk and feces: contribution of both lactation and soil/dust exposure to infants in a lead	2021	10	Environmental Pollution	286		data--human--non-bio--lead-concentrations-and-isotope-ratios-in-blood,-breastmilk-and-feces:-contribution-of-both-lactation-and-soil/dust-exposure-to-infants-in-a-lead	
Journal Article	Health Impacts of Perchlorate and Pesticide Exposure: Protocol for Community-Engaged Research to Evaluate Environmental Toxicants in a US Border Community	2021	e15864	JMIR Res Protoc	10	8	data--human--non-bio--health-impacts-of-perchlorate-and-pesticide-exposure:-protocol-for-community-engaged-research-to-evaluate-environmental-toxicants-in-a-us-border-community	13/08/2021
Journal Article	Marine invertebrate interactions with Harmful Algal Blooms-Implications for One Health	2021	22	Journal of Invertebrate Pathology	186		env--opinion--pathogen--marine-invertebrate-interactions-with-harmful-algal-blooms-implications-for-one-health	
Journal Article	Robotic Antimicrobial Susceptibility Platform (RASP): a next-generation approach to One Health surveillance of antimicrobial resistance	2021	1800-1807	Journal of Antimicrobial Chemotherapy	76	7	amr--risk--robotic-antimicrobial-susceptibility-platform-(rasp):-a-next-generation-approach-to-one-health-surveillance-of-antimicrobial-resistance	
Journal Article	Imported Hyalomma ticks in the Netherlands 2018-2020	2021	244	Parasit Vectors	14	1	climate--data--domestic--human--pathogen--imported-hyalomma-ticks-in-the-netherlands-2018-2020	09/05/2021
Journal Article	Detection of bla(OXA-1), bla(TEM-1), and Virulence Factors in E. coli Isolated From Seals	2021	10	Frontiers in Veterinary Science	8		amr--data--wild--detection-of-bla(oxa-1),-bla(tem-1),-and-virulence-factors-in-e.-coli-isolated-from-seals	
Journal Article	REVIEWING THE ROLE OF VULTURES AT THE HUMAN-WILDLIFE-LIVESTOCK DISEASE INTERFACE: AN AFRICAN PERSPECTIVE	2021	311-327	Journal of Raptor Research	55	3	opinion--pathogen--wild--reviewing-the-role-of-vultures-at-the-human-wildlife-livestock-disease-interface:-an-african-perspective	
Journal Article	Surveillance of foodborne parasitic diseases in Europe in a One Health approach	2021	10	Parasite Epidemiology and Control	13		eval--pathogen--policy--surveillance-of-foodborne-parasitic-diseases-in-europe-in-a-one-health-approach	
Journal Article	The Precautionary Principle in Zoonotic Disease Control	2021	180-190	Public Health Ethics	14	2	opinion--pathogen--the-precautionary-principle-in-zoonotic-disease-control	
Journal Article	Interaction Patterns between Wildlife and Cattle Reveal Opportunities for Mycobacteria Transmission in Farms from North-Eastern Atlantic Iberian Peninsula	2021	20	Animals	11	8	data--domestic--pathogen--risk--wild--interaction-patterns-between-wildlife-and-cattle-reveal-opportunities-for-mycobacteria-transmission-in-farms-from-north-eastern-atlantic-iberian-peninsula	
Journal Article	Occupational exposure to Aspergillus section Fumigati: Tackling the knowledge gap in Portugal	2021	13	Environmental Research	194		env--opinion--pathogen--occupational-exposure-to-aspergillus-section-fumigati:-tackling-the-knowledge-gap-in-portugal	
Journal Article	Emerging Trends in the Epidemiology of COVID-19: The Croatian 'One Health' Perspective	2021		Viruses	13	12	covid--domestic--opinion--emerging-trends-in-the-epidemiology-of-covid-19:-the-croatian-'one-health'-perspective	29/12/2021
Journal Article	Rabies control in Liberia: Joint efforts towards zero by 30	2021	105787	Acta Trop	216		data--domestic--pathogen--rabies-control-in-liberia:-joint-efforts-towards-zero-by-30	02/01/2021
Journal Article	Predators in northern Germany are reservoirs for parasites of One Health concern	2021	4229-4239	Parasitology Research	120	12	data--pathogen--wild--predators-in-northern-germany-are-reservoirs-for-parasites-of-one-health-concern	
Journal Article	The Animal-foods-environment interface of Klebsiella pneumoniae in Germany: an observational study on pathogenicity, resistance development and the current situation	2021	14	Veterinary Research	52	1	opinion--pathogen--the-animal-foods-environment-interface-of-klebsiella-pneumoniae-in-germany:-an-observational-study-on-pathogenicity,-resistance-development-and-the-current-situation	
Journal Article	More diversified antibiotic resistance genes in chickens and workers of the live poultry markets	2021	13	Environment International	153		amr--data--domestic--human--more-diversified-antibiotic-resistance-genes-in-chickens-and-workers-of-the-live-poultry-markets	
Journal Article	The evolutionary history of ACE2 usage within the coronavirus subgenus Sarbecovirus	2021	veab007	Virus Evol	7	1	covid--data--human--the-evolutionary-history-of-ace2-usage-within-the-coronavirus-subgenus-sarbecovirus	24/03/2021
Journal Article	Human density is associated with the increased prevalence of a generalist zoonotic parasite in mammalian wildlife	2021	9	Proceedings of the Royal Society B-Biological Sciences	288	1961	data--pathogen--wild--human-density-is-associated-with-the-increased-prevalence-of-a-generalist-zoonotic-parasite-in-mammalian-wildlife	
Journal Article	Ecology of domestic dogs (Canis familiaris) as a host for Guinea worm (Dracunculus medinensis) infection in Ethiopia	2021	531-542	Transbound Emerg Dis	68	2	domestic--pathogen--risk--ecology-of-domestic-dogs-(canis-familiaris)-as-a-host-for-guinea-worm-(dracunculus-medinensis)-infection-in-ethiopia	03/07/2020
Journal Article	Finding What Is Inaccessible: Antimicrobial Resistance Language Use among the One Health Domains	2021	17	Antibiotics-Basel	10	4	amr--opinion--finding-what-is-inaccessible:-antimicrobial-resistance-language-use-among-the-one-health-domains	
Journal Article	Slaughterhouse Workers, Animals, and the Environment: The Need for a Rights-Centered Regulatory Framework in the United States That Recognizes Interconnected Interests	2021	21-34	Health and Human Rights	23	2	covid--eval--policy--slaughterhouse-workers,-animals,-and-the-environment:-the-need-for-a-rights-centered-regulatory-framework-in-the-united-states-that-recognizes-interconnected-interests	
Journal Article	Concept of an Active Surveillance System for Q Fever in German Small Ruminants-Conflicts Between Best Practices and Feasibility	2021	623786	Front Vet Sci	8		eval--pathogen--risk--concept-of-an-active-surveillance-system-for-q-fever-in-german-small-ruminants-conflicts-between-best-practices-and-feasibility	02/03/2021
Journal Article	Harmful algal bloom resources for livestock veterinarians	2021	151-161	Javma-Journal of the American Veterinary Medical Association	259	2	edu--non-bio--pathogen--policy--harmful-algal-bloom-resources-for-livestock-veterinarians	
Journal Article	Revealing the distribution characteristics of antibiotic resistance genes and bacterial communities in animal-aerosol-human in a chicken farm: From One-Health perspective	2021	11	Ecotoxicology and Environmental Safety	224		amr--data--domestic--env--revealing-the-distribution-characteristics-of-antibiotic-resistance-genes-and-bacterial-communities-in-animal-aerosol-human-in-a-chicken-farm:-from-one-health-perspective	
Journal Article	Systemic factors for enhancing intersectoral collaboration for the operationalization of One Health: a case study in India	2021	14	Health Research Policy and Systems	19	1	eval--policy--systemic-factors-for-enhancing-intersectoral-collaboration-for-the-operationalization-of-one-health:-a-case-study-in-india	
Journal Article	Health System Contact and Awareness of Zoonotic Diseases: Can it Serve as One Health Entry Point in the Urban Community of Ahmedabad, India?	2021	259-269	Yale Journal of Biology and Medicine	94	2	data--eval--human--pathogen--health-system-contact-and-awareness-of-zoonotic-diseases:-can-it-serve-as-one-health-entry-point-in-the-urban-community-of-ahmedabad,-india?	
Journal Article	Updates on the global dissemination of colistin-resistant Escherichia coli: An emerging threat to public health	2021	18	Science of the Total Environment	799		amr--domestic--env--human--opinion--wild--updates-on-the-global-dissemination-of-colistin-resistant-escherichia-coli:-an-emerging-threat-to-public-health	
Journal Article	First detection of an Italian human-to-cat outbreak of SARS-CoV-2 Alpha variant-lineage B.1.1.7	2021	6	One Health	13		covid--data--domestic--human--first-detection-of-an-italian-human-to-cat-outbreak-of-sars-cov-2-alpha-variant-lineage-b.1.1.7	
Journal Article	Hiding in plain sight-wildlife as a neglected reservoir and pathway for the spread of antimicrobial resistance: a narrative review	2022	25	Fems Microbiology Ecology	98	6	amr--opinion--wild--hiding-in-plain-sight-wildlife-as-a-neglected-reservoir-and-pathway-for-the-spread-of-antimicrobial-resistance:-a-narrative-review	
Journal Article	Prevalence and Characterisation of Multiresistant Bacterial Strains Isolated in Pigs from the Island of Tenerife	2022	10	Veterinary Sciences	9	6	amr--data--domestic--pathogen--prevalence-and-characterisation-of-multiresistant-bacterial-strains-isolated-in-pigs-from-the-island-of-tenerife	
Journal Article	Towards a One Health Food Safety Strategy for Palestine: A Mixed-Method Study	2022		Antibiotics (Basel)	11	10	domestic--pathogen--risk--towards-a-one-health-food-safety-strategy-for-palestine:-a-mixed-method-study	28/10/2022
Journal Article	Systematic Review and Meta-Analysis of Integrated Studies on Salmonella and Campylobacter Prevalence, Serovar, and Phenotyping and Genetic of Antimicrobial Resistance in the Middle East-A One Health Perspective	2022	17	Antibiotics-Basel	11	5	amr--data--domestic--human--pathogen--systematic-review-and-meta-analysis-of-integrated-studies-on-salmonella-and-campylobacter-prevalence,-serovar,-and-phenotyping-and-genetic-of-antimicrobial-resistance-in-the-middle-east-a-one-health-perspective	
Journal Article	Implementation of one health approach in Jordan: Joint risk assessment of rabies and avian influenza utilizing the tripartite operational tool	2022	6	One Health	15		pathogen--risk--implementation-of-one-health-approach-in-jordan:-joint-risk-assessment-of-rabies-and-avian-influenza-utilizing-the-tripartite-operational-tool	
Journal Article	Taeniasis-cysticercosis complex in the agrarian reform rural settlements, Brazil	2022	8	Pesquisa Veterinaria Brasileira	42		domestic--env--pathogen--risk--taeniasis-cysticercosis-complex-in-the-agrarian-reform-rural-settlements,-brazil	
Journal Article	HOW MISUSE OF ANTIMICROBIAL AGENTS IS EXACERBATING THE CHALLENGES FACING SOMALIA'S PUBLIC HEALTH	2022	26-32	Afr J Infect Dis	16	2 Suppl	opinion--policy--how-misuse-of-antimicrobial-agents-is-exacerbating-the-challenges-facing-somalia's-public-health	21/09/2022
Journal Article	Evolution and implementation of One Health to control the dissemination of antibiotic-resistant bacteria and resistance genes: A review	2022	1065796	Front Cell Infect Microbiol	12		opinion--evolution-and-implementation-of-one-health-to-control-the-dissemination-of-antibiotic-resistant-bacteria-and-resistance-genes:-a-review	03/02/2023
Journal Article	Estimating the financial impact of livestock schistosomiasis on traditional subsistence and transhumance farmers keeping cattle, sheep and goats in northern Senegal	2022	20	Parasites & Vectors	15	1	domestic--human--pathogen--risk--estimating-the-financial-impact-of-livestock-schistosomiasis-on-traditional-subsistence-and-transhumance-farmers-keeping-cattle,-sheep-and-goats-in-northern-senegal	
Journal Article	Campylobacter in Wild Birds: Is It an Animal and Public Health Concern?	2022	11	Frontiers in Microbiology	12		opinion--pathogen--wild--campylobacter-in-wild-birds:-is-it-an-animal-and-public-health-concern?	
Journal Article	Medical interns' attitudes towards One Health approach	2022	137-144	Turkish Journal of Biochemistry-Turk Biyokimya Dergisi	47	1	eval--human--medical-interns'-attitudes-towards-one-health-approach	
Journal Article	Methodology for laboratory-based antimicrobial resistance surveillance in animals	2022	1066-1079	Veterinary World	15	4	amr--policy--methodology-for-laboratory-based-antimicrobial-resistance-surveillance-in-animals	
Journal Article	Waiting for markets to change me-High-stakeholders' views of antibiotic use and antibiotic resistance in pig production in Brazil	2022	11	Frontiers in Veterinary Science	9		amr--domestic--eval--human--waiting-for-markets-to-change-me-high-stakeholders'-views-of-antibiotic-use-and-antibiotic-resistance-in-pig-production-in-brazil	
Journal Article	Veterinary and Pharmacy Students' Expectations Before and Experiences After Participating in an Interdisciplinary Access to Care Veterinary Clinic, WisCARES	2022	610-617	Journal of Veterinary Medical Education	49	5	edu--veterinary-and-pharmacy-students'-expectations-before-and-experiences-after-participating-in-an-interdisciplinary-access-to-care-veterinary-clinic,-wiscares	
Journal Article	Assessment of the Transmission Dynamics of Clostridioides difficile in a Farm Environment Reveals the Presence of a New Toxigenic Strain Connected to Swine Production	2022	14	Frontiers in Microbiology	13		data--domestic--env--human--pathogen--assessment-of-the-transmission-dynamics-of-clostridioides-difficile-in-a-farm-environment-reveals-the-presence-of-a-new-toxigenic-strain-connected-to-swine-production	
Journal Article	Antimicrobial Resistance, Biofilm Formation, and Virulence Genes in Enterococcus Species from Small Backyard Chicken Flocks	2022		Antibiotics (Basel)	11	3	data--domestic--pathogen--antimicrobial-resistance,-biofilm-formation,-and-virulence-genes-in-enterococcus-species-from-small-backyard-chicken-flocks	26/03/2022
Journal Article	Isolation of extended-spectrum β-lactamase-producing Escherichia coli from Japanese red fox (Vulpes vulpes japonica)	2022	e1317	Microbiologyopen	11	5	amr--data--wild--isolation-of-extended-spectrum-β-lactamase-producing-escherichia-coli-from-japanese-red-fox-(vulpes-vulpes-japonica)	01/11/2022
Journal Article	Foodborne Pathogens and Antimicrobial Resistance in Ethiopia: An Urgent Call for Action on "One Health"	2022	5265-5274	Infection and Drug Resistance	15		opinion--pathogen--foodborne-pathogens-and-antimicrobial-resistance-in-ethiopia:-an-urgent-call-for-action-on-"one-health"	
Journal Article	Dynamics of SARS-CoV-2 spreading under the influence of environmental factors and strategies to tackle the pandemic: A systematic review	2022	103840	Sustain Cities Soc	81		covid--eval--dynamics-of-sars-cov-2-spreading-under-the-influence-of-environmental-factors-and-strategies-to-tackle-the-pandemic:-a-systematic-review	24/03/2022
Journal Article	Knowledge, perceptions, and attitudes by residents in Punjab and Khyber Pakhtunkhwa, Pakistan in connection with bats	2022	43	J Ethnobiol Ethnomed	18	1	data--eval--human--wild--knowledge,-perceptions,-and-attitudes-by-residents-in-punjab-and-khyber-pakhtunkhwa,-pakistan-in-connection-with-bats	07/06/2022
Journal Article	Multidrug-Resistant Gram-Negative Bacteria Contaminating Raw Meat Sold in Accra, Ghana	2022	11	Pathogens	11	12	amr--data--domestic--multidrug-resistant-gram-negative-bacteria-contaminating-raw-meat-sold-in-accra,-ghana	
Journal Article	Ecological Distribution of Virulent Multidrug-Resistant Staphylococcus aureus in Livestock, Environment, and Dairy Products	2022		Antibiotics (Basel)	11	11	data--domestic--env--pathogen--ecological-distribution-of-virulent-multidrug-resistant-staphylococcus-aureus-in-livestock,-environment,-and-dairy-products	25/11/2022
Journal Article	Natural detoxification of antibiotics in the environment: A one health perspective	2022	11	Frontiers in Microbiology	13		amr--opinion--natural-detoxification-of-antibiotics-in-the-environment:-a-one-health-perspective	
Journal Article	Susceptibility of Pets to SARS-CoV-2 Infection: Lessons from a Seroepidemiologic Survey of Cats and Dogs in Portugal	2022	15	Microorganisms	10	2	covid--data--domestic--susceptibility-of-pets-to-sars-cov-2-infection:-lessons-from-a-seroepidemiologic-survey-of-cats-and-dogs-in-portugal	
Journal Article	Designing and validating a One Health Research Translation Framework through literature-based case studies in Egypt	2022	7	One Health	15		edu--policy--designing-and-validating-a-one-health-research-translation-framework-through-literature-based-case-studies-in-egypt	
Journal Article	Accumulation of Metals in the Environment and Grazing Livestock near A Mongolian Mining Area	2022		Toxics	10	12	data--domestic--env--non-bio--accumulation-of-metals-in-the-environment-and-grazing-livestock-near-a-mongolian-mining-area	23/12/2022
Journal Article	Surveillance of Coxiella burnetii Shedding in Three Naturally Infected Dairy Goat Herds after Vaccination, Focusing on Bulk Tank Milk and Dust Swabs	2022		Vet Sci	9	3	data--domestic--pathogen--surveillance-of-coxiella-burnetii-shedding-in-three-naturally-infected-dairy-goat-herds-after-vaccination,-focusing-on-bulk-tank-milk-and-dust-swabs	25/03/2022
Journal Article	Cows as canaries: The effects of ambient air pollution exposure on milk production and somatic cell count in dairy cows	2022	10	Environmental Research	207		domestic--non-bio--risk--cows-as-canaries:-the-effects-of-ambient-air-pollution-exposure-on-milk-production-and-somatic-cell-count-in-dairy-cows	
Journal Article	Optimising predictive models to prioritise viral discovery in zoonotic reservoirs	2022	e625-e637	Lancet Microbe	3	8	eval--pathogen--wild--optimising-predictive-models-to-prioritise-viral-discovery-in-zoonotic-reservoirs	18/01/2022
Journal Article	Methicillin-Resistant Staphylococcus aureus (MRSA) and Other Methicillin-Resistant Staphylococci and Mammaliicoccus (MRNaS) Associated with Animals and Food Products in Arab Countries: A Review	2022	17	Veterinary Sciences	9	7	opinion--pathogen--methicillin-resistant-staphylococcus-aureus-(mrsa)-and-other-methicillin-resistant-staphylococci-and-mammaliicoccus-(mrnas)-associated-with-animals-and-food-products-in-arab-countries:-a-review	
Journal Article	Zoonotic diseases: New directions in human-animal pathology	2022	548-552	International Journal of Osteoarchaeology	32	3	opinion--pathogen--zoonotic-diseases:-new-directions-in-human-animal-pathology	
Journal Article	Detection of SARS-CoV-2 in urban stormwater: An environmental reservoir and potential interface between human and animal sources	2022	10	Science of the Total Environment	807		covid--data--env--detection-of-sars-cov-2-in-urban-stormwater:-an-environmental-reservoir-and-potential-interface-between-human-and-animal-sources	
Journal Article	Anti-Sporothrix Antibody Detection in Domestic Cats as an Indicator of a Possible New Occurrence Area for Sporotrichosis in North Brazil	2022	375-384	Mycopathologia	187	4	data--domestic--pathogen--anti-sporothrix-antibody-detection-in-domestic-cats-as-an-indicator-of-a-possible-new-occurrence-area-for-sporotrichosis-in-north-brazil	02/07/2022
Journal Article	Spatial evaluation of animal health care accessibility and veterinary shortage in France	2022	13022	Sci Rep	12	1	domestic--risk--spatial-evaluation-of-animal-health-care-accessibility-and-veterinary-shortage-in-france	30/07/2022
Journal Article	On the importance of integrating comparative anatomy and One Health perspectives in anatomy education	2022	429-446	Journal of Anatomy	240	3	edu--policy--on-the-importance-of-integrating-comparative-anatomy-and-one-health-perspectives-in-anatomy-education	
Journal Article	The One-Health approach in seaweed food production	2022	13	Environment International	158		env--opinion--wild--the-one-health-approach-in-seaweed-food-production	
Journal Article	Swedish Efforts to Contain Antibiotic Resistance in the Environment-A Qualitative Study among Selected Stakeholders	2022		Antibiotics (Basel)	11	5	amr--eval--policy--swedish-efforts-to-contain-antibiotic-resistance-in-the-environment-a-qualitative-study-among-selected-stakeholders	29/05/2022
Journal Article	The UK Antimicrobial Resistance Strategy 2013-18: A Qualitative Study of International and Domestic Policy and Action Related to Livestock and the Food Chain	2022	15	Frontiers in Sustainable Food Systems	6		amr--eval--the-uk-antimicrobial-resistance-strategy-2013-18:-a-qualitative-study-of-international-and-domestic-policy-and-action-related-to-livestock-and-the-food-chain	
Journal Article	Research Participation Influences Willingness to Reduce Zoonotic Exposure in Uganda	2022	299-314	Ecohealth	19	2	domestic--edu--pathogen--wild--research-participation-influences-willingness-to-reduce-zoonotic-exposure-in-uganda	09/06/2022
Journal Article	Environmental Contamination by Parasites in Public Parks in Belgrade in the Context of One Health Approach	2022	30-44	Acta Veterinaria-Beograd	72	1	data--env--pathogen--environmental-contamination-by-parasites-in-public-parks-in-belgrade-in-the-context-of-one-health-approach	
Journal Article	Minnesota One Health Antibiotic Stewardship Collaborative: A Reproducible Approach to Facilitate Antimicrobial Stewardship Progress	2022	ofac602	Open Forum Infect Dis	9	12	eval--policy--minnesota-one-health-antibiotic-stewardship-collaborative:-a-reproducible-approach-to-facilitate-antimicrobial-stewardship-progress	22/12/2022
Journal Article	Detection of an IMI-2 carbapenemase-producing Enterobacter asburiae at a Swedish feed mill	2022	12	Frontiers in Microbiology	13		amr--data--domestic--human--pathogen--detection-of-an-imi-2-carbapenemase-producing-enterobacter-asburiae-at-a-swedish-feed-mill	
Journal Article	VectorNet: Putting Vectors on the Map	2022	809763	Front Public Health	10		eval--pathogen--vectornet:-putting-vectors-on-the-map	22/04/2022
Journal Article	Multidrug-Resistant High-Risk Escherichia coli and Klebsiella pneumoniae Clonal Lineages Occur in Black-Headed Gulls from Two Conservation Islands in Germany	2022		Antibiotics (Basel)	11	10	amr--data--wild--multidrug-resistant-high-risk-escherichia-coli-and-klebsiella-pneumoniae-clonal-lineages-occur-in-black-headed-gulls-from-two-conservation-islands-in-germany	28/10/2022
Journal Article	One Health collaboration with and among EU Agencies - Bridging research and policy	2022	100464	One Health	15		policy--one-health-collaboration-with-and-among-eu-agencies---bridging-research-and-policy	24/12/2022
Journal Article	A Meta-Analysis to Estimate Prevalence of Resistance to Tetracyclines and Third Generation Cephalosporins in Enterobacteriaceae Isolated from Food Crops	2022		Antibiotics (Basel)	11	10	amr--data--domestic--a-meta-analysis-to-estimate-prevalence-of-resistance-to-tetracyclines-and-third-generation-cephalosporins-in-enterobacteriaceae-isolated-from-food-crops	28/10/2022
Journal Article	Whole genome sequence analysis of ESBL-producing Escherichia coli recovered from New Zealand freshwater sites	2022		Microb Genom	8	10	amr--data--env--whole-genome-sequence-analysis-of-esbl-producing-escherichia-coli-recovered-from-new-zealand-freshwater-sites	07/10/2022
Journal Article	Ionizing radiation and melanism in Chornobyl tree frogs	2022	1469-1479	Evol Appl	15	9	data--non-bio--wild--ionizing-radiation-and-melanism-in-chornobyl-tree-frogs	04/10/2022
Journal Article	Characterizing Antimicrobial Resistance in Clinically Relevant Bacteria Isolated at the Human/Animal/Environment Interface Using Whole-Genome Sequencing in Austria	2022	19	International Journal of Molecular Sciences	23	19	amr--data--env--characterizing-antimicrobial-resistance-in-clinically-relevant-bacteria-isolated-at-the-human/animal/environment-interface-using-whole-genome-sequencing-in-austria	
Journal Article	Antimicrobial Resistance as a Global Health Threat: The Need to Learn Lessons from the COVID-19 Pandemic	2022	179-192	Glob Policy	13	2	opinion--policy--antimicrobial-resistance-as-a-global-health-threat:-the-need-to-learn-lessons-from-the-covid-19-pandemic	24/05/2022
Journal Article	Characterization of the genetic structure of mcr-1 gene among Escherichia coli isolates recovered from surface waters and sediments from Ecuador	2022	150566	Sci Total Environ	806	Pt 2	amr--data--env--characterization-of-the-genetic-structure-of-mcr-1-gene-among-escherichia-coli-isolates-recovered-from-surface-waters-and-sediments-from-ecuador	29/09/2021
Journal Article	Orbivirus RNA in a Banana Serotine (Afronycteris nanus) Bat in the Republic of the Congo	2022	443-449	Ecohealth	19	4	data--pathogen--wild--orbivirus-rna-in-a-banana-serotine-(afronycteris-nanus)-bat-in-the-republic-of-the-congo	12/01/2023
Journal Article	Prevalence of Leishmania infantum in Dogs from Deforested Areas of the Amazon Biome	2022	108-113	Vector Borne Zoonotic Dis	22	2	data--domestic--pathogen--prevalence-of-leishmania-infantum-in-dogs-from-deforested-areas-of-the-amazon-biome	14/12/2021
Journal Article	Carbapenemase-producing Klebsiella pneumoniae strains in Switzerland: human and non-human settings may share high-risk clones	2022	206-215	J Glob Antimicrob Resist	28		amr--data--domestic--human--pathogen--carbapenemase-producing-klebsiella-pneumoniae-strains-in-switzerland:-human-and-non-human-settings-may-share-high-risk-clones	28/01/2022
Journal Article	In critique of anthropocentrism: a more-than-human ethical framework for antimicrobial resistance	2022	9	Medical Humanities	48	4	amr--opinion--in-critique-of-anthropocentrism:-a-more-than-human-ethical-framework-for-antimicrobial-resistance	
Journal Article	Occurrence and Genomic Characterization of mcr-1-Harboring Escherichia coli Isolates from Chicken and Pig Farms in Lima, Peru	2022		Antibiotics (Basel)	11	12	amr--data--domestic--occurrence-and-genomic-characterization-of-mcr-1-harboring-escherichia-coli-isolates-from-chicken-and-pig-farms-in-lima,-peru	24/12/2022
Journal Article	Antibiotic susceptibility among non-clinical Escherichia coli as a marker of antibiotic pressure in Peru (2009-2019): one health approach	2022	e10573	Heliyon	8	9	amr--data--domestic--human--wild--antibiotic-susceptibility-among-non-clinical-escherichia-coli-as-a-marker-of-antibiotic-pressure-in-peru-(2009-2019):-one-health-approach	20/09/2022
Journal Article	An Interdisciplinary Approach to One Health: Course Design, Development, and Delivery	2022	568-574	Journal of Veterinary Medical Education	49	5	edu--eval--an-interdisciplinary-approach-to-one-health:-course-design,-development,-and-delivery	
Journal Article	Clinically healthy household dogs and cats as carriers of multidrug-resistant Salmonella enterica with variable R plasmids	2022		J Med Microbiol	71	2	amr--data--domestic--clinically-healthy-household-dogs-and-cats-as-carriers-of-multidrug-resistant-salmonella-enterica-with-variable-r-plasmids	15/02/2022
Journal Article	Spore-Forming Clostridium (Clostridioides) difficile in Wastewater Treatment Plants in Western Australia	2022	11	Microbiology Spectrum			amr--data--env--pathogen--spore-forming-clostridium-(clostridioides)-difficile-in-wastewater-treatment-plants-in-western-australia	
Journal Article	Understanding and strengthening wildlife and zoonotic disease policy processes: A research imperative	2022	768-776	Zoonoses Public Health	69	6	opinion--policy--understanding-and-strengthening-wildlife-and-zoonotic-disease-policy-processes:-a-research-imperative	14/07/2022
Journal Article	Fecal Microbiome Features Associated with Extended-Spectrum beta-Lactamase-Producing Enterobacterales Carriage in Dairy Heifers	2022	12	Animals	12	14	amr--data--domestic--fecal-microbiome-features-associated-with-extended-spectrum-beta-lactamase-producing-enterobacterales-carriage-in-dairy-heifers	
Journal Article	Prevalence and distribution of extended-spectrum beta-lactamase and AmpC-producing Escherichia coli in two New Zealand dairy farm environments	2022	20	Frontiers in Microbiology	13		amr--data--domestic--prevalence-and-distribution-of-extended-spectrum-beta-lactamase-and-ampc-producing-escherichia-coli-in-two-new-zealand-dairy-farm-environments	
Journal Article	Novel Insights into bla(GES) Mobilome Reveal Extensive Genetic Variation in Hospital Effluents	2022	13	Microbiology Spectrum			amr--data--human--novel-insights-into-bla(ges)-mobilome-reveal-extensive-genetic-variation-in-hospital-effluents	
Journal Article	Perception and Knowledge of Portuguese Veterinarians about the Zoonotic Transmission of Helicobacter pylori and Helicobacter suis: The Need for One Health Intervention	2022		Int J Environ Res Public Health	19	22	data--edu--human--pathogen--perception-and-knowledge-of-portuguese-veterinarians-about-the-zoonotic-transmission-of-helicobacter-pylori-and-helicobacter-suis:-the-need-for-one-health-intervention	27/11/2022
Journal Article	Preliminary Investigation about Aspergillus spp. Spread in Umbrian Avian Farms	2022		J Fungi (Basel)	8	11	amr--data--env--pathogen--preliminary-investigation-about-aspergillus-spp.-spread-in-umbrian-avian-farms	25/11/2022
Journal Article	Serological survey of anti-Leptospira spp. antibodies in individuals with animal hoarding disorder and their dogs in a major city of Southern Brazil	2022	530-536	Vet Med Sci	8	2	data--domestic--pathogen--wild--serological-survey-of-anti-leptospira-spp.-antibodies-in-individuals-with-animal-hoarding-disorder-and-their-dogs-in-a-major-city-of-southern-brazil	02/03/2022
Journal Article	CHANS-Law: preventing the next pandemic through the integration of social and environmental law	2022	577-597	Int Environ Agreem	22	3	opinion--pathogen--policy--chans-law:-preventing-the-next-pandemic-through-the-integration-of-social-and-environmental-law	22/03/2022
Journal Article	How public health crises expose systemic, day-to-day health inequalities in low- and-middle income countries: an example from East Africa	2022	34	Antimicrob Resist Infect Control	11	1	amr--data--eval--human--how-public-health-crises-expose-systemic,-day-to-day-health-inequalities-in-low--and-middle-income-countries:-an-example-from-east-africa	16/02/2022
Journal Article	mcr-mediated colistin resistance in South America, One Health approach: a review	2022	E119-E136	Reviews and Research in Medical Microbiology	33	1	amr--opinion--mcr-mediated-colistin-resistance-in-south-america,-one-health-approach:-a-review	
Journal Article	Community dog program in five municipalities of Parana, Brazil	2022	10-16	Journal of Veterinary Behavior-Clinical Applications and Research	58		domestic--eval--community-dog-program-in-five-municipalities-of-parana,-brazil	
Journal Article	Toxoplasmosis in Human and Animals Around the World. Diagnosis and Perspectives in the One Health Approach	2022	106432	Acta Trop	231		opinion--pathogen--toxoplasmosis-in-human-and-animals-around-the-world.-diagnosis-and-perspectives-in-the-one-health-approach	08/04/2022
Journal Article	Exploring the Bacteriome and Resistome of Humans and Food-Producing Animals in Brazil	2022	e0056522	Microbiol Spectr	10	5	amr--data--domestic--exploring-the-bacteriome-and-resistome-of-humans-and-food-producing-animals-in-brazil	23/08/2022
Journal Article	Screen the unforeseen: Microbiome-profiling for detection of zoonotic pathogens in wild rats	2022	3881-3895	Transbound Emerg Dis	69	6	data--pathogen--wild--screen-the-unforeseen:-microbiome-profiling-for-detection-of-zoonotic-pathogens-in-wild-rats	22/11/2022
Journal Article	One Health, One Hive: A scoping review of honey bees, climate change, pollutants, and antimicrobial resistance	2022	e0242393	PLoS One	17	2	opinion--wild--one-health,-one-hive:-a-scoping-review-of-honey-bees,-climate-change,-pollutants,-and-antimicrobial-resistance	17/02/2022
Journal Article	Diversity in the Characteristics of Klebsiella pneumoniae ST101 of Human, Environmental, and Animal Origin	2022	838207	Front Microbiol	13		data--domestic--env--human--pathogen--diversity-in-the-characteristics-of-klebsiella-pneumoniae-st101-of-human,-environmental,-and-animal-origin	01/03/2022
Journal Article	One Health Index (OHI) applied to Curitiba, the ninth-largest metropolitan area of Brazil, with concomitant assessment of animal, environmental, and human health indicators	2022	100373	One Health	14		risk--one-health-index-(ohi)-applied-to-curitiba,-the-ninth-largest-metropolitan-area-of-brazil,-with-concomitant-assessment-of-animal,-environmental,-and-human-health-indicators	11/06/2022
Journal Article	Drought altered trophic dynamics of an important natural saline lake: A stable isotope approach	2022	155338	Sci Total Environ	834		climate--env--risk--wild--drought-altered-trophic-dynamics-of-an-important-natural-saline-lake:-a-stable-isotope-approach	23/04/2022
Journal Article	Detection of Toxoplasma gondii oocysts in soil and risk mapping in an island environment in the Northeast of Brazil	2022	3457-3467	Transbound Emerg Dis	69	6	data--env--pathogen--detection-of-toxoplasma-gondii-oocysts-in-soil-and-risk-mapping-in-an-island-environment-in-the-northeast-of-brazil	11/09/2022
Journal Article	Assessment of the Costs Related to West Nile Virus Monitoring in Lombardy Region (Italy) between 2014 and 2018	2022		Int J Environ Res Public Health	19	9	eval--pathogen--assessment-of-the-costs-related-to-west-nile-virus-monitoring-in-lombardy-region-(italy)-between-2014-and-2018	15/05/2022
Journal Article	How are large-scale One Health initiatives targeting infectious diseases and antimicrobial resistance evaluated? A scoping review	2022	100380	One Health	14		data--eval--how-are-large-scale-one-health-initiatives-targeting-infectious-diseases-and-antimicrobial-resistance-evaluated?-a-scoping-review	08/04/2022
Journal Article	One Health and human health	2022	37-46	Chinese Science Bulletin-Chinese	67	1	opinion--one-health-and-human-health	
Journal Article	Limited Transmission of Klebsiella pneumoniae among Humans, Animals, and the Environment in a Caribbean Island, Guadeloupe (French West Indies)	2022	e0124222	Microbiol Spectr	10	5	amr--data--domestic--human--pathogen--wild--limited-transmission-of-klebsiella-pneumoniae-among-humans,-animals,-and-the-environment-in-a-caribbean-island,-guadeloupe-(french-west-indies)	13/09/2022
Journal Article	Getting out of crises: Environmental, social-ecological and evolutionary research is needed to avoid future risks of pandemics	2022	12	Environment International	158		covid--opinion--getting-out-of-crises:-environmental,-social-ecological-and-evolutionary-research-is-needed-to-avoid-future-risks-of-pandemics	
Journal Article	Synergies and Trade-Offs in Reducing Impacts of Unconventional Oil and Gas Development on Wildlife and Human Health	2022	472-480	Bioscience	72	5	env--human--non-bio--opinion--synergies-and-trade-offs-in-reducing-impacts-of-unconventional-oil-and-gas-development-on-wildlife-and-human-health	
Journal Article	Oh, deer! How worried should we be about the diversity and abundance of the faecal resistome of red deer?	2022	153831	Sci Total Environ	825		amr--data--wild--oh,-deer!-how-worried-should-we-be-about-the-diversity-and-abundance-of-the-faecal-resistome-of-red-deer?	14/02/2022
Journal Article	A closer look on the variety and abundance of the faecal resistome of wild boar	2022	118406	Environ Pollut	292	Pt B	amr--data--wild--a-closer-look-on-the-variety-and-abundance-of-the-faecal-resistome-of-wild-boar	29/10/2021
Journal Article	The Human Health Implications of Antibiotic Resistance in Environmental Isolates from Two Nebraska Watersheds	2022	e0208221	Microbiol Spectr	10	2	amr--data--env--the-human-health-implications-of-antibiotic-resistance-in-environmental-isolates-from-two-nebraska-watersheds	22/03/2022
Journal Article	Occurrence of Naegleria fowleri and their implication for health- a look under the One Health approaches	2022	12	International Journal of Hygiene and Environmental Health	246		climate--opinion--pathogen--occurrence-of-naegleria-fowleri-and-their-implication-for-health--a-look-under-the-one-health-approaches	
Journal Article	A framework for measuring timeliness in the outbreak response path: lessons learned from the Middle East respiratory syndrome (MERS) epidemic, September 2012 to January 2019	2022		Euro Surveill	27	48	eval--pathogen--a-framework-for-measuring-timeliness-in-the-outbreak-response-path:-lessons-learned-from-the-middle-east-respiratory-syndrome-(mers)-epidemic,-september-2012-to-january-2019	26/01/2023
Journal Article	Earth System's Gatekeeping of "One Health" Approach to Manage Climate-Sensitive Infectious Diseases	2022	e2021GH000543	Geohealth	6	4	climate--opinion--pathogen--earth-system's-gatekeeping-of-"one-health"-approach-to-manage-climate-sensitive-infectious-diseases	26/04/2022
Journal Article	Mycobacterium paratuberculosis zoonosis is a One Health emergency	2022	164-174	Ecohealth	19	2	opinion--pathogen--mycobacterium-paratuberculosis-zoonosis-is-a-one-health-emergency	03/06/2022
Journal Article	Development, feasibility and potential effectiveness of community-based continuous mass dog vaccination delivery strategies: Lessons for optimization and replication	2022	e0010318	PLoS Negl Trop Dis	16	9	domestic--eval--pathogen--policy--development,-feasibility-and-potential-effectiveness-of-community-based-continuous-mass-dog-vaccination-delivery-strategies:-lessons-for-optimization-and-replication	07/09/2022
Journal Article	Nature-Based One Health Approaches to Urban Agriculture Can Deliver Food and Nutrition Security	2022	773746	Front Nutr	9		opinion--policy--nature-based-one-health-approaches-to-urban-agriculture-can-deliver-food-and-nutrition-security	02/04/2022
Journal Article	Genomic insights into zoonotic transmission and antimicrobial resistance in Campylobacter jejuni from farm to fork: a one health perspective	2022	11	Gut Pathogens	14	1	opinion--pathogen--genomic-insights-into-zoonotic-transmission-and-antimicrobial-resistance-in-campylobacter-jejuni-from-farm-to-fork:-a-one-health-perspective	
Journal Article	Synthesizing the connections between environmental disturbances and zoonotic spillover	2022	33	Anais Da Academia Brasileira De Ciencias	94		opinion--pathogen--synthesizing-the-connections-between-environmental-disturbances-and-zoonotic-spillover	
Journal Article	One Health: A social science discussion of a global agenda	2022	17	Parasite	29		opinion--pathogen--one-health:-a-social-science-discussion-of-a-global-agenda	23/03/2022
Journal Article	Extended-spectrum beta-lactamase (ESBL)-producing Escherichia coli survey in wild seabirds at a pristine atoll in the southern Atlantic Ocean, Brazil: First report of the O25b-ST131 clone harboring bla(CTX-M-8)	2022	12	Science of the Total Environment	806		amr--data--wild--extended-spectrum-beta-lactamase-(esbl)-producing-escherichia-coli-survey-in-wild-seabirds-at-a-pristine-atoll-in-the-southern-atlantic-ocean,-brazil:-first-report-of-the-o25b-st131-clone-harboring-bla(ctx-m-8)	
Journal Article	World Health Organization critical priority Escherichia coli clone ST648 in magnificent frigatebird (Fregata magnificens) of an uninhabited insular environment	2022	940600	Front Microbiol	13		amr--data--wild--world-health-organization-critical-priority-escherichia-coli-clone-st648-in-magnificent-frigatebird-(fregata-magnificens)-of-an-uninhabited-insular-environment	30/08/2022
Journal Article	Protective and detoxifying effects conferred by selenium against mycotoxins and livestock viruses: A review	2022	956814	Front Vet Sci	9		non-bio--opinion--protective-and-detoxifying-effects-conferred-by-selenium-against-mycotoxins-and-livestock-viruses:-a-review	20/08/2022
Journal Article	Sensitivity of an international notification system for wildlife diseases: A case study using the OIE-WAHIS data on tularemia	2022	286-294	Zoonoses Public Health	69	4	eval--opinion--pathogen--sensitivity-of-an-international-notification-system-for-wildlife-diseases:-a-case-study-using-the-oie-wahis-data-on-tularemia	30/01/2022
Journal Article	The successful use of -omic technologies to achieve the 'One Health' concept in meat producing animals	2022	108949	Meat Sci	193		opinion--the-successful-use-of--omic-technologies-to-achieve-the-'one-health'-concept-in-meat-producing-animals	28/08/2022
Journal Article	Co-creation and priority setting for applied and implementation research in One Health: Improving capacities in public and animal health systems in Kenya	2022	100460	One Health	15		data--policy--co-creation-and-priority-setting-for-applied-and-implementation-research-in-one-health:-improving-capacities-in-public-and-animal-health-systems-in-kenya	20/12/2022
Journal Article	A European-wide dataset to uncover adaptive traits of Listeria monocytogenes to diverse ecological niches	2022	12	Scientific Data	9	1	data--pathogen--a-european-wide-dataset-to-uncover-adaptive-traits-of-listeria-monocytogenes-to-diverse-ecological-niches	
Journal Article	Risk factors for antimicrobial resistance among Staphylococcus isolated from pets living with a patient diagnosed with methicillin-resistant Staphylococcus aureus infection	2022	550-559	Zoonoses Public Health	69	5	domestic--pathogen--risk--risk-factors-for-antimicrobial-resistance-among-staphylococcus-isolated-from-pets-living-with-a-patient-diagnosed-with-methicillin-resistant-staphylococcus-aureus-infection	15/04/2022
Journal Article	Sarcoptic mange outbreak decimates South American wild camelid populations in San Guillermo National Park, Argentina	2022	e0256616	PLoS One	17	1	data--pathogen--wild--sarcoptic-mange-outbreak-decimates-south-american-wild-camelid-populations-in-san-guillermo-national-park,-argentina	22/01/2022
Journal Article	Hepatitis E Virus Detection in Hunted Wild Boar Liver and Muscle Tissues in Central Italy	2022		Microorganisms	10	8	data--pathogen--wild--hepatitis-e-virus-detection-in-hunted-wild-boar-liver-and-muscle-tissues-in-central-italy	27/08/2022
Journal Article	Filarids (Spirurida: Onchocercidae) in wild carnivores and domestic dogs from the Brazilian Atlantic forest	2022	e0010213	PLoS Negl Trop Dis	16	3	data--domestic--pathogen--wild--filarids-(spirurida:-onchocercidae)-in-wild-carnivores-and-domestic-dogs-from-the-brazilian-atlantic-forest	09/03/2022
Journal Article	Risk-based control of Campylobacter spp. in broiler farms and slaughtered flocks to mitigate risk of human campylobacteriosis - A One Health approach	2022	8	Microbial Risk Analysis	21		domestic--pathogen--risk--risk-based-control-of-campylobacter-spp.-in-broiler-farms-and-slaughtered-flocks-to-mitigate-risk-of-human-campylobacteriosis---a-one-health-approach	
Journal Article	Toxicopathic effects of lithium in mussels	2022	136022	Chemosphere	307	Pt 4	data--non-bio--wild--toxicopathic-effects-of-lithium-in-mussels	25/08/2022
Journal Article	E-WASTE threatens health: The scientific solution adopts the one health strategy	2022	113227	Environ Res	212	Pt A	opinion--e-waste-threatens-health:-the-scientific-solution-adopts-the-one-health-strategy	05/04/2022
Journal Article	Antimicrobial Resistance Through the Lens of One Health in Ethiopia: A Review of the Literature Among Humans, Animals, and the Environment	2022	120-129	Int J Infect Dis	119		amr--domestic--env--human--opinion--antimicrobial-resistance-through-the-lens-of-one-health-in-ethiopia:-a-review-of-the-literature-among-humans,-animals,-and-the-environment	01/04/2022
Journal Article	Understanding Community Perceptions of the St. Kitts' "Monkey Problem" by Adapting Harm Reduction Concepts and Methods	2022	18	Frontiers in Ecology and Evolution	10		policy--wild--understanding-community-perceptions-of-the-st.-kitts'-"monkey-problem"-by-adapting-harm-reduction-concepts-and-methods	
Journal Article	A One Health Evaluation of the Surveillance Systems on Tick-Borne Diseases in the Netherlands, Spain and Italy	2022		Vet Sci	9	9	eval--pathogen--a-one-health-evaluation-of-the-surveillance-systems-on-tick-borne-diseases-in-the-netherlands,-spain-and-italy	23/09/2022
Journal Article	A generalizable one health framework for the control of zoonotic diseases	2022	8588	Sci Rep	12	1	eval--pathogen--policy--a-generalizable-one-health-framework-for-the-control-of-zoonotic-diseases	22/05/2022

Journal Article	The one health approach: Main psychological components	2022	11	Ricerche Di Psicologia	45	3	opinion--the-one-health-approach:-main-psychological-components	
Journal Article	Stunting among children aged 24-59 months and associations with sanitation, enteric infections, and environmental enteric dysfunction in rural northwest Ethiopia	2022	19293	Sci Rep	12	1	data--human--stunting-among-children-aged-24-59-months-and-associations-with-sanitation,-enteric-infections,-and-environmental-enteric-dysfunction-in-rural-northwest-ethiopia	13/11/2022
Journal Article	Co-Radiation of Leptospira and Tenrecidae (Afrotheria) on Madagascar	2022		Trop Med Infect Dis	7	8	data--pathogen--wild--co-radiation-of-leptospira-and-tenrecidae-(afrotheria)-on-madagascar	26/08/2022
Journal Article	Seasonal fishery facilitates a novel transmission pathway in an emerging animal reservoir of Guinea worm	2022	775-782.e4	Curr Biol	32	4	data--domestic--pathogen--wild--seasonal-fishery-facilitates-a-novel-transmission-pathway-in-an-emerging-animal-reservoir-of-guinea-worm	16/12/2021
Journal Article	Different profiles and epidemiological scenarios: past, present and future	2022	e200409	Mem Inst Oswaldo Cruz	117		opinion--pathogen--different-profiles-and-epidemiological-scenarios:-past,-present-and-future	26/05/2022
Journal Article	Factors promoting and limiting antimicrobial resistance in the environment - Existing knowledge gaps	2022	8	Frontiers in Microbiology	13		amr--opinion--factors-promoting-and-limiting-antimicrobial-resistance-in-the-environment---existing-knowledge-gaps	
Journal Article	Genomic Diversity of Campylobacter lari Group Isolates from Europe and Australia in a One Health Context	2022	e0136822	Appl Environ Microbiol	88	23	data--domestic--env--pathogen--wild--genomic-diversity-of-campylobacter-lari-group-isolates-from-europe-and-australia-in-a-one-health-context	11/11/2022
Journal Article	Occurrence of Antimicrobial-Resistant Escherichia coli in Marine Mammals of the North and Baltic Seas: Sentinels for Human Health	2022		Antibiotics (Basel)	11	9	amr--data--wild--occurrence-of-antimicrobial-resistant-escherichia-coli-in-marine-mammals-of-the-north-and-baltic-seas:-sentinels-for-human-health	24/09/2022
Journal Article	Clonal and plasmid-mediated dissemination of environmental carbapenem-resistant Enterobacteriaceae in large animal breeding areas in northern China	2022	118800	Environ Pollut	297		amr--data--env--wild--clonal-and-plasmid-mediated-dissemination-of-environmental-carbapenem-resistant-enterobacteriaceae-in-large-animal-breeding-areas-in-northern-china	11/01/2022
Journal Article	A participatory epidemiological and One Health approach to explore the community's capacity to detect emerging zoonoses and surveillance network opportunities in the forest region of Guinea	2022	e0010462	PLoS Negl Trop Dis	16	7	data--human--pathogen--a-participatory-epidemiological-and-one-health-approach-to-explore-the-community's-capacity-to-detect-emerging-zoonoses-and-surveillance-network-opportunities-in-the-forest-region-of-guinea	12/07/2022
Journal Article	Serological evidence of exposure of Bornean wild carnivores to feline-related viruses at the domestic animal-wildlife interface	2022	e3250-e3254	Transbound Emerg Dis	69	5	data--pathogen--wild--serological-evidence-of-exposure-of-bornean-wild-carnivores-to-feline-related-viruses-at-the-domestic-animal-wildlife-interface	05/04/2022
Journal Article	Identification of Leishmania infantum and Leishmania braziliensis in captive primates from a zoo in Brazil	2022	e23376	Am J Primatol	84	4-5	data--pathogen--wild--identification-of-leishmania-infantum-and-leishmania-braziliensis-in-captive-primates-from-a-zoo-in-brazil	07/04/2022
Journal Article	Different fosA genes were found on mobile genetic elements in Escherichia coli from wastewaters of hospitals and municipals in Turkey	2022	9	Science of the Total Environment	824		amr--data--env--different-fosa-genes-were-found-on-mobile-genetic-elements-in-escherichia-coli-from-wastewaters-of-hospitals-and-municipals-in-turkey	
Journal Article	Environmental factors associated with the prevalence of ESBL/AmpC-producing Escherichia coli in wild boar (Sus scrofa)	2022	13	Frontiers in Veterinary Science	9		amr--data--domestic--env--human--risk--wild--environmental-factors-associated-with-the-prevalence-of-esbl/ampc-producing-escherichia-coli-in-wild-boar-(sus-scrofa)	
Journal Article	Environmental contamination in a high-income country (France) by antibiotics, antibiotic-resistant bacteria, and antibiotic resistance genes: Status and possible causes	2022	107047	Environ Int	159		amr--env--risk--environmental-contamination-in-a-high-income-country-(france)-by-antibiotics,-antibiotic-resistant-bacteria,-and-antibiotic-resistance-genes:-status-and-possible-causes	20/12/2021
Journal Article	SARS-CoV-2 infection in free-ranging white-tailed deer	2022	481-486	Nature	602	7897	covid--data--wild--sars-cov-2-infection-in-free-ranging-white-tailed-deer	24/12/2021
Journal Article	One Health Probiotics as Biocontrol Agents: One Health Tomato Probiotics	2022		Plants (Basel)	11	10	opinion--pathogen--one-health-probiotics-as-biocontrol-agents:-one-health-tomato-probiotics	29/05/2022
Journal Article	Assembling antimicrobial resistance governance in UK animal agriculture	2022	587-610	Sociologia Ruralis	62	3	amr--opinion--assembling-antimicrobial-resistance-governance-in-uk-animal-agriculture	
Journal Article	Cryptosporidiosis: From Prevention to Treatment, a Narrative Review	2022		Microorganisms	10	12	opinion--pathogen--cryptosporidiosis:-from-prevention-to-treatment,-a-narrative-review	24/12/2022
Journal Article	Longitudinal surveillance and comparative characterization of Escherichia albertii in wild raccoons in the United States	2022	127109	Microbiol Res	262		data--pathogen--wild--longitudinal-surveillance-and-comparative-characterization-of-escherichia-albertii-in-wild-raccoons-in-the-united-states	09/07/2022
Journal Article	Operationalizing "One Health" as "One Digital Health" Through a Global Framework That Emphasizes Fair and Equitable Sharing of Benefits From the Use of Artificial Intelligence and Related Digital Technologies	2022	768977	Front Public Health	10		opinion--policy--operationalizing-"one-health"-as-"one-digital-health"-through-a-global-framework-that-emphasizes-fair-and-equitable-sharing-of-benefits-from-the-use-of-artificial-intelligence-and-related-digital-technologies	21/05/2022
Journal Article	A one health approach to plant health	2022	7	Cabi Agriculture & Bioscience	3	1	opinion--a-one-health-approach-to-plant-health	
Journal Article	Elimination of Schistosomiasis Japonica in China: From the One Health Perspective	2022	130-134	China CDC Wkly	4	7	opinion--pathogen--elimination-of-schistosomiasis-japonica-in-china:-from-the-one-health-perspective	11/03/2022
Journal Article	Geographic Range Overlap Rather than Phylogenetic Distance Explains Rabies Virus Transmission among Closely Related Bat Species	2022		Viruses	14	11	data--pathogen--wild--geographic-range-overlap-rather-than-phylogenetic-distance-explains-rabies-virus-transmission-among-closely-related-bat-species	12/11/2022
Journal Article	From gut to mud: dissemination of antimicrobial resistance between animal and agricultural niches	2022	3290-3306	Environ Microbiol	24	8	amr--opinion--from-gut-to-mud:-dissemination-of-antimicrobial-resistance-between-animal-and-agricultural-niches	17/02/2022
Journal Article	First cases of SARS-CoV-2 infection in dogs and cats in Thailand	2022	e979-e991	Transbound Emerg Dis	69	4	covid--data--domestic--first-cases-of-sars-cov-2-infection-in-dogs-and-cats-in-thailand	06/11/2021
Journal Article	The Origin of Internal Genes Contributes to the Replication and Transmission Fitness of H7N9 Avian Influenza Virus	2022	19	Journal of Virology	96	22	data--domestic--pathogen--the-origin-of-internal-genes-contributes-to-the-replication-and-transmission-fitness-of-h7n9-avian-influenza-virus	
Journal Article	Quantifying risk factors and potential geographic extent of African swine fever across the world	2022	e0267128	PLoS One	17	4	domestic--opinion--pathogen--wild--quantifying-risk-factors-and-potential-geographic-extent-of-african-swine-fever-across-the-world	22/04/2022
Journal Article	A systematic review and meta-analysis of the prevalence of Listeria monocytogenes in South-East Asia; a one-health approach of human-animal-food-environment	2022	100417	One Health	15		data--domestic--env--pathogen--risk--a-systematic-review-and-meta-analysis-of-the-prevalence-of-listeria-monocytogenes-in-south-east-asia;-a-one-health-approach-of-human-animal-food-environment	25/10/2022
Journal Article	The impact of the antibiotic resistance on the sustainable development	2022	22	Poblacion Y Salud En Mesoamerica	19	2	amr--opinion--the-impact-of-the-antibiotic-resistance-on-the-sustainable-development	
Journal Article	Airborne transmission as an integral environmental dimension of antimicrobial resistance through the "One Health" lens	2022	4172-4193	Critical Reviews in Environmental Science and Technology	52	23	amr--opinion--airborne-transmission-as-an-integral-environmental-dimension-of-antimicrobial-resistance-through-the-"one-health"-lens	
Journal Article	One Health Approach to Tick and Tick-Borne Disease Surveillance in the United Kingdom	2022	12	International Journal of Environmental Research and Public Health	19	10	eval--opinion--pathogen--one-health-approach-to-tick-and-tick-borne-disease-surveillance-in-the-united-kingdom	
Journal Article	Antimicrobial Resistance and Environmental Health: A Water Stewardship Framework for Global and National Action	2022		Antibiotics (Basel)	11	1	amr--eval--antimicrobial-resistance-and-environmental-health:-a-water-stewardship-framework-for-global-and-national-action	22/01/2022
Journal Article	Knowledge, Attitudes, and Common Practices of Livestock and Poultry Veterinary Practitioners Regarding the AMU and AMR in Bangladesh	2022		Antibiotics (Basel)	11	1	amr--human--policy--knowledge,-attitudes,-and-common-practices-of-livestock-and-poultry-veterinary-practitioners-regarding-the-amu-and-amr-in-bangladesh	22/01/2022
Journal Article	Gaps and opportunities in sustainable medicines use in resource limited settings: A situational analysis of Uganda	2022	3936-3942	Br J Clin Pharmacol	88	9	opinion--policy--gaps-and-opportunities-in-sustainable-medicines-use-in-resource-limited-settings:-a-situational-analysis-of-uganda	29/03/2022
Journal Article	Systematic Review and Meta-Analysis on Knowledge Attitude and Practices on African Animal Trypanocide Resistance	2022		Trop Med Infect Dis	7	9	data--human--pathogen--systematic-review-and-meta-analysis-on-knowledge-attitude-and-practices-on-african-animal-trypanocide-resistance	23/09/2022
Journal Article	Analysis of policies for use of medically important antibiotics in animals in Namibia: implications for antimicrobial stewardship	2022	1365-1379	Expert Review of Anti-Infective Therapy	20	10	amr--eval--data--policy--analysis-of-policies-for-use-of-medically-important-antibiotics-in-animals-in-namibia:-implications-for-antimicrobial-stewardship	
Journal Article	The golden jackal (Canis aureus) and the African swine fever pandemic: Its role is controversial but not negligible (a diet analysis study)	2022	97-103	Veterinary Medicine and Science	8	1	data--pathogen--wild--the-golden-jackal-(canis-aureus)-and-the-african-swine-fever-pandemic:-its-role-is-controversial-but-not-negligible-(a-diet-analysis-study)	
Journal Article	A community-based survey to assess risk for one health challenges in rural Philippines using a mobile application	2022	7	One Health Outlook	4	1	data--human--pathogen--a-community-based-survey-to-assess-risk-for-one-health-challenges-in-rural-philippines-using-a-mobile-application	06/04/2022
Journal Article	Veterinarians as a Risk Group for Zoonoses: Exposure, Knowledge and Protective Practices in Finland	2022	78-85	Safety and Health at Work	13	1	data--human--pathogen--veterinarians-as-a-risk-group-for-zoonoses:-exposure,-knowledge-and-protective-practices-in-finland	
Journal Article	Enhancing inter-organizational collaboration for wildlife disease surveillance in Sri Lanka	2022	792-805	Zoonoses Public Health	69	7	data--eval--human--enhancing-inter-organizational-collaboration-for-wildlife-disease-surveillance-in-sri-lanka	23/05/2022
Journal Article	Cancer Incidence, Mortality, Years of Life Lost, Years Lived With Disability, and Disability-Adjusted Life Years for 29 Cancer Groups From 2010 to 2019: A Systematic Analysis for the Global Burden of Disease Study 2019	2022	420-444	JAMA Oncol	8	3	cancer--data--human--risk--cancer-incidence,-mortality,-years-of-life-lost,-years-lived-with-disability,-and-disability-adjusted-life-years-for-29-cancer-groups-from-2010-to-2019:-a-systematic-analysis-for-the-global-burden-of-disease-study-2019	31/12/2021
Journal Article	Simian adenoviruses: Molecular and serological survey in monkeys and humans in Thailand	2022	100434	One Health	15		data--pathogen--wild--simian-adenoviruses:-molecular-and-serological-survey-in-monkeys-and-humans-in-thailand	25/10/2022
Journal Article	Applications of Probiotic-Based Multi-Components to Human, Animal and Ecosystem Health: Concepts, Methodologies, and Action Mechanisms	2022		Microorganisms	10	9	opinion--pathogen--applications-of-probiotic-based-multi-components-to-human,-animal-and-ecosystem-health:-concepts,-methodologies,-and-action-mechanisms	24/09/2022
Journal Article	Mitigating the Impact of Emerging Animal Infectious Disease Threats: First Emerging Animal Infectious Diseases Conference (EAIDC) Report	2022		Viruses	14	5	eval--pathogen--mitigating-the-impact-of-emerging-animal-infectious-disease-threats:-first-emerging-animal-infectious-diseases-conference-(eaidc)-report	29/05/2022
Journal Article	The host-specific resistome in environmental feces of Eurasian otters (Lutra lutra) and leopard cats (Prionailurus bengalensis) revealed by metagenomic sequencing	2022	100385	One Health	14		amr--data--wild--the-host-specific-resistome-in-environmental-feces-of-eurasian-otters-(lutra-lutra)-and-leopard-cats-(prionailurus-bengalensis)-revealed-by-metagenomic-sequencing	12/04/2022
Journal Article	Beehive products as bioindicators of antimicrobial resistance contamination in the environment	2022	151131	Sci Total Environ	823		amr--data--wild--beehive-products-as-bioindicators-of-antimicrobial-resistance-contamination-in-the-environment	26/10/2021
Journal Article	A multi-jurisdictional outbreak of Salmonella Typhimurium infections linked to backyard poultry-Australia, 2020	2022	835-842	Zoonoses Public Health	69	7	data--domestic--human--pathogen--a-multi-jurisdictional-outbreak-of-salmonella-typhimurium-infections-linked-to-backyard-poultry-australia,-2020	06/07/2022
Journal Article	Factors impacting antimicrobial resistance in the South East Asian food system and potential places to intervene: A participatory, one health study	2022	992507	Front Microbiol	13		data--human--policy--factors-impacting-antimicrobial-resistance-in-the-south-east-asian-food-system-and-potential-places-to-intervene:-a-participatory,-one-health-study	24/01/2023
Journal Article	Coronavirus and Paramyxovirus Shedding by Bats in a Cave and Buildings in Ethiopia	2022	216-232	Ecohealth	19	2	data--pathogen--wild--coronavirus-and-paramyxovirus-shedding-by-bats-in-a-cave-and-buildings-in-ethiopia	01/07/2022
Journal Article	Emergence of methicillin resistance predates the clinical use of antibiotics	2022	135-141	Nature	602	7895	amr--data--wild--emergence-of-methicillin-resistance-predates-the-clinical-use-of-antibiotics	07/01/2022
Journal Article	Human and animal botulism surveillance in France from 2008 to 2019	2022	1003917	Front Public Health	10		domestic--human--opinion--pathogen--human-and-animal-botulism-surveillance-in-france-from-2008-to-2019	13/12/2022
Journal Article	Bio-Ethics and One Health: A Case Study Approach to Building Reflexive Governance	2022	19	Frontiers in Public Health	10		human--opinion--policy--bio-ethics-and-one-health:-a-case-study-approach-to-building-reflexive-governance	
Journal Article	The Role of the Environment in Dynamics of Antibiotic Resistance in Humans and Animals: A Modelling Study	2022		Antibiotics (Basel)	11	10	amr--domestic--env--human--risk--the-role-of-the-environment-in-dynamics-of-antibiotic-resistance-in-humans-and-animals:-a-modelling-study	28/10/2022
Journal Article	Comparative Analysis of Phylogenetic Relationships and Virulence Factor Characteristics between Extended-Spectrum β-Lactamase-Producing Escherichia coli Isolates Derived from Clinical Sites and Chicken Farms	2022	e0255722	Microbiol Spectr	10	6	amr--data--domestic--human--comparative-analysis-of-phylogenetic-relationships-and-virulence-factor-characteristics-between-extended-spectrum-β-lactamase-producing-escherichia-coli-isolates-derived-from-clinical-sites-and-chicken-farms	15/11/2022
Journal Article	Consideration of COVID-19 beyond the human-centred approach of prevention and control: the ONE-HEALTH perspective	2022	2520-2528	Emerg Microbes Infect	11	1	covid--domestic--env--opinion--consideration-of-covid-19-beyond-the-human-centred-approach-of-prevention-and-control:-the-one-health-perspective	15/09/2022
Journal Article	Prototheca Infections and Ecology from a One Health Perspective	2022	18	Microorganisms	10	5	domestic--human--opinion--pathogen--prototheca-infections-and-ecology-from-a-one-health-perspective	
Journal Article	Prioritized antibiotics screening based on comprehensive risk assessments and related management strategy in various animal farms	2022	8	Journal of Environmental Management	319		amr--data--domestic--prioritized-antibiotics-screening-based-on-comprehensive-risk-assessments-and-related-management-strategy-in-various-animal-farms	
Journal Article	Alternative Paradigms in Animal Health Decisions: A Framework for Treating Animals Not Only as Commodities	2022		Animals (Basel)	12	14	domestic--human--pathogen--policy--risk--alternative-paradigms-in-animal-health-decisions:-a-framework-for-treating-animals-not-only-as-commodities	28/07/2022
Journal Article	Risk assessment of SARS-CoV-2 infection in free-ranging wild animals in Belgium	2022	986-996	Transbound Emerg Dis	69	3	covid--opinion--wild--risk-assessment-of-sars-cov-2-infection-in-free-ranging-wild-animals-in-belgium	29/04/2021
Journal Article	Molecular survey of Zika virus in the animal-human interface in traditional farming	2022	1057686	Front Vet Sci	9		data--domestic--pathogen--wild--molecular-survey-of-zika-virus-in-the-animal-human-interface-in-traditional-farming	13/12/2022
Journal Article	A Qualitative Study on the Design and Implementation of the National Action Plan on Antimicrobial Resistance in the Philippines	2022		Antibiotics (Basel)	11	6	amr--eval--human--policy--a-qualitative-study-on-the-design-and-implementation-of-the-national-action-plan-on-antimicrobial-resistance-in-the-philippines	25/06/2022
Journal Article	Determinants for antimicrobial resistance genes in farm dust on 333 poultry and pig farms in nine European countries	2022	112715	Environ Res	208		amr--data--domestic--risk--determinants-for-antimicrobial-resistance-genes-in-farm-dust-on-333-poultry-and-pig-farms-in-nine-european-countries	17/01/2022
Journal Article	Acacia Fiber Protects the Gut from Extended-Spectrum Beta-Lactamase (ESBL)-Producing Escherichia coli Colonization Enabled by Antibiotics	2022	e0007122	mSphere	7	3	amr--data--human--acacia-fiber-protects-the-gut-from-extended-spectrum-beta-lactamase-(esbl)-producing-escherichia-coli-colonization-enabled-by-antibiotics	19/05/2022
Journal Article	Strengthening laboratory biosafety in Liberia during the COVID-19 pandemic: Experience from the Global Laboratory Leadership Programme	2022	100442	One Health	15		covid--edu--eval--human--strengthening-laboratory-biosafety-in-liberia-during-the-covid-19-pandemic:-experience-from-the-global-laboratory-leadership-programme	18/10/2022
Journal Article	Risk-Benefit Assessment of Feed Additives in the One Health Perspective	2022	843124	Front Nutr	9		domestic--opinion--pathogen--risk--risk-benefit-assessment-of-feed-additives-in-the-one-health-perspective	01/03/2022
Journal Article	One Health surveillance approaches for melioidosis and glanders: The Malaysian perspective	2022	12	Frontiers in Veterinary Science	9		domestic--eval--human--opinion--pathogen--one-health-surveillance-approaches-for-melioidosis-and-glanders:-the-malaysian-perspective	
Journal Article	Antimicrobial Resistance in Escherichia coli from the Broiler Farm Environment, with Detection of SHV-12-Producing Isolates	2022	14	Antibiotics-Basel	11	4	amr--data--domestic--antimicrobial-resistance-in-escherichia-coli-from-the-broiler-farm-environment,-with-detection-of-shv-12-producing-isolates	
Journal Article	Urban Birds as Antimicrobial Resistance Sentinels: White Storks Showed Higher Multidrug-Resistant Escherichia coli Levels Than Seagulls in Central Spain	2022	11	Animals	12	19	amr--data--wild--urban-birds-as-antimicrobial-resistance-sentinels:-white-storks-showed-higher-multidrug-resistant-escherichia-coli-levels-than-seagulls-in-central-spain	
Journal Article	From the Intersection of Food-Borne Zoonoses and EU Green Policies to an In-Embryo One Health Financial Model	2022		Foods	11	18	opinion--policy--from-the-intersection-of-food-borne-zoonoses-and-eu-green-policies-to-an-in-embryo-one-health-financial-model	24/09/2022
Journal Article	The Landscape of Participatory Surveillance Systems Across the One Health Spectrum: Systematic Review	2022	12	Jmir Public Health and Surveillance	8	8	data--edu--human--policy--the-landscape-of-participatory-surveillance-systems-across-the-one-health-spectrum:-systematic-review	
Journal Article	Role of regulatory capacity in the animal and human health systems in driving response to zoonotic disease outbreaks in the the Mekong region	2022	100369	One Health	14		eval--human--policy--role-of-regulatory-capacity-in-the-animal-and-human-health-systems-in-driving-response-to-zoonotic-disease-outbreaks-in-the-the-mekong-region	03/02/2022
Journal Article	State of Knowledge on the Acquisition, Diversity, Interspecies Attribution and Spread of Antimicrobial Resistance between Humans, Animals and the Environment: A Systematic Review	2022		Antibiotics (Basel)	12	1	amr--data--eval--state-of-knowledge-on-the-acquisition,-diversity,-interspecies-attribution-and-spread-of-antimicrobial-resistance-between-humans,-animals-and-the-environment:-a-systematic-review	22/01/2023
Journal Article	Multidrug-Resistant Bacteria Isolated From Surgical Site of Dogs, Surgeon's Hands and Operating Room in a Veterinary Teaching Hospital in Brazil	2022	6	Topics in Companion Animal Medicine	49		amr--data--human--multidrug-resistant-bacteria-isolated-from-surgical-site-of-dogs,-surgeon's-hands-and-operating-room-in-a-veterinary-teaching-hospital-in-brazil	
Journal Article	Safety of Retailed Poultry: Analysis of Antibiotic Resistance in Escherichia coli From Raw Chicken and Poultry Fecal Matter From Selected Farms and Retail Outlets in Accra, Ghana	2022	11786361221093278	Microbiol Insights	15		amr--data--domestic--safety-of-retailed-poultry:-analysis-of-antibiotic-resistance-in-escherichia-coli-from-raw-chicken-and-poultry-fecal-matter-from-selected-farms-and-retail-outlets-in-accra,-ghana	07/05/2022
Journal Article	Risk and asset-based strategies in health: priorities in biomedical, life and environmental science literature since the early twentieth century. A rapid review	2022	12	Environmental Health	21	1	covid--data--eval--risk-and-asset-based-strategies-in-health:-priorities-in-biomedical,-life-and-environmental-science-literature-since-the-early-twentieth-century.-a-rapid-review	
Journal Article	Crimean-Congo Hemorrhagic Fever Virus Survey in Humans, Ticks, and Livestock in Agnam (Northeastern Senegal) from February 2021 to March 2022	2022		Trop Med Infect Dis	7	10	climate--data--domestic--human--pathogen--crimean-congo-hemorrhagic-fever-virus-survey-in-humans,-ticks,-and-livestock-in-agnam-(northeastern-senegal)-from-february-2021-to-march-2022	27/10/2022
Journal Article	Antimicrobial Use and Resistance in Plant Agriculture: A One Health Perspective	2022	27	Agriculture-Basel	12	2	amr--opinion--antimicrobial-use-and-resistance-in-plant-agriculture:-a-one-health-perspective	
Journal Article	Comparison of environmental microbiomes in an antibiotic resistance-polluted urban river highlights periphyton and fish gut communities as reservoirs of concern	2022	158042	Sci Total Environ	851	Pt 1	amr--data--env--wild--comparison-of-environmental-microbiomes-in-an-antibiotic-resistance-polluted-urban-river-highlights-periphyton-and-fish-gut-communities-as-reservoirs-of-concern	17/08/2022
Journal Article	SARS-CoV-2 in Namibian Dogs	2022	5	Vaccines	10	12	covid--data--domestic--sars-cov-2-in-namibian-dogs	
Journal Article	One Health compartmental analysis of ESBL-producing Escherichia coli on Reunion Island reveals partitioning between humans and livestock	2022	1254-1262	Journal of Antimicrobial Chemotherapy	77	5	amr--data--domestic--human--wild--one-health-compartmental-analysis-of-esbl-producing-escherichia-coli-on-reunion-island-reveals-partitioning-between-humans-and-livestock	
Journal Article	Clustered Regularly Interspaced Short Palindromic Repeats Genotyping of Multidrug-Resistant Salmonella Heidelberg Strains Isolated From the Poultry Production Chain Across Brazil	2022	867278	Front Microbiol	13		amr--data--domestic--clustered-regularly-interspaced-short-palindromic-repeats-genotyping-of-multidrug-resistant-salmonella-heidelberg-strains-isolated-from-the-poultry-production-chain-across-brazil	06/07/2022
Journal Article	Screening for hepatitis E virus genotype 3 in red deer (Cervus elaphus) and fallow deer (Dama dama), Portugal, 2018-2020	2022	2764-2768	Transbound Emerg Dis	69	5	data--pathogen--wild--screening-for-hepatitis-e-virus-genotype-3-in-red-deer-(cervus-elaphus)-and-fallow-deer-(dama-dama),-portugal,-2018-2020	17/12/2021
Journal Article	Reproductive one health in primates	2022	25	American Journal of Primatology	84	4-5	opinion--wild--reproductive-one-health-in-primates	
Journal Article	A statistical modelling approach for source attribution meta-analysis of sporadic infection with foodborne pathogens	2022	475-486	Zoonoses and Public Health	69	5	domestic--human--pathogen--risk--a-statistical-modelling-approach-for-source-attribution-meta-analysis-of-sporadic-infection-with-foodborne-pathogens	
Journal Article	Evolution and expansion of the One Health approach to promote sustainable and resilient health and well-being: A call to action	2022	1056459	Front Public Health	10		opinion--policy--evolution-and-expansion-of-the-one-health-approach-to-promote-sustainable-and-resilient-health-and-well-being:-a-call-to-action	31/01/2023
Journal Article	Genomic epidemiology of Escherichia coli: antimicrobial resistance through a One Health lens in sympatric humans, livestock and peri-domestic wildlife in Nairobi, Kenya	2022	11	Bmc Medicine	20	1	amr--data--domestic--risk--genomic-epidemiology-of-escherichia-coli:-antimicrobial-resistance-through-a-one-health-lens-in-sympatric-humans,-livestock-and-peri-domestic-wildlife-in-nairobi,-kenya	
Journal Article	Institutional one health and animal-human health connections in Nthongoni, Eastern Kenya	2022	102818	Health Place	77		opinion--institutional-one-health-and-animal-human-health-connections-in-nthongoni,-eastern-kenya	14/05/2022
Journal Article	Sero-epidemiological survey of Coxiella burnetii in livestock and humans in Tana river and garissa counties in Kenya	2022	19	Plos Neglected Tropical Diseases	16	3	data--human--pathogen--risk--sero-epidemiological-survey-of-coxiella-burnetii-in-livestock-and-humans-in-tana-river-and-garissa-counties-in-kenya	
Journal Article	Where Rabies Is Not a Disease. Bridging Healthworlds to Improve Mutual Understanding and Prevention of Rabies	2022	867266	Front Vet Sci	9		domestic--eval--human--pathogen--where-rabies-is-not-a-disease.-bridging-healthworlds-to-improve-mutual-understanding-and-prevention-of-rabies	06/07/2022
Journal Article	Crossing artificial obstacles during migration: The relative global ecological risks and interdependencies illustrated by the migration of common quail Coturnix coturnix	2022	152173	Sci Total Environ	808		data--env--human--wild--crossing-artificial-obstacles-during-migration:-the-relative-global-ecological-risks-and-interdependencies-illustrated-by-the-migration-of-common-quail-coturnix-coturnix	08/12/2021
Journal Article	Narrative review of lead poisoning in humans caused by industrial activities and measures compatible with sustainable industrial activities in Republic of Zambia	2022	157833	Sci Total Environ	850		human--non-bio--opinion--narrative-review-of-lead-poisoning-in-humans-caused-by-industrial-activities-and-measures-compatible-with-sustainable-industrial-activities-in-republic-of-zambia	13/08/2022
Journal Article	The potential distribution of Bacillus anthracis suitability across Uganda using INLA	2022	19967	Sci Rep	12	1	env--eval--human--pathogen--risk--the-potential-distribution-of-bacillus-anthracis-suitability-across-uganda-using-inla	20/11/2022
Journal Article	Herd turnover rate reexamined: a tool for improving profitability, welfare, and sustainability	2022		Am J Vet Res	84	1	domestic--eval--risk--herd-turnover-rate-reexamined:-a-tool-for-improving-profitability,-welfare,-and-sustainability	06/12/2022
Journal Article	Dairy production sustainability through a one-health lens	2022	12-16	J Am Vet Med Assoc	261	1	domestic--opinion--dairy-production-sustainability-through-a-one-health-lens	15/11/2022
Journal Article	Decades of emerging infectious disease, food safety, and antimicrobial resistance response in Vietnam: The role of One Health	2022	100361	One Health	14		edu--eval--pathogen--decades-of-emerging-infectious-disease,-food-safety,-and-antimicrobial-resistance-response-in-vietnam:-the-role-of-one-health	21/12/2021
Journal Article	Outbreak of Multidrug-Resistant Salmonella Heidelberg Infections Linked to Dairy Calf Exposure, United States, 2015-2018	2022	199-208	Foodborne Pathogens and Disease	19	3	data--domestic--human--pathogen--outbreak-of-multidrug-resistant-salmonella-heidelberg-infections-linked-to-dairy-calf-exposure,-united-states,-2015-2018	
Journal Article	Health impact assessment of pet cats caused by organohalogen contaminants by serum metabolomics and thyroid hormone analysis	2022	156490	Sci Total Environ	842		data--domestic--non-bio--health-impact-assessment-of-pet-cats-caused-by-organohalogen-contaminants-by-serum-metabolomics-and-thyroid-hormone-analysis	07/06/2022
Journal Article	Electronic information systems for One Health surveillance of antimicrobial resistance: a systematic scoping review	2022		BMJ Glob Health	7	1	amr--data--eval--policy--electronic-information-systems-for-one-health-surveillance-of-antimicrobial-resistance:-a-systematic-scoping-review	06/01/2022
Journal Article	One Health risk challenges and preparedness regarding bovine tuberculosis at abattoirs in North-central Nigeria: Associated drivers and health belief	2022	e0010729	PLoS Negl Trop Dis	16	9	eval--human--pathogen--one-health-risk-challenges-and-preparedness-regarding-bovine-tuberculosis-at-abattoirs-in-north-central-nigeria:-associated-drivers-and-health-belief	07/09/2022
Journal Article	Role of landscape context in Toxoplasma gondii infection of invasive definitive and intermediate hosts on a World Heritage Island	2022	96-104	Int J Parasitol Parasites Wildl	19		data--pathogen--wild--role-of-landscape-context-in-toxoplasma-gondii-infection-of-invasive-definitive-and-intermediate-hosts-on-a-world-heritage-island	16/09/2022
Journal Article	Molecular characterization of extended spectrum cephalosporin resistant Escherichia coli isolated from livestock and in-contact humans in Southeast Nigeria	2022	937968	Front Microbiol	13		amr--data--domestic--human--molecular-characterization-of-extended-spectrum-cephalosporin-resistant-escherichia-coli-isolated-from-livestock-and-in-contact-humans-in-southeast-nigeria	09/08/2022
Journal Article	Circulation of Ngari Virus in Livestock, Kenya	2022	e0041622	mSphere	7	6	data--domestic--pathogen--wild--circulation-of-ngari-virus-in-livestock,-kenya	07/12/2022
Journal Article	Toxoplasma gondii Infections in Animals and Humans in Southern Africa: A Systematic Review and Meta-Analysis	2022		Pathogens	11	2	eval--pathogen--toxoplasma-gondii-infections-in-animals-and-humans-in-southern-africa:-a-systematic-review-and-meta-analysis	27/02/2022
Journal Article	Leptospirosis is an emerging infectious disease of pig-hunting dogs and humans in North Queensland	2022	e0010100	PLoS Negl Trop Dis	16	1	data--domestic--pathogen--leptospirosis-is-an-emerging-infectious-disease-of-pig-hunting-dogs-and-humans-in-north-queensland	19/01/2022
Journal Article	Impact of Cumulative Environmental and Dietary Xenobiotics on Human Microbiota: Risk Assessment for One Health	2022	56-63	Journal of Xenobiotics	12	1	non-bio--opinion--risk--impact-of-cumulative-environmental-and-dietary-xenobiotics-on-human-microbiota:-risk-assessment-for-one-health	
Journal Article	From Farm to Fork: Streptococcus suis as a Model for the Development of Novel Phage-Based Biocontrol Agents	2022	27	Viruses-Basel	14	9	domestic--opinion--pathogen--from-farm-to-fork:-streptococcus-suis-as-a-model-for-the-development-of-novel-phage-based-biocontrol-agents	
Journal Article	A rich mosaic of resistance in extended-spectrum beta-lactamase-producing Escherichia coli isolated from red foxes (Vulpes vulpes) in Poland as a potential effect of increasing synanthropization	2022	11	Science of the Total Environment	818		amr--data--wild--a-rich-mosaic-of-resistance-in-extended-spectrum-beta-lactamase-producing-escherichia-coli-isolated-from-red-foxes-(vulpes-vulpes)-in-poland-as-a-potential-effect-of-increasing-synanthropization	
Journal Article	Antimicrobial resistance among canine enterococci in the northeastern United States, 2007-2020	2022	1025242	Front Microbiol	13		amr--data--domestic--antimicrobial-resistance-among-canine-enterococci-in-the-northeastern-united-states,-2007-2020	24/01/2023
Journal Article	Catch-22: War, Refugees, COVID-19, and the Scourge of Antimicrobial Resistance	2022	921921	Front Med (Lausanne)	9		amr--opinion--catch-22:-war,-refugees,-covid-19,-and-the-scourge-of-antimicrobial-resistance	12/07/2022
Journal Article	Integrated surveillance of antimicrobial resistance and antimicrobial use: Evaluation of the status in Canada (2014-2019)	2022	161-170	Can Vet J	63	2	eval--pathogen--policy--integrated-surveillance-of-antimicrobial-resistance-and-antimicrobial-use:-evaluation-of-the-status-in-canada-(2014-2019)	04/02/2022
Journal Article	Research Themes, Trends and Future Priorities in the Field of Climate Change and Health: A Review	2022	20	Atmosphere	13	12	climate--data--policy--research-themes,-trends-and-future-priorities-in-the-field-of-climate-change-and-health:-a-review	
Journal Article	Impact of physicochemical parameters of Aedes aegypti breeding habitats on mosquito productivity and the size of emerged adult mosquitoes in Ouagadougou City, Burkina Faso	2022	478	Parasit Vectors	15	1	data--pathogen--wild--impact-of-physicochemical-parameters-of-aedes-aegypti-breeding-habitats-on-mosquito-productivity-and-the-size-of-emerged-adult-mosquitoes-in-ouagadougou-city,-burkina-faso	21/12/2022
Journal Article	Epidemiological Profile of a Human Hepatitis E Virus Outbreak in 2018, Chattogram, Bangladesh	2022		Trop Med Infect Dis	7	8	env--human--pathogen--risk--epidemiological-profile-of-a-human-hepatitis-e-virus-outbreak-in-2018,-chattogram,-bangladesh	26/08/2022
Journal Article	Embed Multisectoral Governance Mechanisms in the Pandemic Instrument for One Health Action	2022	71-81	J Law Med Ethics	50	S2	opinion--policy--embed-multisectoral-governance-mechanisms-in-the-pandemic-instrument-for-one-health-action	01/01/2022
Journal Article	Antimicrobial resistance surveillance system mapping in different countries	2022	36-48	Drug Target Insights	16		amr--data--policy--antimicrobial-resistance-surveillance-system-mapping-in-different-countries	
Journal Article	First report of three novel Bartonella species isolated in rodents and shrews from nine provinces of Thailand	2022	1624-1631	Vet World	15	7	data--pathogen--wild--first-report-of-three-novel-bartonella-species-isolated-in-rodents-and-shrews-from-nine-provinces-of-thailand	04/10/2022
Journal Article	Predicting the potential for zoonotic transmission and host associations for novel viruses	2022	844	Commun Biol	5	1	pathogen--risk--predicting-the-potential-for-zoonotic-transmission-and-host-associations-for-novel-viruses	20/08/2022
Journal Article	Influence of ecological and climatic conditions on the spread of Borrelia burgdorferi in domestic dogs in Ukraine	2022	431-442	Regulatory Mechanisms in Biosystems	13	4	data--domestic--pathogen--influence-of-ecological-and-climatic-conditions-on-the-spread-of-borrelia-burgdorferi-in-domestic-dogs-in-ukraine	
Journal Article	Addressing the urgent health challenges of climate change and ecosystem degradation from a One Health perspective: what can veterinarians contribute?	2022	49-55	J Am Vet Med Assoc	261	1	climate--opinion--addressing-the-urgent-health-challenges-of-climate-change-and-ecosystem-degradation-from-a-one-health-perspective:-what-can-veterinarians-contribute?	03/11/2022
Journal Article	Antibiotic resistance and associated resistance determinants in different Salmonella enterica serovars isolated from pigs in Argentina	2022	1215-1220	Vet World	15	5	amr--data--domestic--antibiotic-resistance-and-associated-resistance-determinants-in-different-salmonella-enterica-serovars-isolated-from-pigs-in-argentina	30/06/2022
Journal Article	Changing dietary patterns is necessary to improve the sustainability of Western diets from a One Health perspective	2022	17	Science of the Total Environment	811		domestic--risk--changing-dietary-patterns-is-necessary-to-improve-the-sustainability-of-western-diets-from-a-one-health-perspective	
Journal Article	Production of extended-spectrum beta-lactamases in Escherichia coli isolated from poultry in Rio de Janeiro, Brazil	2022	e001722	Braz J Vet Med	44		amr--data--domestic--production-of-extended-spectrum-beta-lactamases-in-escherichia-coli-isolated-from-poultry-in-rio-de-janeiro,-brazil	20/12/2022
Journal Article	Antimicrobial Resistance in New Zealand-A One Health Perspective	2022		Antibiotics (Basel)	11	6	amr--env--human--opinion--antimicrobial-resistance-in-new-zealand-a-one-health-perspective	25/06/2022
Journal Article	Driving effect of multiplex factors on human brucellosis in high incidence region, implication for brucellosis based on one health concept	2022	100449	One Health	15		pathogen--risk--driving-effect-of-multiplex-factors-on-human-brucellosis-in-high-incidence-region,-implication-for-brucellosis-based-on-one-health-concept	20/12/2022
Journal Article	One Health: An Effective and Ethical Approach to Leptospirosis Control in Australia	2022		Trop Med Infect Dis	7	11	opinion--pathogen--one-health:-an-effective-and-ethical-approach-to-leptospirosis-control-in-australia	25/11/2022
Journal Article	A systematic review and meta-analysis of integrated studies on antimicrobial resistance in Vietnam, with a focus on Enterobacteriaceae, from a One Health perspective	2022	100465	One Health	15		amr--data--domestic--env--human--wild--a-systematic-review-and-meta-analysis-of-integrated-studies-on-antimicrobial-resistance-in-vietnam,-with-a-focus-on-enterobacteriaceae,-from-a-one-health-perspective	24/12/2022
Journal Article	Drug resistant parasites and fungi from a one-health perspective: A global concern that needs transdisciplinary stewardship programs	2022	100368	One Health	14		human--opinion--pathogen--drug-resistant-parasites-and-fungi-from-a-one-health-perspective:-a-global-concern-that-needs-transdisciplinary-stewardship-programs	28/12/2021
Journal Article	Understanding the Interplay between Antimicrobial Resistance, Microplastics and Xenobiotic Contaminants: A Leap towards One Health?	2022		Int J Environ Res Public Health	20	1	amr--opinion--policy--understanding-the-interplay-between-antimicrobial-resistance,-microplastics-and-xenobiotic-contaminants:-a-leap-towards-one-health?	09/01/2023
Journal Article	Pathogenic Escherichia coli, Salmonella spp. and Campylobacter spp. in Two Natural Conservation Centers of Wildlife in Portugal: Genotypic and Phenotypic Characterization	2022	16	Microorganisms	10	11	data--pathogen--wild--pathogenic-escherichia-coli,-salmonella-spp.-and-campylobacter-spp.-in-two-natural-conservation-centers-of-wildlife-in-portugal:-genotypic-and-phenotypic-characterization	
Journal Article	The Animal-Visitor Interaction Protocol (AVIP) for the assessment of Lemur catta walk-in enclosure in zoos	2022	e0271409	PLoS One	17	7	edu--eval--human--pathogen--policy--the-animal-visitor-interaction-protocol-(avip)-for-the-assessment-of-lemur-catta-walk-in-enclosure-in-zoos	29/07/2022
Journal Article	Antimicrobial Resistance and Genetic Diversity of Pseudomonas aeruginosa Strains Isolated from Equine and Other Veterinary Samples	2022		Pathogens	12	1	amr--data--domestic--pathogen--antimicrobial-resistance-and-genetic-diversity-of-pseudomonas-aeruginosa-strains-isolated-from-equine-and-other-veterinary-samples	22/01/2023
Journal Article	Research and Innovation Opportunities to Improve Epidemiological Knowledge and Control of Environmentally Driven Zoonoses	2022	10	Annals of Global Health	88	1	climate--opinion--pathogen--research-and-innovation-opportunities-to-improve-epidemiological-knowledge-and-control-of-environmentally-driven-zoonoses	
Journal Article	Investigation of methicillin-resistant Staphylococcus aureus, methicillin-susceptible Staphylococcus aureus, and Staphylococcus argenteus from wild long-tailed macaques (Macaca fascicularis) at Kosumpee Forest Park, Maha Sarakham, Thailand	2022	2693-2698	Vet World	15	11	amr--data--wild--investigation-of-methicillin-resistant-staphylococcus-aureus,-methicillin-susceptible-staphylococcus-aureus,-and-staphylococcus-argenteus-from-wild-long-tailed-macaques-(macaca-fascicularis)-at-kosumpee-forest-park,-maha-sarakham,-thailand	03/01/2023
Journal Article	What Is in the Salad? Escherichia coli and Antibiotic Resistance in Lettuce Irrigated with Various Water Sources in Ghana	2022		Int J Environ Res Public Health	19	19	data--env--pathogen--what-is-in-the-salad?-escherichia-coli-and-antibiotic-resistance-in-lettuce-irrigated-with-various-water-sources-in-ghana	15/10/2022
Journal Article	Deciphering the genetic network and programmed regulation of antimicrobial resistance in bacterial pathogens	2022	952491	Front Cell Infect Microbiol	12		amr--opinion--deciphering-the-genetic-network-and-programmed-regulation-of-antimicrobial-resistance-in-bacterial-pathogens	13/12/2022
Journal Article	One Health Perspective of Salmonella Serovars in South Africa Using Pooled Prevalence: Systematic Review and Meta-Analysis	2022	8952669	Int J Microbiol	2022		data--domestic--env--human--pathogen--one-health-perspective-of-salmonella-serovars-in-south-africa-using-pooled-prevalence:-systematic-review-and-meta-analysis	03/05/2022
Journal Article	Salmonella Yoruba: A rare serotype revealed through genomic sequencing along the farm-to-fork continuum of an intensive poultry farm in KwaZulu-Natal, South Africa	2022	13	Acta Tropica	234		data--pathogen--salmonella-yoruba:-a-rare-serotype-revealed-through-genomic-sequencing-along-the-farm-to-fork-continuum-of-an-intensive-poultry-farm-in-kwazulu-natal,-south-africa	
Journal Article	Antimicrobial resistance and genetic diversity of Staphylococcus aureus collected from livestock, poultry and humans	2022	100407	One Health	15		amr--data--domestic--human--antimicrobial-resistance-and-genetic-diversity-of-staphylococcus-aureus-collected-from-livestock,-poultry-and-humans	25/10/2022
Journal Article	Healthy planet healthy people	2022	7	Conservation Letters	15	3	eval--policy--healthy-planet-healthy-people	
Journal Article	Tuberculosis caused by Mycobacterium orygis in wild ungulates in Chennai, South India	2022	e3327-e3333	Transbound Emerg Dis	69	5	data--pathogen--wild--tuberculosis-caused-by-mycobacterium-orygis-in-wild-ungulates-in-chennai,-south-india	10/06/2022
Journal Article	Current Insights Regarding the Role of Farm Animals in the Spread of Antimicrobial Resistance from a One Health Perspective	2022	17	Veterinary Sciences	9	9	amr--domestic--human--opinion--current-insights-regarding-the-role-of-farm-animals-in-the-spread-of-antimicrobial-resistance-from-a-one-health-perspective	
Journal Article	High diversity of pathogenic Escherichia coli clones carrying mcr-1 among gulls underlines the need for strategies at the environment-livestock-human interface	2022	4702-4713	Environmental Microbiology	24	10	amr--data--wild--high-diversity-of-pathogenic-escherichia-coli-clones-carrying-mcr-1-among-gulls-underlines-the-need-for-strategies-at-the-environment-livestock-human-interface	
Journal Article	Zoonoses and the Aboriginal and Torres Strait Islander population: A One Health scoping review	2022	e0000921	PLOS Glob Public Health	2	10	eval--pathogen--zoonoses-and-the-aboriginal-and-torres-strait-islander-population:-a-one-health-scoping-review	25/03/2023
Journal Article	Antimicrobial resistance-Do we share more than companionship with our dogs?	2022	1027-1039	J Appl Microbiol	133	2	amr--data--domestic--antimicrobial-resistance-do-we-share-more-than-companionship-with-our-dogs?	22/05/2022
Journal Article	Transmission of Methicillin-Resistant Staphylococcus spp. from Infected Dogs to the Home Environment and Owners	2022		Antibiotics (Basel)	11	5	amr--data--domestic--human--pathogen--transmission-of-methicillin-resistant-staphylococcus-spp.-from-infected-dogs-to-the-home-environment-and-owners	29/05/2022
Journal Article	A Focus on Methodology: A Mixed-Methods Approach to Conduct a Comprehensive Evaluation of the Need for One Health Education for Medical and Veterinary Students in the Context of COVID-19	2022	8	Environmental Health Insights	16		covid--edu--eval--pathogen--a-focus-on-methodology:-a-mixed-methods-approach-to-conduct-a-comprehensive-evaluation-of-the-need-for-one-health-education-for-medical-and-veterinary-students-in-the-context-of-covid-19	
Journal Article	Prevalence, Antibiotic-Resistance, and Replicon-Typing of Salmonella Strains among Serovars Mainly Isolated from Food Chain in Marche Region, Italy	2022	15	Antibiotics-Basel	11	6	data--domestic--human--pathogen--prevalence,-antibiotic-resistance,-and-replicon-typing-of-salmonella-strains-among-serovars-mainly-isolated-from-food-chain-in-marche-region,-italy	
Journal Article	Prevalence and Phenotypic Antimicrobial Resistance among ESKAPE Bacteria and Enterobacterales Strains in Wild Birds	2022	11	Antibiotics-Basel	11	12	amr--data--wild--prevalence-and-phenotypic-antimicrobial-resistance-among-eskape-bacteria-and-enterobacterales-strains-in-wild-birds	
Journal Article	In the beginning it was zoonosis: One Health to combat this and future pandemics. SESPAS Report 2022	2022	S61-S67	Gaceta Sanitaria	36		covid--opinion--in-the-beginning-it-was-zoonosis:-one-health-to-combat-this-and-future-pandemics.-sespas-report-2022	
Journal Article	Genomic and Evolutionary Analysis of Salmonella enterica Serovar Kentucky Sequence Type 198 Isolated From Livestock In East Africa	2022	12	Frontiers in Cellular and Infection Microbiology	12		amr--data--domestic--wild--genomic-and-evolutionary-analysis-of-salmonella-enterica-serovar-kentucky-sequence-type-198-isolated-from-livestock-in-east-africa	
Journal Article	Antimicrobial resistance in the globalized food chain: a One Health perspective applied to the poultry industry	2022	465-486	Brazilian Journal of Microbiology	53	1	amr--domestic--opinion--antimicrobial-resistance-in-the-globalized-food-chain:-a-one-health-perspective-applied-to-the-poultry-industry	
Journal Article	Slaughterhouse wastewater as a reservoir for extended-spectrum β-lactamase (ESBL)-producing, and colistin-resistant Klebsiella spp. and their impact in a "One Health" perspective	2022	150000	Sci Total Environ	804		data--domestic--env--pathogen--slaughterhouse-wastewater-as-a-reservoir-for-extended-spectrum-β-lactamase-(esbl)-producing,-and-colistin-resistant-klebsiella-spp.-and-their-impact-in-a-"one-health"-perspective	14/09/2021
Journal Article	Strengthening the global one health workforce: Veterinarians in CDC-supported field epidemiology training programs	2022	7	One Health	14		data--edu--eval--human--strengthening-the-global-one-health-workforce:-veterinarians-in-cdc-supported-field-epidemiology-training-programs	
Journal Article	The significance of biofilms to human, animal, plant and ecosystem health	2022	294-313	Functional Ecology	36	2	opinion--pathogen--the-significance-of-biofilms-to-human,-animal,-plant-and-ecosystem-health	
Journal Article	Antimicrobial resistance and One Health	2022	37-40	Revista Espanola De Quimioterapia	35		amr--opinion--policy--antimicrobial-resistance-and-one-health	
Journal Article	Environmental Contamination by Echinococcus spp. Eggs as a Risk for Human Health in Educational Farms of Sardinia, Italy	2022	11	Veterinary Sciences	9	3	data--domestic--pathogen--environmental-contamination-by-echinococcus-spp.-eggs-as-a-risk-for-human-health-in-educational-farms-of-sardinia,-italy	
Journal Article	The concept of one health applied to the problem of zoonotic diseases	2022	14	Reviews in Medical Virology	32	4	opinion--pathogen--the-concept-of-one-health-applied-to-the-problem-of-zoonotic-diseases	
Journal Article	A call to prioritise prevention: Action is needed to reduce the risk of zoonotic disease emergence	2022	6	Lancet Regional Health-Europe	23		opinion--pathogen--policy--a-call-to-prioritise-prevention:-action-is-needed-to-reduce-the-risk-of-zoonotic-disease-emergence	
Journal Article	Interventions to optimize the use of antibiotics in China: A scoping review of evidence from humans, animals, and the environment from a One Health perspective	2022	10	One Health	14		amr--data--eval--interventions-to-optimize-the-use-of-antibiotics-in-china:-a-scoping-review-of-evidence-from-humans,-animals,-and-the-environment-from-a-one-health-perspective	
Journal Article	The risk of reverse zoonotic transmission to pet animals during the current global monkeypox outbreak, United Kingdom, June to mid-September 2022	2022		Euro Surveill	27	39	data--domestic--pathogen--the-risk-of-reverse-zoonotic-transmission-to-pet-animals-during-the-current-global-monkeypox-outbreak,-united-kingdom,-june-to-mid-september-2022	01/10/2022
Journal Article	Contamination Status of Pet Cats in Thailand with Organohalogen Compounds (OHCs) and Their Hydroxylated and Methoxylated Derivatives and Estimation of Sources of Exposure to These Contaminants	2022		Animals (Basel)	12	24	domestic--non-bio--opinion--contamination-status-of-pet-cats-in-thailand-with-organohalogen-compounds-(ohcs)-and-their-hydroxylated-and-methoxylated-derivatives-and-estimation-of-sources-of-exposure-to-these-contaminants	24/12/2022
Journal Article	Estimation of the Effects of Neonicotinoid Insecticides on Wild Raccoon, Procyon lotor, in Hokkaido, Japan: Urinary Concentrations and Hepatic Metabolic Capability of Neonicotinoids	2022	1865-1874	Environ Toxicol Chem	41	8	data--non-bio--wild--estimation-of-the-effects-of-neonicotinoid-insecticides-on-wild-raccoon,-procyon-lotor,-in-hokkaido,-japan:-urinary-concentrations-and-hepatic-metabolic-capability-of-neonicotinoids	23/04/2022
Journal Article	Characteristics of cytochrome P450-dependent metabolism in the liver of the wild raccoon, Procyon lotor	2022	1665-1672	J Vet Med Sci	84	12	data--non-bio--wild--characteristics-of-cytochrome-p450-dependent-metabolism-in-the-liver-of-the-wild-raccoon,-procyon-lotor	04/11/2022
Journal Article	Viral infections in orthopedics: A systematic review and classification proposal	2022	1015-1028	World J Orthop	13	11	data--domestic--human--pathogen--viral-infections-in-orthopedics:-a-systematic-review-and-classification-proposal	29/11/2022
Journal Article	Trace Elements in Beef Cattle: A Review of the Scientific Approach from One Health Perspective	2022	15	Animals	12	17	data--domestic--eval--non-bio--trace-elements-in-beef-cattle:-a-review-of-the-scientific-approach-from-one-health-perspective	
Journal Article	Pathological Findings in African Pygmy Hedgehogs Admitted into a Portuguese Rehabilitation Center	2022	17	Animals	12	11	data--opinion--pathogen--wild--pathological-findings-in-african-pygmy-hedgehogs-admitted-into-a-portuguese-rehabilitation-center	
Journal Article	Geodemography, environment and societal characteristics drive the global diversity of emerging, zoonotic and human pathogens	2022	1131-1143	Transbound Emerg Dis	69	3	eval--pathogen--policy--risk--geodemography,-environment-and-societal-characteristics-drive-the-global-diversity-of-emerging,-zoonotic-and-human-pathogens	17/03/2021

Journal Article	Microbial community and antimicrobial resistance in fecal samples from wild and domestic ruminants in Maiella National Park, Italy	2022	100403	One Health	15		amr--data--domestic--wild--microbial-community-and-antimicrobial-resistance-in-fecal-samples-from-wild-and-domestic-ruminants-in-maiella-national-park,-italy	02/06/2022
Journal Article	Resistance Patterns, mcr-4 and OXA-48 Genes, and Virulence Factors of Escherichia coli from Apennine Chamois Living in Sympatry with Domestic Species, Italy	2022	12	Animals	12	2	amr--data--wild--resistance-patterns,-mcr-4-and-oxa-48-genes,-and-virulence-factors-of-escherichia-coli-from-apennine-chamois-living-in-sympatry-with-domestic-species,-italy	
Journal Article	Molecular Epidemiology of Antibiotic Resistance Genes and Virulence Factors in Multidrug-Resistant Escherichia coli Isolated from Rodents, Humans, Chicken, and Household Soils in Karatu, Northern Tanzania	2022		Int J Environ Res Public Health	19	9	amr--data--domestic--env--human--wild--molecular-epidemiology-of-antibiotic-resistance-genes-and-virulence-factors-in-multidrug-resistant-escherichia-coli-isolated-from-rodents,-humans,-chicken,-and-household-soils-in-karatu,-northern-tanzania	15/05/2022
Journal Article	Participation in One Health Networks and Involvement in the COVID-19 Pandemic Response: A Global Study	2022	10	Frontiers in Public Health	10		covid--eval--participation-in-one-health-networks-and-involvement-in-the-covid-19-pandemic-response:-a-global-study	
Journal Article	One Health Surveillance of Antimicrobial Resistance Phenotypes in Selected Communities in Thailand	2022		Antibiotics (Basel)	11	5	amr--data--domestic--wild--one-health-surveillance-of-antimicrobial-resistance-phenotypes-in-selected-communities-in-thailand	29/05/2022
Journal Article	Aggressiveness, ADHD-like behaviour, and environment influence repetitive behaviour in dogs	2022	10	Scientific Reports	12	1	data--domestic--aggressiveness,-adhd-like-behaviour,-and-environment-influence-repetitive-behaviour-in-dogs	
Journal Article	Prevalence and genetic diversity of Giardia duodenalis in pet dogs from Zhengzhou, central China and the association between gut microbiota and fecal characteristics during infection	2022	100401	One Health	14		data--domestic--pathogen--prevalence-and-genetic-diversity-of-giardia-duodenalis-in-pet-dogs-from-zhengzhou,-central-china-and-the-association-between-gut-microbiota-and-fecal-characteristics-during-infection	11/06/2022
Journal Article	A One Health real-time surveillance system for nowcasting Campylobacter gastrointestinal illness outbreaks, Norway, week 30 2010 to week 11 2022	2022		Euro Surveill	27	43	domestic--opinion--pathogen--a-one-health-real-time-surveillance-system-for-nowcasting-campylobacter-gastrointestinal-illness-outbreaks,-norway,-week-30-2010-to-week-11-2022	29/10/2022
Journal Article	Human Colonization with Antibiotic-Resistant Bacteria from Nonoccupational Exposure to Domesticated Animals in Low- and Middle-Income Countries: A Critical Review	2022	14875-14890	Environmental Science & Technology	56	21	amr--data--domestic--env--human--policy--human-colonization-with-antibiotic-resistant-bacteria-from-nonoccupational-exposure-to-domesticated-animals-in-low--and-middle-income-countries:-a-critical-review	
Journal Article	Differential Overlap in Human and Animal Fecal Microbiomes and Resistomes in Rural versus Urban Bangladesh	2022	18	Applied and Environmental Microbiology	88	14	amr--data--domestic--human--pathogen--differential-overlap-in-human-and-animal-fecal-microbiomes-and-resistomes-in-rural-versus-urban-bangladesh	
Journal Article	Understanding leptospirosis: application of state-of-the-art molecular typing tools with a One Health lens	2022		Am J Vet Res	83	10	opinion--pathogen--understanding-leptospirosis:-application-of-state-of-the-art-molecular-typing-tools-with-a-one-health-lens	21/08/2022
Journal Article	A global one health perspective on leptospirosis in humans and animals	2022	1589-1596	Javma-Journal of the American Veterinary Medical Association	260	13	climate--opinion--pathogen--a-global-one-health-perspective-on-leptospirosis-in-humans-and-animals	
Journal Article	A Rapid Review of Environmental Health Gaps in Antimicrobial Resistance and Water-Related Research from 1990-2020	2022		Int J Environ Res Public Health	19	11	env--eval--wild--a-rapid-review-of-environmental-health-gaps-in-antimicrobial-resistance-and-water-related-research-from-1990-2020	11/06/2022
Journal Article	Faecal carriage of ESBL producing and colistin resistant Escherichia coli in avian species over a 2-year period (2017-2019) in Zimbabwe	2022	1035145	Front Cell Infect Microbiol	12		amr--data--domestic--faecal-carriage-of-esbl-producing-and-colistin-resistant-escherichia-coli-in-avian-species-over-a-2-year-period-(2017-2019)-in-zimbabwe	10/01/2023
Journal Article	Toxicokinetic analysis of the anticoagulant rodenticides warfarin & diphacinone in Egyptian fruit bats (Rousettus aegyptiacus) as a comparative sensitivity assessment for Bonin fruit bats (Pteropus pselaphon)	2022	113971	Ecotoxicol Environ Saf	243		data--non-bio--wild--toxicokinetic-analysis-of-the-anticoagulant-rodenticides-warfarin-&-diphacinone-in-egyptian-fruit-bats-(rousettus-aegyptiacus)-as-a-comparative-sensitivity-assessment-for-bonin-fruit-bats-(pteropus-pselaphon)	19/08/2022
Journal Article	Combating the global spread of poverty-related Monkeypox outbreaks and beyond	2022	5	Infectious Diseases of Poverty	11	1	opinion--pathogen--combating-the-global-spread-of-poverty-related-monkeypox-outbreaks-and-beyond	
Journal Article	INITIAL ASSESSMENT OF THE GENETIC DIVERSITY OF THE LONG-TAILED MACAQUES (MACACA FASCICULARIS) AT KOSUMPEE FOREST PARK, THAILAND	2022	945-953	Journal of Animal and Plant Sciences-Japs	32	4	data--wild--initial-assessment-of-the-genetic-diversity-of-the-long-tailed-macaques-(macaca-fascicularis)-at-kosumpee-forest-park,-thailand	
Journal Article	An Overview of Anthropogenic Actions as Drivers for Emerging and Re-Emerging Zoonotic Diseases	2022		Pathogens	11	11	climate--opinion--pathogen--an-overview-of-anthropogenic-actions-as-drivers-for-emerging-and-re-emerging-zoonotic-diseases	25/11/2022
Journal Article	Novel variant Hendra virus genotype 2 infection in a horse in the greater Newcastle region, New South Wales, Australia	2022	7	One Health	15		domestic--eval--pathogen--novel-variant-hendra-virus-genotype-2-infection-in-a-horse-in-the-greater-newcastle-region,-new-south-wales,-australia	
Journal Article	Distribution of Theileria orientalis in Virginia Market Cattle, 2018-2020	2022		Pathogens	11	11	data--domestic--pathogen--distribution-of-theileria-orientalis-in-virginia-market-cattle,-2018-2020	25/11/2022
Journal Article	A large-scale genomic snapshot of Klebsiella spp. isolates in Northern Italy reveals limited transmission between clinical and non-clinical settings	2022	2054-2067	Nat Microbiol	7	12	data--domestic--env--human--pathogen--a-large-scale-genomic-snapshot-of-klebsiella-spp.-isolates-in-northern-italy-reveals-limited-transmission-between-clinical-and-non-clinical-settings	22/11/2022
Journal Article	Microbes and Climate Change: a Research Prospectus for the Future	2022	e0080022	mBio	13	3	climate--opinion--microbes-and-climate-change:-a-research-prospectus-for-the-future	20/04/2022
Journal Article	Wastewater surveillance of antibiotic-resistant bacterial pathogens: A systematic review	2022	19	Frontiers in Microbiology	13		amr--data--pathogen--wastewater-surveillance-of-antibiotic-resistant-bacterial-pathogens:-a-systematic-review	
Journal Article	Clostridioides difficile positivity rate and PCR ribotype distribution on retail potatoes in 12 European countries, January to June 2018	2022		Euro Surveill	27	15	data--domestic--pathogen--clostridioides-difficile-positivity-rate-and-pcr-ribotype-distribution-on-retail-potatoes-in-12-european-countries,-january-to-june-2018	16/04/2022
Journal Article	Factors affecting preventive behavior against leptospirosis among the population at risk in Si Sa Ket, Thailand	2022	7	One Health	14		data--human--pathogen--policy--factors-affecting-preventive-behavior-against-leptospirosis-among-the-population-at-risk-in-si-sa-ket,-thailand	
Journal Article	Data report on three datasets: Mortality patterns between agricultural and non-agricultural ward areas	2022	953167	Front Genet	13		data--domestic--data-report-on-three-datasets:-mortality-patterns-between-agricultural-and-non-agricultural-ward-areas	24/01/2023
Journal Article	Occurrence of Antimicrobial Resistance in the Environment in Germany, Austria, and Switzerland: A Narrative Review of Existing Evidence	2022	19	Microorganisms	10	4	amr--env--opinion--occurrence-of-antimicrobial-resistance-in-the-environment-in-germany,-austria,-and-switzerland:-a-narrative-review-of-existing-evidence	
Journal Article	The emergence of Clostridioides difficile PCR ribotype 127 at a hospital in northeastern Taiwan	2022	896-909	Journal of Microbiology Immunology and Infection	55	5	data--human--pathogen--the-emergence-of-clostridioides-difficile-pcr-ribotype-127-at-a-hospital-in-northeastern-taiwan	
Journal Article	Evaluating the contribution of a wildlife health capacity building program on orangutan conservation	2022	13	American Journal of Primatology	84	4-5	evaluation--policy--wild--evaluating-the-contribution-of-a-wildlife-health-capacity-building-program-on-orangutan-conservation	
Journal Article	Current status and future perspective of antimicrobial-resistant bacteria and resistance genes in animal-breeding environments	2022	1292-1298	J Vet Med Sci	84	9	amr--domestic--env--opinion--pathogen--wild--current-status-and-future-perspective-of-antimicrobial-resistant-bacteria-and-resistance-genes-in-animal-breeding-environments	25/07/2022
Journal Article	Signalling and responding to zoonotic threats using a One Health approach: a decade of the Zoonoses Structure in the Netherlands, 2011 to 2021	2022		Euro Surveill	27	31	opinion--pathogen--policy--signalling-and-responding-to-zoonotic-threats-using-a-one-health-approach:-a-decade-of-the-zoonoses-structure-in-the-netherlands,-2011-to-2021	06/08/2022
Journal Article	Implications of Veterinary Medicine in the comprehension and stewardship of antimicrobial resistance phenomenon. From the origin till nowadays	2022	12	Veterinary and Animal Science	16		amr--domestic--opinion--implications-of-veterinary-medicine-in-the-comprehension-and-stewardship-of-antimicrobial-resistance-phenomenon.-from-the-origin-till-nowadays	
Journal Article	One Health, One Welfare, One Right: Introducing Animal Rights in Europe	2022	277-310	Journal for European Environmental & Planning Law	19	4	opinion--policy--wild--one-health,-one-welfare,-one-right:-introducing-animal-rights-in-europe	
Journal Article	The role of religion in One Health. Lessons from the Hanuman langur (Semnopithecus entellus) and other human-non-human primate interactions	2022	10	American Journal of Primatology	84	4-5	covid--opinion--pathogen--wild--the-role-of-religion-in-one-health.-lessons-from-the-hanuman-langur-(semnopithecus-entellus)-and-other-human-non-human-primate-interactions	
Journal Article	COVID-19 and zoonoses in Brazil: Environmental scan of one health preparedness and response	2022	9	One Health	14		covid--data--policy--covid-19-and-zoonoses-in-brazil:-environmental-scan-of-one-health-preparedness-and-response	
Journal Article	Governance Processes and Challenges for Reservation of Antimicrobials Exclusively for Human Use and Restriction of Antimicrobial Use in Animals	2022	55-63	Journal of Law Medicine & Ethics	50		amr--opinion--governance-processes-and-challenges-for-reservation-of-antimicrobials-exclusively-for-human-use-and-restriction-of-antimicrobial-use-in-animals	
Journal Article	Averting wildlife-borne infectious disease epidemics requires a focus on socio-ecological drivers and a redesign of the global food system	2022	32	EClinicalMedicine	47		opinion--policy--wild--averting-wildlife-borne-infectious-disease-epidemics-requires-a-focus-on-socio-ecological-drivers-and-a-redesign-of-the-global-food-system	
Journal Article	A systematic literature review on the economic impact of endemic disease in UK sheep and cattle using a One Health conceptualisation	2022	9	Preventive Veterinary Medicine	209		data--domestic--pathogen--a-systematic-literature-review-on-the-economic-impact-of-endemic-disease-in-uk-sheep-and-cattle-using-a-one-health-conceptualisation	
Journal Article	High contamination rates of shoes of veterinarians, veterinary support staff and veterinary students with Clostridioides difficile spores	2022	685-693	Transbound Emerg Dis	69	2	data--human--pathogen--high-contamination-rates-of-shoes-of-veterinarians,-veterinary-support-staff-and-veterinary-students-with-clostridioides-difficile-spores	10/02/2021
Journal Article	An argument for pandemic risk management using a multidisciplinary One Health approach to governance: an Australian case study	2022	12	Globalization and Health	18	1	covid--opinion--policy--an-argument-for-pandemic-risk-management-using-a-multidisciplinary-one-health-approach-to-governance:-an-australian-case-study	
Journal Article	A One Health strategy for emerging infectious diseases based on the COVID-19 outbreak	2022	5-11	J Biosaf Biosecur	4	1	covid--opinion--a-one-health-strategy-for-emerging-infectious-diseases-based-on-the-covid-19-outbreak	04/11/2021
Journal Article	Prevalence, molecular characterization, and antimicrobial resistance profile of Clostridium perfringens from India: A scoping review	2022	102639	Anaerobe	77		amr--data--domestic--env--pathogen--prevalence,-molecular-characterization,-and-antimicrobial-resistance-profile-of-clostridium-perfringens-from-india:-a-scoping-review	16/09/2022
Journal Article	Dissemination of bla(NDM-5) and mcr-8.1 in carbapenem-resistant Klebsiella pneumoniae and Klebsiella quasipneumoniae in an animal breeding area in Eastern China	2022	12	Frontiers in Microbiology	13		amr--data--domestic--env--human--pathogen--dissemination-of-bla(ndm-5)-and-mcr-8.1-in-carbapenem-resistant-klebsiella-pneumoniae-and-klebsiella-quasipneumoniae-in-an-animal-breeding-area-in-eastern-china	
Journal Article	One Health Paradigm to Confront Zoonotic Health Threats: A Pakistan Prospective	2022	11	Frontiers in Microbiology	12		opinion--one-health-paradigm-to-confront-zoonotic-health-threats:-a-pakistan-prospective	
Journal Article	Spatiotemporal risk assessment for avian influenza outbreak based on the dynamics of habitat suitability for wild birds	2022	e953-e967	Transbound Emerg Dis	69	4	pathogen--risk--wild--spatiotemporal-risk-assessment-for-avian-influenza-outbreak-based-on-the-dynamics-of-habitat-suitability-for-wild-birds	06/11/2021
Journal Article	Survivability of Kluyveromyces marxianus Isolated From Korean Kefir in a Simulated Gastrointestinal Environment	2022	842097	Front Microbiol	13		data--survivability-of-kluyveromyces-marxianus-isolated-from-korean-kefir-in-a-simulated-gastrointestinal-environment	15/03/2022
Journal Article	Antibiotic resistance genes of public health importance in livestock and humans in an informal urban community in Nepal	2022	13	Scientific Reports	12	1	amr--data--domestic--human--wild--antibiotic-resistance-genes-of-public-health-importance-in-livestock-and-humans-in-an-informal-urban-community-in-nepal	
Journal Article	Toward the Adoption of Loop-Mediated Isothermal Amplification for Salmonella Screening at the National Antimicrobial Resistance Monitoring System's Retail Meat Sites	2022	758-766	Foodborne Pathogens and Disease	19	11	amr--data--domestic--pathogen--toward-the-adoption-of-loop-mediated-isothermal-amplification-for-salmonella-screening-at-the-national-antimicrobial-resistance-monitoring-system's-retail-meat-sites	
Journal Article	Naturalized Escherichia coli in Wastewater and the Co-evolution of Bacterial Resistance to Water Treatment and Antibiotics	2022	810312	Front Microbiol	13		opinion--pathogen--naturalized-escherichia-coli-in-wastewater-and-the-co-evolution-of-bacterial-resistance-to-water-treatment-and-antibiotics	17/06/2022
Journal Article	The association between natural drinking water sources and the emergence of zoonotic leptospirosis among grazing beef cattle herds during a human outbreak	2022	100372	One Health	14		data--domestic--pathogen--the-association-between-natural-drinking-water-sources-and-the-emergence-of-zoonotic-leptospirosis-among-grazing-beef-cattle-herds-during-a-human-outbreak	12/02/2022
Journal Article	Minimizing Risks of Antimicrobial Resistance Development in the Environment from a Public One Health Perspective	2022	1105-1109	China CDC Wkly	4	49	amr--opinion--policy--minimizing-risks-of-antimicrobial-resistance-development-in-the-environment-from-a-public-one-health-perspective	09/02/2023
Journal Article	Low Clostridioides difficile positivity rate in wild animal shelter in Slovenia	2022	102643	Anaerobe	77		data--pathogen--wild--low-clostridioides-difficile-positivity-rate-in-wild-animal-shelter-in-slovenia	17/09/2022
Journal Article	A One-Health Sampling Strategy to Explore the Dissemination and Relationship Between Colistin Resistance in Human, Animal, and Environmental Sectors in Laos	2022	45-56	Engineering	15		data--domestic--env--human--pathogen--wild--a-one-health-sampling-strategy-to-explore-the-dissemination-and-relationship-between-colistin-resistance-in-human,-animal,-and-environmental-sectors-in-laos	

Supplementary figure 1

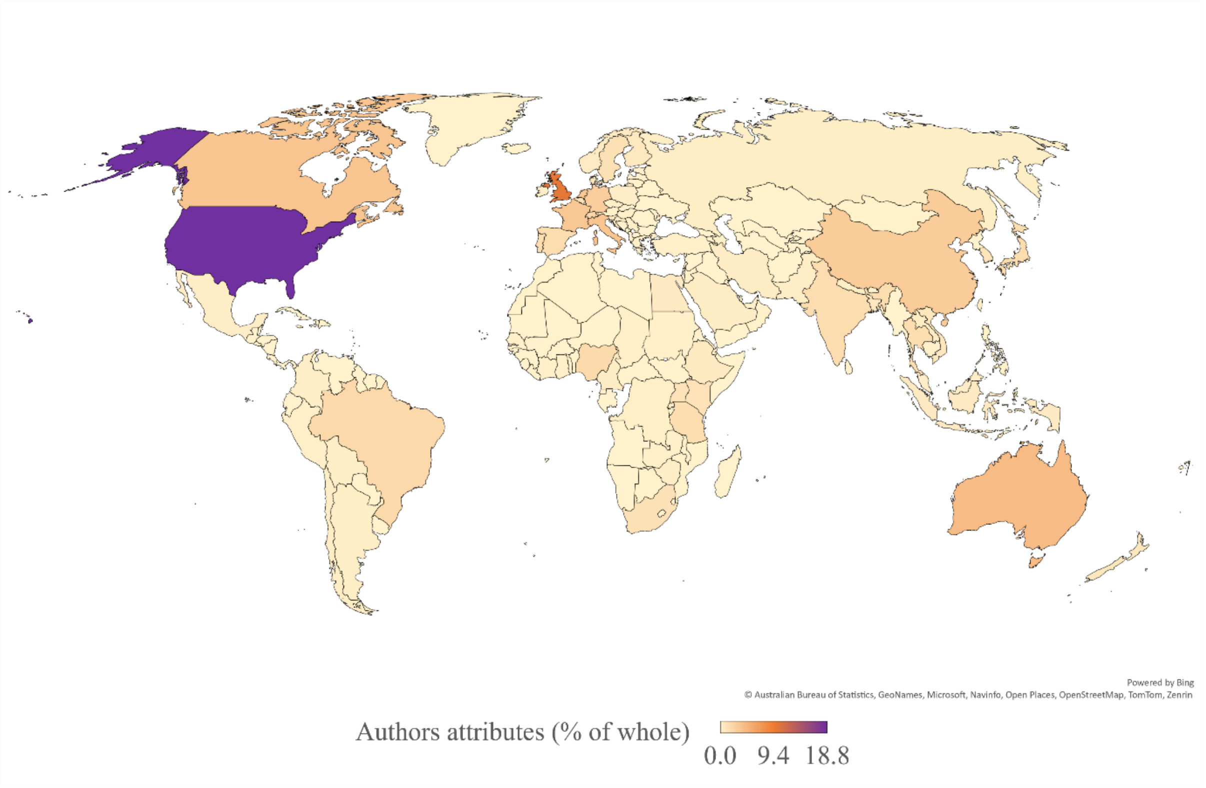

Supplementary figure 1. Global map showing the percentage of all authors (as a total of all the OH manuscripts) affiliated with each country.
